# Supplementary material for: A Novel Modulator of Resistance for Oxaliplatin-Based Therapy for Colorectal Cancer: The ESCRT Family Member VPS4A
Source: Cells. 2025 Jun 19;14(12):929. doi: 10.3390/cells14120929 (PMC12190599; doi:10.3390/cells14120929)
Supplement: Supplementary file 1 [file cells-14-00929-s001.zip › cells-3673677-supplementary.pdf]

Supplementary Figure S1

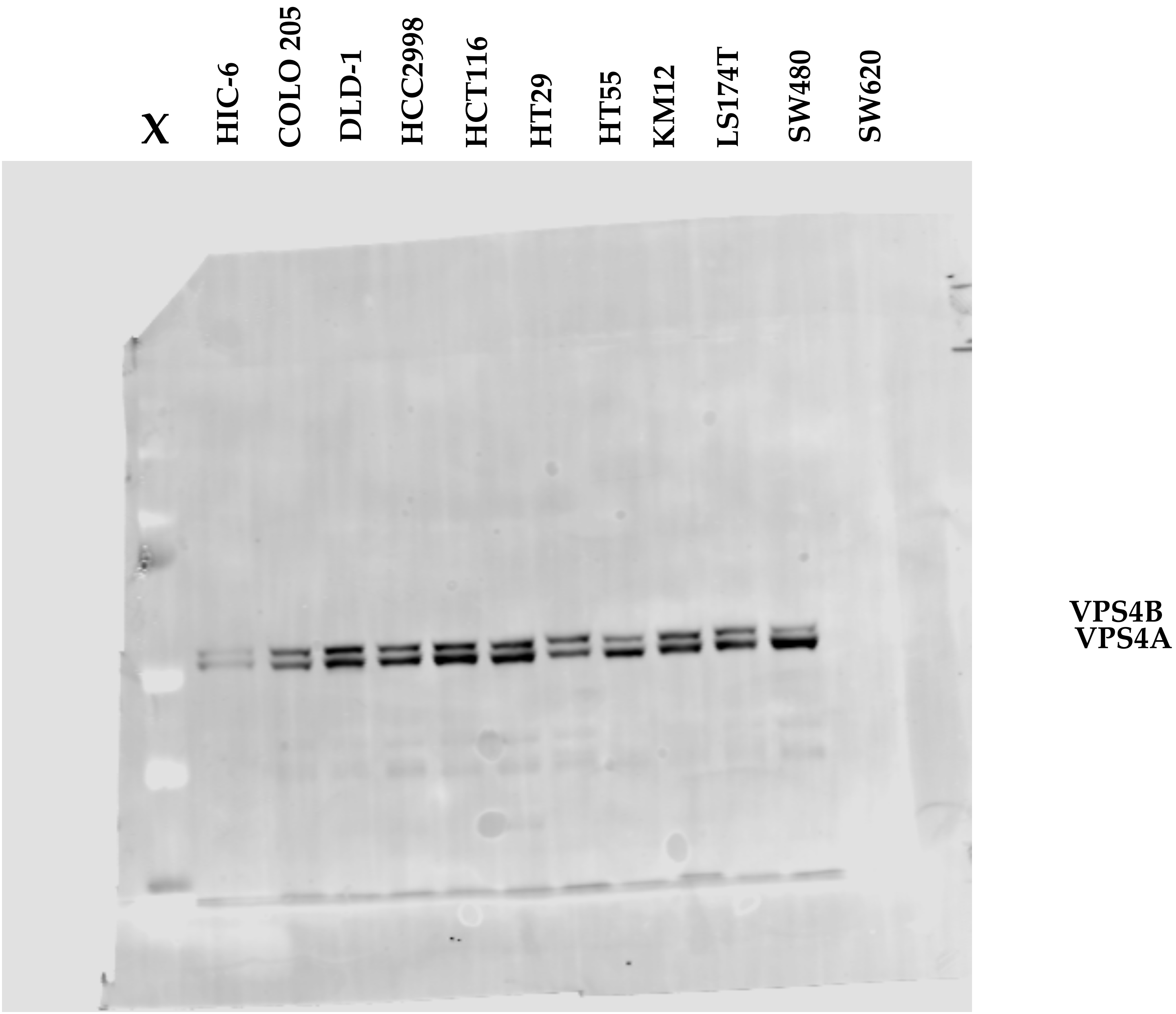

S1. Characterisation of ESCRT protein expression in a panel of human CRC cell lines using Western immunoblotting

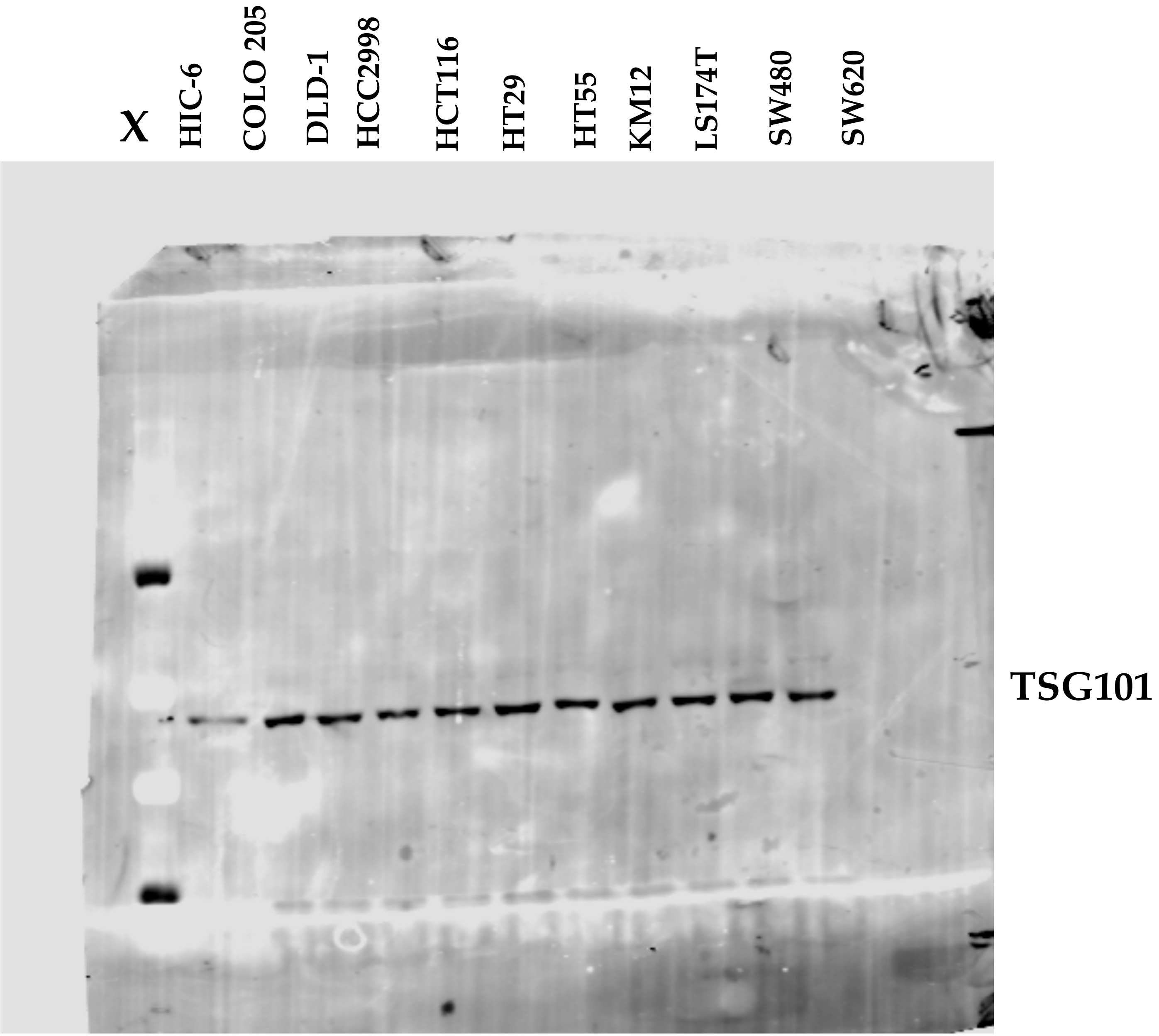

S1. Characterisation of ESCRT protein expression in a panel of human CRC cell lines using Western immunoblotting

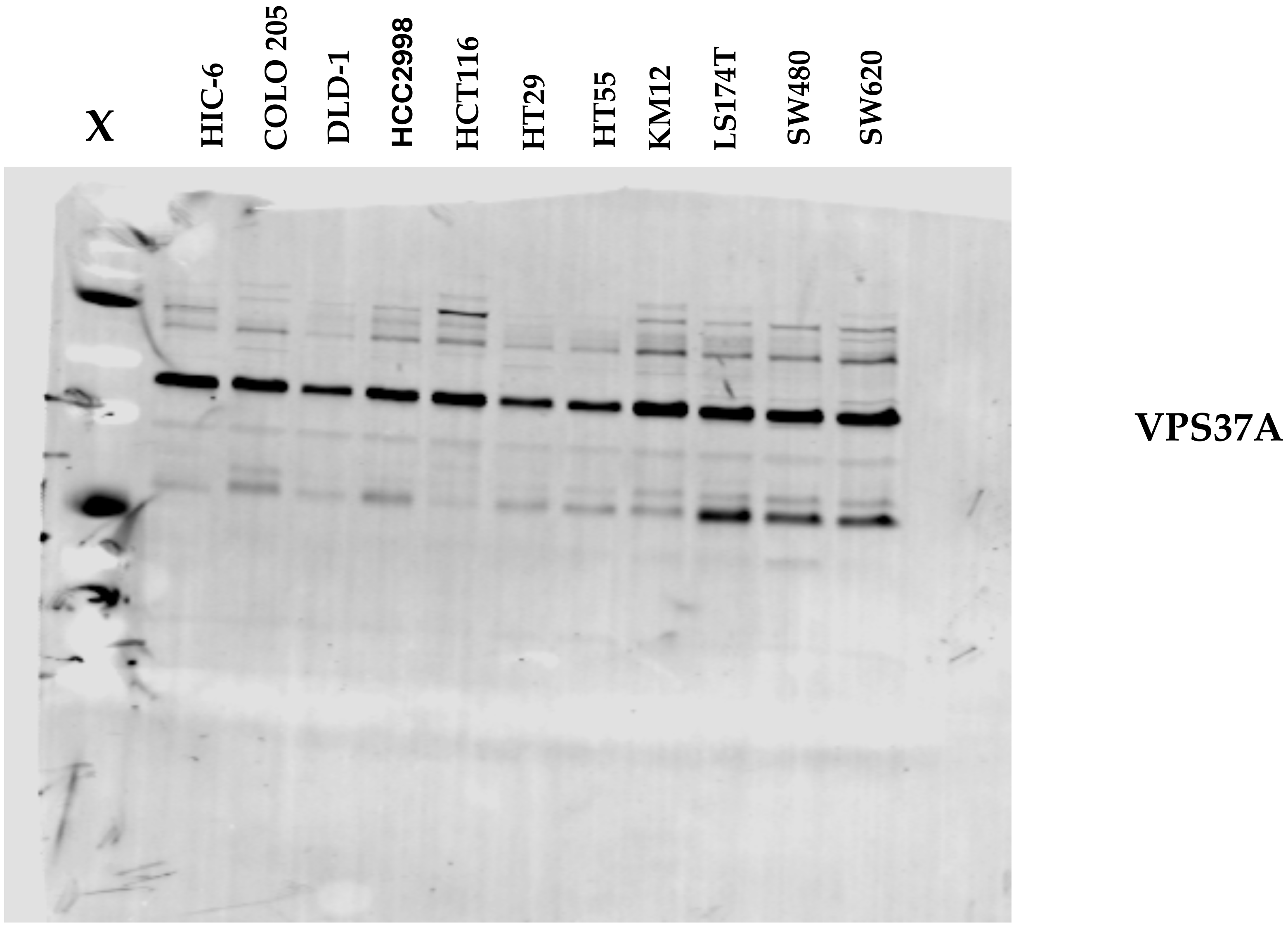

S1. Characterisation of ESCRT protein expression in a panel of human CRC cell lines using Western immunoblotting

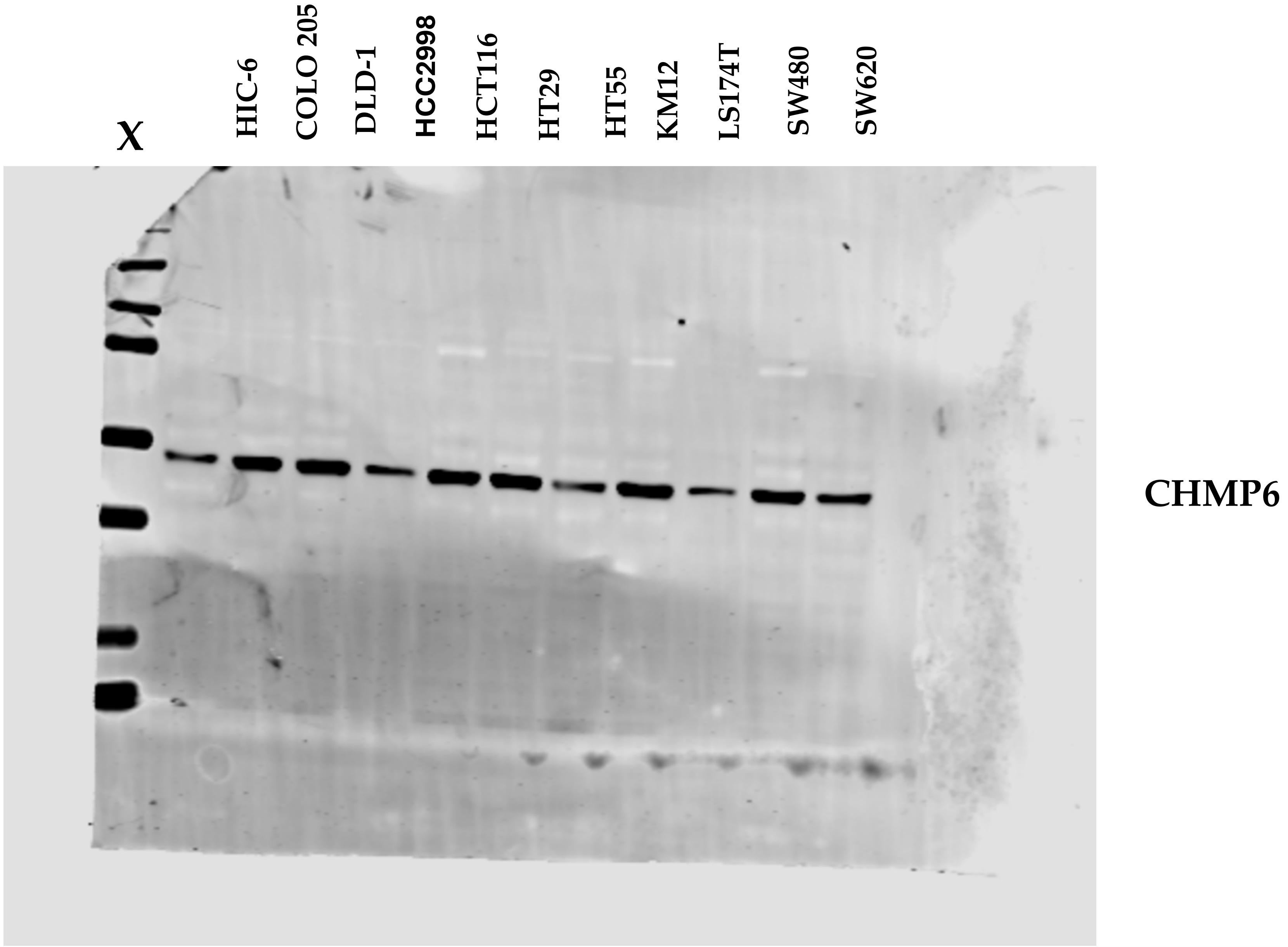

S1. Characterisation of ESCRT protein expression in a panel of human CRC cell lines using Western immunoblotting

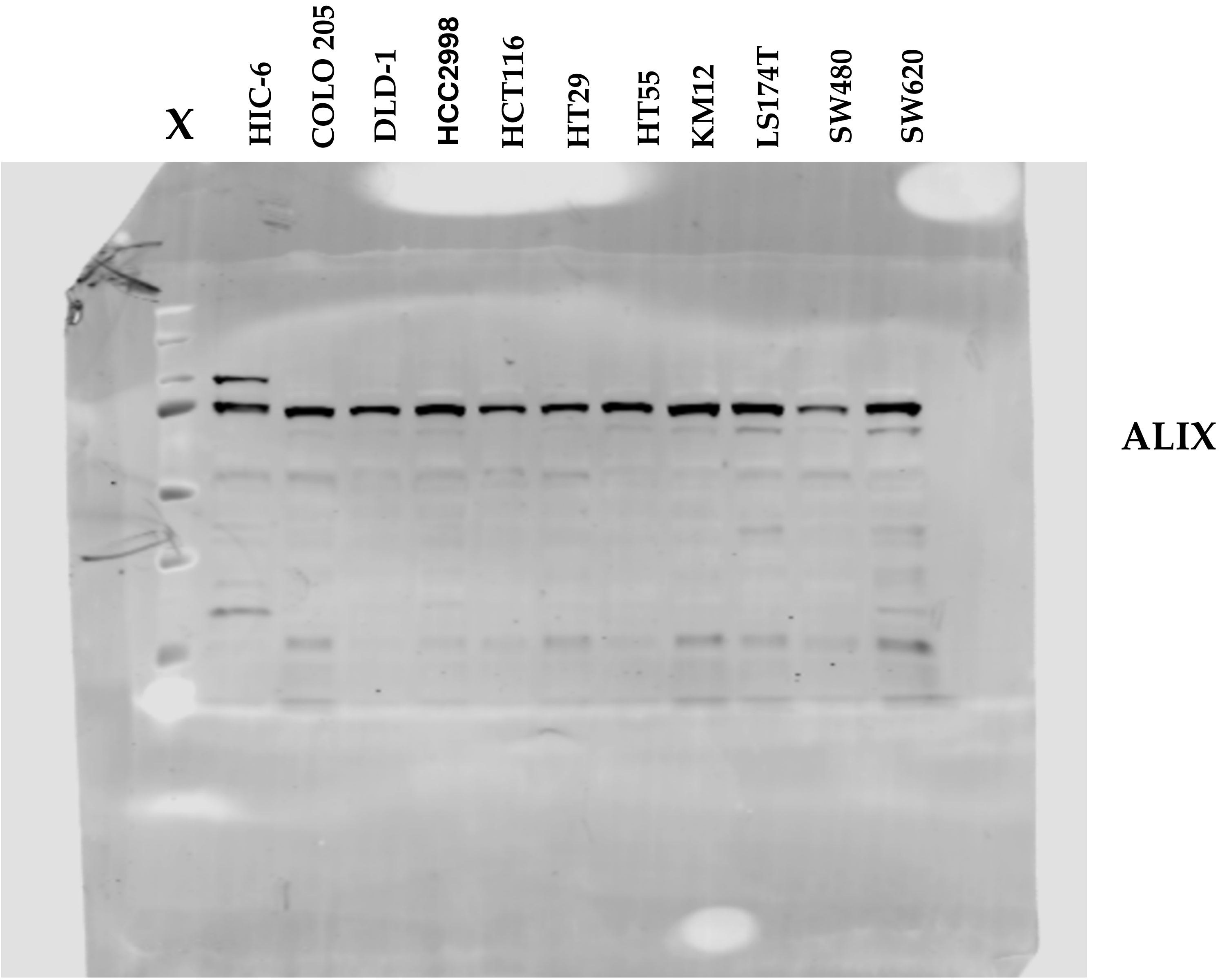

S1. Characterisation of ESCRT protein expression in a panel of human CRC cell lines using Western immunoblotting

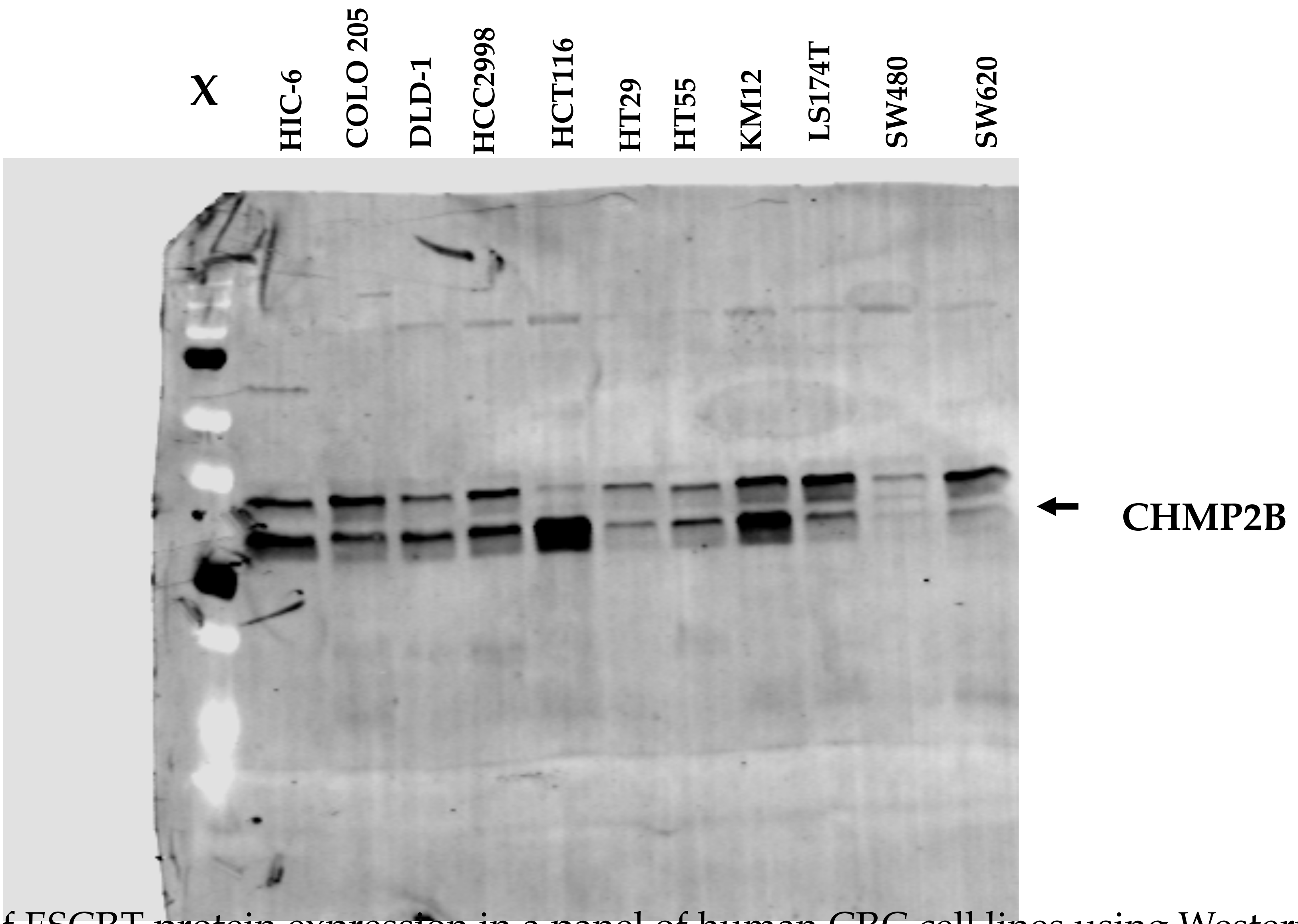

S1. Characterisation of ESCRT protein expression in a panel of human CRC cell lines using Western immunoblotting

Sample 4 cropped and re-added

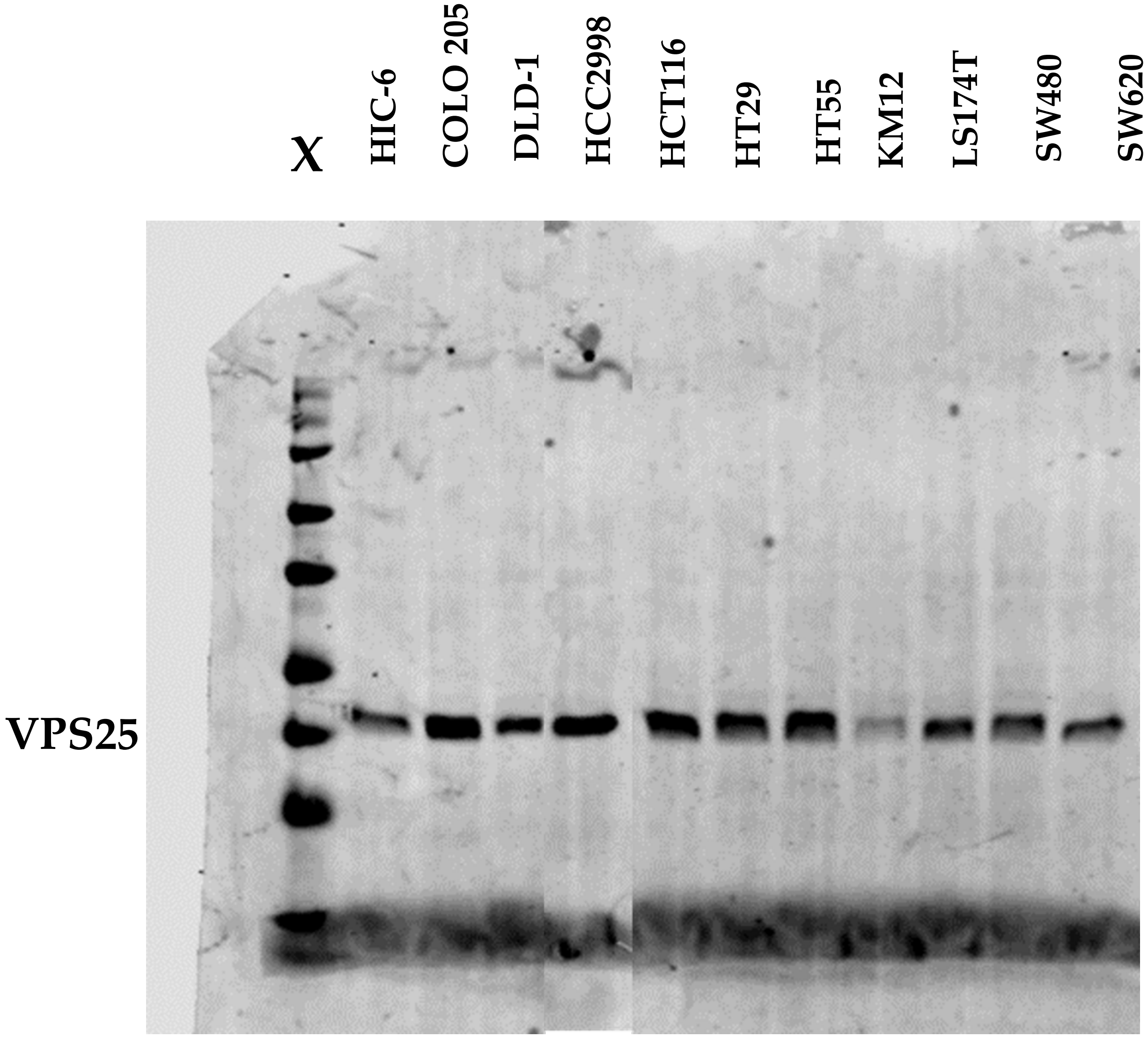

Original

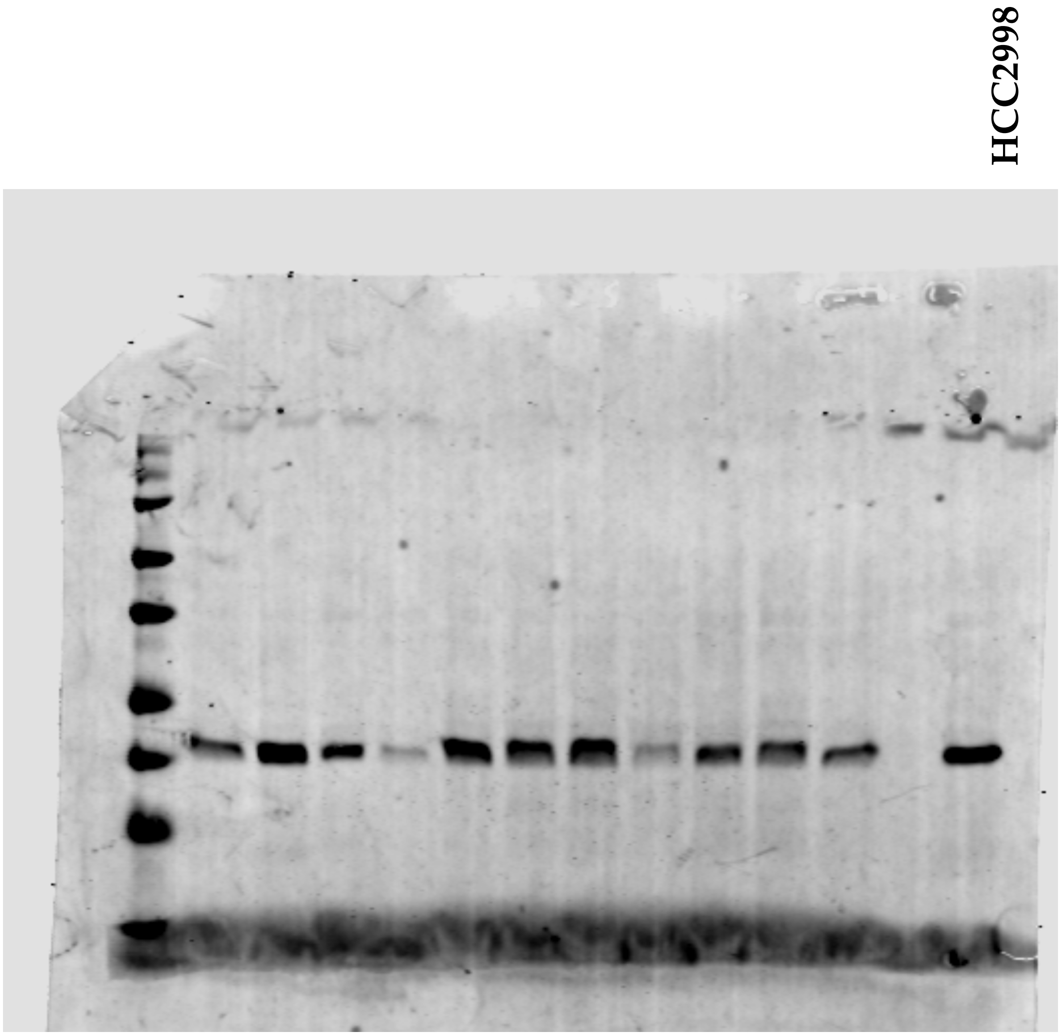

Sample 4 was miss loaded so reloaded in the end of the membrane and membrane cropped to add correct loaded Sample in place

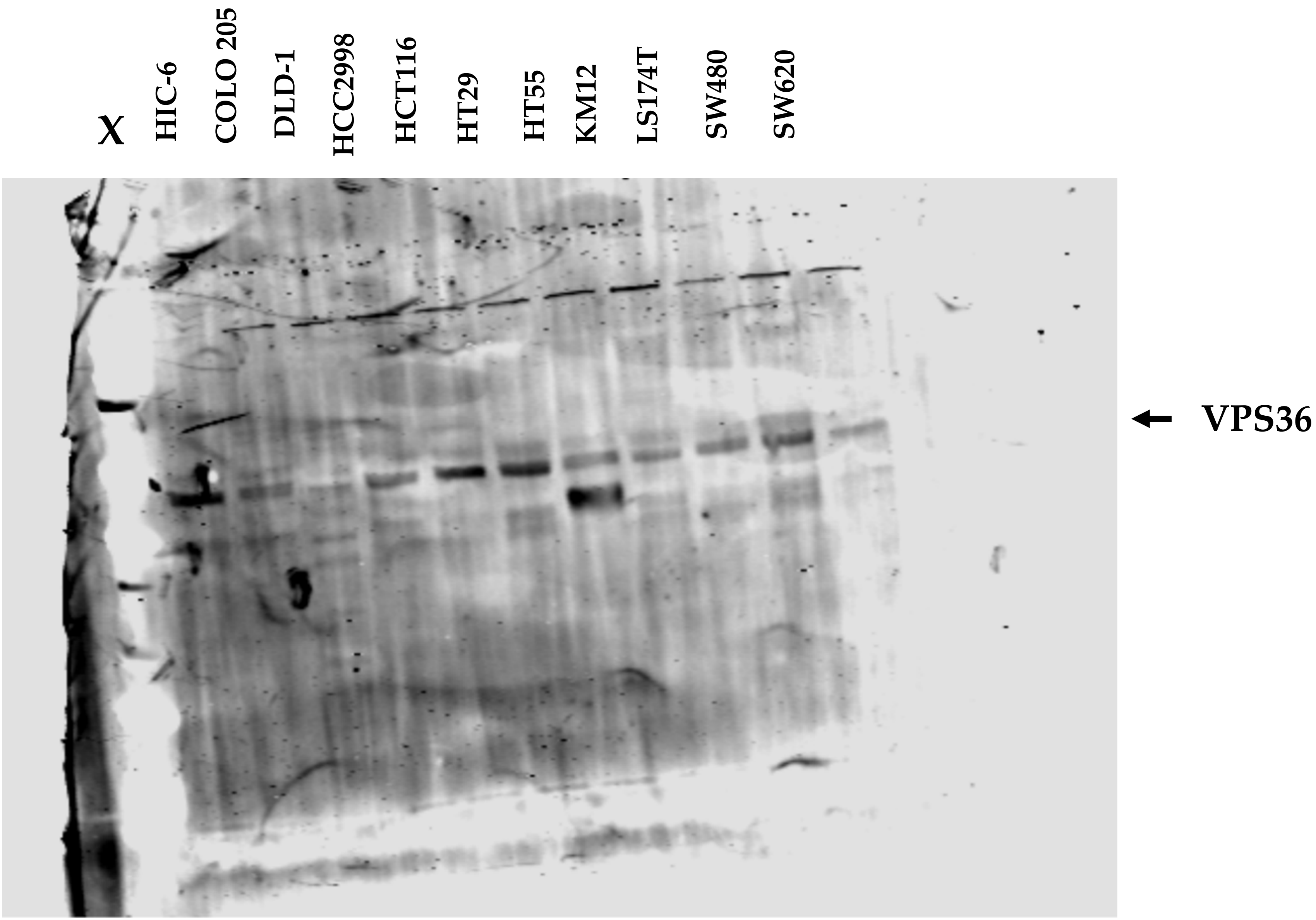

S1. Characterisation of ESCRT protein expression in a panel of human CRC cell lines using Western immunoblotting

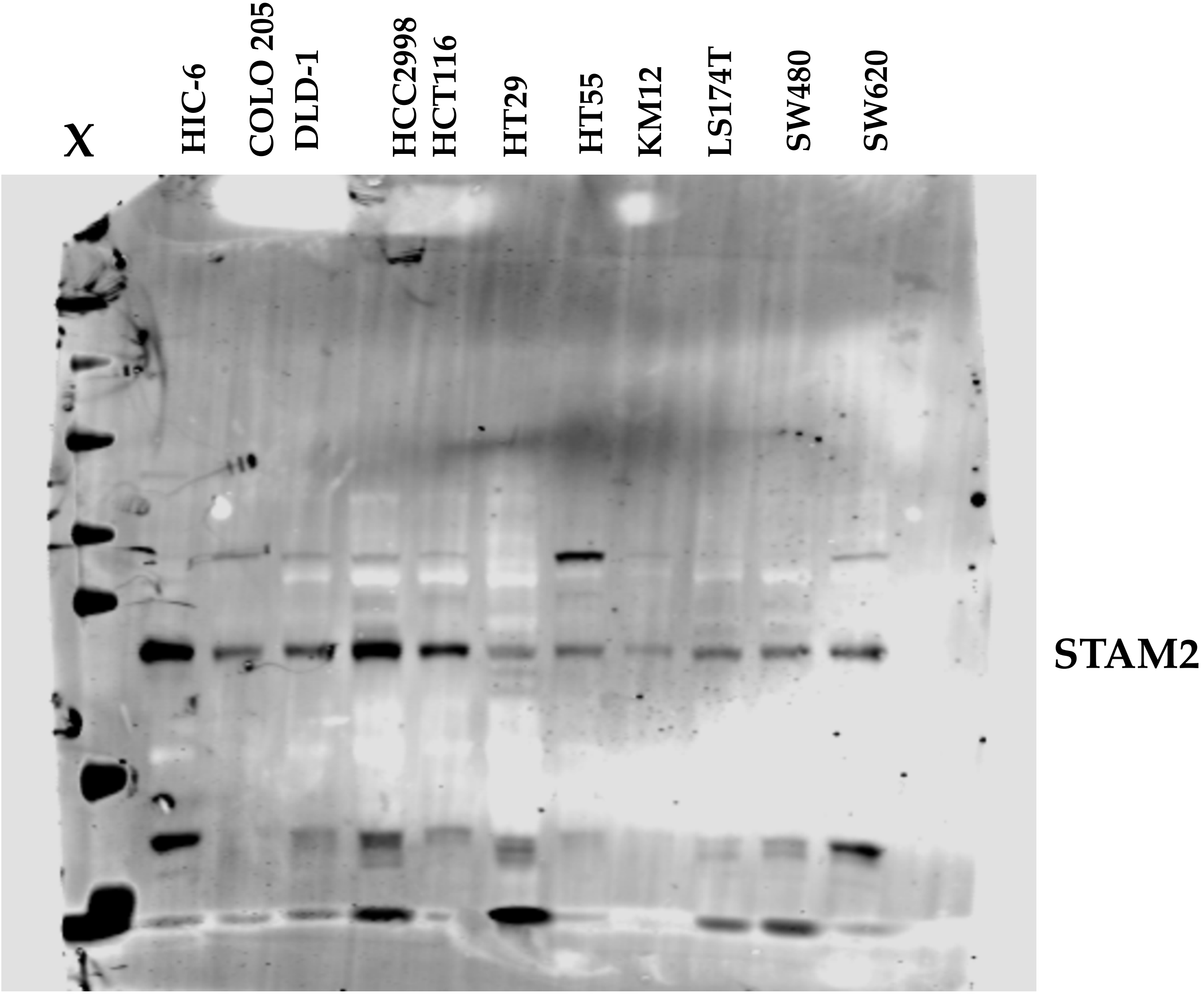

S1. Characterisation of ESCRT protein expression in a panel of human CRC cell lines using Western immunoblotting

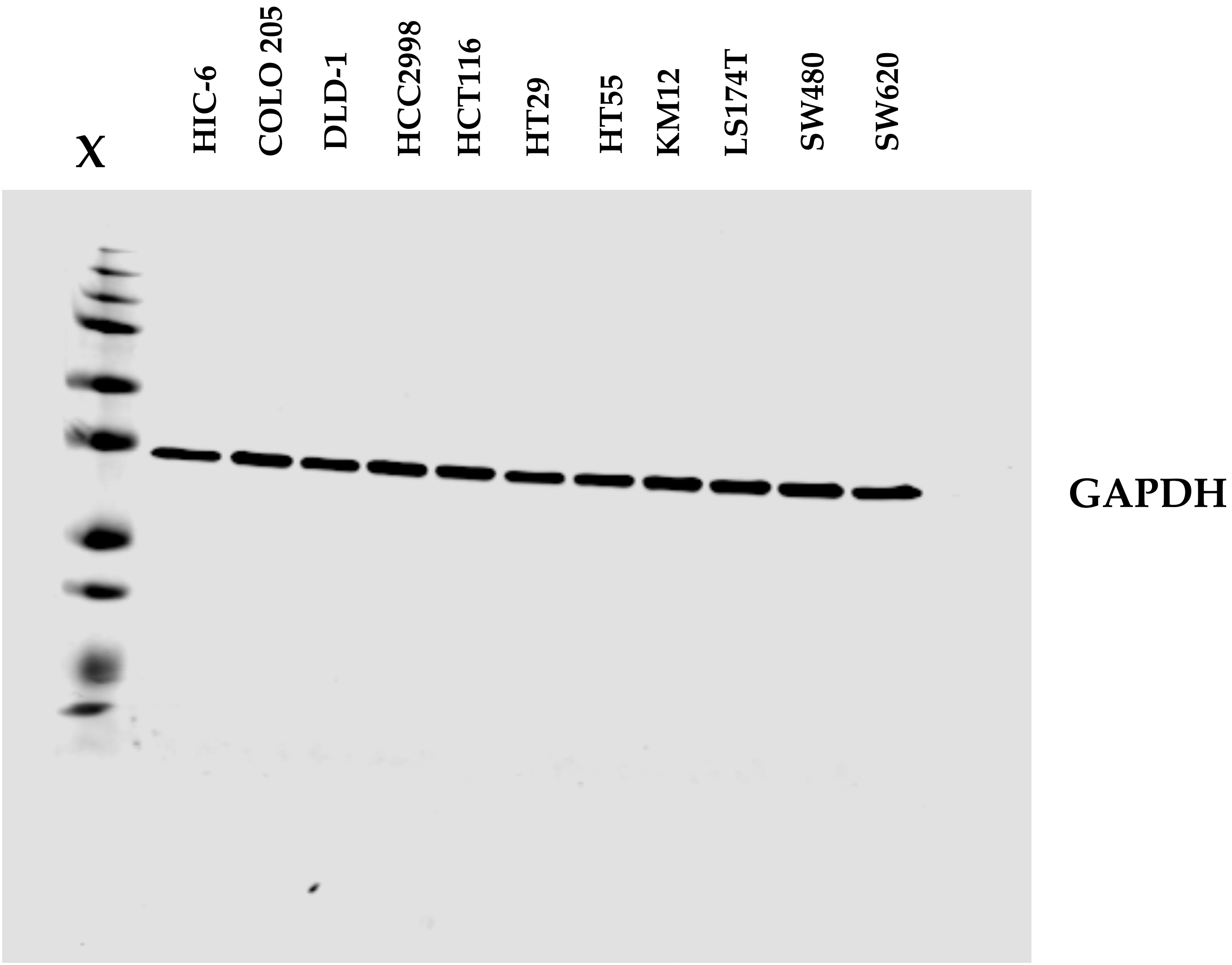

S1. Characterisation of ESCRT protein expression in a panel of human CRC cell lines using Western immunoblotting

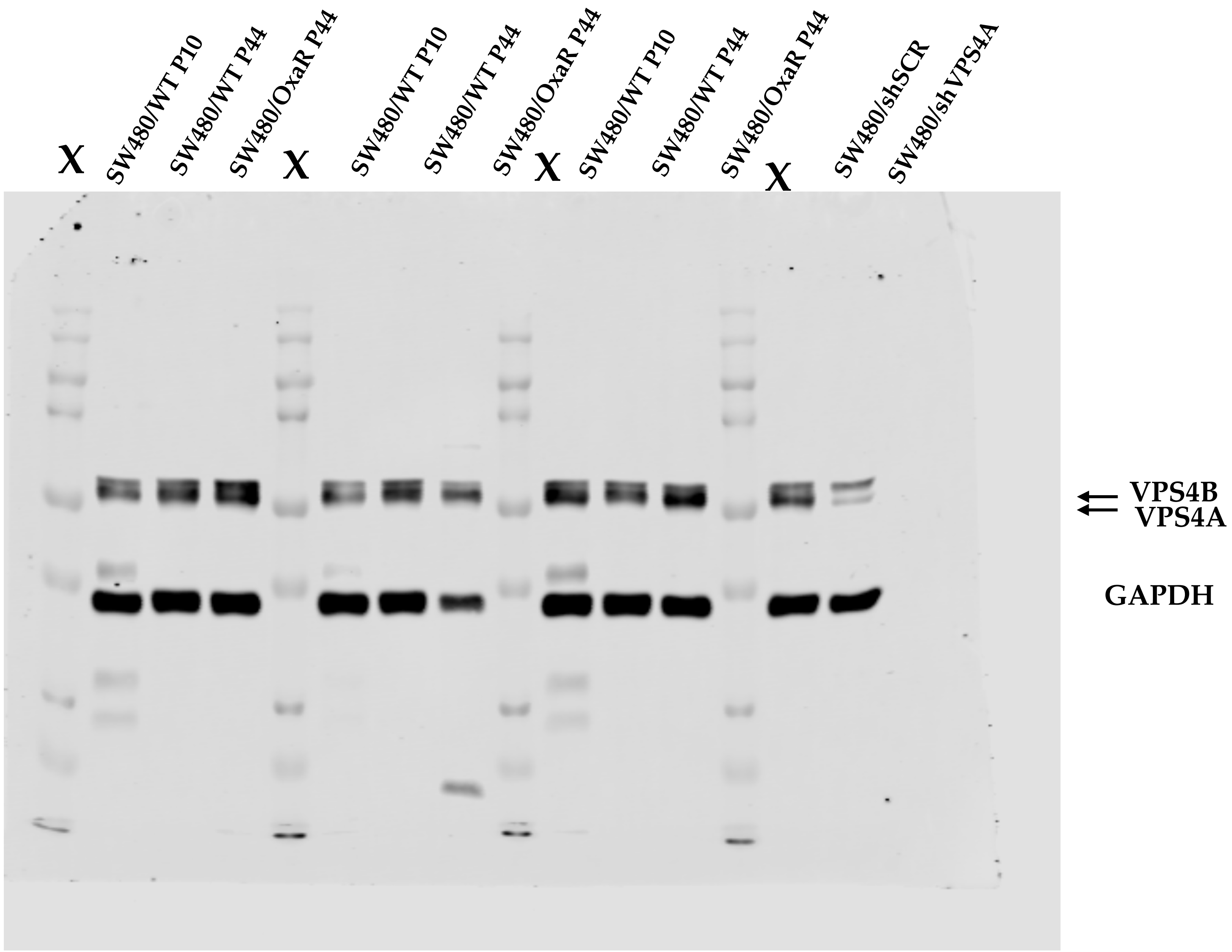

S3.A Generation of SW480 Oxaliplatin resistant cell lines

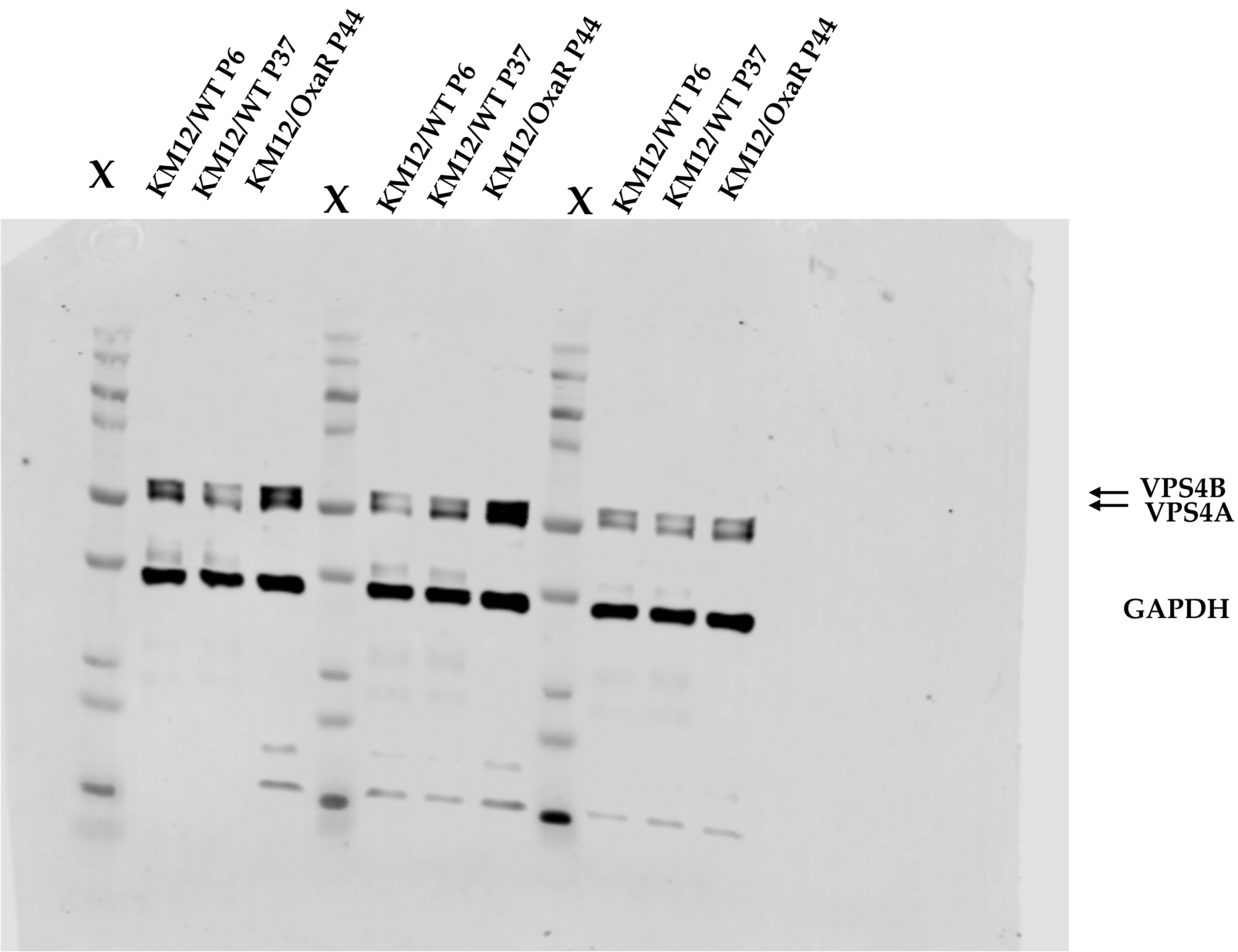

S3.B Generation of KM12 Oxaliplatin resistant cell lines

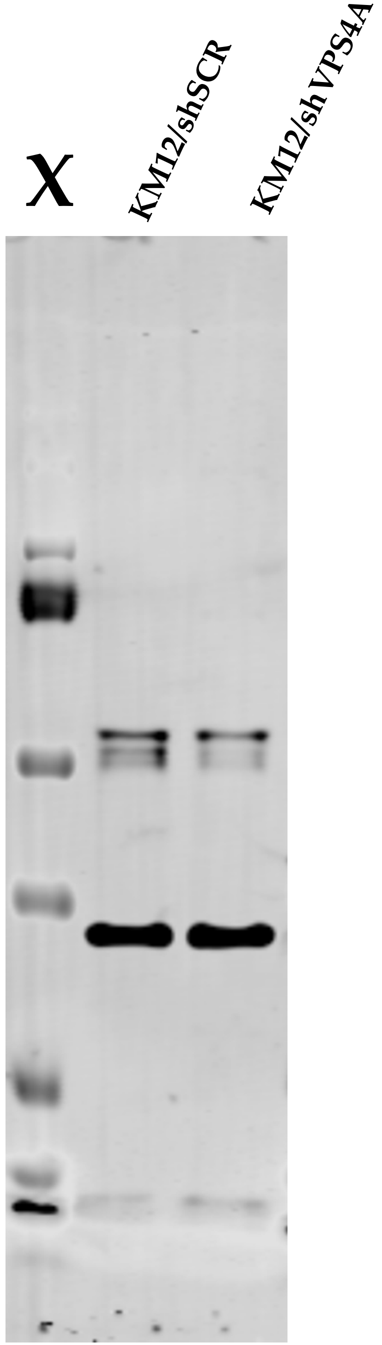

S3. Subline of KM12 (KM12/shVPS4A) with constitutively lower expression of VPS4A were established as confirmed using immunoblotting

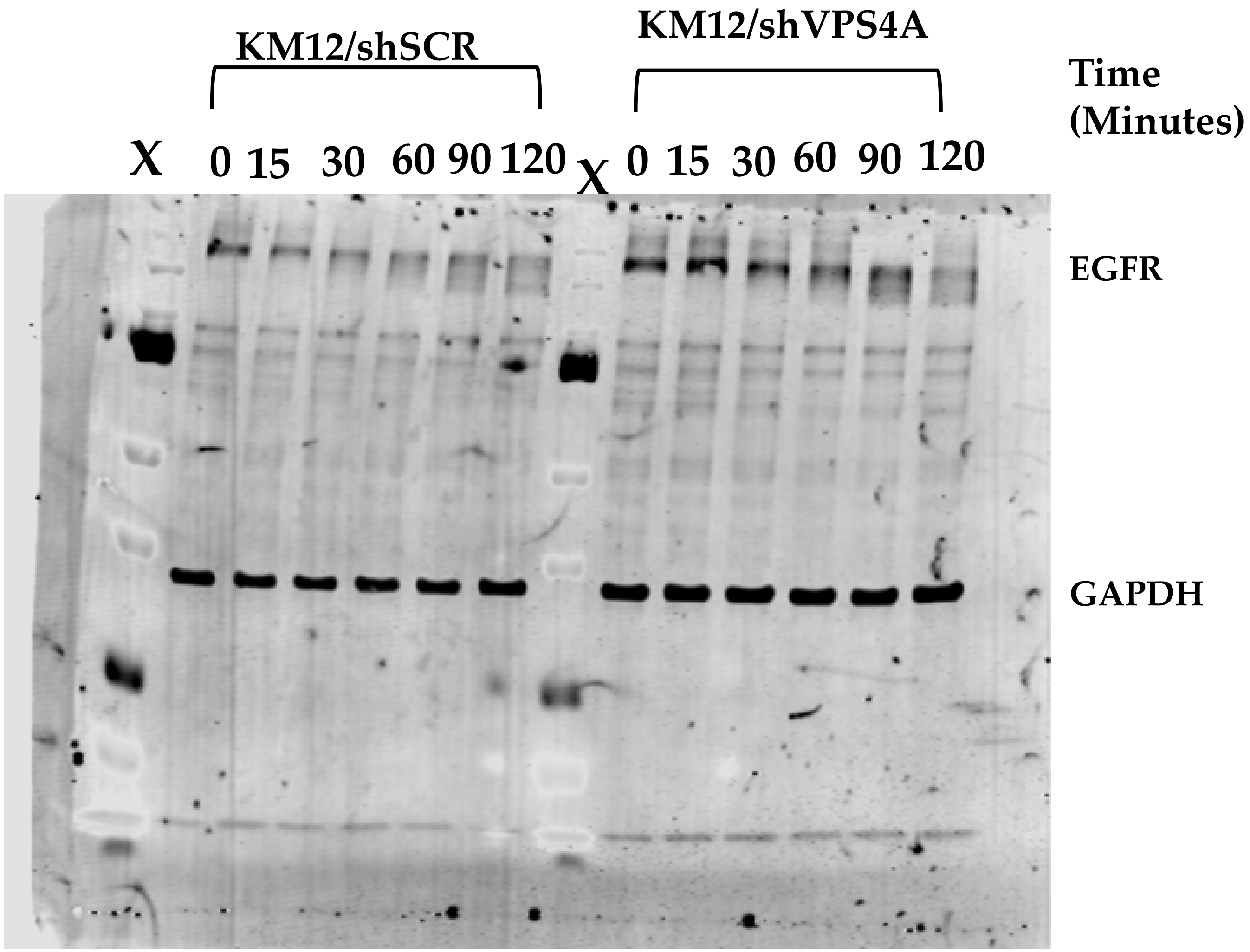

S3. Loss of ESCRT machinery function as a consequence of this knockdown was confirmed in KM12 sublines using the EGFR assay, using immunoblotting

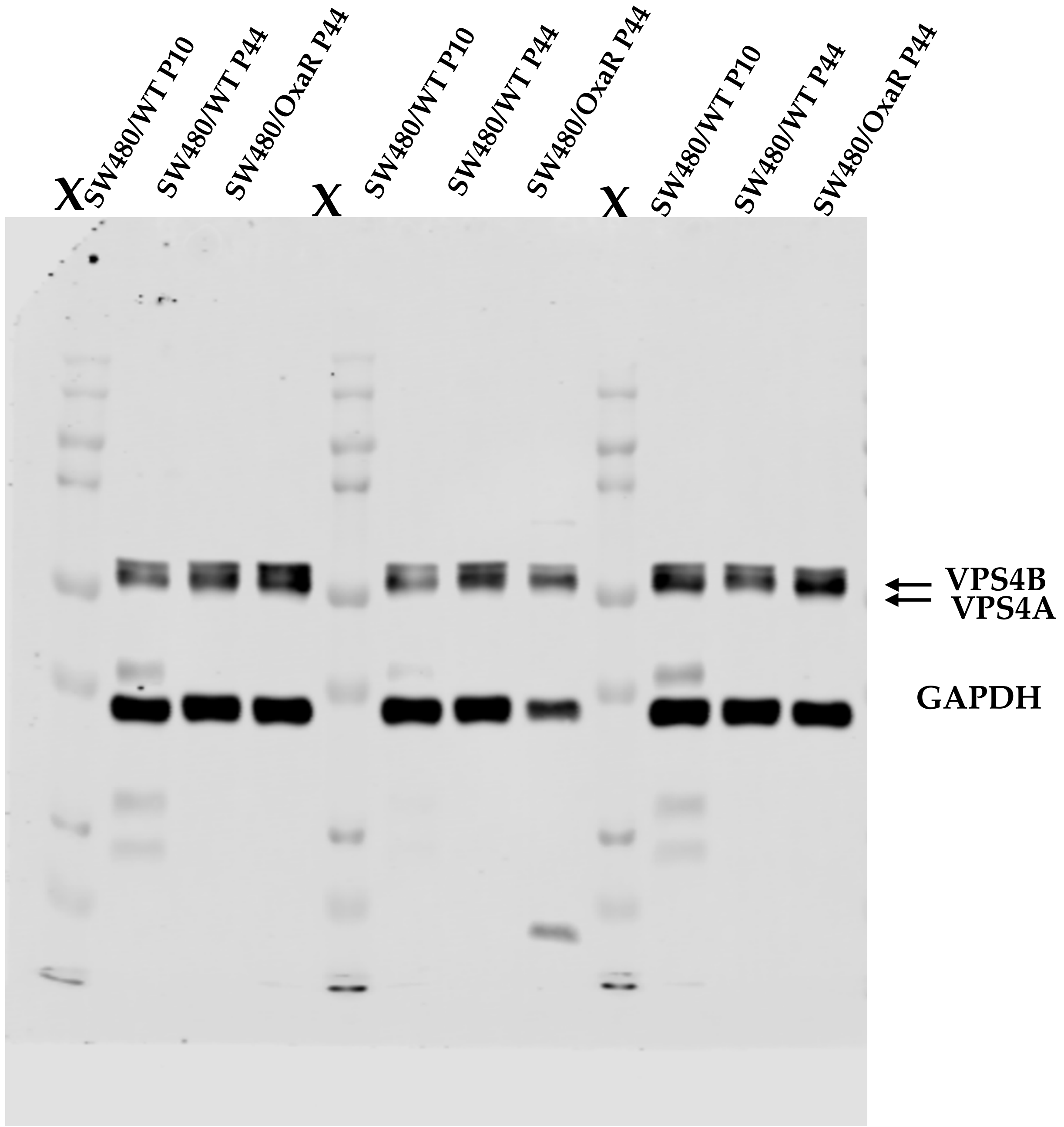

S4. raised expression of VPS4A in SW480the resistant cell lines respective to their parent wild type cell lines using immunoblotting

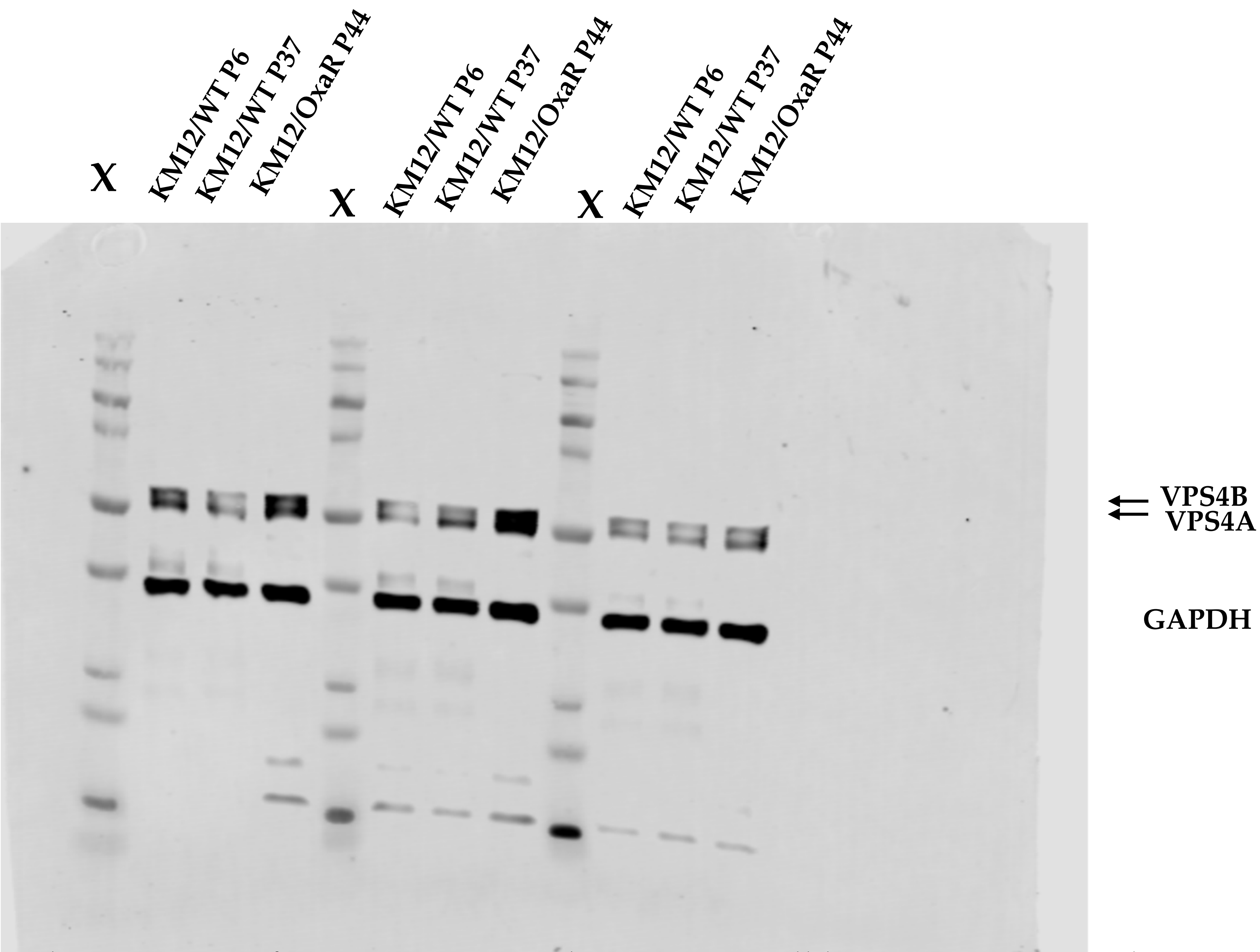

S4. raised expression of VPS4A in KM12 the resistant cell lines respective to their parent wild type cell lines using immunoblotting

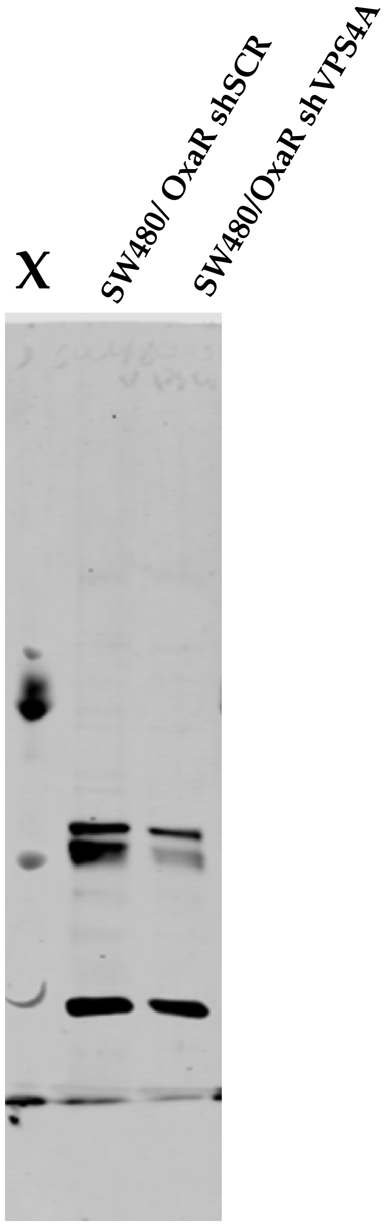

S5. A subline of SW480/OxaR with constitutively lower ex-pression of VPS4A was established as confirmed using immunoblotting

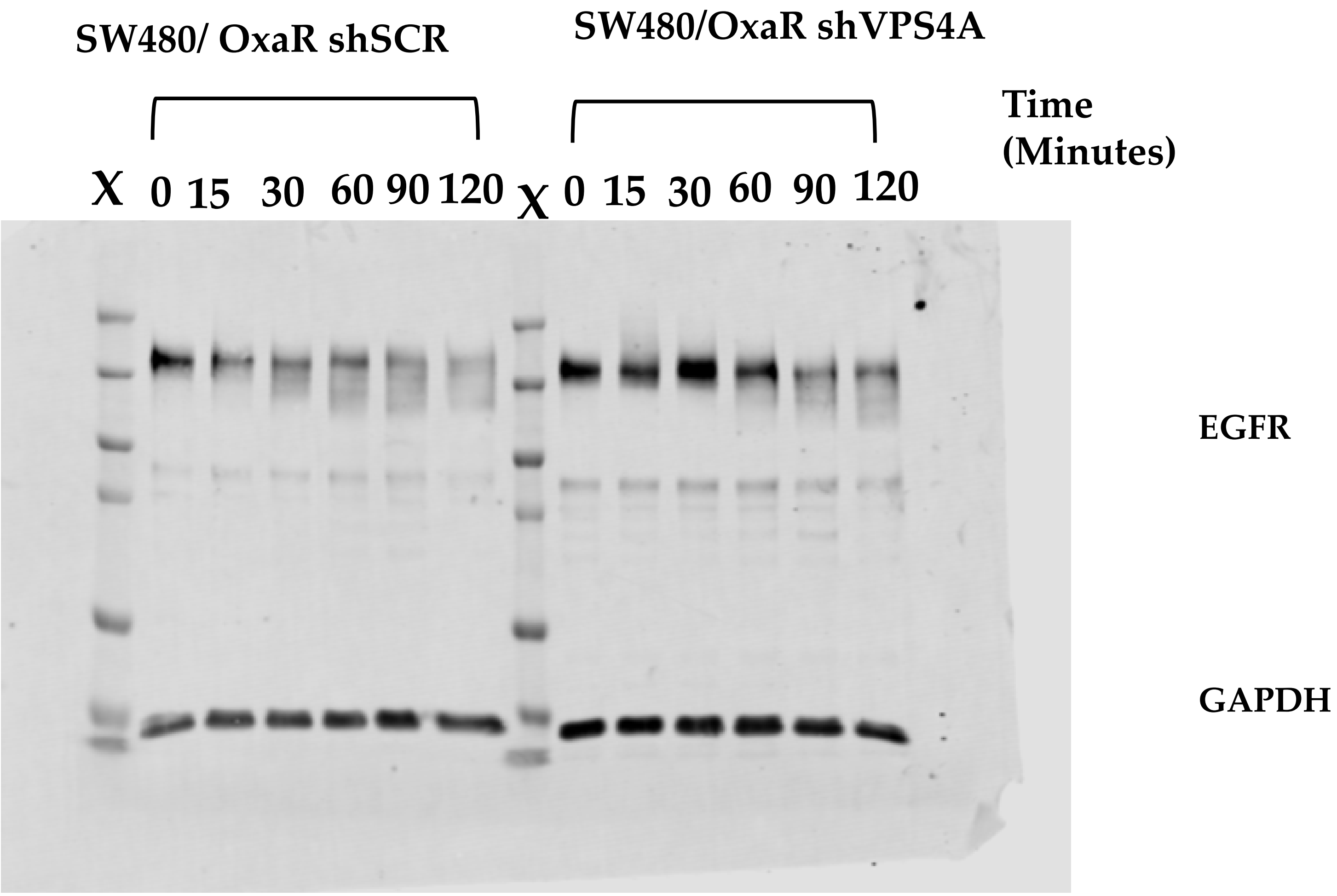

S5. loss of ESCRT machinery function as a consequence of VPS4A knockdown was confirmed using the EGFR assay in SW480/OxaR , using immunoblotting

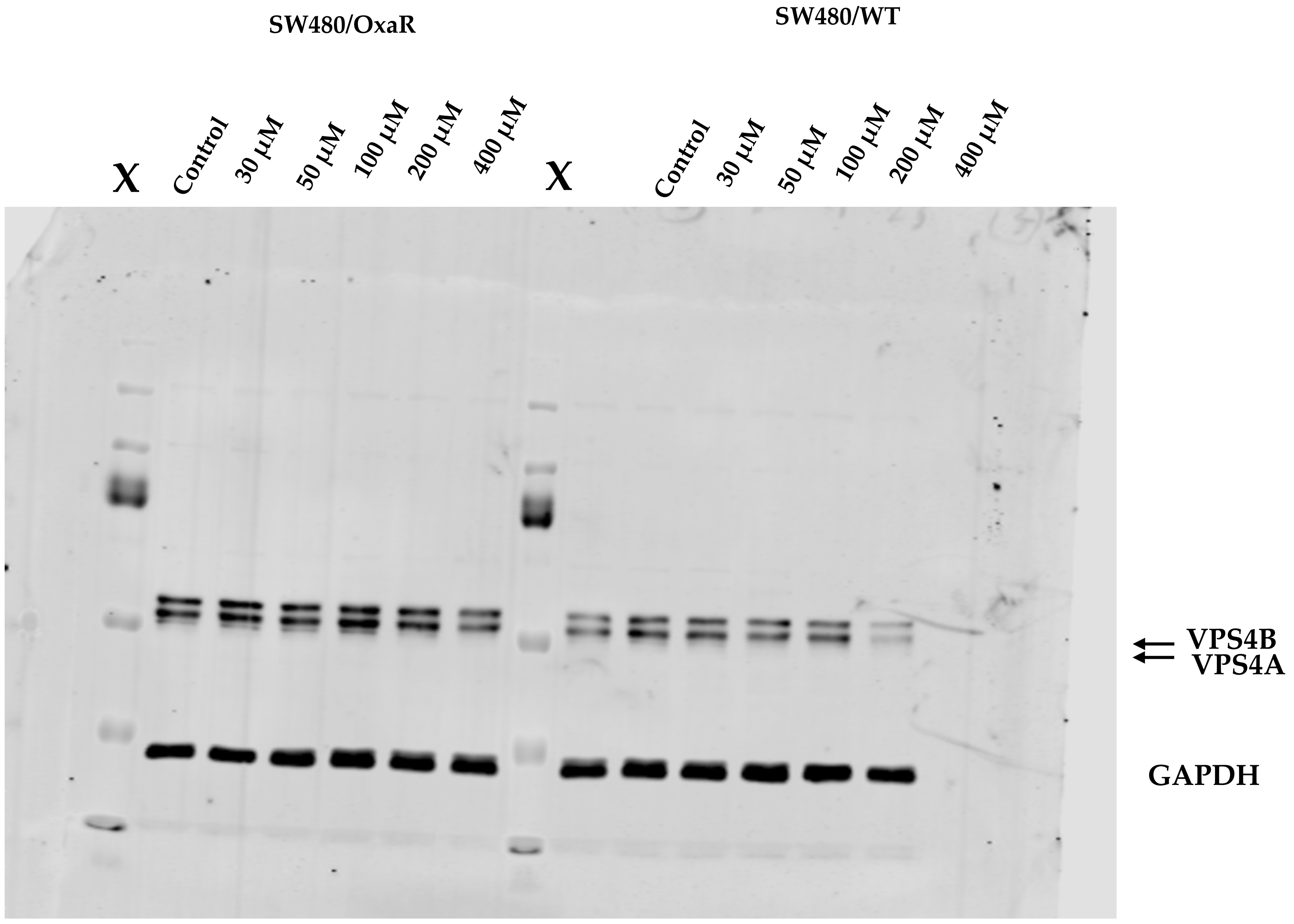

S6. Aloperine inhibits expression of VPS4 proteins.

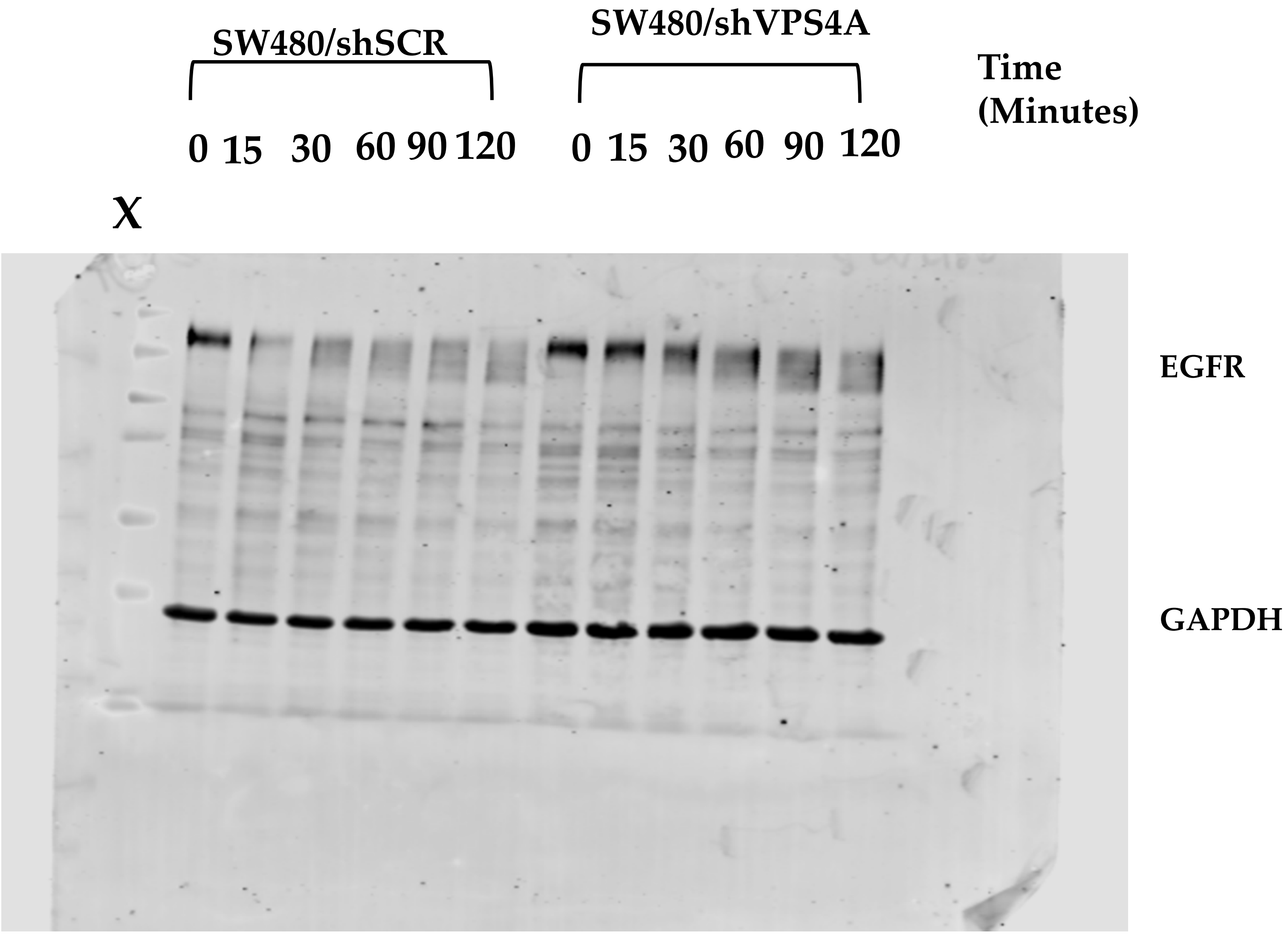

S6. EGFR degradation in SW480/shSCR vs SW480/shVPS4

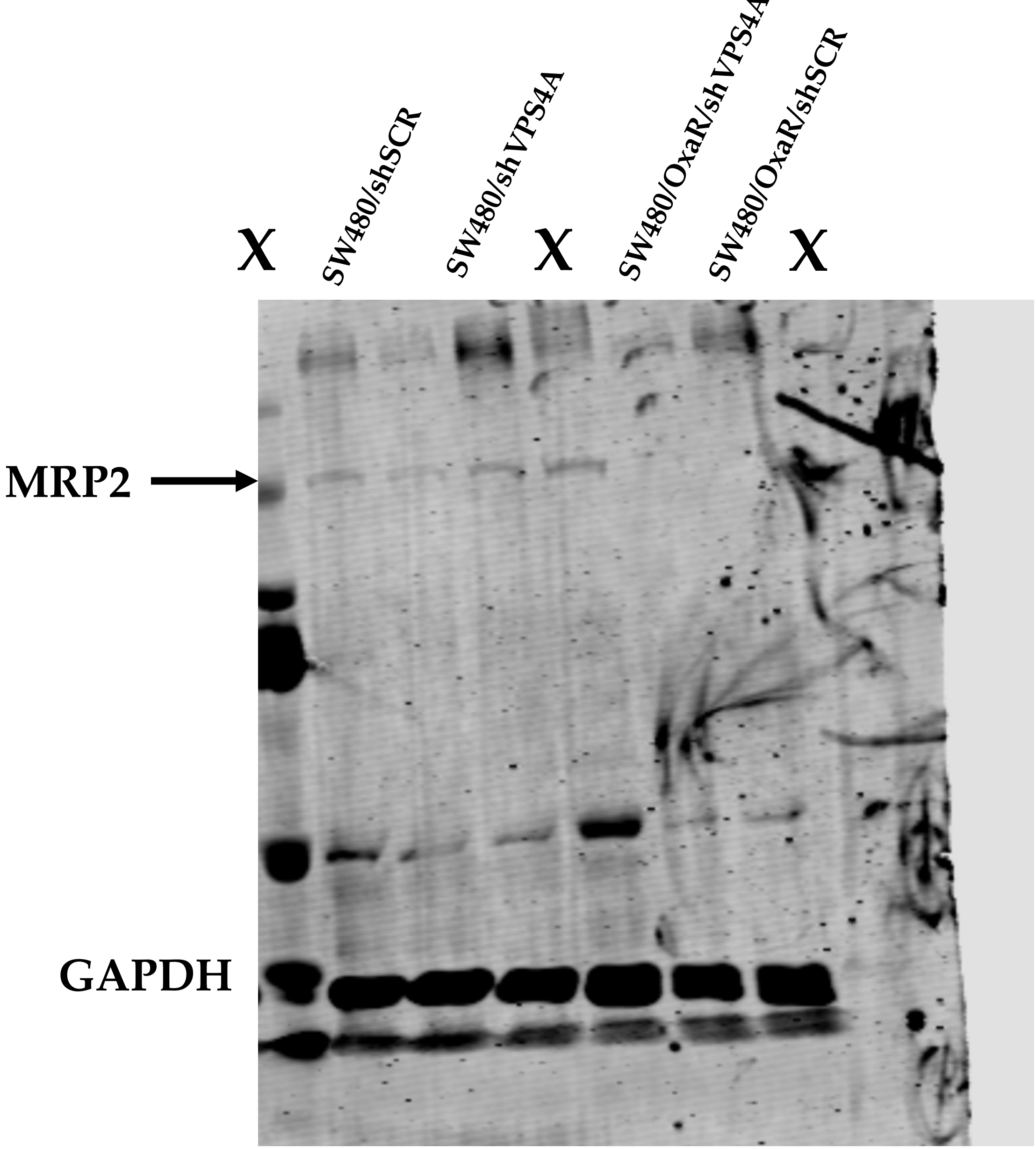

S7. MRP2 protein expression fold change in SW480/shVPS4A and SW480 OxaR shVPS4A compared to the respective scrambled controls using immunoblotting

Supplementary Figure S2

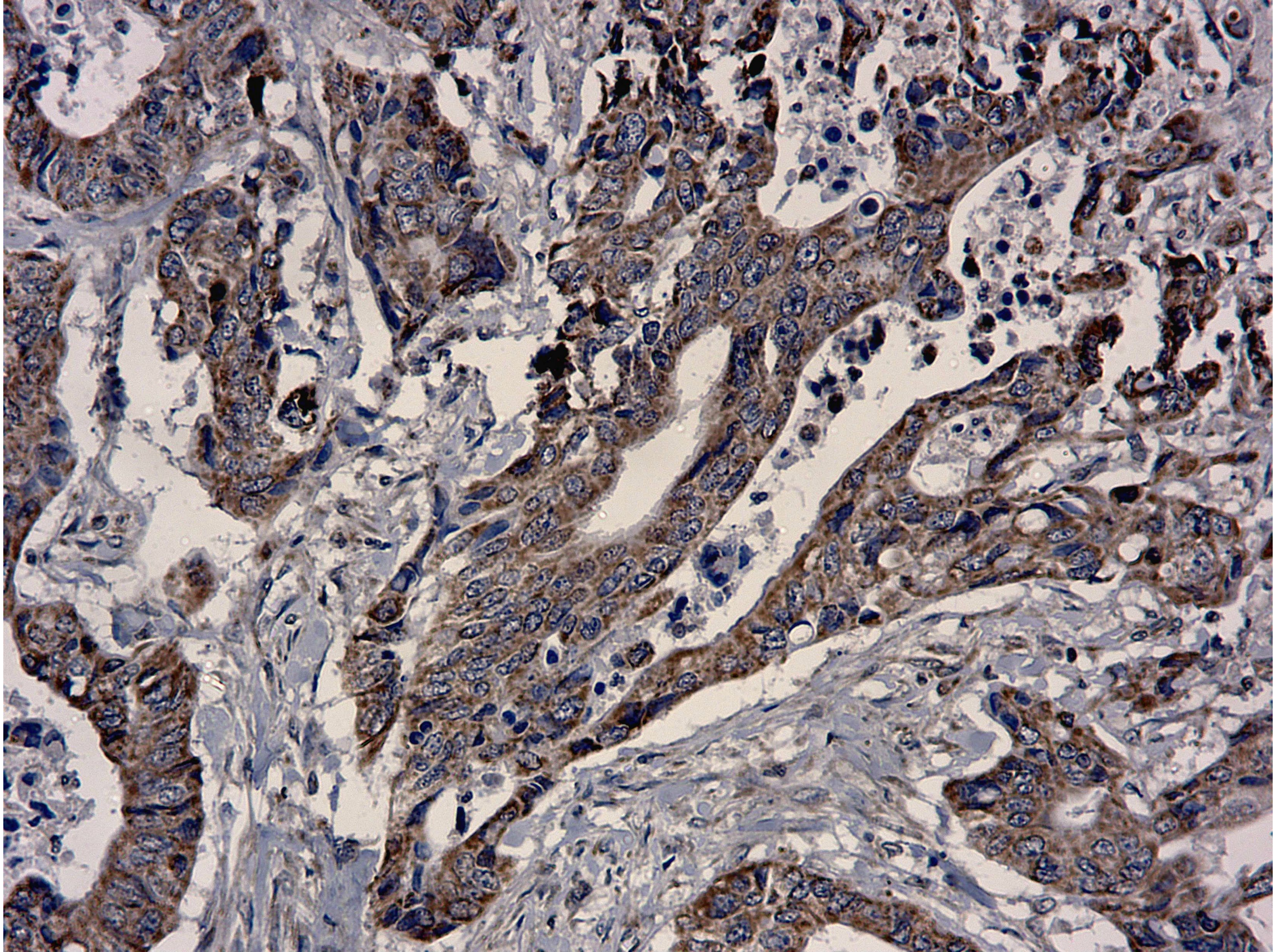

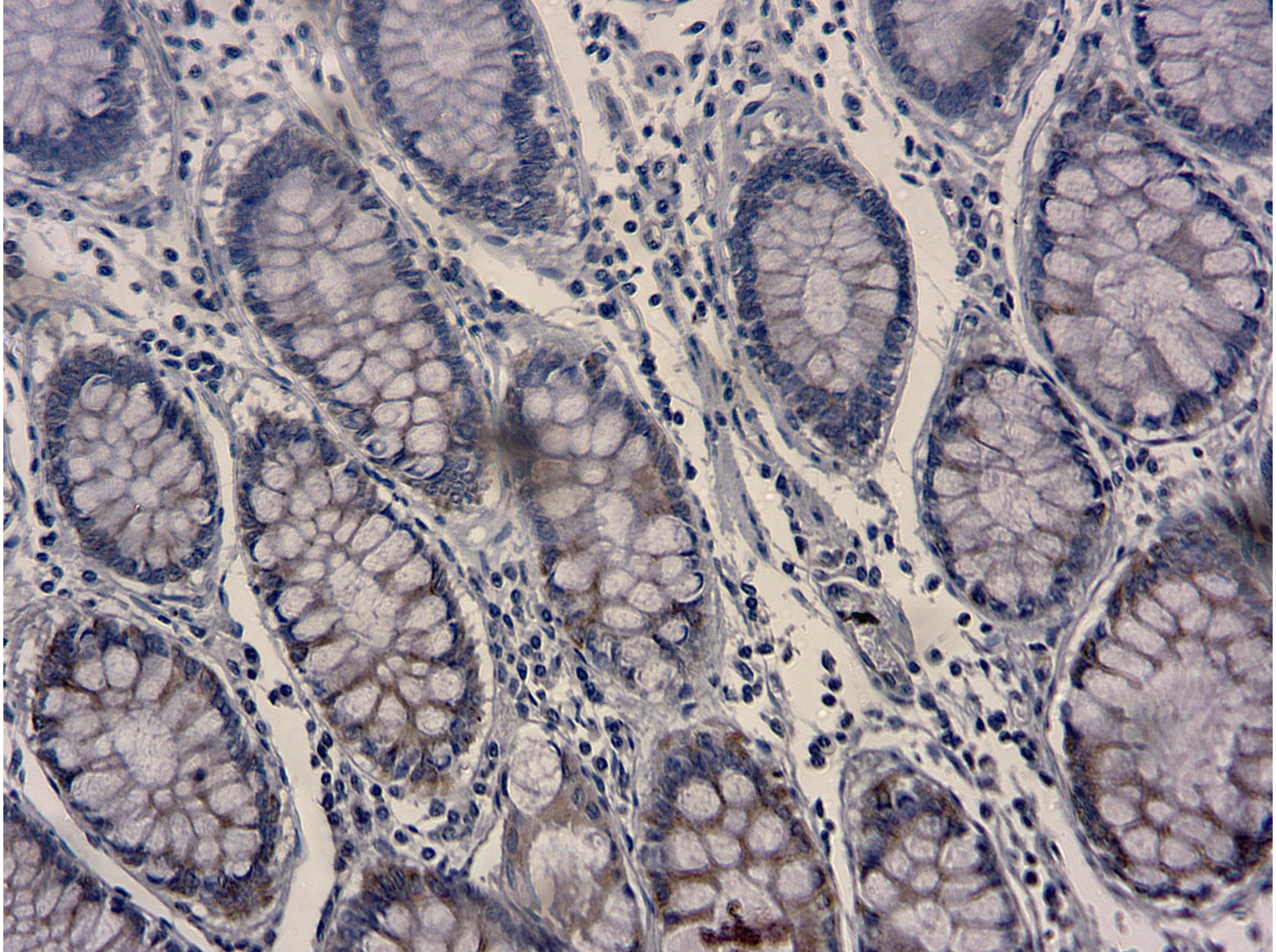

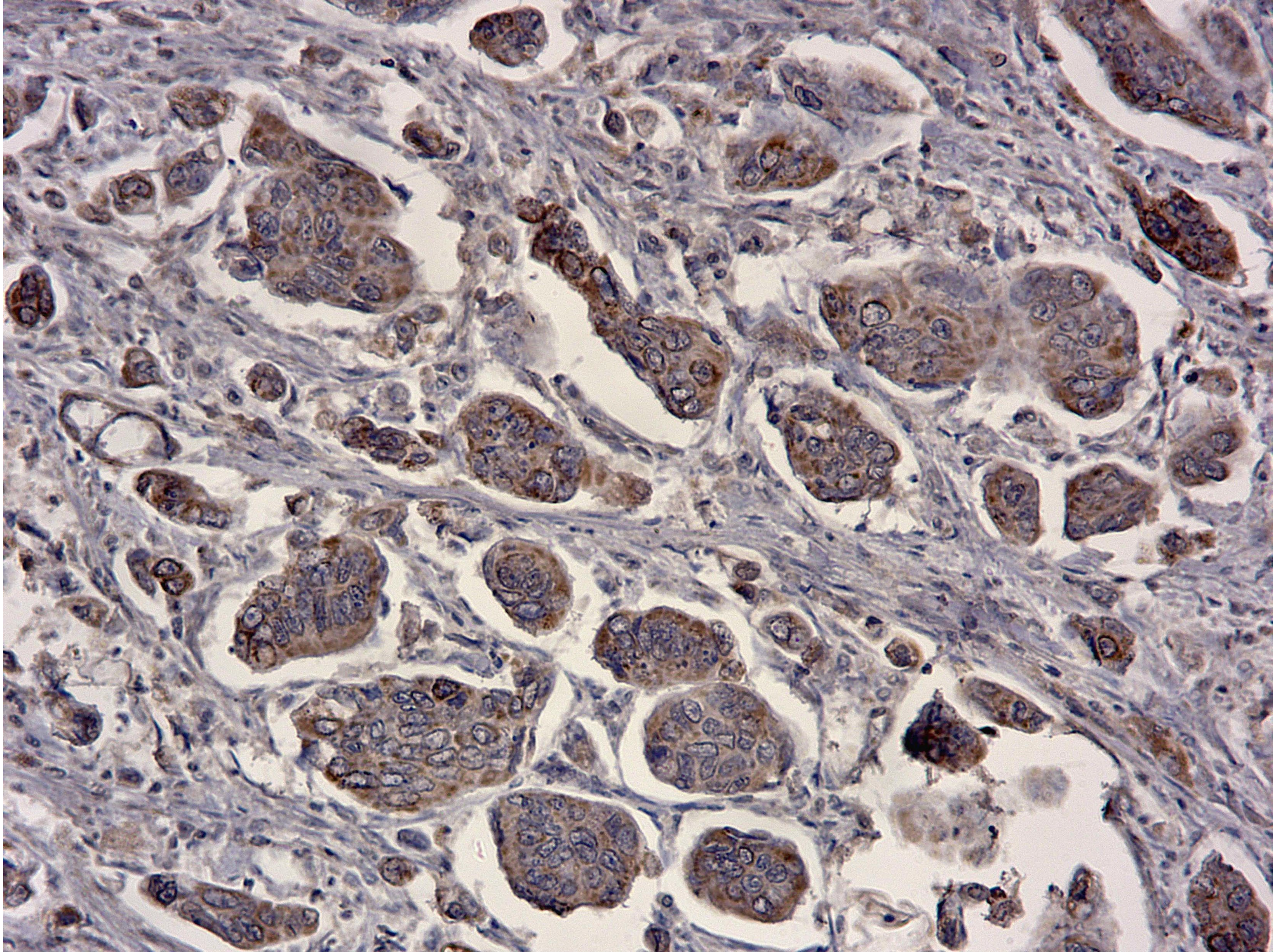

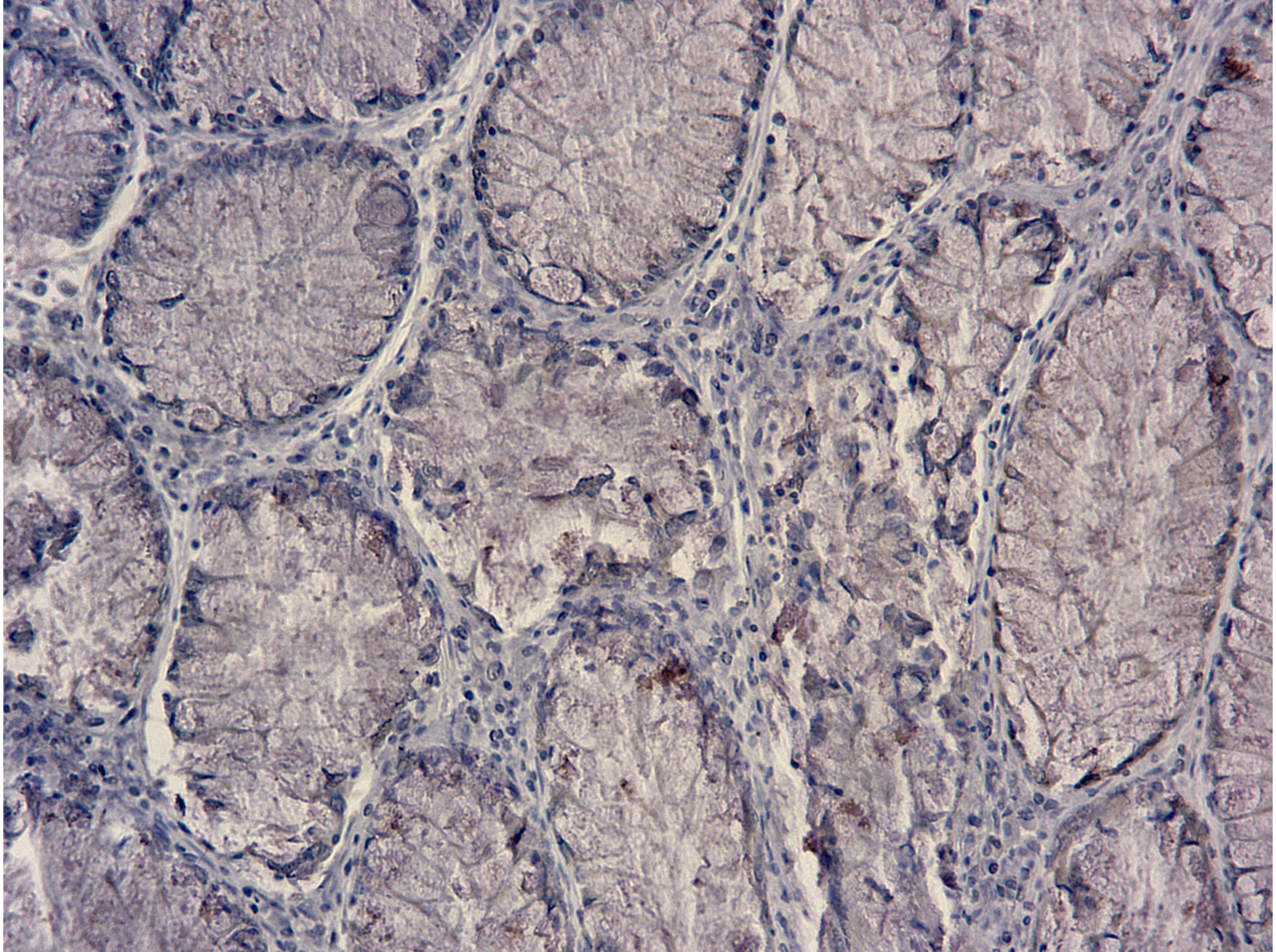

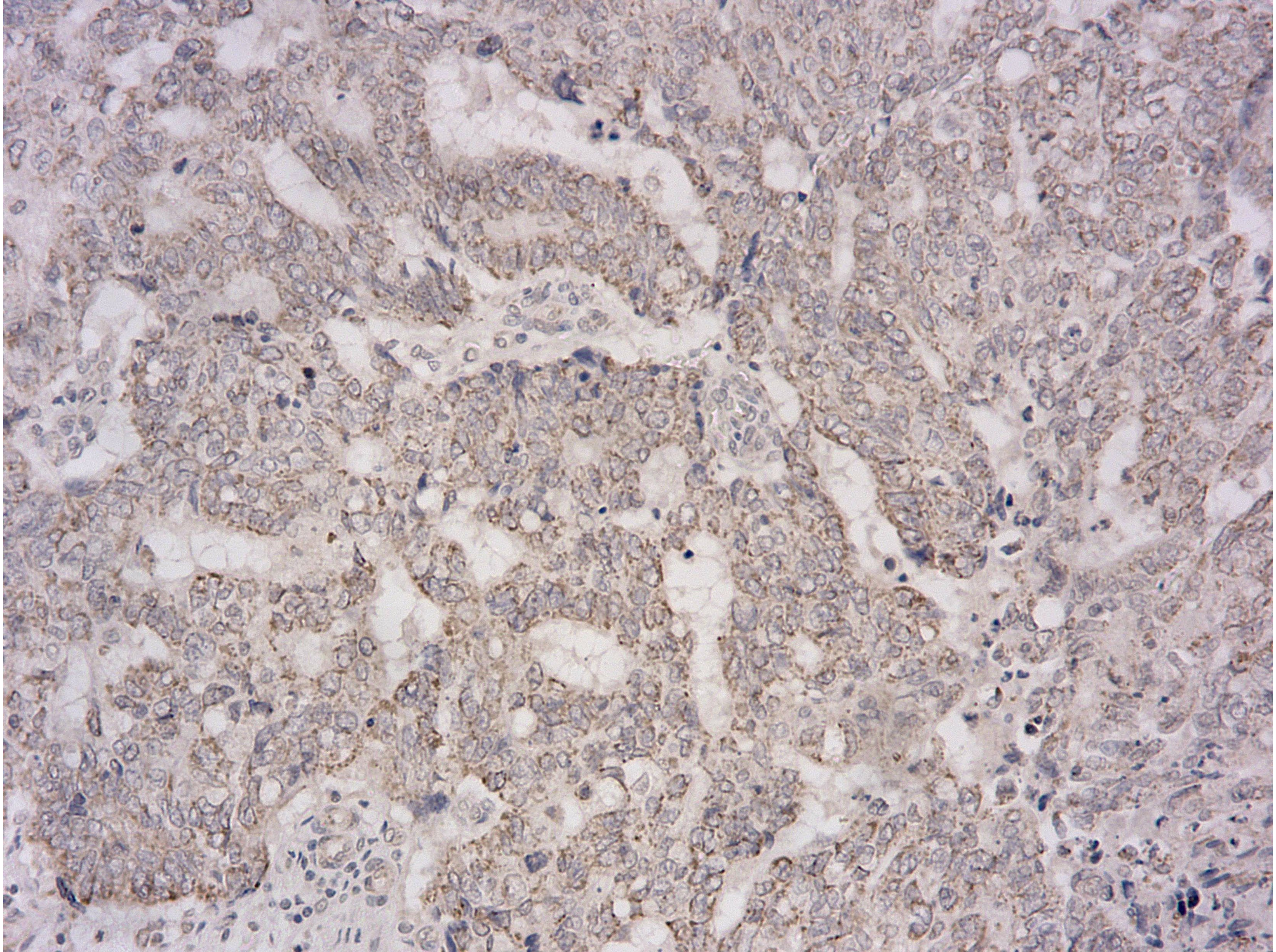

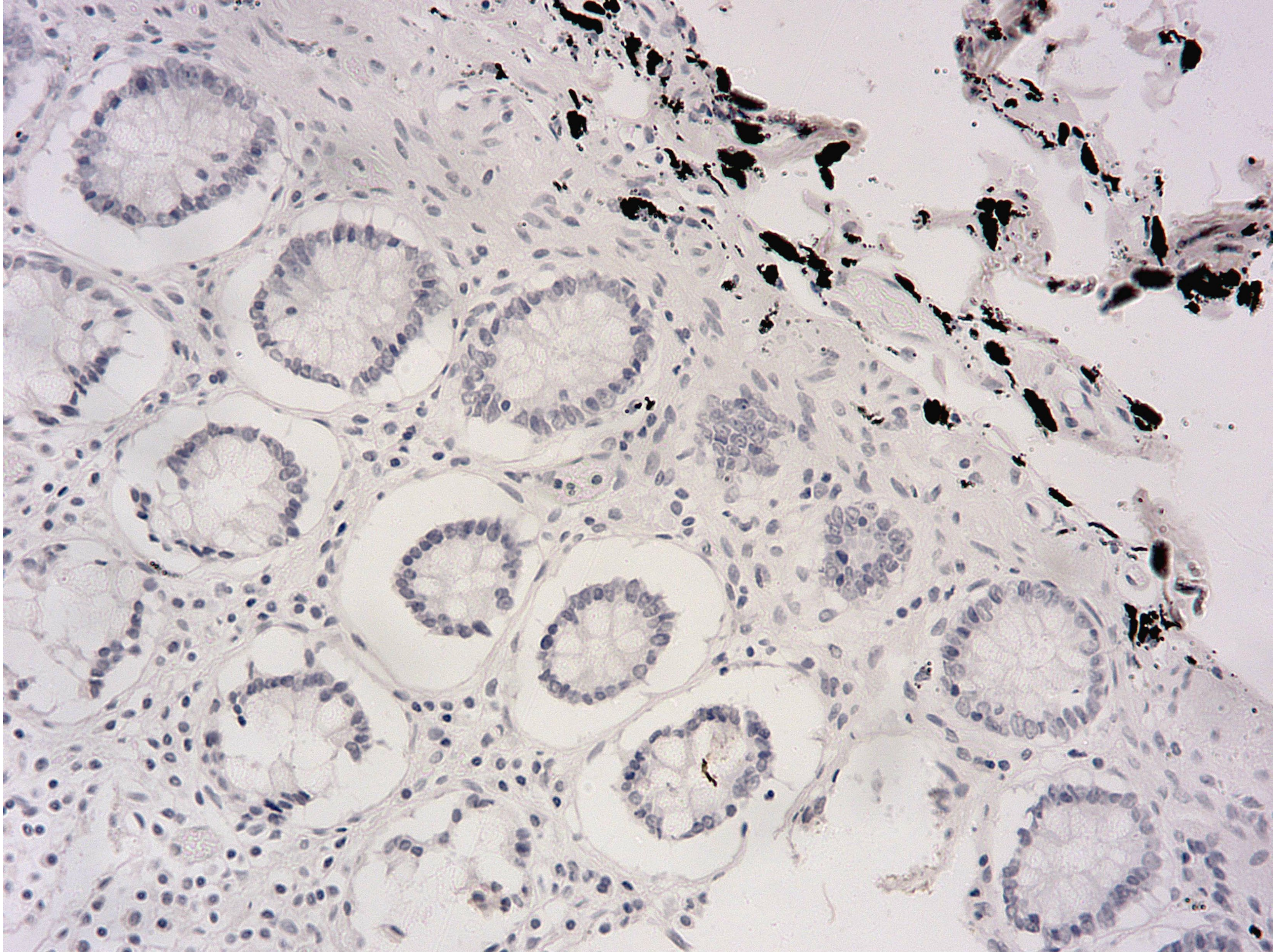

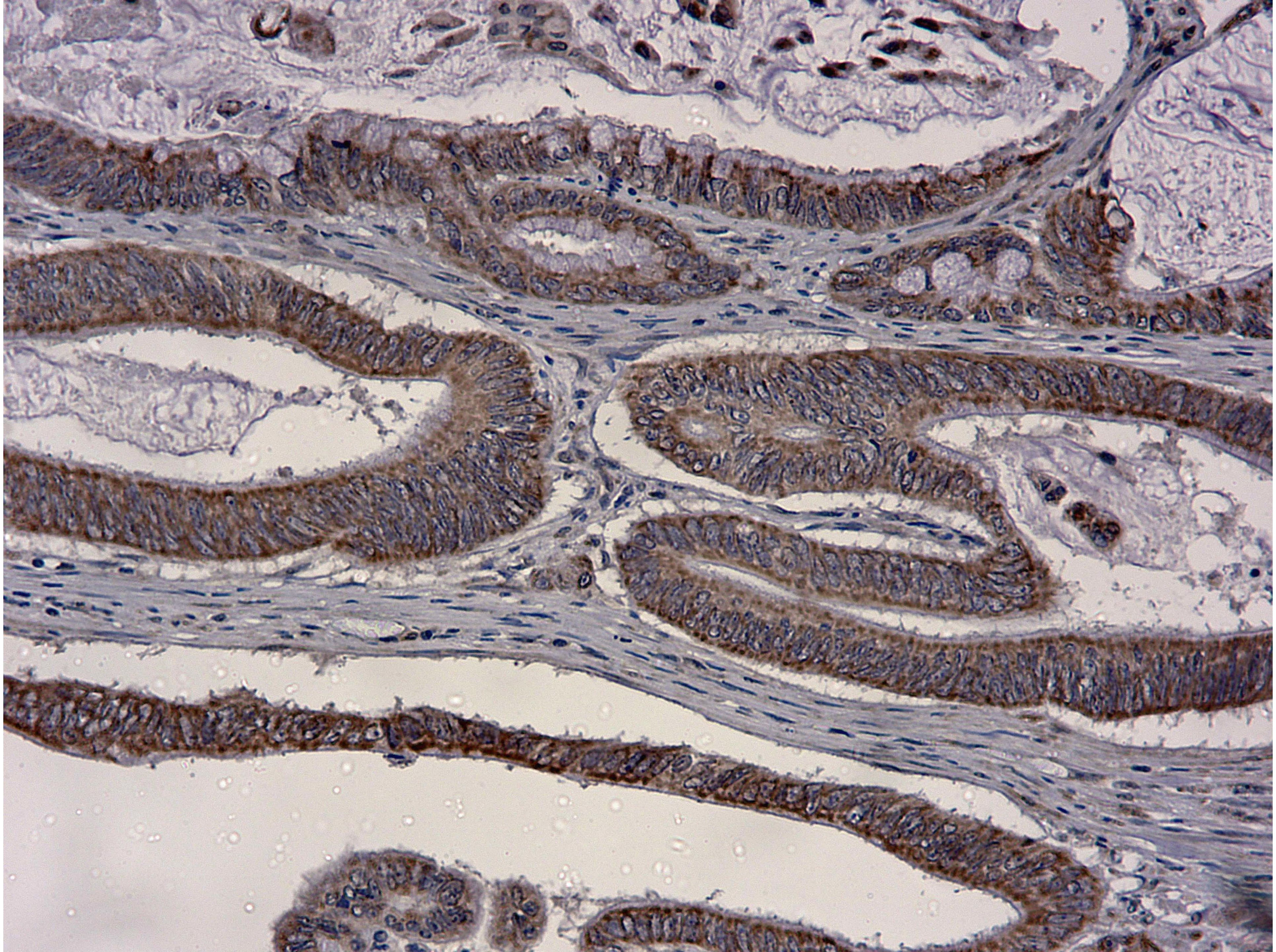

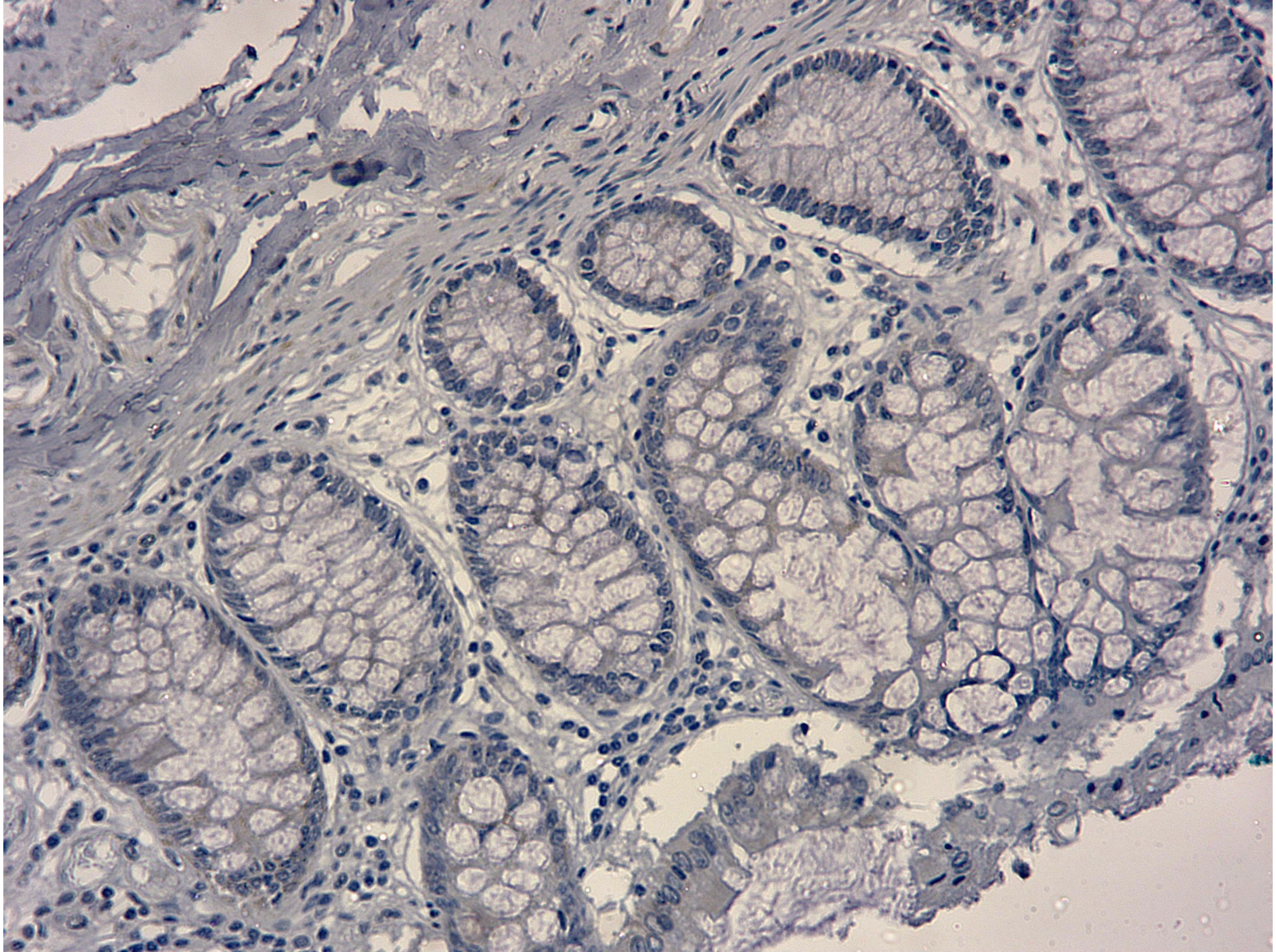

Supplementary Figure S3

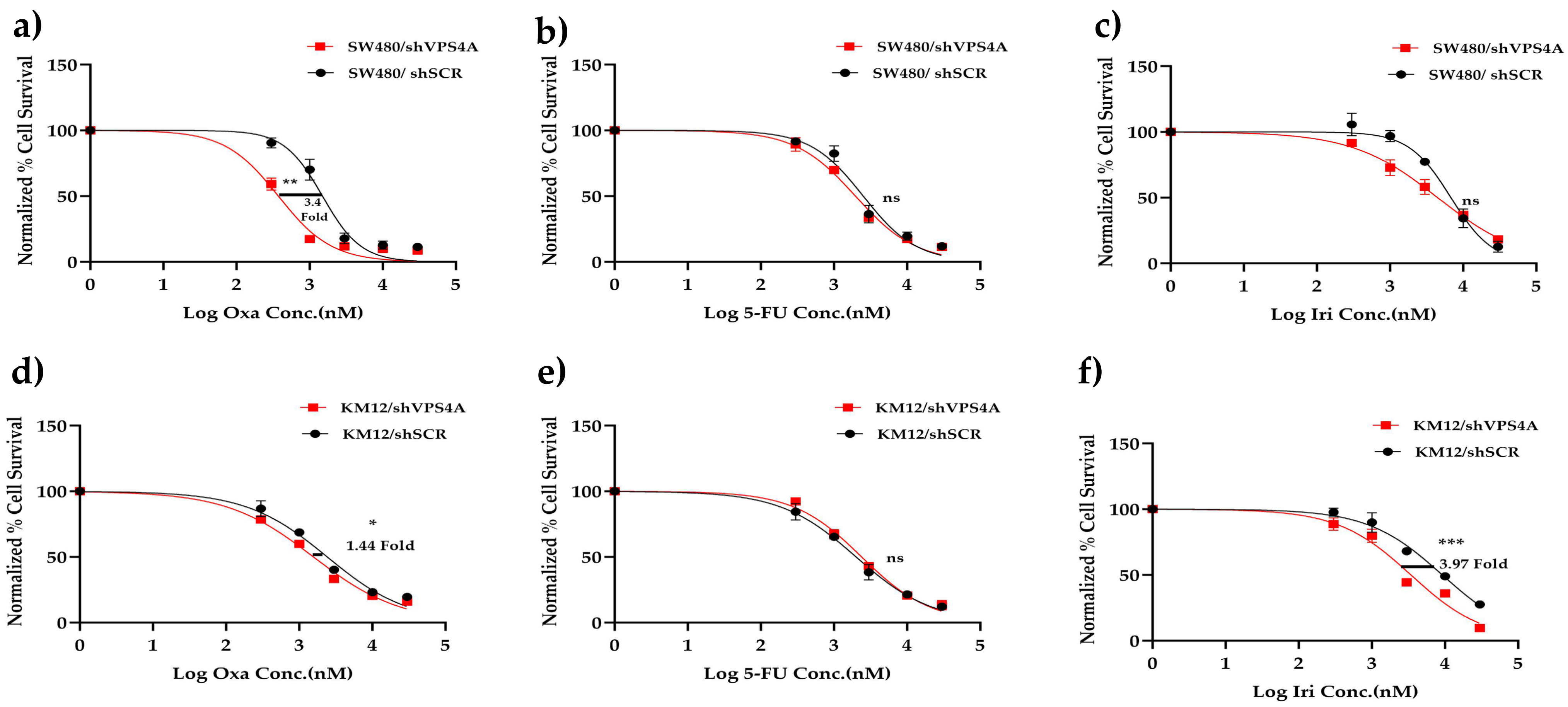

Supplementary Figure S3 . MTT assays for Section 3.4.

Supplementary Figure S4

gene_id	TA481_R1	TA481_R2	TA481_R3	S480_R1	S480_R2	S480_R3	KD	SCR	log2FoldChange	pvalue	padj	gene_name	gene_chr	gene_start	gene_end	gene_st	gene_lengt	gene_biotyp	gene_description
ENSG0000	111.852	155.5656	121.5744	7231.098	8294.978	8266.297	129.664	7930.791	-5.933544656	0	0	NR4A2	2	156324432	156342348	-	3967	protein_cod	nuclear receptor subfamily 4 group A member 2 [Source:HGNC Symbol;Acc:HGNC:7981]
ENSG0000	3165.719	2814.699	2752.31	16093.1	16448.96	15039.19	2910.909	15860.42	-2.445592137	1.09E-289	1.31E-285	EGR1	5	138465490	138469315	+	3138	protein_cod	early growth response 1 [Source:HGNC Symbol;Acc:HGNC:3238]
ENSG0000	4347.861	4280.127	4202.197	18555.83	17790.68	17046.73	4276.729	17797.75	-2.056997105	1.06E-288	8.45E-285	CYP24A1	20	54153449	54173973	-	3972	protein_cod	cytochrome P450 family 24 subfamily A member 1 [Source:HGNC Symbol;Acc:HGNC:2602]
ENSG0000	6252.424	5966.458	5700.489	1231.851	1237.106	1141.439	5973.124	1203.465	2.311651201	2.73E-248	1.64E-244	CCNB1	5	69167010	69178245	+	2558	protein_cod	cyclin B1 [Source:HGNC Symbol;Acc:HGNC:1579]
ENSG0000	5532.056	5632.51	6030.316	1012.084	973.3114	845.6092	5731.627	943.6681	2.603120237	4.92E-248	2.36E-244	OAS3	12	112938352	112973249	+	8251	protein_cod	2'-5'-oligoadenylate synthetase 3 [Source:HGNC Symbol;Acc:HGNC:8088]
ENSG0000	6226.77	6086.762	6012.305	1472.823	1398.112	1393.607	6108.612	1421.514	2.10372053	3.85E-247	1.54E-243	TPX2	20	31739271	31801805	+	3605	protein_cod	TPX2, microtubule nucleation factor [Source:HGNC Symbol;Acc:HGNC:1249]
ENSG0000	4872.232	4455.397	4660.353	1103.653	1074.281	1013.127	4662.661	1063.687	2.132432272	1.29E-199	4.44E-196	CDC20	1	43358955	43363203	+	2038	protein_cod	cell division cycle 20 [Source:HGNC Symbol;Acc:HGNC:1723]
ENSG0000	2773.724	2829.219	2674.637	10024.45	10338.93	9401.499	2759.193	9921.627	-1.846135115	1.61E-196	4.82E-193	TRIB1	8	125430321	125438405	+	4138	protein_cod	tribbles pseudokinase 1 [Source:HGNC Symbol;Acc:HGNC:16891]
ENSG0000	7894.288	8131.93	7419.417	1843.92	1902.051	1638.646	7815.212	1794.872	2.122702541	6.56E-196	1.75E-192	TOP2A	17	40388516	40417950	-	6437	protein_cod	DNA topoisomerase II alpha [Source:HGNC Symbol;Acc:HGNC:11989]
ENSG0000	3928.16	4127.673	3723.779	867.5004	868.7032	906.2008	3926.537	880.8014	2.156355902	1.19E-191	2.85E-188	ASPM	1	197084128	197146694	-	10887	protein_cod	abnormal spindle microtubule assembly [Source:HGNC Symbol;Acc:HGNC:19048]
ENSG0000	7134.926	7096.901	7667.068	23005.15	22390.71	22185.43	7299.632	22527.1	-1.62588963	2.82E-191	6.15E-188	MAT2A	2	85539165	85545280	+	4487	protein_cod	methionine adenosyltransferase 2A [Source:HGNC Symbol;Acc:HGNC:6904]
ENSG0000	2846.582	3170.426	3228.476	577.3697	499.3906	539.087	3081.828	538.6158	2.5168596	1.11E-187	2.22E-184	IFI6	1	27666061	27672218	-	865	protein_cod	interferon alpha inducible protein 6 [Source:HGNC Symbol;Acc:HGNC:4054]
ENSG0000	269.8814	235.4225	225.1378	1810.184	1799.262	1828.44	243.4806	1812.629	-2.894538554	6.22E-184	1.15E-180	DUSP2	2	96143166	96145440	-	1932	protein_cod	dual specificity phosphatase 2 [Source:HGNC Symbol;Acc:HGNC:3068]
ENSG0000	3328.879	3573.859	3226.225	694.0003	582.1676	614.8265	3376.321	630.3314	2.42218176	2.55E-182	4.38E-179	KIF20A	5	138178719	138187715	+	3958	protein_cod	kinesin family member 20A [Source:HGNC Symbol;Acc:HGNC:9787]
ENSG0000	12455.59	11794.98	11464.02	3562.535	3389.307	3188.187	11904.86	3380.01	1.816697993	2.98E-182	4.78E-179	KPNA2	17	68035519	68046842	+	3140	protein_cod	karyopherin subunit alpha 2 [Source:HGNC Symbol;Acc:HGNC:6395]
ENSG0000	913.2869	783.0133	738.452	5369.827	5620.646	7042.882	811.5841	6011.118	-2.888233497	5.20E-179	7.81E-176	FOSB	19	45467995	45475179	+	5553	protein_cod	FosB proto-oncogene, AP-1 transcription factor subunit [Source:HGNC Symbol;Acc:HGNC:3797]
ENSG0000	5229.337	5242.559	4796.561	1256.912	1354.449	1266.186	5089.486	1292.516	1.977385484	3.70E-177	5.23E-174	ANLN	7	36389806	36453791	+	6012	protein_cod	anillin actin binding protein [Source:HGNC Symbol;Acc:HGNC:14082]
ENSG0000	3450.993	3472.223	3489.636	800.992	710.4264	655.8149	3470.951	722.4111	2.265387208	2.18E-171	2.91E-168	PLK1	16	23677656	23690367	+	4760	protein_cod	polo like kinase 1 [Source:HGNC Symbol;Acc:HGNC:9077]
ENSG0000	15467.39	15351.21	14631.71	5385.25	5125.803	5274.142	15150.1	5261.732	1.525816858	6.61E-170	8.36E-167	TUBB4B	9	137241213	137243707	+	2111	protein_cod	tubulin beta 4B class IVb [Source:HGNC Symbol;Acc:HGNC:20771]
ENSG0000	2963.565	3060.493	2754.561	667.0114	649.4807	651.3596	2926.206	655.9506	2.157749867	8.50E-167	1.02E-163	KIF2C	1	44739818	44767767	+	3646	protein_cod	kinesin family member 2C [Source:HGNC Symbol;Acc:HGNC:6393]
ENSG0000	2272.956	2156.139	2306.537	402.9057	452.9991	394.7364	2245.21	416.8804	2.428901534	3.39E-166	3.88E-163	DLGAP5	14	55148112	55191678	-	3667	protein_cod	DLG associated protein 5 [Source:HGNC Symbol;Acc:HGNC:16864]
ENSG0000	1694.199	1671.811	1667.146	115.6667	177.3792	106.0353	1677.718	133.0271	3.656024463	6.80E-164	7.43E-161	IFIT1	10	89392546	89406486	+	4613	protein_cod	interferon induced protein with tetratricopeptide repeats 1 [Source:HGNC Symbol;Acc:HGNC:5407]
ENSG0000	920.4701	1019.473	1033.383	3948.091	3798.643	3775.391	991.1085	3840.708	-1.954705998	3.54E-157	3.70E-154	DUSP5	10	110497838	110511544	+	2615	protein_cod	dual specificity phosphatase 5 [Source:HGNC Symbol;Acc:HGNC:3071]
ENSG0000	6386.851	6741.174	7038.934	2124.412	2013.936	2015.562	6722.32	2051.303	1.712468059	2.41E-155	2.42E-152	STAT1	2	190964358	191020960	-	6053	protein_cod	signal transducer and activator of transcription 1 [Source:HGNC Symbol;Acc:HGNC:11362]
ENSG0000	3909.689	4176.417	3782.315	12871.78	14584.21	15106.02	3956.14	14187.33	-1.842374547	1.12E-151	1.07E-148	FOS	14	75278774	75282230	+	3238	protein_cod	Fos proto-oncogene, AP-1 transcription factor subunit [Source:HGNC Symbol;Acc:HGNC:3796]
ENSG0000	5485.878	5364.937	5349.274	1573.067	1630.069	1742.008	5400.03	1648.382	1.711631437	1.16E-151	1.07E-148	HMGB2	4	173331695	173335125	-	2775	protein_cod	high mobility group box 2 [Source:HGNC Symbol;Acc:HGNC:5000]
ENSG0000	9672.632	9644.027	9201.382	2719.132	3124.602	2772.065	9506.014	2871.933	1.726754755	2.75E-148	2.45E-145	ARL6IP1	16	18791667	18801678	-	3666	protein_cod	ADP ribosylation factor like GTPase 6 interacting protein 1 [Source:HGNC Symbol;Acc:HGNC:697]
ENSG0000	3242.682	3067.753	2840.114	727.7364	665.8541	635.3207	3050.183	676.3038	2.174051171	1.13E-147	9.66E-145	BUB1	2	110637698	110678114	-	6032	protein_cod	BUB1 mitotic checkpoint serine/threonine kinase [Source:HGNC Symbol;Acc:HGNC:1148]
ENSG0000	1310.413	1419.795	1380.095	4399.191	4514.528	4563.082	1370.101	4492.267	-1.713326868	5.94E-146	4.92E-143	JUNB	19	12791496	12793315	+	1820	protein_cod	JunB proto-oncogene, AP-1 transcription factor subunit [Source:HGNC Symbol;Acc:HGNC:6205]
ENSG0000	15646.96	16332.31	16159.27	4792.458	4098.823	3819.053	16046.18	4236.778	1.921430458	2.35E-145	1.88E-142	MKI67	10	128096659	128126385	-	12807	protein_cod	marker of proliferation Ki-67 [Source:HGNC Symbol;Acc:HGNC:7107]
ENSG0000	3067.207	2913.224	2754.561	676.6503	742.2636	702.1497	2911.664	707.0212	2.041939809	5.93E-145	4.60E-142	CCNA2	4	121816444	121823933	-	2798	protein_cod	cyclin A2 [Source:HGNC Symbol;Acc:HGNC:1578]
ENSG0000	2690.605	2771.141	2402.22	557.128	526.6797	587.2038	2621.322	557.0039	2.234682471	9.78E-145	7.34E-142	CEP55	10	93496632	93529092	+	3271	protein_cod	centrosomal protein 55 [Source:HGNC Symbol;Acc:HGNC:1161]
ENSG0000	3332.984	3351.919	3451.363	907.9837	776.8298	891.9439	3378.755	858.9192	1.976101159	2.81E-141	2.05E-138	CIT	12	119685790	119877291	-	13230	protein_cod	citron rho-interacting serine/threonine kinase [Source:HGNC Symbol;Acc:HGNC:1985]
ENSG0000	2691.631	2651.874	2684.768	650.6253	688.5951	730.6633	2676.091	689.9612	1.954926976	1.55E-139	1.10E-136	UBE2C	20	45812576	45816957	+	1286	protein_cod	ubiquitin conjugating enzyme E2 C [Source:HGNC Symbol;Acc:HGNC:15937]
ENSG0000	2763.463	2645.652	2502.407	403.8696	544.8725	453.5459	2637.174	467.4293	2.495437644	2.69E-139	1.85E-136	HMMR	5	163460203	163491945	+	3936	protein_cod	hyaluronan mediated motility receptor [Source:HGNC Symbol;Acc:HGNC:5012]
ENSG0000	2085.167	2150.953	2076.896	5882.617	5992.687	5862.237	2104.339	5912.514	-1.490321385	3.25E-137	2.17E-134	HOXB6	17	48595751	48604992	-	2604	protein_cod	homeobox B6 [Source:HGNC Symbol;Acc:HGNC:5117]
ENSG0000	3850.171	3696.238	3545.921	1025.578	1069.733	940.9518	3697.443	1012.088	1.869554484	3.39E-136	2.20E-133	BIRC5	17	78214186	78225636	+	3815	protein_cod	baculoviral IAP repeat containing 5 [Source:HGNC Symbol;Acc:HGNC:593]
ENSG0000	2057.461	1902.048	2004.852	347.0002	426.6197	392.9543	1988.12	388.858	2.353076271	7.31E-136	4.62E-133	KIF14	1	200551497	200620734	-	7277	protein_cod	kinesin family member 14 [Source:HGNC Symbol;Acc:HGNC:19181]
ENSG0000	1877.882	1992.276	1858.513	320.9751	308.3669	390.2812	1909.557	339.8744	2.489273526	1.21E-135	7.46E-133	NEK2	1	211658657	211675630	-	3095	protein_cod	NIMA related kinase 2 [Source:HGNC Symbol;Acc:HGNC:7745]
ENSG0000	6811.684	7077.196	6661.828	2478.159	2491.495	2412.971	6850.236	2460.875	1.477104339	1.43E-135	8.60E-133	CENPF	1	214603195	214664588	+	11690	protein_cod	centromere protein F [Source:HGNC Symbol;Acc:HGNC:1857]
ENSG0000	406.3614	393.0623	417.6306	2022.24	2135.827	2477.127	405.6848	2211.731	-2.447370238	1.78E-135	1.04E-132	JUN	1	58780788	58784327	-	3540	protein_cod	Jun proto-oncogene, AP-1 transcription factor subunit [Source:HGNC Symbol;Acc:HGNC:6204]
ENSG0000	2008.205	1953.903	1885.529	382.6641	444.8124	389.3901	1949.213	405.6222	2.264444046	2.73E-135	1.56E-132	KNL1	15	40594020	40664342	+	10250	protein_cod	kinetochore scaffold 1 [Source:HGNC Symbol;Acc:HGNC:24054]
ENSG0000	103.6427	103.7104	108.0662	982.2032	1140.685	1002.434	105.1397	1041.774	-3.309460296	2.05E-133	1.14E-130	PDE4B	1	65792514	66374579	+	8644	protein_cod	phosphodiesterase 4B [Source:HGNC Symbol;Acc:HGNC:8781]
ENSG0000	10361.19	10511.05	10475.66	3917.246	3563.047	3465.305	10449.3	3648.533	1.518240853	1.10E-132	6.01E-130	NCAPD2	12	6493356	6531955	+	6487	protein_cod	non-SMC condensin I complex subunit D2 [Source:HGNC Symbol;Acc:HGNC:24305]
ENSG0000	2633.14	2675.728	2851.37	660.2642	742.2636	732.4455	2720.079	678.6076	2.002416922	1.60E-132	8.54E-130	IQGAP3	1	156525405	156572604	-	6742	protein_cod	IQ motif containing GTPase activating protein 3 [Source:HGNC Symbol;Acc:HGNC:20669]
ENSG0000	2148.79	2005.759	2170.329	521.4641	484.8364	487.4059	2108.292	497.9022	2.082502484	4.00E-132	2.09E-129	TROAP	12	49323236	49331731	+	3954	protein_cod	trophinin associated protein [Source:HGNC Symbol;Acc:HGNC:12327]
ENSG0000	1005.642	848.3508	938.8247	3414.096	3677.662	3567.776	930.9391	3553.178	-1.932408981	1.31E-131	6.69E-129	ZFP36	19	39406813	39409412	+	2010	protein_cod	ZFP36 ring finger protein [Source:HGNC Symbol;Acc:HGNC:12862]
ENSG0000	2057.461	2033.76	2037.497	500.2586	507.5773	452.6549	2042.906	486.8303	2.069616734	2.30E-130	1.15E-127	CCNB2	15	59105078	59125045	+	2801	protein_cod	cyclin B2 [Source:HGNC Symbol;Acc:HGNC:1580]
ENSG0000	2461.77	2517.051	2450.625	691.1086	631.2879	616.6086	2476.482	646.3351	1.938649991	2.38E-130	1.17E-127	HJURP	2	233833416	233854566	-	3821	protein_cod	Holliday junction recognition protein [Source:HGNC Symbol;Acc:HGNC:25444]
ENSG0000	1831.705	1696.702	1786.469	405.7974	359.3065	383.1527	1771.625	382.7522	2.211147049	1.58E-127	7.58E-125	GTSE1	22	46296741	46330810	+	4242	protein_cod	G2 and S-phase expressed 1 [Source:HGNC Symbol;Acc:HGNC:13698]
ENSG0000	1841.966	1877.158	1789.846	442.4252	442.9931	403.6469	1836.323	429.6884	2.096059348	7.47E-125	3.52E-122	CDCA8	1	37692418	37709719	+	2469	protein_cod	cell division cycle associated 8 [Source:HGNC Symbol;Acc:HGNC:14629]
ENSG0000	747.0482	816.2006	764.3429	31.80835	24.56019	50.79001	775.8639	35.71952	4.436107087	1.78E-123	8.23E-121	SAMD9	7	93099513	93118023	-	6855	protein_cod	sterile alpha motif domain containing 9 [Source:HGNC Symbol;Acc:HGNC:1348]
ENSG0000	3604.918	3487.78	3380.444	1167.27	1094.293	1120.053	3491.047	1127.206	1.631270202	8.39E-120	3.80E-117	TACC3	4	1721490	1745176	+	6528	protein_cod	transforming acidic coiled-coil containing protein 3 [Source:HGNC Symbol;Acc:HGNC:11524]
ENSG0000	974.8568	958.2838	894.9228	3188.546	3477.542	3120.467	942.6878	3262.185	-1.790482144	1.67E-118	7.42E-116	AREG	4	74445134	74455009	+	1648	protein_cod	amphiregulin [Source:HGNC Symbol;Acc:HGNC:651]
ENSG0000	2997.428	2854.109	2843.491	685.3253	760.4564	833.1344	2898.343	759.6387	1.931094388	2.85E-118	1.24E-115	PBK	8	27809620	27838095	-	2165	protein_cod	PDZ binding kinase [Source:HGNC Symbol;Acc:HGNC:18282]
ENSG0000	2708.05	2628.021	2505.784	637.1308	724.9805	597.8965	2613.951	653.336	2.000518841	1.29E-117	5.54E-115	ARHGAP11A	15	32615144	32639949	+	6428	protein_cod	Rho GTPase activating protein 11A [Source:HGNC Symbol;Acc:HGNC:15783]
ENSG0000	2070.801	1901.011	1823.616	384.5918	444.8124	360.8764	1931.809	396.7602	2.283852502	1.65E-117	6.94E-115	PRR11	17	59155499	59204705	+	4080	protein_cod	proline rich 11 [Source:HGNC Symbol;Acc:HGNC:25619]
ENSG0000	2400.2	2368.745	2228.864	494.4752	411.1558	338.6001	2332.603	414.7437	2.493469847	1.51E-116	6.26E-114	AURKA	20	56369389	56392337	-	2928	protein_cod	aurora kinase A [Source:HGNC Symbol;Acc:HGNC:11393]
ENSG0000	2092.351	2249.478	2289.652	540.7419	468.463	420.577	2210.493	476.5939	2.214682243	1.81E-116	7.37E-114	NEURL1B	5	172641266	172691540	+	6424	protein_cod	neuralized E3 ubiquitin protein ligase 1B [Source:HGNC Symbol;Acc:HGNC:35422]
ENSG0000	2234.988	2110.566	2005.978	587.0086	540.3243	547.1065	2117.157	558.1464	1.924129183	8.73E-112	3.48E-109	PTTG1	5	160421822	160428744	+	2013	protein_cod	pituitary tumor-transforming 1 [Source:HGNC Symbol;Acc:HGNC:9690]
ENSG0000	1676.754	1644.846	1573.713	329.6501	301.9994	380.4796	1631.771	337.3764	2.273552223	8.85E-112	3.48E-109	CDCA3	12	6844793	6852066	-	5653	protein_cod	cell division cycle associated 3 [Source:HGNC Symbol;Acc:HGNC:14624]
ENSG0000	3311.435	3253.394	3199.208	987.9866	1109.757	1024.711	3254.679	1040.818	1.64459484	1.07E-111	4.15E-109	CD109	6	73695785	73828316	+	9782	protein_cod	CD109 molecule [Source:HGNC Symbol;Acc:HGNC:21685]
ENSG0000	2397.122	2373.913	2374.078	740.267	748.6311	708.387	2381.71	732.4284	1.701428647	5.54E-111	2.11E-108	KLK6	19	50958631	50969673	-	1976	protein_cod	kallikrein related peptidase 6 [Source:HGNC Symbol;Acc:HGNC:6367]
ENSG0000	3291.938	3245.097	3065.251	932.081	1051.54	994.415	3200.762	992.6787	1.688811547	1.06E-110	3.96E-108	CKAP2	13	52455429	52476628	+	4547	protein_cod	cytoskeleton associated protein 2 [Source:HGNC Symbol;Acc:HGNC:1990]
ENSG0000	3621.337	3543.783	3455.865	783.642	994.233	973.0297	3540.328	916.9683	1.948193756	1.17E-110	4.31E-108	KIF23	15	69414246	69448427	+	6576	protein_cod	kinesin family member 23 [Source:HGNC Symbol;Acc:HGNC:6392]
ENSG0000	1442.788	1463.353	1463.396	4721.13	6070.006	5614.524	1456.512	5468.553	-1.908811174	3.94E-110	1.43E-107	PDLIM5	4	94451857	94668227	+	11090	protein_cod	PDZ and LIM domain 5 [Source:HGNC Symbol;Acc:HGNC:17468]
ENSG0000	1735.245	1669.737	1583.845	394.2307	410.2462	418.7948	1662.942	407.7573	2.027819154	7.19E-110	2.58E-107	KNSTRN	15	40382721	40394246	+	3654	protein_cod	kinetochore localized astrin (SPAG5) binding protein [Source:HGNC Symbol;Acc:HGNC:30767]
ENSG0000	2487.424	2430.971	2470.888	651.5892	497.5713	513.2464	2463.094	554.1357	2.153432948	2.00E-109	7.07E-107	SUSD2	22	24181259	24189110	+	5279	protein_cod	sushi domain containing 2 [Source:HGNC Symbol;Acc:HGNC:30667]

ENSG0000	2762.436	2808.477	2598.09	734.4837	839.5948	729.7723	2723.001	767.9502	1.826111549	3.71E-109	1.29E-106	NCAPG2	7	158631311	158704829	-	6988	protein_cod	non-SMC condensin II complex subunit G2 [Source:HGNC Symbol;Acc:HGNC:21904]
ENSG0000	3343.246	3223.318	3335.417	934.9726	927.8296	1091.54	3300.66	984.7806	1.744375554	1.09E-106	3.74E-104	S100A10	1	151982915	151994390	-	1543	protein_cod	S100 calcium binding protein A10 [Source:HGNC Symbol;Acc:HGNC:10487]
ENSG0000	467.9313	510.255	461.5325	2145.618	3010.898	2866.517	479.9063	2674.344	-2.478317024	1.08E-104	3.66E-102	SEMA3C	7	80742538	80922359	-	6268	protein_cod	semaphorin 3C [Source:HGNC Symbol;Acc:HGNC:10725]
ENSG0000	5146.218	4952.17	4902.376	1657.89	1882.948	1816.857	5000.255	1785.898	1.485094181	8.34E-104	2.78E-101	MALT1	18	58671386	58754477	+	11527	protein_cod	MALT1 paracaspase [Source:HGNC Symbol;Acc:HGNC:6819]
ENSG0000	9705.469	8989.615	9005.513	3161.557	2804.41	2488.711	9233.532	2818.226	1.712451555	1.00E-103	3.29E-101	TUBA1B	12	49127782	49131397	-	3574	protein_cod	tubulin alpha 1b [Source:HGNC Symbol;Acc:HGNC:18809]
ENSG0000	2259.615	2203.845	2193.968	483.8724	483.9268	345.7285	2219.143	437.8426	2.342692512	1.01E-103	3.29E-101	FAM83D	20	38926312	38953106	+	2475	protein_cod	family with sequence similarity 83 member D [Source:HGNC Symbol;Acc:HGNC:16122]
ENSG0000	5009.738	4841.2	4908.004	1819.823	1549.111	1691.218	4919.647	1686.718	1.544660339	1.12E-103	3.58E-101	TK1	17	78174075	78187233	-	1861	protein_cod	thymidine kinase 1 [Source:HGNC Symbol;Acc:HGNC:11830]
ENSG0000	1531.038	1516.246	1582.719	389.4113	379.3186	412.5575	1543.334	393.7624	1.97024751	8.62E-103	2.72E-100	AURKB	17	8204733	8210600	-	2241	protein_cod	aurora kinase B [Source:HGNC Symbol;Acc:HGNC:11390]
ENSG0000	3995.887	4015.665	3686.632	1255.948	1224.371	1051.442	3899.395	1177.254	1.728434983	1.06E-102	3.30E-100	ECT2	3	172750682	172821474	+	5499	protein_cod	epithelial cell transforming 2 [Source:HGNC Symbol;Acc:HGNC:3155]
ENSG0000	2417.645	2253.626	2260.384	6104.311	6422.946	7001.893	2310.552	6509.717	-1.494325243	6.99E-102	2.15E-99	KLF6	10	3775996	3785281	-	6139	protein_cod	Kruppel like factor 6 [Source:HGNC Symbol;Acc:HGNC:2235]
ENSG0000	167.2649	147.2687	166.602	1024.614	1012.426	927.586	160.3785	988.2087	-2.623778846	1.25E-101	3.81E-99	TESC	12	117038923	117099479	-	1926	protein_cod	tescalcin [Source:HGNC Symbol;Acc:HGNC:26065]
ENSG0000	2604.407	2564.757	2564.32	870.3921	865.0646	895.5081	2577.828	876.9883	1.555450975	2.29E-101	6.87E-99	SPAG5	17	28577565	28599279	-	5472	protein_cod	sperm associated antigen 5 [Source:HGNC Symbol;Acc:HGNC:13452]
ENSG0000	207.2853	257.2017	216.1323	1179.801	1248.931	1387.369	226.8731	1272.034	-2.486882309	1.44E-99	4.27E-97	LONRF2	2	100273291	100322733	-	13933	protein_cod	LON peptidase N-terminal domain and ring finger 2 [Source:HGNC Symbol;Acc:HGNC:24788]
ENSG0000	1769.109	1820.117	1668.271	501.2224	464.8244	463.3475	1752.499	476.4648	1.879734576	2.90E-99	8.50E-97	KIF4A	X	70290090	70420832	+	4533	protein_cod	kinesin family member 4A [Source:HGNC Symbol;Acc:HGNC:13339]
ENSG0000	1761.925	1804.56	1694.162	4754.866	5002.093	4513.183	1753.549	4756.714	-1.439423157	5.58E-99	1.61E-96	SYT1	12	78863993	79452008	+	6494	protein_cod	synaptotagmin 1 [Source:HGNC Symbol;Acc:HGNC:11509]
ENSG0000	1881.987	2025.464	1712.173	372.0613	449.3606	449.9817	1873.208	423.8012	2.143286181	1.78E-98	5.08E-96	KIF20B	10	89701610	89774939	+	6851	protein_cod	kinesin family member 20B [Source:HGNC Symbol;Acc:HGNC:7212]
ENSG0000	1420.212	1355.495	1276.531	263.1418	318.3729	264.6427	1350.746	282.0525	2.259559947	6.32E-98	1.79E-95	SGO2	2	200510008	200583782	+	6022	protein_cod	shugoshin 2 [Source:HGNC Symbol;Acc:HGNC:30812]
ENSG0000	15.39248	24.89049	10.1312	578.3336	711.336	783.2355	16.80472	690.9683	-5.350084255	2.70E-97	7.55E-95	RF00157	9	21524307	21524382	-	76	snoRNA	
ENSG0000	1803.998	1679.071	1740.315	441.4613	483.9268	400.0827	1741.128	441.8236	1.978763972	3.60E-97	9.94E-95	CCNF	16	2429394	2458854	+	6309	protein_cod	cyclin F [Source:HGNC Symbol;Acc:HGNC:1591]
ENSG0000	554.1291	660.6351	627.0088	2909.982	2710.718	2289.115	613.9243	2636.605	-2.102615284	1.80E-96	4.92E-94	THSD4	15	71096952	71783383	+	15837	protein_cod	thrombospondin type 1 domain containing 4 [Source:HGNC Symbol;Acc:HGNC:25835]
ENSG0000	1038.479	943.7644	992.8578	121.4501	145.5419	175.5374	991.7004	147.5098	2.746310375	3.56E-96	9.60E-94	PIF1	15	64815632	64825668	-	4656	protein_cod	PIF1 5'-to-3' DNA helicase [Source:HGNC Symbol;Acc:HGNC:26220]
ENSG0000	1470.495	1394.904	1267.526	3927.849	4231.63	4419.622	1377.642	4193.034	-1.605326494	5.60E-96	1.49E-93	IER2	19	13150415	13154908	+	3072	protein_cod	immediate early response 2 [Source:HGNC Symbol;Acc:HGNC:28871]
ENSG0000	2674.186	2521.199	2667.883	850.1504	854.149	745.8112	2621.089	816.7035	1.682669479	6.16E-96	1.63E-93	FOXM1	12	2857681	2877155	-	4899	protein_cod	forkhead box M1 [Source:HGNC Symbol;Acc:HGNC:3818]
ENSG0000	317.085	282.0922	294.9305	1443.906	1299.871	1275.097	298.0359	1339.625	-2.167711052	1.97E-94	5.14E-92	CSRNP1	3	39141855	39154562	-	3214	protein_cod	cysteine and serine rich nuclear protein 1 [Source:HGNC Symbol;Acc:HGNC:14300]
ENSG0000	2369.415	2362.522	2222.11	628.4558	482.1075	613.9354	2318.016	574.8329	2.012225903	2.08E-94	5.37E-92	ESPL1	12	53268299	53293643	+	8890	protein_cod	extra spindle pole bodies like 1, separase [Source:HGNC Symbol;Acc:HGNC:16856]
ENSG0000	10905.06	10936.26	11113.93	4788.602	4446.305	4156.762	10985.08	4463.889	1.299347431	2.45E-94	6.26E-92	SLC2A1	1	42925375	42959176	-	4724	protein_cod	solute carrier family 2 member 1 [Source:HGNC Symbol;Acc:HGNC:11005]
ENSG0000	2123.136	2199.697	2267.138	5258.98	5691.598	5536.111	2196.657	5495.563	-1.323257511	4.13E-94	1.05E-91	SOX4	6	21592768	21598619	+	5852	protein_cod	SRY-box 4 [Source:HGNC Symbol;Acc:HGNC:11200]
ENSG0000	5800.911	5485.241	5637.451	2122.484	2348.682	2268.621	5641.201	2246.596	1.328056531	2.37E-93	5.92E-91	HMGN2	1	26472450	26475972	+	2509	protein_cod	high mobility group nucleosomal binding domain 2 [Source:HGNC Symbol;Acc:HGNC:4986]
ENSG0000	657.7718	841.0911	781.2282	2689.251	2899.922	2690.98	760.0304	2760.051	-1.860979326	6.44E-93	1.59E-90	RBM12B	8	93729356	93741017	-	7618	protein_cod	RNA binding motif protein 12B [Source:HGNC Symbol;Acc:HGNC:32310]
ENSG0000	59.51757	41.48415	41.6505	607.2503	679.4987	842.045	47.55074	709.598	-3.896234102	1.00E-91	2.46E-89	AADAC	3	151814037	151828488	+	1668	protein_cod	arylacetamide deacetylase [Source:HGNC Symbol;Acc:HGNC:17]
ENSG0000	1225.241	1228.968	1230.378	312.3001	266.5236	296.7206	1228.196	291.8481	2.073888698	9.39E-91	2.28E-88	CDKN3	14	54396849	54420218	+	1836	protein_cod	cyclin dependent kinase inhibitor 3 [Source:HGNC Symbol;Acc:HGNC:1791]
ENSG0000	2340.683	2006.796	2055.508	595.6836	582.1676	637.9938	2134.329	605.2817	1.818052988	1.63E-90	3.91E-88	KIF18B	17	44924709	44947711	-	4655	protein_cod	kinesin family member 18B [Source:HGNC Symbol;Acc:HGNC:27102]
ENSG0000	8809.627	8962.65	8246.798	3397.71	3713.137	3296.896	8673.025	3469.248	1.321986744	3.79E-90	9.02E-88	NUCKS1	1	205712819	205750276	-	6546	protein_cod	nuclear casein kinase and cyclin dependent kinase substrate 1 [Source:HGNC Symbol;Acc:HGNC:29923]
ENSG0000	1760.899	1671.811	1691.911	528.2113	465.734	485.6238	1708.207	493.1897	1.792964317	4.81E-90	1.13E-87	TACSTD2	1	58575423	58577773	-	2351	protein_cod	tumor associated calcium signal transducer 2 [Source:HGNC Symbol;Acc:HGNC:11530]
ENSG0000	3410.973	3405.849	2988.704	934.0087	1098.841	1006.89	3268.509	1013.247	1.68953257	1.86E-89	4.33E-87	SMC4	3	160399274	160434962	+	8457	protein_cod	structural maintenance of chromosomes 4 [Source:HGNC Symbol;Acc:HGNC:14013]
ENSG0000	843.5077	843.1653	816.1246	142.6556	150.0901	159.4985	834.2659	150.7481	2.467557751	2.49E-89	5.75E-87	SPC25	2	168834132	168913371	-	1841	protein_cod	SPC25, NDC80 kinetochore complex component [Source:HGNC Symbol;Acc:HGNC:24031]
ENSG0000	1370.957	1366.903	1358.707	303.6251	348.3909	366.2227	1365.522	339.4129	2.00713387	3.35E-89	7.66E-87	DEPDC1B	5	60596912	60700190	-	3806	protein_cod	DEP domain containing 1B [Source:HGNC Symbol;Acc:HGNC:24902]
ENSG0000	9.235486	12.44524	10.1312	574.478	668.5831	664.7254	10.60398	635.9288	-5.905580849	5.55E-89	1.26E-86	EGR3	8	22687659	22693302	-	4855	protein_cod	early growth response 3 [Source:HGNC Symbol;Acc:HGNC:3240]
ENSG0000	1495.123	1536.988	1550.074	429.8946	452.9991	394.7364	1527.395	425.8767	1.842783057	9.43E-88	2.12E-85	NCAPH	2	96335787	96373845	+	3278	protein_cod	non-SMC condensin I complex subunit H [Source:HGNC Symbol;Acc:HGNC:1112]
ENSG0000	533.6058	460.474	476.1665	14.45834	13.64455	12.47474	490.0821	13.52588	5.181659433	3.07E-87	6.82E-85	KLK5	19	50943303	50953093	-	1653	protein_cod	kallikrein related peptidase 5 [Source:HGNC Symbol;Acc:HGNC:6366]
ENSG0000	763.4668	557.9618	622.5061	2548.523	2858.079	3168.584	647.9782	2858.395	-2.140916419	5.03E-87	1.11E-84	CYR61	1	85580761	85583962	+	2455	protein_cod	cysteine rich angiogenic inducer 61 [Source:HGNC Symbol;Acc:HGNC:2654]
ENSG0000	832.2199	874.2784	844.2668	2479.123	2743.465	2441.485	850.255	2554.691	-1.587173332	8.69E-87	1.90E-84	PLCXD2	3	111674676	111846447	+	8974	protein_cod	phosphatidylinositol specific phospholipase C X domain containing 2 [Source:HGNC Symbol;Acc:HGNC:26462]
ENSG0000	583.8879	599.4459	611.2492	1825.606	1920.243	2023.581	598.1943	1923.144	-1.685278805	1.11E-86	2.41E-84	TARID	6	133502252	133892802	-	7862	antisense	TCF21 antisense RNA inducing promoter demethylation [Source:HGNC Symbol;Acc:HGNC:50506]
ENSG0000	1445.867	1395.942	1234.881	345.0724	326.5596	335.9269	1358.896	335.853	2.017250882	2.28E-86	4.89E-84	PIMREG	17	6444415	6451469	+	2823	protein_cod	PICALM interacting mitotic regulator [Source:HGNC Symbol;Acc:HGNC:25483]
ENSG0000	400.2044	468.7709	441.2701	1735.001	1912.966	2298.025	436.7485	1981.997	-2.182583735	5.76E-86	1.22E-83	ATF3	1	212565334	212620777	+	4040	protein_cod	activating transcription factor 3 [Source:HGNC Symbol;Acc:HGNC:785]
ENSG0000	1902.51	1862.638	1917.048	640.9864	640.3843	621.9549	1894.066	634.4419	1.578002127	1.16E-85	2.45E-83	KIFC1	6	33391536	33409924	+	3345	protein_cod	kinesin family member C1 [Source:HGNC Symbol;Acc:HGNC:6389]
ENSG0000	9084.639	9242.668	8598.013	4006.888	3702.222	3559.756	8975.107	3756.289	1.256886234	1.59E-85	3.32E-83	LMNB2	19	2427638	2456996	-	5432	protein_cod	lamin B2 [Source:HGNC Symbol;Acc:HGNC:6638]
ENSG0000	12421.73	12597.7	13030.98	5049.816	4051.522	4086.368	12683.47	4395.902	1.528924327	3.45E-85	7.15E-83	ITGB4	17	75721328	75757818	+	6761	protein_cod	integrin subunit beta 4 [Source:HGNC Symbol;Acc:HGNC:6158]
ENSG0000	2717.285	2671.579	2605.97	971.6004	983.3174	878.5781	2664.945	944.4987	1.496906923	3.61E-85	7.40E-83	MAD2L1	4	120055608	120067074	-	5748	protein_cod	mitotic arrest deficient 2 like 1 [Source:HGNC Symbol;Acc:HGNC:6763]
ENSG0000	38.99427	47.70677	59.66152	563.8753	556.6977	567.6007	48.78752	562.7246	-3.534189545	8.64E-85	1.76E-82	AADACP1	3	151770428	151784894	+	2012	transcribed_	arylacetamide deacetylase pseudogene 1 [Source:HGNC Symbol;Acc:HGNC:50305]
ENSG0000	2846.582	2841.664	2753.435	954.2504	1019.703	849.1734	2813.894	941.0422	1.580575971	2.16E-84	4.37E-82	NCAPG	4	17810902	17844862	+	4921	protein_cod	non-SMC condensin I complex subunit G [Source:HGNC Symbol;Acc:HGNC:24304]
ENSG0000	805.5396	855.6105	834.1356	164.8251	141.9033	162.1716	831.7619	156.3	2.412317989	6.70E-84	1.34E-81	FAT2	5	151504093	151568944	-	14537	protein_cod	FAT atypical cadherin 2 [Source:HGNC Symbol;Acc:HGNC:3596]
ENSG0000	1816.312	1926.939	1713.299	452.0641	551.2399	501.6628	1818.85	501.6556	1.857778864	8.11E-84	1.61E-81	CAV1	7	116524785	116561184	+	4082	protein_cod	caveolin 1 [Source:HGNC Symbol;Acc:HGNC:1527]
ENSG0000	6953.295	7473.369	6946.627	3072.879	2732.549	2979.681	7124.43	2928.37	1.282837908	1.80E-83	3.54E-81	UBE2S	19	55399745	55407777	-	2788	protein_cod	ubiquitin conjugating enzyme E2 S [Source:HGNC Symbol;Acc:HGNC:17895]
ENSG0000	806.5657	855.6105	809.3704	2246.826	2427.821	2436.139	823.8489	2370.262	-1.524607408	5.24E-83	1.02E-80	MEIS2	15	36889204	37101299	-	10798	protein_cod	Meis homeobox 2 [Source:HGNC Symbol;Acc:HGNC:7001]
ENSG0000	1849.149	1784.855	1822.491	619.7808	617.6434	579.1844	1818.832	605.5362	1.587032795	1.01E-81	1.94E-79	NMU	4	55595229	55636698	-	975	protein_cod	neuromedin U [Source:HGNC Symbol;Acc:HGNC:7859]
ENSG0000	715.2371	722.8613	872.409	124.3417	109.1564	118.51	770.1691	117.3361	2.714529292	1.01E-81	1.94E-79	NCAM1	11	112961247	113278436	+	12737	protein_cod	neural cell adhesion molecule 1 [Source:HGNC Symbol;Acc:HGNC:7656]
ENSG0000	1033.348	945.8386	1031.131	2734.554	3204.65	3070.568	1003.439	3003.258	-1.581948903	1.85E-81	3.52E-79	GNAI1	7	79768028	80226181	+	24206	protein_cod	G protein subunit alpha i1 [Source:HGNC Symbol;Acc:HGNC:4384]
ENSG0000	414.5707	441.8062	364.7233	1440.051	1494.533	1513.899	407.0334	1482.828	-1.863850486	1.09E-80	2.06E-78	DUSP1	5	172768090	172771195	-	2019	protein_cod	dual specificity phosphatase 1 [Source:HGNC Symbol;Acc:HGNC:3064]
ENSG0000	924.5747	881.5381	968.0926	2535.993	2578.82	2452.177	924.7352	2522.33	-1.448073097	1.19E-80	2.23E-78	BHLHE40	3	4979116	4985323	+	3837	protein_cod	basic helix-loop-helix family member e40 [Source:HGNC Symbol;Acc:HGNC:1046]
ENSG0000	1100.049	1133.554	1107.678	248.6834	291.9934	284.2459	1113.76	274.9742	2.017015683	3.66E-80	6.82E-78	PSRC1	1	109279556	109283186	-	2855	protein_cod	proline and serine rich coiled-coil 1 [Source:HGNC Symbol;Acc:HGNC:24472]
ENSG0000	10493.56	10237.25	10311.31	4564.98	5023.924	4860.693	10347.38	4816.532	1.103086785	5.04E-80	9.31E-78	PLK2	5	58453982	58460260	-	4768	protein_cod	polo like kinase 2 [Source:HGNC Symbol;Acc:HGNC:19699]
ENSG0000	864.031	833.8314	836.387	161.9334	186.4755	177.3195	844.7498	175.2428	2.26829911	2.88E-79	5.28E-77	KLK10	19	51012739	51020175	-	3853	protein_cod	kallikrein related peptidase 10 [Source:HGNC Symbol;Acc:HGNC:6358]
ENSG0000	3047.71	3126.868	2932.42	8061.97	10842.87	10127.71	3035.666	9677.516	-1.672629312	3.52E-79	6.41E-77	KITLG	12	88492793	88580851	-	6205	protein_cod	KIT ligand [Source:HGNC Symbol;Acc:HGNC:6343]
ENSG0000	3173.929	3208.799	3168.815	1053.531	940.5645	768.0876	3183.847	920.7277	1.790763183	6.12E-79	1.10E-76	RACGAP1	12	49976923	50033136	-	4487	protein_cod	Rac GTPase activating protein 1 [Source:HGNC Symbol;Acc:HGNC:9804]
ENSG0000	4989.215	4529.032	4519.642	937.8643	1347.172	1313.412	4679.296	1199.483	1.963264577	2.22E-78	3.97E-76	RRM2	2	10120698	10131419	+	5323	protein_cod	ribonucleotide reductase regulatory subunit M2 [Source:HGNC Symbol;Acc:HGNC:10452]
ENSG0000	526.4227	516.4776	569.5987	1768.737	1613.696	1792.798	537.4997	1725.077	-1.683026028	8.94E-77	1.59E-74	R3HDM4	19	896503	913245	-	2957	protein_cod	R3H domain containing 4 [Source:HGNC Symbol;Acc:HGNC:28270]
ENSG0000	2136.476	2084.578	2103.913	791.3531	693.1433	749.3755	2108.322	744.6239	1.501937007	7.66E-76	1.35E-73	KIF22	16	29790719	29805385	+	4312	protein_cod	kinesin family member 22 [Source:HGNC Symbol;Acc:HGNC:6391]
ENSG0000	16400.17	15972.43	15730.38	7730.392	7298.926	6708.737	16034.33	7246.018	1.146041911	1.21E-75	2.12E-73	TUBB	6	30720201	30725426	+	3359	protein_cod	tubulin beta class I [Source:HGNC Symbol;Acc:HGNC:20778]

ENSG0000	1634.681	1742.334	1848.381	4822.338	6460.241	5684.026	1741.799	5655.535	-1.699462412	4.15E-75	7.22E-73	MAN1A1	6	119177209	119349761	-	5014	protein_cod	mannosidase alpha class 1A member 1 [Source:HGNC Symbol;Acc:HGNC:6821]
ENSG0000	698.8184	418.9899	473.9151	9871.191	10140.63	11189.84	530.5745	10400.55	-4.292686165	1.12E-72	1.93E-70	NR4A1	12	52022832	52059507	+	7295	protein_cod	nuclear receptor subfamily 4 group A member 1 [Source:HGNC Symbol;Acc:HGNC:7980]
ENSG0000	2463.822	2641.503	2549.686	978.3477	1009.697	895.5081	2551.67	961.1842	1.408783168	1.29E-72	2.19E-70	UBALD2	17	76265202	76271299	+	1797	protein_cod	UBA like domain containing 2 [Source:HGNC Symbol;Acc:HGNC:28438]
ENSG0000	1868.647	2122.951	2108.416	5000.658	5117.617	4696.74	2033.338	4938.338	-1.280385315	2.26E-71	3.82E-69	HOXB9	17	48621159	48626356	-	2581	protein_cod	homeobox B9 [Source:HGNC Symbol;Acc:HGNC:5120]
ENSG0000	3587.473	3378.884	3520.03	1504.631	1339.895	1306.283	3495.462	1383.603	1.337516062	2.33E-71	3.90E-69	H2AFX	11	119093854	119095467	-	1614	protein_cod	H2A histone family member X [Source:HGNC Symbol;Acc:HGNC:4739]
ENSG0000	75.93622	86.07961	67.54134	555.2002	539.4146	589.877	76.51906	561.4973	-2.872949332	8.38E-71	1.40E-68	AL391684.1	10	99430919	99461743	+	6898	bidirectiona	novel transcript
ENSG0000	1489.992	1436.389	1392.477	3582.777	4466.317	4045.38	1439.619	4031.491	-1.485571811	1.85E-70	3.07E-68	PPP3CA	4	101023409	101348278	-	5213	protein_cod	protein phosphatase 3 catalytic subunit alpha [Source:HGNC Symbol;Acc:HGNC:9314]
ENSG0000	3065.155	3131.016	2898.649	1179.801	1164.335	1290.245	3031.607	1211.46	1.323254825	2.03E-70	3.34E-68	LMNB1	5	126776623	126837020	+	3633	protein_cod	lamin B1 [Source:HGNC Symbol;Acc:HGNC:6637]
ENSG0000	1858.385	1820.117	1869.77	344.1085	513.0352	484.7328	1849.424	447.2921	2.04632142	1.45E-69	2.37E-67	CENPE	4	103105806	103198409	-	9444	protein_cod	centromere protein E [Source:HGNC Symbol;Acc:HGNC:1856]
ENSG0000	1831.705	1751.668	1648.009	564.8391	523.0412	437.507	1743.794	508.4624	1.779258412	2.20E-69	3.54E-67	INCENP	11	62123973	62153163	+	4473	protein_cod	inner centromere protein [Source:HGNC Symbol;Acc:HGNC:6058]
ENSG0000	1160.593	1235.19	1258.52	347.0002	381.1378	328.7985	1218.101	352.3122	1.789574917	3.62E-69	5.80E-67	PHF19	9	120855652	120894896	-	6733	protein_cod	PHD finger protein 19 [Source:HGNC Symbol;Acc:HGNC:24566]
ENSG0000	135.4538	122.3782	104.6891	699.7836	689.5047	652.2507	120.8404	680.513	-2.490531056	3.67E-69	5.83E-67	ZBED2	3	111592900	111595443	-	2311	protein_cod	zinc finger BED-type containing 2 [Source:HGNC Symbol;Acc:HGNC:20710]
ENSG0000	5296.038	4850.534	5448.335	13042.39	12261.9	15242.35	5198.302	13515.55	-1.378625237	5.34E-69	8.43E-67	NEAT1	11	65422774	65445540	+	22767	lincRNA	nuclear paraspeckle assembly transcript 1 [Source:HGNC Symbol;Acc:HGNC:30815]
ENSG0000	1964.08	1904.122	1760.578	695.9281	647.6614	631.7565	1876.26	658.4487	1.511506465	1.60E-68	2.50E-66	BUB1B	15	40161023	40221136	+	4476	protein_cod	BUB1 mitotic checkpoint serine/threonine kinase B [Source:HGNC Symbol;Acc:HGNC:1149]
ENSG0000	2270.903	2440.305	2091.53	826.0531	842.3237	801.9476	2267.58	823.4415	1.461795178	2.70E-68	4.21E-66	CKS1B	1	154974653	154979249	+	2338	protein_cod	CDC28 protein kinase regulatory subunit 1B [Source:HGNC Symbol;Acc:HGNC:19083]
ENSG0000	2776.803	2575.128	2464.133	866.5365	1016.974	868.7765	2605.355	917.429	1.505826998	7.76E-68	1.20E-65	SLC12A2	5	128083766	128189688	+	10664	protein_cod	solute carrier family 12 member 2 [Source:HGNC Symbol;Acc:HGNC:10911]
ENSG0000	1719.853	1653.143	1482.533	401.9418	484.8364	348.4017	1618.509	411.7266	1.975374739	1.58E-67	2.44E-65	CDCA2	8	25458997	25507920	+	3908	protein_cod	cell division cycle associated 2 [Source:HGNC Symbol;Acc:HGNC:14623]
ENSG0000	230.8871	176.3076	171.1047	972.5643	877.7995	907.9829	192.7665	919.4489	-2.251661658	3.14E-67	4.80E-65	NAV3	12	77324641	78213008	+	13265	protein_cod	neuron navigator 3 [Source:HGNC Symbol;Acc:HGNC:15998]
ENSG0000	1126.729	1085.848	1028.88	268.9251	322.0114	269.098	1080.486	286.6782	1.914080456	8.42E-67	1.28E-64	NDC80	18	2571511	2616635	+	3600	protein_cod	NDC80, kinetochore complex component [Source:HGNC Symbol;Acc:HGNC:16909]
ENSG0000	444.3295	489.5129	512.1885	65.54447	64.58421	65.04686	482.0103	65.05852	2.888926378	6.29E-66	9.49E-64	NEDD9	6	11183298	11382348	-	10359	protein_cod	neural precursor cell expressed, developmentally down-regulated 9 [Source:HGNC Symbol;Acc:HGNC:7733]
ENSG0000	1230.372	1231.042	1150.454	268.9251	355.668	328.7985	1203.956	317.7972	1.920472042	8.46E-66	1.27E-63	NUF2	1	163266576	163355764	+	3362	protein_cod	NUF2, NDC80 kinetochore complex component [Source:HGNC Symbol;Acc:HGNC:14621]
ENSG0000	571.5739	555.8876	579.7299	1572.103	1867.484	1879.23	569.0638	1772.939	-1.639995889	1.43E-65	2.13E-63	PNRC1	6	89080751	89085160	+	2992	protein_cod	proline rich nuclear receptor coactivator 1 [Source:HGNC Symbol;Acc:HGNC:17278]
ENSG0000	2863.001	2530.533	2880.638	1091.123	1072.462	1115.598	2758.057	1093.061	1.335086908	3.13E-65	4.63E-63	ANO1	11	70078302	70189528	+	6021	protein_cod	anoctamin 1 [Source:HGNC Symbol;Acc:HGNC:21625]
ENSG0000	1197.535	1366.903	1281.034	3824.713	3211.018	3353.032	1281.824	3462.921	-1.433658096	3.71E-65	5.46E-63	HOXB-AS3	17	48549630	48606414	+	1913	antisense	HOXB cluster antisense RNA 3 [Source:HGNC Symbol;Acc:HGNC:40283]
ENSG0000	2501.79	2670.542	2696.025	6728.911	6118.217	5689.373	2622.786	6178.834	-1.236239805	2.04E-64	2.98E-62	TGM2	20	38127387	38166578	-	8036	protein_cod	transglutaminase 2 [Source:HGNC Symbol;Acc:HGNC:11778]
ENSG0000	2994.35	2696.47	2536.177	529.1752	801.39	744.0291	2742.332	691.5315	1.986735726	2.50E-64	3.64E-62	DEPDC1	1	68474152	68497221	-	5904	protein_cod	DEP domain containing 1 [Source:HGNC Symbol;Acc:HGNC:22949]
ENSG0000	4174.44	4107.968	3819.463	985.0949	1389.925	1329.451	4033.957	1234.824	1.707345186	1.01E-63	1.47E-61	CDK1	10	60778331	60794852	+	3931	protein_cod	cyclin dependent kinase 1 [Source:HGNC Symbol;Acc:HGNC:1722]
ENSG0000	2189.836	2104.283	2198.471	4655.585	5481.471	5510.271	2164.197	5215.776	-1.26927474	6.00E-63	8.63E-61	ZFP36L1	14	68787660	68796253	-	5160	protein_cod	ZFP36 ring finger protein like 1 [Source:HGNC Symbol;Acc:HGNC:1107]
ENSG0000	761.4145	742.5662	828.5072	198.5612	158.2768	173.7553	777.496	176.8644	2.137176631	5.56E-62	7.95E-60	ADGRF1	6	46997703	47042363	-	12401	protein_cod	adhesion G protein-coupled receptor F1 [Source:HGNC Symbol;Acc:HGNC:18990]
ENSG0000	1939.452	1834.636	1631.123	420.2557	539.4146	563.1454	1801.737	507.6053	1.826764817	7.06E-62	1.00E-59	KIF11	10	92593286	92655395	+	4860	protein_cod	kinesin family member 11 [Source:HGNC Symbol;Acc:HGNC:6388]
ENSG0000	4962.534	5178.259	5161.284	11224.49	10658.21	9831.877	5100.692	10571.53	-1.051387223	8.62E-62	1.22E-59	DUSP4	8	29333064	29350668	-	7257	protein_cod	dual specificity phosphatase 4 [Source:HGNC Symbol;Acc:HGNC:3070]
ENSG0000	55.41291	43.55836	39.39912	406.7613	521.2219	442.8533	46.12346	456.9455	-3.304800678	1.25E-61	1.75E-59	TMEM200A	6	130365734	130443063	+	6137	protein_cod	transmembrane protein 200A [Source:HGNC Symbol;Acc:HGNC:21075]
ENSG0000	2806.561	2779.438	2673.512	1183.656	1095.203	983.7224	2753.17	1087.527	1.340731817	2.95E-61	4.11E-59	NCAPD3	11	134150119	134225454	-	9114	protein_cod	non-SMC condensin II complex subunit D3 [Source:HGNC Symbol;Acc:HGNC:28952]
ENSG0000	1272.445	1323.344	1185.351	315.1918	392.9631	389.3901	1260.38	365.8483	1.783584222	4.45E-61	6.18E-59	GAS2L3	12	100573683	100628286	+	6799	protein_cod	growth arrest specific 2 like 3 [Source:HGNC Symbol;Acc:HGNC:27475]
ENSG0000	804.5134	805.8296	771.097	176.3917	201.9394	213.8527	793.8133	197.3946	2.00646801	1.04E-60	1.44E-58	BORA	13	72727749	72756198	+	3189	protein_cod	bora, aurora kinase A activator [Source:HGNC Symbol;Acc:HGNC:24724]
ENSG0000	2141.606	1967.386	1854.01	712.3142	765.0046	763.6323	1987.667	746.9837	1.411926252	3.48E-60	4.78E-58	ZWINT	10	56357228	56361275	-	2378	protein_cod	ZW10 interacting kinetochore protein [Source:HGNC Symbol;Acc:HGNC:13195]
ENSG0000	590.0449	682.4142	723.8181	106.9917	130.9877	138.1132	665.4257	125.3642	2.405621875	5.79E-60	7.86E-58	PLEKHS1	10	113751454	113783429	+	5402	protein_cod	pleckstrin homology domain containing S1 [Source:HGNC Symbol;Acc:HGNC:26285]
ENSG0000	2542.837	2992.044	2993.207	7805.576	6482.982	7191.688	2842.696	7160.082	-1.332846818	1.71E-59	2.29E-57	AHNAK2	14	104937244	104978357	-	18771	protein_cod	AHNAK nucleoprotein 2 [Source:HGNC Symbol;Acc:HGNC:20125]
ENSG0000	947.1504	955.1725	971.4697	295.914	295.632	261.9695	957.9308	284.5052	1.752024018	3.48E-59	4.64E-57	DIAPH3	13	59665583	60163987	-	7442	protein_cod	diaphanous related formin 3 [Source:HGNC Symbol;Acc:HGNC:15480]
ENSG0000	52.33442	64.30043	69.79272	453.028	435.716	509.6822	62.14252	466.1421	-2.910457225	1.09E-58	1.45E-56	AC010618.3	19	17488990	17511889	-	2067	lincRNA	novel transcript
ENSG0000	2841.451	2764.918	2760.19	1310.889	1307.148	1265.295	2788.853	1294.444	1.107512579	4.55E-58	6.00E-56	FANCI	15	89243949	89317261	+	7766	protein_cod	FA complementation group I [Source:HGNC Symbol;Acc:HGNC:25568]
ENSG0000	852.7432	868.0558	844.2668	1954.768	2082.159	2044.075	855.0219	2027	-1.245310561	1.85E-57	2.43E-55	ZNF503	10	75397830	75401906	-	3347	protein_cod	zinc finger protein 503 [Source:HGNC Symbol;Acc:HGNC:23589]
ENSG0000	339.6606	410.6931	373.7288	1222.212	1133.407	1287.571	374.6942	1214.397	-1.696611231	4.28E-57	5.58E-55	GATA2-AS1	3	128489212	128502348	+	2283	antisense	GATA2 antisense RNA 1 [Source:HGNC Symbol;Acc:HGNC:51108]
ENSG0000	535.6582	555.8876	610.1235	1514.27	1511.816	1531.72	567.2231	1519.269	-1.422371292	5.83E-57	7.57E-55	PER1	17	8140472	8156506	-	7002	protein_cod	period circadian regulator 1 [Source:HGNC Symbol;Acc:HGNC:8845]
ENSG0000	515.1349	446.9917	449.1499	80.00281	64.58421	53.46317	470.4255	66.01673	2.837152867	5.87E-57	7.58E-55	KRT80	12	52168996	52192000	-	4350	protein_cod	keratin 80 [Source:HGNC Symbol;Acc:HGNC:27056]
ENSG0000	1369.93	1199.929	1301.297	377.8446	312.9151	418.7948	1290.385	369.8515	1.802610161	5.94E-57	7.63E-55	CYP4F3	19	15640897	15662825	+	5517	protein_cod	cytochrome P450 family 4 subfamily F member 3 [Source:HGNC Symbol;Acc:HGNC:2646]
ENSG0000	958.4382	1061.994	1066.028	256.3946	326.5596	278.0085	1028.82	286.9876	1.840917682	8.84E-57	1.13E-54	SKAP2	7	26667062	26995239	-	5520	protein_cod	src kinase associated phosphoprotein 2 [Source:HGNC Symbol;Acc:HGNC:15687]
ENSG0000	1833.757	1800.412	1661.517	635.2031	711.336	684.3286	1765.229	676.9559	1.382593586	1.00E-56	1.28E-54	EMP1	12	13196716	13219939	+	7838	protein_cod	epithelial membrane protein 1 [Source:HGNC Symbol;Acc:HGNC:3333]
ENSG0000	1413.029	1455.056	1216.87	464.5947	403.8787	387.608	1361.652	418.6938	1.702776317	1.14E-56	1.45E-54	TTK	6	80003887	80042527	+	4579	protein_cod	TTK protein kinase [Source:HGNC Symbol;Acc:HGNC:12401]
ENSG0000	1749.611	2044.131	1867.518	4366.419	5560.61	5072.764	1887.087	4999.931	-1.405867498	1.42E-56	1.79E-54	FAM84B	8	126552442	126558393	-	5404	protein_cod	family with sequence similarity 84 member B [Source:HGNC Symbol;Acc:HGNC:24166]
ENSG0000	1660.335	1408.387	1517.429	536.8864	567.6134	545.3244	1528.717	549.9414	1.474914565	3.11E-55	3.89E-53	UBE2T	1	202331657	202341980	-	1652	protein_cod	ubiquitin conjugating enzyme E2 T [Source:HGNC Symbol;Acc:HGNC:25009]
ENSG0000	407.3875	445.9546	556.0904	1613.551	1465.425	1743.79	469.8108	1607.589	-1.776577673	3.39E-55	4.22E-53	LINC00173	12	116533422	116536518	+	2065	processed_t	long intergenic non-protein coding RNA 173 [Source:HGNC Symbol;Acc:HGNC:33791]
ENSG0000	2988.193	3124.793	3007.841	1457.401	1258.028	1292.918	3040.276	1336.115	1.186640898	1.66E-54	2.04E-52	RANGAP1	22	41245611	41286251	-	5348	protein_cod	Ran GTPase activating protein 1 [Source:HGNC Symbol;Acc:HGNC:9854]
ENSG0000	4735.752	4568.442	4353.04	1946.093	2255.899	2044.966	4552.411	2082.319	1.128373385	1.73E-54	2.13E-52	F2RL1	5	76818933	76835315	+	2958	protein_cod	F2R like trypsin receptor 1 [Source:HGNC Symbol;Acc:HGNC:3538]
ENSG0000	1101.075	1056.809	1184.225	2565.873	2546.073	2444.158	1114.036	2518.702	-1.177356223	3.90E-54	4.75E-52	HOXB8	17	48611377	48614939	-	2051	protein_cod	homeobox B8 [Source:HGNC Symbol;Acc:HGNC:5119]
ENSG0000	718.3155	716.6387	630.3859	102.1723	130.0781	162.1716	688.4467	131.474	2.385944634	4.42E-54	5.36E-52	CENPA	2	26764289	26801067	+	1983	protein_cod	centromere protein A [Source:HGNC Symbol;Acc:HGNC:1851]
ENSG0000	1562.849	1585.732	1602.981	3142.279	3299.253	3281.748	1583.854	3241.093	-1.033241323	5.50E-54	6.63E-52	MIDN	19	1248553	1259140	+	4492	protein_cod	midnolin [Source:HGNC Symbol;Acc:HGNC:16298]
ENSG0000	1156.488	1082.736	908.4311	289.1668	306.5476	306.5222	1049.218	300.7455	1.803015359	1.18E-53	1.41E-51	CIP2A	3	108549869	108589644	-	4579	protein_cod	cell proliferation regulating inhibitor of protein phosphatase 2A [Source:HGNC Symbol;Acc:HGNC:29302]
ENSG0000	412.5184	371.2831	468.2867	1241.489	1255.299	1265.295	417.3627	1254.028	-1.588692836	1.59E-53	1.90E-51	SCG2	2	223596940	223602503	-	2719	protein_cod	secretogranin II [Source:HGNC Symbol;Acc:HGNC:10575]
ENSG0000	360.1839	314.2424	369.226	38.55557	38.20475	41.87948	347.8841	39.5466	3.13588908	3.10E-53	3.68E-51	IFI27	14	94104836	94116698	+	4147	protein_cod	interferon alpha inducible protein 27 [Source:HGNC Symbol;Acc:HGNC:5397]
ENSG0000	3985.625	4019.814	3928.655	1954.768	1665.545	1630.627	3978.031	1750.313	1.18494592	3.27E-53	3.87E-51	CEMIP2	9	71683366	71816690	-	9625	protein_cod	cell migration inducing hyaluronidase 2 [Source:HGNC Symbol;Acc:HGNC:11869]
ENSG0000	954.3335	955.1725	881.4145	282.4196	291.0838	308.3043	930.3069	293.9359	1.662022341	3.90E-53	4.59E-51	SPDL1	5	169583634	169604778	+	4777	protein_cod	spindle apparatus coiled-coil protein 1 [Source:HGNC Symbol;Acc:HGNC:26010]
ENSG0000	4164.178	4203.381	4059.235	1855.487	2049.412	2092.192	4142.265	1999.03	1.050857365	6.96E-53	8.15E-51	TAGLN2	1	159918107	159925732	-	2487	protein_cod	transgelin 2 [Source:HGNC Symbol;Acc:HGNC:11554]
ENSG0000	6635.183	6259.956	5917.747	2865.643	2579.73	2211.593	6270.963	2552.322	1.297285504	1.37E-52	1.59E-50	TUBA1C	12	49188736	49274603	+	4631	protein_cod	tubulin alpha 1c [Source:HGNC Symbol;Acc:HGNC:20768]
ENSG0000	983.0661	888.7979	861.1521	270.8529	292.9031	261.9695	911.0054	275.2418	1.727136068	2.40E-52	2.78E-50	SHCBP1	16	46580554	46621626	-	4459	protein_cod	SHC binding and spindle associated 1 [Source:HGNC Symbol;Acc:HGNC:29547]
ENSG0000	1032.322	1000.805	880.2888	309.4085	300.1801	274.4443	971.1387	294.6776	1.72171296	2.77E-52	3.20E-50	WDR62	19	36054881	36105106	+	9812	protein_cod	WD repeat domain 62 [Source:HGNC Symbol;Acc:HGNC:24502]
ENSG0000	1751.664	1828.414	1754.749	489.6558	684.9565	567.6007	1778.342	580.7377	1.613842883	7.35E-52	8.41E-50	NEMP1	12	57055643	57088063	-	5708	protein_cod	nuclear envelope integral membrane protein 1 [Source:HGNC Symbol;Acc:HGNC:29001]
ENSG0000	5144.165	5189.667	5421.319	2034.77	2551.531	2113.577	5251.717	2233.293	1.233388659	9.63E-52	1.10E-49	EIF2AK2	2	37099210	37157065	-	10753	protein_cod	eukaryotic translation initiation factor 2 alpha kinase 2 [Source:HGNC Symbol;Acc:HGNC:9437]
ENSG0000	1858.385	1754.779	1700.916	718.0975	753.1793	758.286	1771.36	743.1876	1.25302661	1.03E-51	1.16E-49	FANCD2	3	10026414	10101930	+	8378	protein_cod	FA complementation group D2 [Source:HGNC Symbol;Acc:HGNC:3585]

ENSG0000	153.9248	147.2687	128.3286	755.6892	584.8965	664.7254	143.174	668.437	-2.220835168	2.36E-51	2.66E-49	GRAMD1B	11	123358428	123627774	+	14863	protein_cod	GRAM domain containing 1B [Source:HGNC Symbol;Acc:HGNC:29214]
ENSG0000	1040.531	944.8015	937.699	220.7307	272.891	305.6311	974.3439	266.4176	1.86918429	2.66E-51	2.99E-49	CKAP2L	2	112736607	112764677	-	5219	protein_cod	cytoskeleton associated protein 2 like [Source:HGNC Symbol;Acc:HGNC:26877]
ENSG0000	791.1733	758.1228	822.8787	1863.198	1857.478	1778.542	790.7249	1833.073	-1.21334857	1.31E-50	1.47E-48	NT5E	6	85449584	85495791	+	4533	protein_cod	5'-nucleotidase ecto [Source:HGNC Symbol;Acc:HGNC:8021]
ENSG0000	1778.344	1740.26	1662.643	744.1226	614.9145	609.4802	1727.082	656.1724	1.397269289	3.57E-49	3.97E-47	STIL	1	47250139	47314147	-	7291	protein_cod	STIL, centriolar assembly protein [Source:HGNC Symbol;Acc:HGNC:10879]
ENSG0000	4286.291	4137.007	4122.273	1402.459	1879.31	1732.207	4181.857	1671.325	1.322731862	7.16E-49	7.93E-47	LBR	1	225401502	225428925	-	5257	protein_cod	lamin B receptor [Source:HGNC Symbol;Acc:HGNC:6518]
ENSG0000	1204.718	1302.602	1249.515	465.5585	498.481	499.8807	1252.278	487.9734	1.35924287	7.02E-48	7.73E-46	GPSM2	1	108875350	108934545	+	9130	protein_cod	G protein signaling modulator 2 [Source:HGNC Symbol;Acc:HGNC:29501]
ENSG0000	16045.12	16015.99	16172.77	36486.1	33455.53	29190	16077.96	33043.88	-1.039272082	1.35E-47	1.48E-45	AKAP12	6	151239999	151358557	+	8741	protein_cod	A-kinase anchoring protein 12 [Source:HGNC Symbol;Acc:HGNC:370]
ENSG0000	1730.114	1710.184	1657.014	704.6031	749.5407	627.3012	1699.104	693.815	1.292575222	2.29E-47	2.50E-45	MELK	9	36572862	36677683	+	3087	protein_cod	maternal embryonic leucine zipper kinase [Source:HGNC Symbol;Acc:HGNC:16870]
ENSG0000	3103.123	2901.816	3024.727	1556.681	1468.154	1453.307	3009.889	1492.714	1.01201344	3.83E-47	4.16E-45	RECQL4	8	144511288	144517845	-	4236	protein_cod	RecQ like helicase 4 [Source:HGNC Symbol;Acc:HGNC:9949]
ENSG0000	9169.811	8170.303	8375.127	3883.51	4527.262	4253.886	8571.747	4221.553	1.021720938	9.67E-47	1.05E-44	RHOBTB3	5	95713522	95824383	+	8833	protein_cod	Rho related BTB domain containing 3 [Source:HGNC Symbol;Acc:HGNC:18757]
ENSG0000	859.9263	813.0893	899.4256	287.239	289.2645	291.3743	857.4804	289.2926	1.567173939	2.97E-46	3.19E-44	SNTB1	8	120535745	120813273	-	6868	protein_cod	syntrophin beta 1 [Source:HGNC Symbol;Acc:HGNC:11168]
ENSG0000	327.3467	260.313	244.2745	1021.723	933.2874	896.3992	277.3114	950.4697	-1.775156008	3.66E-46	3.93E-44	GALNT5	2	157257598	157314211	+	6818	protein_cod	polypeptide N-acetylgalactosaminyltransferase 5 [Source:HGNC Symbol;Acc:HGNC:4127]
ENSG0000	476.1406	480.179	432.2646	97.35282	101.8793	102.4711	462.8614	100.5677	2.202396307	4.54E-46	4.84E-44	CENPI	X	101098218	101163681	+	4321	protein_cod	centromere protein I [Source:HGNC Symbol;Acc:HGNC:3968]
ENSG0000	1369.93	1376.237	1408.237	565.803	567.6134	610.3712	1384.801	581.2625	1.251982225	7.47E-46	7.93E-44	RCCD1	15	90954870	90963125	+	4969	protein_cod	RCC1 domain containing 1 [Source:HGNC Symbol;Acc:HGNC:30457]
ENSG0000	401.2305	350.541	423.2591	61.68892	74.59022	59.70054	391.6769	65.32656	2.583130279	1.42E-45	1.51E-43	AC099850.3	17	59202677	59203829	-	321	antisense	novel transcript, antisense to PRR11
ENSG0000	520.2657	498.8469	522.3197	129.1612	92.78296	116.7279	513.8108	112.8907	2.187711294	1.84E-45	1.94E-43	HKDC1	10	69220303	69267559	+	4259	protein_cod	hexokinase domain containing 1 [Source:HGNC Symbol;Acc:HGNC:23302]
ENSG0000	1022.06	1132.517	1017.623	356.639	404.7884	341.2732	1057.4	367.5669	1.52458238	2.10E-45	2.20E-43	MBNL3	X	132369317	132489968	-	12199	protein_cod	muscleblind like splicing regulator 3 [Source:HGNC Symbol;Acc:HGNC:20564]
ENSG0000	85.1717	71.56015	70.91841	395.1946	410.2462	425.9233	75.88342	410.4547	-2.433933633	3.72E-45	3.87E-43	AL391244.3	1	1409096	1410618	+	1523	lincRNA	novel transcript
ENSG0000	1371.983	1332.678	1598.478	3249.271	3034.548	3142.743	1434.38	3142.188	-1.132001817	7.69E-45	7.96E-43	ABCC2	10	99782640	99852594	+	7837	protein_cod	ATP binding cassette subfamily C member 2 [Source:HGNC Symbol;Acc:HGNC:53]
ENSG0000	1503.332	1599.214	1527.56	689.1809	597.6314	572.0559	1543.369	619.6227	1.317515541	8.46E-45	8.72E-43	CDCA5	11	65066300	65084164	-	3925	protein_cod	cell division cycle associated 5 [Source:HGNC Symbol;Acc:HGNC:14626]
ENSG0000	1829.652	1821.154	1765.08	860.7532	822.3117	777.8891	1805.296	820.318	1.138530254	9.17E-45	9.41E-43	ASF1B	19	14119509	14136956	-	2304	protein_cod	anti-silencing function 1B histone chaperone [Source:HGNC Symbol;Acc:HGNC:20996]
ENSG0000	225.7563	277.9438	225.1378	823.1615	784.1069	788.5818	242.946	798.6167	-1.715845256	9.83E-45	1.00E-42	C16orf45	16	15434295	15625028	+	3688	protein_cod	chromosome 16 open reading frame 45 [Source:HGNC Symbol;Acc:HGNC:19213]
ENSG0000	1349.407	1326.456	1342.947	555.2002	586.7157	518.5928	1339.603	553.5029	1.275317429	1.10E-44	1.12E-42	DDX58	9	32455705	32526324	-	4353	protein_cod	DExD/H-box helicase 58 [Source:HGNC Symbol;Acc:HGNC:19102]
ENSG0000	446.3818	490.5	450.2756	96.38893	78.22877	108.7084	462.4025	94.44205	2.29138876	2.64E-44	2.68E-42	KRT13	17	41500981	41505705	-	2913	protein_cod	keratin 13 [Source:HGNC Symbol;Acc:HGNC:6415]
ENSG0000	241.1488	212.6063	249.903	855.9337	726.7998	770.7607	234.5527	784.4981	-1.742381331	3.00E-44	3.03E-42	KCNQ1OT1	11	2608328	2699994	-	91667	antisense	KCNQ1 opposite strand/antisense transcript 1 [Source:HGNC Symbol;Acc:HGNC:6295]
ENSG0000	296.5617	339.1329	317.4443	899.3087	902.3597	906.2008	317.713	902.6231	-1.506525383	3.76E-44	3.78E-42	SKAP1	17	48133440	48430275	-	2648	protein_cod	src kinase associated phosphoprotein 1 [Source:HGNC Symbol;Acc:HGNC:15605]
ENSG0000	1596.713	1746.483	1742.567	771.1115	755.9082	714.6244	1695.254	747.2147	1.182024503	3.98E-44	3.97E-42	TRIM14	9	98069275	98119212	-	5469	protein_cod	tripartite motif containing 14 [Source:HGNC Symbol;Acc:HGNC:16283]
ENSG0000	1542.326	1407.35	1485.91	369.1696	553.9688	473.1491	1478.528	465.4292	1.666447897	3.99E-44	3.97E-42	G2E3	14	30559123	30620063	+	9353	protein_cod	G2/M-phase specific E3 ubiquitin protein ligase [Source:HGNC Symbol;Acc:HGNC:20338]
ENSG0000	2690.605	2694.395	2408.975	1245.345	1046.082	1154.805	2597.992	1148.744	1.177938762	6.66E-44	6.58E-42	WDR34	9	128633661	128656787	-	2604	protein_cod	WD repeat domain 34 [Source:HGNC Symbol;Acc:HGNC:28296]
ENSG0000	5839.905	5165.813	5496.74	2936.971	2571.543	2552.866	5500.82	2687.127	1.033860783	8.08E-44	7.95E-42	HNRNPM	19	8444767	8489114	+	5614	protein_cod	heterogeneous nuclear ribonucleoprotein M [Source:HGNC Symbol;Acc:HGNC:5046]
ENSG0000	1437.657	1495.504	1364.335	624.6003	614.9145	619.2817	1432.499	619.5988	1.209463061	1.41E-43	1.38E-41	MYBL2	20	43667019	43716496	+	2777	protein_cod	MYB proto-oncogene like 2 [Source:HGNC Symbol;Acc:HGNC:7548]
ENSG0000	832.2199	896.0576	793.6108	232.2973	286.5356	278.0085	840.6294	265.6138	1.661238429	1.64E-43	1.59E-41	RTKN2	10	62183035	62268707	-	9548	protein_cod	rhotekin 2 [Source:HGNC Symbol;Acc:HGNC:19364]
ENSG0000	889.6851	951.0241	909.5568	333.5057	296.5416	342.1643	916.7553	324.0705	1.500335158	2.04E-43	1.97E-41	GNE	9	36214441	36277056	-	5921	protein_cod	glucosamine (UDP-N-acetyl)-2-epimerase/N-acetylmannosamine kinase [Source:HGNC Symbol;Acc:HGNC:23657]
ENSG0000	3485.883	3227.467	3324.16	1769.701	1530.919	1594.094	3345.836	1631.571	1.036493473	3.54E-43	3.40E-41	SERINC2	1	31409565	31434680	+	3549	protein_cod	serine incorporator 2 [Source:HGNC Symbol;Acc:HGNC:23231]
ENSG0000	1750.638	1694.627	1765.08	764.3642	843.2333	749.3755	1736.782	785.6577	1.14429784	2.08E-42	1.98E-40	USP13	3	179652755	179789401	+	8440	protein_cod	ubiquitin specific peptidase 13 [Source:HGNC Symbol;Acc:HGNC:12611]
ENSG0000	24.62796	17.63076	19.13671	261.214	231.0478	276.2264	20.46515	256.1627	-3.643386324	2.20E-42	2.08E-40	TRIM72	16	31214021	31231537	+	8500	protein_cod	tripartite motif containing 72 [Source:HGNC Symbol;Acc:HGNC:32671]
ENSG0000	2204.203	2077.319	2099.41	1069.917	1035.167	989.9597	2126.977	1031.681	1.044184422	2.42E-42	2.28E-40	TIMELESS	12	56416373	56449403	-	5818	protein_cod	timeless circadian regulator [Source:HGNC Symbol;Acc:HGNC:11813]
ENSG0000	2810.666	2671.579	2845.742	1447.762	1300.781	1409.646	2775.996	1386.063	1.002095535	4.33E-42	4.05E-40	CDC25B	20	3786772	3806121	+	4314	protein_cod	cell division cycle 25B [Source:HGNC Symbol;Acc:HGNC:1726]
ENSG0000	130.323	117.1927	150.8423	535.9225	612.1856	512.3554	132.786	553.4878	-2.061687196	5.08E-42	4.73E-40	DEPTOR	8	119873717	120050913	+	2622	protein_cod	DEP domain containing MTOR interacting protein [Source:HGNC Symbol;Acc:HGNC:22953]
ENSG0000	1122.625	1083.773	1120.061	2387.554	2169.484	2254.364	1108.82	2270.467	-1.033879585	6.29E-42	5.83E-40	GATA2	3	128479427	128493185	-	4459	protein_cod	GATA binding protein 2 [Source:HGNC Symbol;Acc:HGNC:4171]
ENSG0000	2375.572	2193.474	2193.968	1142.209	1046.082	1038.077	2254.338	1075.456	1.068285231	8.07E-42	7.45E-40	RFWD3	16	74621394	74666881	-	6465	protein_cod	ring finger and WD repeat domain 3 [Source:HGNC Symbol;Acc:HGNC:25539]
ENSG0000	984.0923	1047.475	1077.284	417.3641	406.6077	381.3706	1036.284	401.7808	1.367115159	1.07E-41	9.86E-40	MATN2	8	97868840	98036716	+	6933	protein_cod	matrilin 2 [Source:HGNC Symbol;Acc:HGNC:6908]
ENSG0000	1513.593	1517.283	1580.467	3511.449	3151.892	2958.296	1537.115	3207.212	-1.061041095	1.28E-41	1.18E-39	EPPK1	8	143857324	143878464	-	16002	protein_cod	epiplakin 1 [Source:HGNC Symbol;Acc:HGNC:15577]
ENSG0000	321.1897	399.2849	481.7949	1290.648	1242.564	1217.178	400.7565	1250.13	-1.643257492	1.40E-41	1.27E-39	CEACAM1	19	42507304	42561234	-	5524	protein_cod	carcinoembryonic antigen related cell adhesion molecule 1 [Source:HGNC Symbol;Acc:HGNC:1814]
ENSG0000	934.8364	858.7219	910.6825	307.4807	349.3005	321.6701	901.4136	326.1504	1.466171576	1.45E-41	1.32E-39	ARHGEF39	9	35658875	35675866	-	6496	protein_cod	Rho guanine nucleotide exchange factor 39 [Source:HGNC Symbol;Acc:HGNC:25909]
ENSG0000	2134.423	2250.515	2116.295	1081.484	1049.721	954.3176	2167.078	1028.507	1.075697693	2.18E-41	1.96E-39	SORT1	1	109309568	109397951	-	9160	protein_cod	sortilin 1 [Source:HGNC Symbol;Acc:HGNC:11186]
ENSG0000	151.8724	181.4931	169.979	720.0253	710.4264	554.2349	167.7815	661.5622	-1.979210349	3.16E-41	2.82E-39	TCN1	11	59852800	59866575	-	1586	protein_cod	transcobalamin 1 [Source:HGNC Symbol;Acc:HGNC:11652]
ENSG0000	1126.729	1147.037	1168.465	473.2697	395.692	354.639	1147.41	407.8669	1.493487791	3.16E-41	2.82E-39	TYMS	18	657604	673578	+	2470	protein_cod	thymidylate synthetase [Source:HGNC Symbol;Acc:HGNC:12441]
ENSG0000	1052.845	1117.998	1018.749	391.3391	432.0775	428.5964	1063.197	417.3377	1.348802084	6.40E-41	5.67E-39	MIS18BP1	14	45203190	45253540	-	6250	protein_cod	MIS18 binding protein 1 [Source:HGNC Symbol;Acc:HGNC:20190]
ENSG0000	51.30825	72.59726	66.41565	337.3613	378.4089	388.499	63.44039	368.0897	-2.538389215	1.46E-40	1.28E-38	CAPN10-DT	2	240582700	240586699	-	4000	antisense	CAPN10 divergent transcript [Source:HGNC Symbol;Acc:HGNC:48839]
ENSG0000	86.19787	61.18912	76.54686	354.7113	544.8725	516.8107	74.64461	472.1315	-2.661854244	1.66E-40	1.46E-38	LINC-PINT	7	130938963	131110176	-	9030	processed_t	long intergenic non-protein coding RNA, p53 induced transcript [Source:HGNC Symbol;Acc:HGNC:26885]
ENSG0000	7.183155	26.9647	22.51378	287.239	253.7887	261.9695	18.88721	267.6657	-3.83175159	1.92E-40	1.69E-38	COL3A1	2	188974320	189012746	+	6811	protein_cod	collagen type III alpha 1 chain [Source:HGNC Symbol;Acc:HGNC:2201]
ENSG0000	14.36631	9.333933	7.879823	228.4418	229.2285	216.5258	10.52669	224.732	-4.406775437	2.05E-40	1.78E-38	AC068647.2	3	151797047	151808249	+	523	transcribed_	arylacetamide deacetylase (esterase) (AADAC) pseudogene
ENSG0000	494.6116	496.7727	538.0794	1175.945	1342.624	1224.307	509.8212	1247.625	-1.291939834	7.89E-40	6.76E-38	TMTC2	12	82686880	83134870	+	8323	protein_cod	transmembrane and tetratricopeptide repeat containing 2 [Source:HGNC Symbol;Acc:HGNC:25440]
ENSG0000	2382.755	2430.971	2336.931	1253.056	1067.004	1121.836	2383.552	1147.299	1.055421705	1.09E-39	9.32E-38	PSMC3	11	47418769	47426473	-	2276	protein_cod	proteasome 26S subunit, ATPase 3 [Source:HGNC Symbol;Acc:HGNC:9549]
ENSG0000	1754.742	1613.733	1730.184	806.7754	793.2033	816.2044	1699.553	805.3944	1.077293042	1.20E-39	1.02E-37	REEP4	8	22138020	22141951	-	2266	protein_cod	receptor accessory protein 4 [Source:HGNC Symbol;Acc:HGNC:26176]
ENSG0000	2356.075	2510.828	2602.593	1205.826	1265.305	1088.867	2489.832	1186.666	1.069139776	1.34E-39	1.13E-37	PARP9	3	122527924	122564577	-	8938	protein_cod	poly(ADP-ribose) polymerase family member 9 [Source:HGNC Symbol;Acc:HGNC:24118]
ENSG0000	732.6819	777.8278	748.5832	250.6112	279.2585	261.0785	753.031	263.6494	1.51367921	1.47E-39	1.24E-37	ZNF714	19	21082159	21125270	+	8033	protein_cod	zinc finger protein 714 [Source:HGNC Symbol;Acc:HGNC:27124]
ENSG0000	760.3883	686.5626	677.6648	206.2723	249.2405	205.8332	708.2053	220.4487	1.683658637	1.48E-39	1.25E-37	ESCO2	8	27771949	27812640	+	7973	protein_cod	establishment of sister chromatid cohesion N-acetyltransferase 2 [Source:HGNC Symbol;Acc:HGNC:27230]
ENSG0000	2065.67	1931.087	1892.283	988.9504	902.3597	871.4497	1963.014	920.92	1.092613436	2.70E-39	2.26E-37	FEN1	11	61792637	61797244	+	2478	protein_cod	flap structure-specific endonuclease 1 [Source:HGNC Symbol;Acc:HGNC:3650]
ENSG0000	1793.737	1903.085	1891.158	943.6476	827.7695	840.2628	1862.66	870.56	1.097791842	4.88E-39	4.04E-37	CDK5RAP2	9	120388869	120580170	-	10080	protein_cod	CDK5 regulatory subunit associated protein 2 [Source:HGNC Symbol;Acc:HGNC:18672]
ENSG0000	68.75306	53.92939	79.92392	457.8474	345.662	378.6975	67.53546	394.069	-2.547467897	8.26E-39	6.81E-37	AC139887.2	4	762387	781849	-	1575	antisense	uncharacterized LOC100129917 [Source:NCBI gene;Acc:100129917]
ENSG0000	413.5445	450.103	415.3793	105.0639	108.2468	96.23371	426.3423	103.1815	2.047476461	9.06E-39	7.45E-37	IQGAP2	5	76403249	76708132	+	7904	protein_cod	IQ motif containing GTPase activating protein 2 [Source:HGNC Symbol;Acc:HGNC:6111]
ENSG0000	1132.886	1101.404	1046.891	352.7835	418.4329	453.5459	1093.727	408.2541	1.420749789	1.40E-38	1.15E-36	DTYMK	2	241675742	241686991	-	2451	protein_cod	deoxythymidylate kinase [Source:HGNC Symbol;Acc:HGNC:3061]
ENSG0000	469.9836	456.3256	507.6858	92.53337	118.2528	130.9848	477.9983	113.9236	2.066025017	3.02E-38	2.45E-36	CDKN2C	1	50960745	50974633	+	3318	protein_cod	cyclin dependent kinase inhibitor 2C [Source:HGNC Symbol;Acc:HGNC:1789]
ENSG0000	606.4636	570.407	575.2271	189.8862	169.1924	185.339	584.0326	181.4725	1.686829302	3.60E-38	2.91E-36	POC1A	3	52075253	52154690	-	2247	protein_cod	POC1 centriolar protein A [Source:HGNC Symbol;Acc:HGNC:24488]
ENSG0000	1517.698	1611.659	1392.477	686.2892	644.9325	582.7486	1507.278	637.9901	1.24129503	4.30E-38	3.46E-36	JCAD	10	30012800	30115494	-	9515	protein_cod	junctional cadherin 5 associated [Source:HGNC Symbol;Acc:HGNC:29283]
ENSG0000	201.1284	175.2705	191.3671	637.1308	587.6254	598.7875	189.2553	607.8479	-1.683223495	7.96E-38	6.39E-36	BCL6	3	187721377	187745727	-	5938	protein_cod	B cell CLL/lymphoma 6 [Source:HGNC Symbol;Acc:HGNC:1001]
ENSG0000	2122.109	2106.358	2082.525	1031.362	1116.124	976.5939	2103.664	1041.36	1.014551278	1.02E-37	8.10E-36	HMGB3	X	150980509	150990775	+	3967	protein_cod	high mobility group box 3 [Source:HGNC Symbol;Acc:HGNC:5004]

ENSG0000	752.179	708.3418	783.4796	275.6723	251.0598	271.7711	748.0001	266.1677	1.490654271	1.17E-37	9.25E-36	DBF4B	17	44708608	44752264	+	7146	protein_cod	DBF4 zinc finger B [Source:HGNC Symbol;Acc:HGNC:17883]
ENSG0000	1111.337	1033.992	1059.273	348.9279	436.6257	430.3785	1068.201	405.3107	1.397036377	1.37E-37	1.08E-35	TRIM59	3	160432445	160485773	+	7865	protein_cod	tripartite motif containing 59 [Source:HGNC Symbol;Acc:HGNC:30834]
ENSG0000	290.4047	300.7601	245.4002	755.6892	973.3114	1001.543	278.855	910.1813	-1.70581884	1.85E-37	1.46E-35	THUMPD3-A	3	9349689	9398579	-	6275	antisense	THUMPD3 antisense RNA 1 [Source:HGNC Symbol;Acc:HGNC:44478]
ENSG0000	3652.121	3417.257	3379.319	1908.501	1642.804	1566.471	3482.899	1705.925	1.030287126	8.43E-37	6.59E-35	PSMD1	2	231056864	231172827	+	5187	protein_cod	proteasome 26S subunit, non-ATPase 1 [Source:HGNC Symbol;Acc:HGNC:9554]
ENSG0000	104.6688	127.5638	97.93495	481.9447	412.9751	507.0091	110.0558	467.3096	-2.08444945	1.35E-36	1.05E-34	RNF139-AS1	8	124462485	124474576	-	6943	lincRNA	RNF139 antisense RNA 1 (head to head) [Source:HGNC Symbol;Acc:HGNC:48940]
ENSG0000	1001.537	1018.436	1068.279	432.7863	429.3486	362.6585	1029.417	408.2645	1.334749719	1.41E-36	1.10E-34	MPHOSPH9	12	123152320	123244014	-	10304	protein_cod	M-phase phosphoprotein 9 [Source:HGNC Symbol;Acc:HGNC:7215]
ENSG0000	563.3646	501.9582	551.5876	157.114	170.1021	160.3895	538.9702	162.5352	1.729037559	1.48E-36	1.15E-34	AXIN2	17	65528563	65561647	-	5226	protein_cod	axin 2 [Source:HGNC Symbol;Acc:HGNC:904]
ENSG0000	1537.195	1606.474	1673.9	791.3531	767.7335	708.387	1605.856	755.8245	1.087390169	1.83E-36	1.41E-34	TTF2	1	117060303	117107453	+	10181	protein_cod	transcription termination factor 2 [Source:HGNC Symbol;Acc:HGNC:12398]
ENSG0000	1070.29	1168.816	982.7266	363.3863	448.4509	400.9738	1073.944	404.2703	1.409274578	2.39E-36	1.83E-34	PLK4	4	127880861	127899195	+	4482	protein_cod	polo like kinase 4 [Source:HGNC Symbol;Acc:HGNC:11397]
ENSG0000	76.96238	52.89229	66.41565	341.2168	334.7463	358.2033	65.42344	344.7221	-2.397498316	2.49E-36	1.90E-34	EGR2	10	62811996	62919900	-	3689	protein_cod	early growth response 2 [Source:HGNC Symbol;Acc:HGNC:3239]
ENSG0000	1659.309	1618.919	1706.545	815.4504	677.6794	778.7802	1661.591	757.3033	1.133869512	3.13E-36	2.38E-34	IGFBP6	12	53097436	53102345	+	1346	protein_cod	insulin like growth factor binding protein 6 [Source:HGNC Symbol;Acc:HGNC:5475]
ENSG0000	1020.008	1070.291	1136.946	2170.679	2214.056	2592.964	1075.748	2325.9	-1.113089437	3.34E-36	2.53E-34	COL4A5	X	108439844	108697545	+	11865	protein_cod	collagen type IV alpha 5 chain [Source:HGNC Symbol;Acc:HGNC:2207]
ENSG0000	105.695	93.33933	96.80926	382.6641	429.3486	412.5575	98.61453	408.19	-2.04915173	5.39E-36	4.06E-34	PKIB	6	122471917	122726373	+	3432	protein_cod	cAMP-dependent protein kinase inhibitor beta [Source:HGNC Symbol;Acc:HGNC:9018]
ENSG0000	637.2485	658.5608	598.8666	225.5501	205.5779	210.2885	631.5586	213.8055	1.563557921	6.20E-36	4.64E-34	SPC24	19	11131520	11155808	-	2839	protein_cod	SPC24, NDC80 kinetochore complex component [Source:HGNC Symbol;Acc:HGNC:26913]
ENSG0000	141.6108	129.638	168.8534	529.1752	496.6617	581.8575	146.7007	535.8982	-1.871597312	6.91E-36	5.16E-34	RAD51-AS1	15	40686243	40695107	-	2177	processed_t	RAD51 antisense RNA 1 [Source:HGNC Symbol;Acc:HGNC:48621]
ENSG0000	1068.238	1074.439	959.0871	418.328	412.9751	449.9817	1033.921	427.0949	1.275654503	9.21E-36	6.85E-34	POLQ	3	121431427	121546641	-	9400	protein_cod	DNA polymerase theta [Source:HGNC Symbol;Acc:HGNC:9186]
ENSG0000	997.4324	1026.733	989.4807	420.2557	446.6317	387.608	1004.549	418.1651	1.264725706	1.30E-35	9.60E-34	TICRR	15	89575482	89631056	+	8402	protein_cod	TOPBP1 interacting checkpoint and replication regulator [Source:HGNC Symbol;Acc:HGNC:28704]
ENSG0000	756.2837	757.0857	739.5777	279.5279	294.7223	287.8101	750.9824	287.3534	1.385811383	1.35E-35	9.95E-34	AC091057.1	15	30658717	30772993	+	5637	processed_t	OTU deubiquitinase 7A pseudogene [Source:NCBI gene;Acc:100288637]
ENSG0000	559.26	519.589	614.6262	1304.142	1235.287	1314.303	564.4917	1284.577	-1.187181025	2.89E-35	2.12E-33	KIAA0895L	16	67175602	67184040	-	4780	protein_cod	KIAA0895 like [Source:HGNC Symbol;Acc:HGNC:34408]
ENSG0000	1491.018	1372.088	1502.795	695.9281	622.1916	691.457	1455.3	669.8589	1.119405209	3.44E-35	2.51E-33	UNC13D	17	75827225	75844717	-	7752	protein_cod	unc-13 homolog D [Source:HGNC Symbol;Acc:HGNC:23147]
ENSG0000	1241.66	1446.76	1318.182	2506.112	2834.428	2770.283	1335.534	2703.608	-1.017612395	3.70E-35	2.69E-33	BTG2	1	203305491	203309602	+	2757	protein_cod	BTG anti-proliferation factor 2 [Source:HGNC Symbol;Acc:HGNC:1131]
ENSG0000	815.8012	862.8703	1035.634	331.5779	286.5356	335.0359	904.7685	317.7165	1.509247223	1.59E-34	1.15E-32	DDX60	4	168216293	168318807	-	6750	protein_cod	DExD/H-box helicase 60 [Source:HGNC Symbol;Acc:HGNC:25942]
ENSG0000	4120.053	4137.007	3935.409	2307.551	1867.484	1907.744	4064.156	2027.593	1.003673371	1.68E-34	1.21E-32	COL18A1	21	45405137	45513720	+	8525	protein_cod	collagen type XVIII alpha 1 chain [Source:HGNC Symbol;Acc:HGNC:2195]
ENSG0000	1386.349	1291.194	1483.658	648.6975	614.9145	543.5422	1387.067	602.3847	1.203644607	2.74E-34	1.97E-32	FERMT1	20	6074845	6123544	-	5359	protein_cod	fermitin family member 1 [Source:HGNC Symbol;Acc:HGNC:15889]
ENSG0000	324.2682	322.5392	314.0672	769.1837	849.6008	941.8429	320.2916	853.5424	-1.414296045	7.12E-34	5.07E-32	FGF9	13	21671383	21704498	+	4539	protein_cod	fibroblast growth factor 9 [Source:HGNC Symbol;Acc:HGNC:3687]
ENSG0000	789.1209	777.8278	708.0584	296.8779	291.0838	253.059	758.3357	280.3402	1.43686455	9.87E-34	6.97E-32	CENPN	16	81006498	81033114	+	3568	protein_cod	centromere protein N [Source:HGNC Symbol;Acc:HGNC:30873]
ENSG0000	1361.721	1428.092	1420.62	634.2392	643.1132	500.7717	1403.477	592.708	1.2442167	2.22E-33	1.56E-31	CTDSPL	3	37861960	37984469	+	5796	protein_cod	CTD small phosphatase like [Source:HGNC Symbol;Acc:HGNC:16890]
ENSG0000	1637.759	2023.389	1948.568	880.0309	859.6068	850.0644	1869.906	863.2341	1.115037072	2.23E-33	1.56E-31	STC2	5	173314713	173329503	-	6128	protein_cod	stanniocalcin 2 [Source:HGNC Symbol;Acc:HGNC:11374]
ENSG0000	227.8086	249.942	256.6571	645.8058	700.4203	675.4181	244.8026	673.8814	-1.461864882	2.91E-33	2.03E-31	SOCS2	12	93569814	93583487	+	6776	protein_cod	suppressor of cytokine signaling 2 [Source:HGNC Symbol;Acc:HGNC:19382]
ENSG0000	243.2011	183.5674	299.4333	813.5226	765.9142	960.555	242.0673	846.6639	-1.808839425	5.55E-33	3.85E-31	MYO15B	17	75588058	75626501	+	12761	protein_cod	myosin XVB [Source:HGNC Symbol;Acc:HGNC:14083]
ENSG0000	141.6108	112.0072	108.0662	440.4974	483.0171	434.8338	120.5614	452.7828	-1.907084796	5.82E-33	4.03E-31	EVA1C	21	32412006	32515397	+	4353	protein_cod	eva-1 homolog C [Source:HGNC Symbol;Acc:HGNC:13239]
ENSG0000	257.5674	273.7954	276.9195	702.6753	706.7878	695.9123	269.4274	701.7918	-1.381603703	7.84E-33	5.39E-31	DNM3	1	171841498	172418466	+	11329	protein_cod	dynamin 3 [Source:HGNC Symbol;Acc:HGNC:29125]
ENSG0000	909.1823	806.8667	749.7089	273.7446	332.9271	279.7906	821.9193	295.4874	1.476012186	8.81E-33	6.04E-31	SKA3	13	21153595	21176602	-	3048	protein_cod	spindle and kinetochore associated complex subunit 3 [Source:HGNC Symbol;Acc:HGNC:20262]
ENSG0000	882.502	1119.035	1005.24	314.2279	394.7824	384.0438	1002.259	364.3514	1.458814004	9.10E-33	6.22E-31	CNTRL	9	121074863	121177610	+	9417	protein_cod	centriolin [Source:HGNC Symbol;Acc:HGNC:1858]
ENSG0000	105.695	96.45064	83.30099	392.303	362.9451	394.7364	95.14888	383.3282	-2.007824243	1.63E-32	1.11E-30	TPTEP2	22	38335762	38398929	-	3851	transcribed_	TPTE pseudogene 2 [Source:HGNC Symbol;Acc:HGNC:53828]
ENSG0000	1753.716	1754.779	1606.358	635.2031	804.1189	808.1849	1704.951	749.169	1.185761803	1.96E-32	1.33E-30	SKA2	17	59109951	59155269	-	3148	protein_cod	spindle and kinetochore associated complex subunit 2 [Source:HGNC Symbol;Acc:HGNC:28006]
ENSG0000	1718.826	1684.256	1640.129	907.9837	805.9382	808.1849	1681.071	840.7023	1.000389704	2.27E-32	1.54E-30	ODF2	9	128455186	128501292	+	6393	protein_cod	outer dense fiber of sperm tails 2 [Source:HGNC Symbol;Acc:HGNC:8114]
ENSG0000	237.0441	301.7972	300.559	842.4393	731.348	782.3444	279.8001	785.3772	-1.489853133	3.31E-32	2.24E-30	OLFM2	19	9853718	9936552	-	2500	protein_cod	olfactomedin 2 [Source:HGNC Symbol;Acc:HGNC:17189]
ENSG0000	1236.529	1100.367	1024.377	378.8085	477.5593	461.5654	1120.424	439.3111	1.350236431	3.32E-32	2.24E-30	E2F7	12	77021247	77065580	-	6303	protein_cod	E2F transcription factor 7 [Source:HGNC Symbol;Acc:HGNC:23820]
ENSG0000	763.4668	772.6423	745.2062	312.3001	307.4572	315.4327	760.4384	311.73	1.286659728	4.79E-32	3.22E-30	ARL4D	17	43398959	43401137	+	1612	protein_cod	ADP ribosylation factor like GTPase 4D [Source:HGNC Symbol;Acc:HGNC:656]
ENSG0000	792.1994	817.2377	679.9162	293.0224	263.7947	294.9385	763.1178	283.9185	1.427226782	6.10E-32	4.08E-30	KIF15	3	44761717	44873376	+	5285	protein_cod	kinesin family member 15 [Source:HGNC Symbol;Acc:HGNC:17273]
ENSG0000	288.3524	203.2723	279.1709	865.5726	774.1009	727.9902	256.9319	789.2212	-1.619480081	7.06E-32	4.71E-30	ST3GAL1	8	133454848	133571940	-	8852	protein_cod	ST3 beta-galactoside alpha-2,3-sialyltransferase 1 [Source:HGNC Symbol;Acc:HGNC:10862]
ENSG0000	41.0466	19.70497	23.63947	230.3695	224.6803	298.5027	28.13035	251.1842	-3.154281406	7.18E-32	4.78E-30	AC025259.3	12	52058459	52059503	-	915	lincRNA	novel transcript
ENSG0000	942.0195	949.987	955.71	435.678	428.4389	397.4096	949.2388	420.5088	1.175021176	8.77E-32	5.82E-30	FANCG	9	35073835	35080016	-	3614	protein_cod	FA complementation group G [Source:HGNC Symbol;Acc:HGNC:3588]
ENSG0000	929.7056	807.9038	794.7365	279.5279	349.3005	258.4053	844.1153	295.7446	1.51333706	1.23E-31	8.14E-30	SMC2	9	104094260	104141417	+	6468	protein_cod	structural maintenance of chromosomes 2 [Source:HGNC Symbol;Acc:HGNC:14011]
ENSG0000	918.4177	1000.805	876.9118	408.6891	382.9571	401.8648	932.0449	397.837	1.228755759	1.39E-31	9.17E-30	DNMT3B	20	32762385	32809356	+	4630	protein_cod	DNA methyltransferase 3 beta [Source:HGNC Symbol;Acc:HGNC:2979]
ENSG0000	586.9664	609.817	657.4024	205.3084	233.7767	177.3195	618.0619	205.4682	1.588838629	1.49E-31	9.73E-30	KIAA1217	10	23694746	24547848	+	10807	protein_cod	KIAA1217 [Source:HGNC Symbol;Acc:HGNC:25428]
ENSG0000	1610.053	1605.437	1578.216	728.7003	751.36	858.975	1597.902	779.6784	1.034726951	1.65E-31	1.07E-29	CKS2	9	89311198	89316703	+	613	protein_cod	CDC28 protein kinase regulatory subunit 2 [Source:HGNC Symbol;Acc:HGNC:2000]
ENSG0000	1959.975	1968.423	1629.998	563.8753	804.1189	766.3055	1852.799	711.4332	1.380389134	2.00E-31	1.30E-29	ITGB8	7	20330702	20415754	+	15156	protein_cod	integrin subunit beta 8 [Source:HGNC Symbol;Acc:HGNC:6163]
ENSG0000	40.02044	65.33753	40.52481	277.6001	281.9874	302.958	48.62759	287.5152	-2.561180383	2.11E-31	1.36E-29	SESN3	11	95165513	95232541	-	10573	protein_cod	sestrin 3 [Source:HGNC Symbol;Acc:HGNC:23060]
ENSG0000	615.699	525.8116	620.2547	187.9584	170.1021	212.9616	587.2551	190.3407	1.62464166	2.67E-31	1.72E-29	SP110	2	230167293	230225729	-	6865	protein_cod	SP110 nuclear body protein [Source:HGNC Symbol;Acc:HGNC:5401]
ENSG0000	1883.013	1782.781	1875.398	842.4393	987.8656	780.5623	1847.064	870.2891	1.085635496	3.89E-31	2.48E-29	NUP107	12	68686734	68745809	+	8320	protein_cod	nucleoporin 107 [Source:HGNC Symbol;Acc:HGNC:29914]
ENSG0000	530.5273	490.55	511.0628	178.3195	166.4635	172.8643	510.7134	172.5491	1.565867624	5.53E-31	3.49E-29	ERCC6L	X	72204657	72239047	-	4364	protein_cod	ERCC excision repair 6 like, spindle assembly checkpoint helicase [Source:HGNC Symbol;Acc:HGNC:20794]
ENSG0000	2010.257	2135.397	1831.496	1063.17	931.4681	916.0023	1992.383	970.2135	1.038943336	6.48E-31	4.08E-29	SAPCD2	9	137062124	137070588	-	3848	protein_cod	suppressor APC domain containing 2 [Source:HGNC Symbol;Acc:HGNC:28055]
ENSG0000	4754.223	5796.373	5637.451	2344.179	2865.356	2769.392	5396.015	2659.642	1.020350241	8.60E-31	5.40E-29	PSAT1	9	78297143	78330093	+	2188	protein_cod	phosphoserine aminotransferase 1 [Source:HGNC Symbol;Acc:HGNC:19129]
ENSG0000	767.5715	767.4567	697.9272	215.9112	297.4512	272.6622	744.3185	262.0082	1.505238133	1.00E-30	6.27E-29	CCSAP	1	229321005	229343294	-	6263	protein_cod	centriole, cilia and spindle associated protein [Source:HGNC Symbol;Acc:HGNC:29578]
ENSG0000	599.2804	546.5536	505.4344	196.6334	185.5659	166.6269	550.4228	182.9421	1.590707751	1.76E-30	1.09E-28	PLAC8	4	83090048	83137075	-	3853	protein_cod	placenta specific 8 [Source:HGNC Symbol;Acc:HGNC:19254]
ENSG0000	7.183155	16.59366	10.1312	152.2945	198.3008	184.4479	11.30267	178.3478	-3.978945074	1.79E-30	1.11E-28	AC010864.1	10	43136824	43138334	-	1511	lincRNA	novel transcript
ENSG0000	819.9059	786.1246	739.5777	260.2501	303.8187	332.3627	781.8694	298.8105	1.38675748	1.90E-30	1.17E-28	LSM3	3	14178358	14201119	+	3815	protein_cod	LSM3 homolog, U6 small nuclear RNA and mRNA degradation associated [Source:HGNC Symbol;Acc:HGNC:17874]
ENSG0000	371.4718	393.0623	400.7453	103.1362	119.1624	105.1442	388.4265	109.1476	1.830631568	1.93E-30	1.19E-28	MTFR2	6	136231024	136250335	-	2450	protein_cod	mitochondrial fission regulator 2 [Source:HGNC Symbol;Acc:HGNC:21115]
ENSG0000	1097.997	1172.964	1108.804	439.5335	546.6917	499.8807	1126.588	495.3686	1.184710485	2.32E-30	1.42E-28	GEN1	2	17753858	17788941	+	11827	protein_cod	GEN1, Holliday junction 5' flap endonuclease [Source:HGNC Symbol;Acc:HGNC:26881]
ENSG0000	1072.342	1016.362	1055.896	491.5836	510.3063	492.7522	1048.2	498.214	1.072975377	8.80E-30	5.34E-28	SCARA3	8	27633868	27676776	+	4031	protein_cod	scavenger receptor class A member 3 [Source:HGNC Symbol;Acc:HGNC:19000]
ENSG0000	797.3303	766.4196	830.7585	345.0724	281.9874	334.1448	798.1695	320.4015	1.317047307	9.68E-30	5.84E-28	OSBPL10	3	31657890	32077580	-	11976	protein_cod	oxysterol binding protein like 10 [Source:HGNC Symbol;Acc:HGNC:16395]
ENSG0000	1608.001	1735.074	1736.938	644.842	795.0226	853.6286	1693.338	764.4977	1.146305038	1.24E-29	7.45E-28	PLSCR1	3	146515180	146544864	-	4340	protein_cod	phospholipid scramblase 1 [Source:HGNC Symbol;Acc:HGNC:9092]
ENSG0000	86.19787	96.45064	95.68357	352.7835	333.8367	379.5885	92.77736	355.4029	-1.938458584	1.40E-29	8.36E-28	AC006504.5	19	27793463	27918863	+	5141	lincRNA	uncharacterized LOC101927151 [Source:NCBI gene;Acc:101927151]
ENSG0000	101.5903	87.11671	132.8313	376.8807	469.3726	564.9275	107.1795	470.3936	-2.137755918	1.86E-29	1.11E-27	AC110285.2	17	81388126	81390256	-	1565	lincRNA	novel transcript
ENSG0000	1322.727	1294.305	1199.985	539.778	653.1192	568.4917	1272.339	587.1297	1.115626885	2.91E-29	1.72E-27	FAM102B	1	108560089	108644909	+	9355	protein_cod	family with sequence similarity 102 member B [Source:HGNC Symbol;Acc:HGNC:27637]
ENSG0000	1168.802	1157.408	1228.127	2680.576	2102.171	2679.396	1184.779	2487.381	-1.070125741	3.07E-29	1.81E-27	OBSCN	1	228208130	228378874	+	32290	protein_cod	obscurin, cytoskeletal calmodulin and titin-interacting RhoGEF [Source:HGNC Symbol;Acc:HGNC:15719]
ENSG0000	12.31398	10.37104	10.1312	167.7167	137.3552	204.9422	10.93874	170.0047	-3.955828874	5.34E-29	3.13E-27	PTOV1-AS1	19	49838639	49851676	-	4203	antisense	PTOV1 antisense RNA 1 [Source:HGNC Symbol;Acc:HGNC:44174]

ENSG0000	1010.773	1045.401	1063.776	495.4391	489.3846	410.7754	1039.983	465.1997	1.161200684	8.05E-29	4.69E-27	TENM3	4	182143987	182803024	+	11983	protein_cod	teneurin transmembrane protein 3 [Source:HGNC Symbol;Acc:HGNC:29944]
ENSG0000	1506.41	1475.799	1645.757	738.3392	662.2156	810.8581	1542.655	737.1376	1.064936223	8.56E-29	4.98E-27	ILVBL	19	15114984	15125785	-	5615	protein_cod	ilvB acetolactate synthase like [Source:HGNC Symbol;Acc:HGNC:6041]
ENSG0000	107.7473	90.22802	94.55788	442.4252	324.7403	401.8648	97.51108	389.6768	-1.997521681	1.21E-28	7.01E-27	VGF	7	101162509	101165593	-	2704	protein_cod	VGF nerve growth factor inducible [Source:HGNC Symbol;Acc:HGNC:12684]
ENSG0000	424.8323	381.6542	401.871	130.1251	88.23477	80.19476	402.7858	99.5182	2.020603041	1.81E-28	1.04E-26	LAMA3	18	23689443	23956222	+	14678	protein_cod	laminin subunit alpha 3 [Source:HGNC Symbol;Acc:HGNC:6483]
ENSG0000	926.6271	910.577	803.742	1672.348	2056.689	1800.818	880.3154	1843.285	-1.065737779	2.21E-28	1.27E-26	MPP7	10	28050993	28334486	-	6167	protein_cod	membrane palmitoylated protein 7 [Source:HGNC Symbol;Acc:HGNC:26542]
ENSG0000	1243.712	1125.258	1087.416	561.9475	484.8364	546.2154	1152.128	530.9998	1.118167864	2.61E-28	1.49E-26	COMMD4	15	75335891	75343224	+	6779	protein_cod	COMM domain containing 4 [Source:HGNC Symbol;Acc:HGNC:26027]
ENSG0000	364.2886	355.7266	361.3462	80.00281	110.9757	99.79792	360.4538	96.92548	1.892864371	3.73E-28	2.12E-26	LAMP3	3	183122213	183163839	-	3816	protein_cod	lysosomal associated membrane protein 3 [Source:HGNC Symbol;Acc:HGNC:14582]
ENSG0000	485.3761	497.8098	547.0849	192.7779	140.9937	140.7864	510.0902	158.186	1.690925239	3.99E-28	2.27E-26	CDKN2D	19	10566462	10569059	-	1503	protein_cod	cyclin dependent kinase inhibitor 2D [Source:HGNC Symbol;Acc:HGNC:1790]
ENSG0000	324.2682	353.6524	281.4223	788.4615	750.4504	819.7686	319.7809	786.2268	-1.296468562	5.75E-28	3.25E-26	MOAP1	14	93182196	93184928	-	2537	protein_cod	modulator of apoptosis 1 [Source:HGNC Symbol;Acc:HGNC:16658]
ENSG0000	234.9918	183.5674	207.1268	567.7308	587.6254	576.5112	208.562	577.2891	-1.468566282	6.25E-28	3.52E-26	MIR22HG	17	1711493	1717174	-	2986	lincRNA	MIR22 host gene [Source:HGNC Symbol;Acc:HGNC:28219]
ENSG0000	373.5241	327.7248	309.5645	747.0142	923.2814	924.0218	336.9378	864.7725	-1.359337315	6.73E-28	3.78E-26	C15orf41	15	36579611	36810248	+	9293	protein_cod	chromosome 15 open reading frame 41 [Source:HGNC Symbol;Acc:HGNC:26929]
ENSG0000	327.3467	353.6524	292.6792	75.18337	90.05404	58.80949	324.5594	74.6823	2.121103474	1.02E-27	5.68E-26	APOLD1	12	12725917	12829975	+	7243	protein_cod	apolipoprotein L domain containing 1 [Source:HGNC Symbol;Acc:HGNC:25268]
ENSG0000	96.45952	128.6009	106.9405	471.3419	348.3909	511.4643	110.6669	443.7324	-2.003203943	1.07E-27	5.97E-26	DLGAP1-AS1	18	3593732	3598352	+	2105	antisense	DLGAP1 antisense RNA 1 [Source:HGNC Symbol;Acc:HGNC:31676]
ENSG0000	1484.861	1448.834	1296.794	647.7336	730.4384	710.1691	1410.163	696.1137	1.018469322	1.13E-27	6.28E-26	NR2F2	15	96325938	96340263	+	6459	protein_cod	nuclear receptor subfamily 2 group F member 2 [Source:HGNC Symbol;Acc:HGNC:7976]
ENSG0000	400.2044	372.3202	339.9581	118.5584	85.50586	89.99634	370.8276	98.0202	1.922599093	1.14E-27	6.31E-26	METTL7A	12	50923472	50932517	+	4083	protein_cod	methyltransferase like 7A [Source:HGNC Symbol;Acc:HGNC:24550]
ENSG0000	19.49714	30.07601	12.38258	198.5612	178.2888	191.5764	20.65191	189.4755	-3.188270201	1.59E-27	8.76E-26	AC068205.2	11	43578889	43840030	+	2971	processed_t	novel transcript
ENSG0000	126.2183	156.6027	113.6946	459.7752	487.5653	405.4291	132.1719	450.9232	-1.768343805	1.69E-27	9.30E-26	DEPP1	10	44970981	44978810	-	2979	protein_cod	DEPP1, autophagy regulator [Source:HGNC Symbol;Acc:HGNC:23355]
ENSG0000	1705.486	1738.186	1739.19	648.6975	831.408	888.3797	1727.621	789.4951	1.128852854	1.73E-27	9.48E-26	C12orf75	12	105235290	105396097	+	2693	protein_cod	chromosome 12 open reading frame 75 [Source:HGNC Symbol;Acc:HGNC:35164]
ENSG0000	1560.797	1445.723	1515.177	477.1252	721.342	638.8849	1507.232	612.4507	1.298301178	1.97E-27	1.08E-25	KDELC2	11	108472105	108498432	-	4993	protein_cod	KDEL motif containing 2 [Source:HGNC Symbol;Acc:HGNC:28496]
ENSG0000	45.15126	46.66967	46.15325	240.9723	221.9514	250.3859	45.99139	237.7699	-2.370273442	2.01E-27	1.09E-25	SIK1B	21	6111134	6123739	+	4806	protein_cod	salt inducible kinase 1B (putative) [Source:HGNC Symbol;Acc:HGNC:52389]
ENSG0000	119.0351	95.41354	114.8203	540.7419	421.1558	356.4211	109.7563	439.4416	-2.001002464	2.19E-27	1.19E-25	SMPD3	16	68358325	68448688	-	7166	protein_cod	sphingomyelin phosphodiesterase 3 [Source:HGNC Symbol;Acc:HGNC:14240]
ENSG0000	324.2682	328.7619	392.8655	764.3642	979.6788	956.0997	348.6318	900.0476	-1.37007287	3.40E-27	1.84E-25	NFKBIZ	3	101827991	101861022	+	5902	protein_cod	NFKB inhibitor zeta [Source:HGNC Symbol;Acc:HGNC:29805]
ENSG0000	551.0506	668.9319	630.3859	219.7668	230.1381	246.8216	616.7895	232.2422	1.408301589	3.80E-27	2.05E-25	NEIL3	4	177309836	177362943	+	2959	protein_cod	nei like DNA glycosylase 3 [Source:HGNC Symbol;Acc:HGNC:24573]
ENSG0000	394.0474	407.5818	412.0022	865.5726	863.2453	867.8855	404.5438	865.5678	-1.097628711	5.20E-27	2.79E-25	DNASE1	16	3611728	3680143	+	13801	protein_cod	deoxyribonuclease 1 [Source:HGNC Symbol;Acc:HGNC:2956]
ENSG0000	57.46524	50.81808	60.78721	245.7918	254.6983	310.0864	56.35684	270.1922	-2.263312958	5.67E-27	3.04E-25	TIGD1	2	232547970	232550592	-	2623	protein_cod	tigger transposable element derived 1 [Source:HGNC Symbol;Acc:HGNC:14523]
ENSG0000	108.7735	124.4524	150.8423	471.3419	384.7764	483.8417	128.0228	446.6533	-1.805439658	6.01E-27	3.21E-25	CYP3A5	7	99648194	99680026	-	6759	protein_cod	cytochrome P450 family 3 subfamily A member 5 [Source:HGNC Symbol;Acc:HGNC:2638]
ENSG0000	919.4439	815.1635	843.1411	350.8557	411.1558	356.4211	859.2495	372.8109	1.20448162	6.03E-27	3.22E-25	ARHGAP19	10	97222173	97292673	-	6167	protein_cod	Rho GTPase activating protein 19 [Source:HGNC Symbol;Acc:HGNC:23724]
ENSG0000	337.6083	332.9103	267.914	73.25559	49.12039	79.3037	312.8109	67.22656	2.219278564	6.61E-27	3.51E-25	CDC25C	5	138285265	138338355	-	3338	protein_cod	cell division cycle 25C [Source:HGNC Symbol;Acc:HGNC:1727]
ENSG0000	73.88388	70.52305	92.3065	286.2751	343.8427	334.1448	78.90448	321.4209	-2.029825885	6.97E-27	3.69E-25	PCDHB14	5	141222932	141227759	+	4828	protein_cod	protocadherin beta 14 [Source:HGNC Symbol;Acc:HGNC:8685]
ENSG0000	1452.024	1784.855	1584.97	828.9448	723.1613	785.0176	1607.283	779.0412	1.045148003	7.29E-27	3.85E-25	PCK2	14	24094053	24110598	+	4189	protein_cod	phosphoenolpyruvate carboxykinase 2, mitochondrial [Source:HGNC Symbol;Acc:HGNC:8725]
ENSG0000	838.3769	873.2413	814.9989	328.6863	383.8667	395.6275	842.2057	369.3935	1.188216453	1.32E-26	6.92E-25	CCDC88A	2	55287842	55419895	-	33942	protein_cod	coiled-coil domain containing 88A [Source:HGNC Symbol;Acc:HGNC:25523]
ENSG0000	441.251	450.103	419.882	967.7449	873.2513	981.0492	437.0787	940.6818	-1.105298117	1.67E-26	8.71E-25	MAP3K12	12	53479669	53500063	-	7971	protein_cod	mitogen-activated protein kinase kinase kinase 12 [Source:HGNC Symbol;Acc:HGNC:6851]
ENSG0000	189.8405	236.4596	172.2304	536.8864	578.529	605.0249	199.5102	573.4801	-1.521914149	1.92E-26	1.00E-24	ATXN1	6	16299112	16761491	-	17812	protein_cod	ataxin 1 [Source:HGNC Symbol;Acc:HGNC:10548]
ENSG0000	1365.826	1322.307	1322.685	641.9503	562.1556	417.9038	1336.939	540.6699	1.307274607	2.95E-26	1.52E-24	RNF26	11	119334527	119337313	+	2787	protein_cod	ring finger protein 26 [Source:HGNC Symbol;Acc:HGNC:14646]
ENSG0000	1344.276	1292.231	1286.663	718.0975	644.0229	597.0054	1307.723	653.0419	1.002674131	3.19E-26	1.64E-24	AJUBA	14	22971174	22982642	-	5979	protein_cod	ajuba LIM protein [Source:HGNC Symbol;Acc:HGNC:20250]
ENSG0000	1376.087	1123.183	1224.75	586.0447	599.4507	607.6981	1241.34	597.7311	1.054318239	3.73E-26	1.92E-24	RND3	2	150468195	150539011	-	3594	protein_cod	Rho family GTPase 3 [Source:HGNC Symbol;Acc:HGNC:671]
ENSG0000	1126.729	1138.74	1010.869	502.1863	560.3363	521.2659	1092.113	527.9295	1.048866841	3.99E-26	2.05E-24	BRCA1	17	43044295	43170245	-	9187	protein_cod	BRCA1, DNA repair associated [Source:HGNC Symbol;Acc:HGNC:1100]
ENSG0000	923.5486	897.0947	1039.011	460.7391	389.3246	432.1606	953.2181	427.4081	1.157167625	4.30E-26	2.19E-24	AIFM2	10	70098223	70132934	-	5135	protein_cod	apoptosis inducing factor, mitochondria associated 2 [Source:HGNC Symbol;Acc:HGNC:21411]
ENSG0000	1119.546	983.1743	1026.628	466.5224	532.1375	474.9312	1043.116	491.197	1.086414137	4.71E-26	2.39E-24	GINS1	20	25407727	25452628	+	4068	protein_cod	GINS complex subunit 1 [Source:HGNC Symbol;Acc:HGNC:28980]
ENSG0000	437.1463	486.4016	460.4068	173.5001	162.825	152.37	461.3183	162.8984	1.502610255	5.60E-26	2.83E-24	USP18	22	18149899	18177397	+	2129	protein_cod	ubiquitin specific peptidase 18 [Source:HGNC Symbol;Acc:HGNC:12616]
ENSG0000	33.86345	33.18732	39.39912	208.2001	206.4876	198.7048	35.48329	204.4641	-2.529040774	6.17E-26	3.10E-24	AC139795.1	5	177619059	177672209	-	2230	transcribed_	SUMO interacting motifs containing 1 (SIMC1) pseudogene
ENSG0000	1198.561	1228.968	1302.422	2138.87	2540.616	2822.855	1243.317	2500.78	-1.008751566	6.66E-26	3.34E-24	HOXB5	17	48591257	48593961	-	2041	protein_cod	homeobox B5 [Source:HGNC Symbol;Acc:HGNC:5116]
ENSG0000	216.5208	255.1275	309.5645	33.73613	57.30712	49.89896	260.4043	46.98074	2.46604554	8.07E-26	4.04E-24	OAS1	12	112906777	112933222	+	4947	protein_cod	2'-5'-oligoadenylate synthetase 1 [Source:HGNC Symbol;Acc:HGNC:8086]
ENSG0000	34.88961	14.51945	19.13671	167.7167	202.849	211.1795	22.84859	193.9151	-3.080753219	1.76E-25	8.70E-24	PAN3-AS1	13	28136843	28138193	-	1351	antisense	PAN3 antisense RNA 1 [Source:HGNC Symbol;Acc:HGNC:39932]
ENSG0000	859.9263	900.206	757.5887	336.3974	394.7824	306.5222	839.2404	345.9006	1.279305531	1.80E-25	8.89E-24	RAD51AP1	12	4538798	4560048	+	2558	protein_cod	RAD51 associated protein 1 [Source:HGNC Symbol;Acc:HGNC:16956]
ENSG0000	564.3908	530.9971	507.6858	196.6334	214.6743	212.0706	534.3579	207.7928	1.362508896	2.29E-25	1.12E-23	SPATA5	4	122923074	123319450	+	8247	protein_cod	spermatogenesis associated 5 [Source:HGNC Symbol;Acc:HGNC:18119]
ENSG0000	1187.273	1294.305	1117.809	388.4474	573.0712	508.7912	1199.796	490.1033	1.290848362	2.40E-25	1.17E-23	BRIP1	17	61681266	61863521	-	7991	protein_cod	BRCA1 interacting protein C-terminal helicase 1 [Source:HGNC Symbol;Acc:HGNC:20473]
ENSG0000	829.1414	713.5273	772.2227	355.6752	326.5596	340.3822	771.6305	340.8723	1.17906928	2.42E-25	1.18E-23	ORC6	16	46689643	46698394	+	4309	protein_cod	origin recognition complex subunit 6 [Source:HGNC Symbol;Acc:HGNC:17151]
ENSG0000	1000.511	880.501	875.7861	340.2529	375.68	447.3085	918.9327	387.7472	1.244043754	2.52E-25	1.23E-23	DBF4	7	87876216	87909541	+	4583	protein_cod	DBF4 zinc finger [Source:HGNC Symbol;Acc:HGNC:17364]
ENSG0000	22.57563	20.74207	19.13671	168.6806	151.9093	178.2106	20.81814	166.2669	-2.995414237	3.11E-25	1.50E-23	LINC01311	22	19171395	19172839	+	1445	antisense	long intergenic non-protein coding RNA 1311 [Source:HGNC Symbol;Acc:HGNC:50503]
ENSG0000	384.8119	353.6524	408.6251	859.7893	789.5648	876.796	382.3631	842.05	-1.139718906	3.86E-25	1.86E-23	TMEM74B	20	1180561	1185415	-	2210	protein_cod	transmembrane protein 74B [Source:HGNC Symbol;Acc:HGNC:15893]
ENSG0000	748.0743	785.0875	737.3263	324.8307	365.674	296.7206	756.8294	329.0751	1.201861904	6.33E-25	3.02E-23	BARD1	2	214725646	214809717	-	6067	protein_cod	BRCA1 associated RING domain 1 [Source:HGNC Symbol;Acc:HGNC:952]
ENSG0000	34.88961	46.66967	37.14774	232.2973	188.2948	237.9111	39.56901	219.5011	-2.470751989	6.88E-25	3.28E-23	ZKSCAN2-DT	16	25257952	25261066	+	3115	lincRNA	ZKSCAN2 divergent transcript [Source:HGNC Symbol;Acc:HGNC:53912]
ENSG0000	356.0793	407.5818	341.0838	786.5337	975.1307	842.936	368.2483	868.2001	-1.236909882	8.27E-25	3.93E-23	KLHL24	3	183635568	183684477	+	8650	protein_cod	kelch like family member 24 [Source:HGNC Symbol;Acc:HGNC:25947]
ENSG0000	18.47097	37.33573	24.76516	161.9334	228.3188	215.6348	26.85729	201.9623	-2.910634303	9.17E-25	4.34E-23	AL031432.4	1	25232586	25234775	+	2190	bidirectiona	novel transcript
ENSG0000	299.6402	320.465	356.8434	720.9892	752.2696	736.9007	325.6496	736.7199	-1.179188239	1.09E-24	5.14E-23	ARID3A	19	925781	975934	+	7210	protein_cod	AT-rich interaction domain 3A [Source:HGNC Symbol;Acc:HGNC:3031]
ENSG0000	567.4693	683.4513	588.7354	251.5751	254.6983	231.6737	613.2187	245.9824	1.318385704	1.89E-24	8.86E-23	SORBS1	10	95311771	95561414	-	10951	protein_cod	sorbin and SH3 domain containing 1 [Source:HGNC Symbol;Acc:HGNC:14565]
ENSG0000	87.22403	71.56015	81.04961	293.9862	296.5416	292.2653	79.9446	294.2644	-1.880071387	1.90E-24	8.86E-23	FOXD2-AS1	1	47432133	47434641	-	2509	lincRNA	FOXD2 adjacent opposite strand RNA 1 [Source:HGNC Symbol;Acc:HGNC:44256]
ENSG0000	767.5715	658.5608	686.6703	318.0835	294.7223	252.168	704.2675	288.3246	1.289701856	1.98E-24	9.24E-23	ADAM19	5	157395534	157575775	-	7704	protein_cod	ADAM metallopeptidase domain 19 [Source:HGNC Symbol;Acc:HGNC:197]
ENSG0000	987.1708	837.9798	817.2503	417.3641	393.8727	366.2227	880.8003	392.4865	1.167216553	2.05E-24	9.53E-23	PKMYT1	16	2968024	2980539	-	5034	protein_cod	protein kinase, membrane associated tyrosine/threonine 1 [Source:HGNC Symbol;Acc:HGNC:29650]
ENSG0000	813.7489	788.1988	805.9934	400.978	378.4089	363.5496	802.647	380.9788	1.075577653	2.82E-24	1.30E-22	BCL2L12	19	49665142	49673916	+	2216	protein_cod	BCL2 like 12 [Source:HGNC Symbol;Acc:HGNC:13787]
ENSG0000	773.7285	649.2269	792.4851	243.864	332.0174	278.8995	738.4802	284.927	1.372767856	2.90E-24	1.33E-22	TMEM19	12	71686087	71705046	+	6488	protein_cod	transmembrane protein 19 [Source:HGNC Symbol;Acc:HGNC:25605]
ENSG0000	446.3818	480.179	478.4179	188.9223	166.4635	171.0821	468.3262	175.4893	1.416742505	2.98E-24	1.37E-22	TRAIP	3	49828599	49856574	-	3263	protein_cod	TRAF interacting protein [Source:HGNC Symbol;Acc:HGNC:30764]
ENSG0000	101.5903	103.7104	135.0827	388.4474	341.1138	417.0127	113.4611	382.1913	-1.755174387	3.84E-24	1.75E-22	LINC00482	17	81303771	81309248	+	2197	lincRNA	long intergenic non-protein coding RNA 482 [Source:HGNC Symbol;Acc:HGNC:26816]
ENSG0000	427.9108	497.8098	493.0518	916.6587	1141.594	1210.941	472.9241	1089.731	-1.205232983	4.43E-24	2.01E-22	DLGAP1-AS2	18	3603000	3608336	+	3171	antisense	DLGAP1 antisense RNA 2 [Source:HGNC Symbol;Acc:HGNC:28146]
ENSG0000	1575.163	1678.034	1564.708	923.406	743.1733	717.2976	1605.968	794.6256	1.016036254	4.44E-24	2.01E-22	MYO10	5	16661914	16936276	-	13963	protein_cod	myosin X [Source:HGNC Symbol;Acc:HGNC:7593]
ENSG0000	366.3409	318.3908	380.4829	72.29167	63.67458	115.8369	355.0716	83.93438	2.078043707	4.88E-24	2.20E-22	HERC5	4	88457117	88506163	+	4766	protein_cod	HECT and RLD domain containing E3 ubiquitin protein ligase 5 [Source:HGNC Symbol;Acc:HGNC:24368]
ENSG0000	289.3785	323.5764	366.9746	818.342	698.6011	853.6286	326.6432	790.1906	-1.2758847	5.18E-24	2.33E-22	DNAH1	3	52316319	52400491	+	13872	protein_cod	dynein axonemal heavy chain 1 [Source:HGNC Symbol;Acc:HGNC:2940]
ENSG0000	45.15126	35.26153	33.77067	206.2723	208.3068	196.0316	38.06115	203.5369	-2.415952023	5.55E-24	2.49E-22	AC004980.1	7	76549360	76627982	+	3110	transcribed_	uncharacterized LOC100133091 [Source:NCBI gene;Acc:100133091]

ENSG0000	295.5355	293.5003	324.1985	709.4225	690.4143	651.3596	304.4114	683.7322	-1.168049691	1.22E-23	5.43E-22	ZNF469	16	88427471	88440757	+	13287	protein_cod	zinc finger protein 469 [Source:HGNC Symbol;Acc:HGNC:23216]
ENSG0000	875.3188	779.902	745.2062	367.2418	381.1378	329.6896	800.1423	359.3564	1.155673182	1.33E-23	5.89E-22	PRIM2	6	57314805	57646849	+	4667	protein_cod	DNA primase subunit 2 [Source:HGNC Symbol;Acc:HGNC:9370]
ENSG0000	750.1267	791.3101	765.4686	337.3613	376.5896	371.569	768.9684	361.84	1.086980512	1.93E-23	8.46E-22	CEP97	3	101723925	101770562	+	9550	protein_cod	centrosomal protein 97 [Source:HGNC Symbol;Acc:HGNC:26244]
ENSG0000	386.8642	408.6189	437.893	134.9445	158.2768	140.7864	411.1254	144.6692	1.505752332	2.88E-23	1.25E-21	KIF24	9	34252381	34311371	-	6131	protein_cod	kinesin family member 24 [Source:HGNC Symbol;Acc:HGNC:19916]
ENSG0000	821.9582	801.6812	860.0264	352.7835	424.8004	326.1253	827.8886	367.9031	1.169886777	4.97E-23	2.12E-21	TMEM97	17	28319095	28328655	+	3034	protein_cod	transmembrane protein 97 [Source:HGNC Symbol;Acc:HGNC:28106]
ENSG0000	826.0629	848.3508	784.6053	365.3141	327.4693	417.9038	819.673	370.229	1.146483938	5.72E-23	2.43E-21	CST6	11	66011841	66013505	+	759	protein_cod	cystatin E/M [Source:HGNC Symbol;Acc:HGNC:2478]
ENSG0000	6.15699	10.37104	5.628445	116.6306	125.5299	127.4206	7.385491	123.1937	-4.053990456	5.76E-23	2.44E-21	AC023024.1	15	101295419	101305737	+	2590	antisense	novel transcript, antisense to SNRPA1 and PCSK6
ENSG0000	701.8969	747.7518	738.452	298.8057	352.0294	345.7285	729.3669	332.1879	1.133657808	6.64E-23	2.81E-21	PIGK	1	77088990	77219430	-	5513	protein_cod	phosphatidylinositol glycan anchor biosynthesis class K [Source:HGNC Symbol;Acc:HGNC:8965]
ENSG0000	942.0195	1015.325	1032.257	538.8141	474.8304	443.7443	996.5336	485.7963	1.03720913	8.27E-23	3.48E-21	RGS3	9	113444731	113597743	+	13793	protein_cod	regulator of G protein signaling 3 [Source:HGNC Symbol;Acc:HGNC:9999]
ENSG0000	321.1897	344.3184	359.0948	105.0639	121.8913	93.56055	341.5343	106.8386	1.676473422	9.74E-23	4.10E-21	ZNF703	8	37695751	37700021	+	3349	protein_cod	zinc finger protein 703 [Source:HGNC Symbol;Acc:HGNC:25883]
ENSG0000	543.8675	456.3256	595.4895	186.0306	177.3792	218.308	531.8942	193.9059	1.454483991	1.04E-22	4.35E-21	ARHGAP33	19	35774532	35788822	+	5260	protein_cod	Rho GTPase activating protein 33 [Source:HGNC Symbol;Acc:HGNC:23085]
ENSG0000	43.09893	63.26333	55.15876	226.514	227.4092	267.3159	53.84034	240.413	-2.160051643	1.12E-22	4.67E-21	PELI2	14	56117814	56301526	+	6278	protein_cod	pellino E3 ubiquitin protein ligase family member 2 [Source:HGNC Symbol;Acc:HGNC:8828]
ENSG0000	138.5323	86.07961	123.8258	409.653	351.1198	437.507	116.1459	399.4266	-1.782563793	1.79E-22	7.44E-21	SSPO	7	149776042	149833979	+	21144	protein_cod	SCO-spondin [Source:HGNC Symbol;Acc:HGNC:21998]
ENSG0000	135.4538	143.1203	91.18081	406.7613	377.4993	419.6859	123.2516	401.3155	-1.6994306	1.80E-22	7.48E-21	GNB1L	22	19783224	19854939	-	6988	protein_cod	G protein subunit beta 1 like [Source:HGNC Symbol;Acc:HGNC:4397]
ENSG0000	808.6181	985.2485	863.4035	437.6058	374.7704	413.4485	885.7567	408.6082	1.116693174	1.93E-22	7.99E-21	TNFAIP8L1	19	4639518	4655568	+	6667	protein_cod	TNF alpha induced protein 8 like 1 [Source:HGNC Symbol;Acc:HGNC:28279]
ENSG0000	974.8568	918.8739	954.5843	445.3169	454.8184	520.3749	949.4383	473.5034	1.003053409	2.91E-22	1.19E-20	GMNN	6	24774931	24786099	+	2476	protein_cod	geminin, DNA replication inhibitor [Source:HGNC Symbol;Acc:HGNC:17493]
ENSG0000	15.39248	10.37104	20.2624	175.4279	136.4455	126.5295	15.34197	146.1343	-3.258620158	2.98E-22	1.22E-20	AL359878.1	10	971146	988341	-	4964	processed_t	novel transcript, antisense to GTPBP4
ENSG0000	537.7105	510.255	508.8115	165.789	220.1321	213.8527	518.9257	199.9246	1.374626076	3.51E-22	1.42E-20	GSTCD	4	105708778	105847728	+	6295	protein_cod	glutathione S-transferase C-terminal domain containing [Source:HGNC Symbol;Acc:HGNC:25806]
ENSG0000	419.7015	362.9863	417.6306	69.40003	112.795	136.3311	400.1061	106.1754	1.91029649	3.85E-22	1.56E-20	DLEU2	13	49982552	50125720	-	5103	antisense	deleted in lymphocytic leukemia 2 [Source:HGNC Symbol;Acc:HGNC:13748]
ENSG0000	802.4611	860.7961	709.1841	296.8779	385.686	339.4911	790.8138	340.685	1.214623696	4.31E-22	1.74E-20	VRK1	14	96797304	96931722	+	2744	protein_cod	vaccinia related kinase 1 [Source:HGNC Symbol;Acc:HGNC:12718]
ENSG0000	252.4366	266.5356	335.4553	75.18337	83.68659	64.15581	284.8092	74.34192	1.937207962	4.42E-22	1.78E-20	IFIT3	10	89327894	89340971	+	2640	protein_cod	interferon induced protein with tetratricopeptide repeats 3 [Source:HGNC Symbol;Acc:HGNC:5411]
ENSG0000	700.8707	715.6015	774.4741	363.3863	339.2945	333.2538	730.3155	345.3115	1.080671812	4.69E-22	1.89E-20	CTSV	9	97029679	97039643	-	4700	protein_cod	cathepsin V [Source:HGNC Symbol;Acc:HGNC:2538]
ENSG0000	344.7915	313.2053	361.3462	117.5945	113.7046	89.99634	339.781	107.0985	1.666787635	6.08E-22	2.44E-20	ROR1	1	63774022	64181498	+	6715	protein_cod	receptor tyrosine kinase like orphan receptor 1 [Source:HGNC Symbol;Acc:HGNC:10256]
ENSG0000	1831.705	1879.232	1572.588	632.3114	924.191	841.1539	1761.175	799.2188	1.139439742	6.26E-22	2.51E-20	DHFR	5	80626228	80654983	-	4409	protein_cod	dihydrofolate reductase [Source:HGNC Symbol;Acc:HGNC:2861]
ENSG0000	298.614	337.0587	363.5976	112.7751	62.76494	60.59159	333.0901	78.71053	2.085273973	6.41E-22	2.56E-20	VTCN1	1	117143587	117210960	-	3027	protein_cod	V-set domain containing T cell activation inhibitor 1 [Source:HGNC Symbol;Acc:HGNC:28873]
ENSG0000	461.7743	534.1084	567.3473	196.6334	222.861	195.1406	521.0767	204.8783	1.345883226	6.63E-22	2.65E-20	HYLS1	11	125883614	125900648	+	2999	protein_cod	HYLS1, centriolar and ciliogenesis associated [Source:HGNC Symbol;Acc:HGNC:26558]
ENSG0000	9.235486	6.222622	5.628445	91.56949	130.9877	155.9343	7.028851	126.1638	-4.160181122	6.68E-22	2.66E-20	AGGF1P1	4	190041342	190043414	+	2073	processed_p	angiogenic factor with G-patch and FHA domains 1 pseudogene 1 [Source:HGNC Symbol;Acc:HGNC:38051]
ENSG0000	648.5363	749.826	681.0419	329.6501	304.7283	324.3432	693.1347	319.5739	1.117251125	1.14E-21	4.50E-20	SVIL	10	29457338	29736781	-	9937	protein_cod	supervillin [Source:HGNC Symbol;Acc:HGNC:11480]
ENSG0000	141.6108	113.0443	164.3506	389.4113	406.6077	446.4175	139.6686	414.1455	-1.570826871	1.51E-21	5.95E-20	GPR3	1	27392644	27395814	+	2115	protein_cod	G protein-coupled receptor 3 [Source:HGNC Symbol;Acc:HGNC:4484]
ENSG0000	2228.831	2094.949	2081.399	6405.045	10916.55	9847.916	2135.06	9056.504	-2.084687557	1.55E-21	6.10E-20	EREG	4	74365143	74388751	+	5717	protein_cod	epiregulin [Source:HGNC Symbol;Acc:HGNC:3443]
ENSG0000	46.17743	73.63436	83.30099	307.4807	289.2645	250.3859	67.70426	282.377	-2.06408885	1.76E-21	6.84E-20	RNF152	18	61808063	61894247	-	10632	protein_cod	ring finger protein 152 [Source:HGNC Symbol;Acc:HGNC:26811]
ENSG0000	572.6001	625.3735	585.3583	256.3946	279.2585	264.6427	594.444	266.7653	1.155743238	1.97E-21	7.62E-20	HERC6	4	88378739	88443111	+	5793	protein_cod	HECT and RLD domain containing E3 ubiquitin protein ligase family member 6 [Source:HGNC Symbol;Acc:HGNC:26072]
ENSG0000	424.8323	421.0641	371.4774	146.5112	120.0721	159.4985	405.7913	142.0272	1.515041602	2.04E-21	7.88E-20	PARPBP	12	102120185	102197520	+	4745	protein_cod	PARP1 binding protein [Source:HGNC Symbol;Acc:HGNC:26074]
ENSG0000	378.6549	431.4351	464.9096	856.8976	849.6008	896.3992	424.9999	867.6325	-1.030859877	4.07E-21	1.55E-19	MZF1	19	58561931	58573575	-	6691	protein_cod	myeloid zinc finger 1 [Source:HGNC Symbol;Acc:HGNC:13108]
ENSG0000	90.30253	109.933	123.8258	348.9279	322.0114	335.9269	108.0204	335.6221	-1.637940902	4.62E-21	1.76E-19	CORIN	4	47593998	47838106	-	10087	protein_cod	corin, serine peptidase [Source:HGNC Symbol;Acc:HGNC:19012]
ENSG0000	1029.244	931.3191	927.5678	468.4502	531.2279	444.6354	962.7102	481.4378	1.000005906	5.84E-21	2.21E-19	SLC30A6	2	32165841	32224379	+	5410	protein_cod	solute carrier family 30 member 6 [Source:HGNC Symbol;Acc:HGNC:19305]
ENSG0000	194.9714	225.0515	244.2745	524.3558	512.1255	581.8575	221.4325	539.4463	-1.286328729	6.35E-21	2.40E-19	CROCCP3	1	16467436	16499257	-	6813	transcribed_	ciliary rootlet coiled-coil, rootletin pseudogene 3 [Source:HGNC Symbol;Acc:HGNC:29405]
ENSG0000	1007.694	946.8757	1025.503	514.7169	536.6857	432.1606	993.3575	494.5211	1.006626963	6.40E-21	2.41E-19	DARS2	1	173824568	173858808	+	7438	protein_cod	aspartyl-tRNA synthetase 2, mitochondrial [Source:HGNC Symbol;Acc:HGNC:25538]
ENSG0000	104.6688	123.4153	121.5744	358.5668	312.0054	403.6469	116.5529	358.0731	-1.62025518	7.32E-21	2.75E-19	MHENCR	20	63627227	63628824	+	801	antisense	melanoma highly expressed competing endogenous lncRNA for miR-425 and miR-489 [Source:HGNC Symbol;Acc:HGNC:53110]
ENSG0000	2.05233	3.111311	5.628445	96.38893	130.0781	122.0742	3.597362	116.1804	-5.034572868	7.70E-21	2.88E-19	CTAGE7P	10	130106046	130108481	+	2436	processed_p	CTAGE family member 7, pseudogene [Source:HGNC Symbol;Acc:HGNC:25111]
ENSG0000	780.9116	758.1228	674.2878	371.0974	348.3909	326.1253	737.7741	348.5379	1.083086884	8.16E-21	3.05E-19	MCM8	20	5950652	5998977	+	7691	protein_cod	minichromosome maintenance 8 homologous recombination repair factor [Source:HGNC Symbol;Acc:HGNC:16147]
ENSG0000	405.3352	427.2867	513.3142	847.2587	1170.703	1144.112	448.6454	1054.024	-1.233836532	1.05E-20	3.88E-19	IL18	11	112143251	112164117	-	2628	protein_cod	interleukin 18 [Source:HGNC Symbol;Acc:HGNC:5986]
ENSG0000	617.7514	609.817	602.2437	265.0696	280.1681	300.2848	609.9373	281.8408	1.113199079	1.07E-20	3.93E-19	MICB	6	31494881	31511124	+	2798	protein_cod	MHC class I polypeptide-related sequence B [Source:HGNC Symbol;Acc:HGNC:7091]
ENSG0000	245.2535	296.6117	226.2635	555.2002	654.0289	652.2507	256.0429	620.4933	-1.276222352	1.15E-20	4.22E-19	ANGPT2	8	6499651	6563409	-	6775	protein_cod	angiopoietin 2 [Source:HGNC Symbol;Acc:HGNC:485]
ENSG0000	79.01471	123.4153	61.9129	303.6251	357.4873	386.7169	88.11432	349.2764	-1.983757061	1.47E-20	5.39E-19	EYA4	6	133240598	133532120	+	8968	protein_cod	EYA transcriptional coactivator and phosphatase 4 [Source:HGNC Symbol;Acc:HGNC:3522]
ENSG0000	422.78	443.8804	484.0463	151.3306	174.6503	193.3585	450.2356	173.6511	1.376986241	1.53E-20	5.62E-19	CCDC34	11	27330827	27363868	-	3733	protein_cod	coiled-coil domain containing 34 [Source:HGNC Symbol;Acc:HGNC:25079]
ENSG0000	501.7947	472.9193	512.1885	158.0778	206.4876	215.6348	495.6342	193.4001	1.35571964	2.02E-20	7.39E-19	MYBL1	8	66562175	66614247	-	7239	protein_cod	MYB proto-oncogene like 1 [Source:HGNC Symbol;Acc:HGNC:7547]
ENSG0000	280.1431	267.5728	315.1929	90.6056	90.96368	90.88739	287.6363	90.81889	1.662424366	2.12E-20	7.70E-19	CENPW	6	126340174	126348875	+	795	protein_cod	centromere protein W [Source:HGNC Symbol;Acc:HGNC:21488]
ENSG0000	200.1022	229.1999	269.0397	57.83336	50.93966	64.15581	232.7806	57.64294	2.012229934	2.15E-20	7.79E-19	IFI44	1	78649796	78664078	+	2038	protein_cod	interferon induced protein 44 [Source:HGNC Symbol;Acc:HGNC:16938]
ENSG0000	434.0678	346.3926	310.6902	914.731	750.4504	1059.462	363.7169	908.2144	-1.319062166	2.93E-20	1.06E-18	AC020916.1	19	13823880	13842928	-	19049	antisense	novel transcript
ENSG0000	585.9403	510.255	512.1885	129.1612	226.4996	188.0122	536.1279	181.2243	1.563116225	3.62E-20	1.30E-18	SKA1	18	50374995	50394173	+	2950	protein_cod	spindle and kinetochore associated complex subunit 1 [Source:HGNC Symbol;Acc:HGNC:28109]
ENSG0000	426.8847	372.3202	401.871	157.114	161.0057	133.6579	400.3586	150.5925	1.411433575	3.65E-20	1.31E-18	CENPM	22	41938721	41947164	-	1778	protein_cod	centromere protein M [Source:HGNC Symbol;Acc:HGNC:18352]
ENSG0000	7.183155	5.185518	10.1312	95.42504	110.0661	125.6385	7.499959	110.3765	-3.890266572	3.78E-20	1.36E-18	FLJ21408	16	27268205	27290492	+	3266	antisense	uncharacterized LOC400512 [Source:NCBI gene;Acc:400512]
ENSG0000	83.11937	72.59726	73.16979	249.6473	261.0658	264.6427	76.29547	258.4519	-1.75951559	4.25E-20	1.52E-18	AC139530.1	17	81701324	81703300	-	1977	antisense	novel transcript, antisense to HGS
ENSG0000	167.2649	213.6434	173.3561	477.1252	435.716	518.5928	184.7548	477.1447	-1.368301277	4.93E-20	1.76E-18	ZNF594	17	5179536	5191883	-	5314	protein_cod	zinc finger protein 594 [Source:HGNC Symbol;Acc:HGNC:29392]
ENSG0000	9.235486	7.259726	12.38258	114.7028	113.7046	106.9263	9.62593	111.7779	-3.545359354	5.59E-20	1.99E-18	AGGF1P2	10	133640789	133642894	+	2106	processed_p	angiogenic factor with G-patch and FHA domains 1 pseudogene 2 [Source:HGNC Symbol;Acc:HGNC:38052]
ENSG0000	675.2166	595.2975	610.1235	232.2973	298.3609	288.7011	626.8792	273.1198	1.197654254	6.37E-20	2.26E-18	HSPB11	1	53916574	53945929	-	3236	protein_cod	heat shock protein family B (small) member 11 [Source:HGNC Symbol;Acc:HGNC:25019]
ENSG0000	61.5699	38.37284	74.29548	245.7918	243.7827	241.4753	58.07941	243.6833	-2.073303418	6.78E-20	2.40E-18	HEXIM2	17	45160700	45170040	+	2038	protein_cod	hexamethylene bisacetamide inducible 2 [Source:HGNC Symbol;Acc:HGNC:28591]
ENSG0000	252.4366	228.1628	251.0287	51.08613	56.39748	80.19476	243.876	62.55946	1.959667989	6.96E-20	2.46E-18	FAM72D	1	145096000	145112696	+	2397	protein_cod	family with sequence similarity 72 member D [Source:HGNC Symbol;Acc:HGNC:33593]
ENSG0000	356.0793	383.7284	378.2315	144.5834	148.2708	132.7669	372.6797	141.8737	1.39358087	7.09E-20	2.50E-18	DDIAS	11	82899975	82958277	+	5339	protein_cod	DNA damage induced apoptosis suppressor [Source:HGNC Symbol;Acc:HGNC:26351]
ENSG0000	45.15126	60.15261	57.41014	213.0195	210.1261	225.4364	54.23781	216.194	-1.996553875	8.02E-20	2.82E-18	AC010834.3	8	93719574	93721167	+	939	antisense	novel transcript
ENSG0000	67.72689	103.7104	69.79272	335.4335	252.879	310.0864	80.41	299.4663	-1.894975863	8.11E-20	2.84E-18	PCDHB13	5	141213919	141218979	+	5061	protein_cod	protocadherin beta 13 [Source:HGNC Symbol;Acc:HGNC:8684]
ENSG0000	336.5821	394.0994	364.7233	148.439	120.0721	122.9653	365.1349	130.4921	1.485878487	8.32E-20	2.91E-18	ABCA12	2	214931542	215138428	-	9416	protein_cod	ATP binding cassette subfamily A member 12 [Source:HGNC Symbol;Acc:HGNC:14637]
ENSG0000	287.3262	253.0533	265.6626	608.2142	553.9688	605.9159	268.6807	589.3663	-1.13291901	8.64E-20	3.02E-18	TP53INP2	20	34704290	34713439	+	4228	protein_cod	tumor protein p53 inducible nuclear protein 2 [Source:HGNC Symbol;Acc:HGNC:16104]
ENSG0000	668.0335	684.4884	669.785	310.3724	315.644	354.639	674.1023	326.8851	1.043594859	1.03E-19	3.58E-18	EME1	17	50373220	50381483	+	3294	protein_cod	essential meiotic structure-specific endonuclease 1 [Source:HGNC Symbol;Acc:HGNC:24965]
ENSG0000	161.1079	144.1574	165.4763	398.0863	425.71	383.1527	156.9139	402.3163	-1.359072123	1.39E-19	4.77E-18	SSC4D	7	76389334	76409695	-	2819	protein_cod	scavenger receptor cysteine rich family member with 4 domains [Source:HGNC Symbol;Acc:HGNC:14461]
ENSG0000	296.5617	336.0216	377.1058	754.7253	665.8541	776.107	336.563	732.2288	-1.122796601	1.40E-19	4.82E-18	BCL3	19	44747705	44760044	+	3242	protein_cod	B cell CLL/lymphoma 3 [Source:HGNC Symbol;Acc:HGNC:998]
ENSG0000	440.2248	458.3998	378.2315	816.4143	862.3357	1012.236	425.6187	896.9953	-1.074899854	1.57E-19	5.35E-18	C22orf46	22	41688939	41698136	+	5341	protein_cod	chromosome 22 open reading frame 46 [Source:HGNC Symbol;Acc:HGNC:26294]
ENSG0000	153.9248	129.638	142.9625	350.8557	386.5956	463.3475	142.1751	400.2663	-1.493900029	1.60E-19	5.45E-18	TUBB3	16	89921392	89938761	+	4430	protein_cod	tubulin beta 3 class III [Source:HGNC Symbol;Acc:HGNC:20772]

ENSG0000	401.2305	464.6225	371.4774	139.764	150.0901	171.9732	412.4435	153.9424	1.421275425	2.06E-19	7.01E-18	ENC1	5	74627406	74641424	-	5858	protein_cod	ectodermal-neural cortex 1 [Source:HGNC Symbol;Acc:HGNC:3345]
ENSG0000	424.8323	546.5536	490.8004	144.5834	211.9454	130.9848	487.3955	162.5045	1.584234075	2.32E-19	7.85E-18	SCLT1	4	128864921	129093607	-	8292	protein_cod	sodium channel and clathrin linker 1 [Source:HGNC Symbol;Acc:HGNC:26406]
ENSG0000	33.86345	54.9665	46.15325	280.4918	214.6743	172.8643	44.9944	222.6768	-2.307030324	2.76E-19	9.34E-18	RBM12B-AS	8	93740121	93740773	+	653	TEC	RBM12B antisense RNA 1 [Source:HGNC Symbol;Acc:HGNC:28818]
ENSG0000	136.48	182.5302	111.4432	9.638893	20.92165	22.27632	143.4845	17.61229	3.019717204	2.97E-19	1.00E-17	EDA2R	X	66595637	66639298	-	3579	protein_cod	ectodysplasin A2 receptor [Source:HGNC Symbol;Acc:HGNC:17756]
ENSG0000	795.2779	887.7608	792.4851	347.964	422.0715	432.1606	825.1746	400.7321	1.041254308	3.30E-19	1.11E-17	BRCA2	13	32315474	32400266	+	12273	protein_cod	BRCA2, DNA repair associated [Source:HGNC Symbol;Acc:HGNC:1101]
ENSG0000	957.412	854.5734	861.1521	404.8335	494.8424	372.4601	891.0459	424.0453	1.071471878	3.35E-19	1.12E-17	IPO11	5	62403972	62628582	+	7540	protein_cod	importin 11 [Source:HGNC Symbol;Acc:HGNC:20628]
ENSG0000	23.6018	28.0018	29.26792	265.0696	165.5539	125.6385	26.95717	185.4206	-2.782441317	5.65E-19	1.86E-17	AC092053.3	3	39148281	39172952	+	4975	antisense	novel transcript, antisense CSRNP1
ENSG0000	6.15699	13.48235	10.1312	106.9917	105.5179	114.0548	9.923513	108.8548	-3.457419339	6.69E-19	2.20E-17	CASP16P	16	3144015	3149963	+	3538	transcribed_	caspase 16, pseudogene [Source:HGNC Symbol;Acc:HGNC:27290]
ENSG0000	32.83728	20.74207	25.89085	144.5834	180.1081	139.8953	26.49007	154.8623	-2.546488084	8.62E-19	2.81E-17	MRPS30-DT	5	44744900	44808777	-	2211	antisense	MRPS30 divergent transcript [Source:HGNC Symbol;Acc:HGNC:53420]
ENSG0000	637.2485	679.3029	639.3914	337.3613	318.3729	317.2148	651.9809	324.3163	1.007933418	9.13E-19	2.97E-17	IFIH1	2	162267079	162318703	-	5108	protein_cod	interferon induced with helicase C domain 1 [Source:HGNC Symbol;Acc:HGNC:18873]
ENSG0000	547.9721	548.6279	651.774	239.0446	257.4272	281.5727	582.7913	259.3482	1.166499896	9.22E-19	3.00E-17	BST2	19	17402939	17405648	-	1119	protein_cod	bone marrow stromal cell antigen 2 [Source:HGNC Symbol;Acc:HGNC:1119]
ENSG0000	746.022	683.4513	679.9162	375.9168	327.4693	293.1564	703.1298	332.1808	1.083237691	9.86E-19	3.19E-17	JADE1	4	128809623	128875224	+	9129	protein_cod	jade family PHD finger 1 [Source:HGNC Symbol;Acc:HGNC:30027]
ENSG0000	499.7424	487.4387	495.3032	238.0807	209.2165	218.308	494.1614	221.8684	1.155957837	1.07E-18	3.44E-17	CAPS	19	5911707	5915877	+	4097	protein_cod	calcyphosine [Source:HGNC Symbol;Acc:HGNC:1487]
ENSG0000	206.2592	229.1999	202.624	55.90558	64.58421	43.66159	212.6944	54.71713	1.960066803	1.13E-18	3.63E-17	C2orf78	2	73784189	73817147	+	3045	protein_cod	chromosome 2 open reading frame 78 [Source:HGNC Symbol;Acc:HGNC:34349]
ENSG0000	33.86345	51.85518	54.03308	173.5001	212.855	244.1485	46.5839	210.1679	-2.177767848	1.21E-18	3.91E-17	LINC00861	8	125922308	125951249	-	5724	lincRNA	long intergenic non-protein coding RNA 861 [Source:HGNC Symbol;Acc:HGNC:45133]
ENSG0000	101.5903	106.8217	70.91841	2.891668	5.457821	6.23737	93.11014	4.862286	4.250387479	1.25E-18	4.02E-17	TGFBI	5	136028895	136063818	+	7810	protein_cod	transforming growth factor beta induced [Source:HGNC Symbol;Acc:HGNC:11771]
ENSG0000	87.22403	98.52485	85.55237	257.3584	276.5296	347.5106	90.43375	293.7996	-1.69989595	1.26E-18	4.05E-17	ATP8A1	4	42408373	42657105	-	11199	protein_cod	ATPase phospholipid transporting 8A1 [Source:HGNC Symbol;Acc:HGNC:13531]
ENSG0000	211.39	184.6045	208.2525	495.4391	434.8064	489.188	201.4156	473.1445	-1.232315687	1.28E-18	4.08E-17	ZFYVE28	4	2269582	2418663	-	9624	protein_cod	zinc finger FYVE-type containing 28 [Source:HGNC Symbol;Acc:HGNC:29334]
ENSG0000	134.4276	99.56195	121.5744	298.8057	374.7704	383.1527	118.5213	352.2429	-1.572216211	1.49E-18	4.73E-17	GCSAM	3	112120841	112133305	-	3783	protein_cod	germinal center associated signaling and motility [Source:HGNC Symbol;Acc:HGNC:20253]
ENSG0000	214.4685	169.0479	196.9956	462.6669	496.6617	440.1801	193.504	466.5029	-1.269530345	1.55E-18	4.91E-17	ST6GAL1	3	186930485	187078553	+	9151	protein_cod	ST6 beta-galactoside alpha-2,6-sialyltransferase 1 [Source:HGNC Symbol;Acc:HGNC:10860]
ENSG0000	645.4578	536.1826	608.9978	182.1751	264.7043	266.4248	596.8794	237.7681	1.326123068	1.66E-18	5.25E-17	SPIN4	X	63347228	63351344	-	4117	protein_cod	spindlin family member 4 [Source:HGNC Symbol;Acc:HGNC:27040]
ENSG0000	183.6835	243.7194	221.7607	49.15836	46.39148	65.93791	216.3879	53.82925	2.005076455	1.96E-18	6.18E-17	FAM72B	1	121167646	121185539	-	3033	protein_cod	family with sequence similarity 72 member B [Source:HGNC Symbol;Acc:HGNC:24805]
ENSG0000	219.5993	257.2017	281.4223	559.0558	583.0772	543.5422	252.7411	561.8917	-1.154178329	2.16E-18	6.80E-17	SLC35E2A	1	1724838	1745999	-	7713	protein_cod	solute carrier family 35 member E2A [Source:HGNC Symbol;Acc:HGNC:20863]
ENSG0000	492.5592	607.7428	503.183	253.5029	180.1081	209.3974	534.495	214.9426	1.315770592	2.91E-18	9.09E-17	LOXL4	10	98247690	98268250	-	3653	protein_cod	lysyl oxidase like 4 [Source:HGNC Symbol;Acc:HGNC:17171]
ENSG0000	157.0033	130.6751	148.591	388.4474	410.2462	343.0554	145.4231	380.583	-1.38788096	3.09E-18	9.61E-17	FLRT3	20	14322988	14337616	-	5190	protein_cod	fibronectin leucine rich transmembrane protein 3 [Source:HGNC Symbol;Acc:HGNC:3762]
ENSG0000	290.4047	238.5338	303.936	620.7447	574.8905	616.6086	277.6249	604.0813	-1.122554118	3.19E-18	9.91E-17	EIF4A1	17	7572706	7579005	+	5316	protein_cod	eukaryotic translation initiation factor 4A1 [Source:HGNC Symbol;Acc:HGNC:3282]
ENSG0000	566.4431	569.3699	631.5116	291.0946	288.3549	263.7516	589.1082	281.067	1.067526976	3.47E-18	1.08E-16	RBPMS	8	30384479	30572261	+	8931	protein_cod	RNA binding protein, mRNA processing factor [Source:HGNC Symbol;Acc:HGNC:19097]
ENSG0000	102.6165	117.1927	119.323	298.8057	331.1078	310.0864	113.0441	313.3333	-1.472054216	4.97E-18	1.53E-16	NKD2	5	1008829	1038943	+	2885	protein_cod	naked cuticle homolog 2 [Source:HGNC Symbol;Acc:HGNC:17046]
ENSG0000	287.3262	290.389	305.0617	98.31671	50.93966	94.4516	294.259	81.23599	1.858012654	5.50E-18	1.69E-16	ZNF488	10	47365496	47384273	-	3496	protein_cod	zinc finger protein 488 [Source:HGNC Symbol;Acc:HGNC:23535]
ENSG0000	265.7768	238.5338	312.9416	667.0114	551.2399	806.4028	272.4174	674.8847	-1.310342575	5.70E-18	1.75E-16	AC073957.3	7	879790	886547	-	6758	antisense	novel transcript, antisense to GET4
ENSG0000	282.1954	258.2388	281.4223	634.2392	533.9568	590.768	273.9522	586.3213	-1.097715635	5.80E-18	1.77E-16	UBALD1	16	4608883	4615027	-	3619	protein_cod	UBA like domain containing 1 [Source:HGNC Symbol;Acc:HGNC:29576]
ENSG0000	65.67456	94.37644	85.55237	254.4668	261.0658	278.8995	81.86779	264.8107	-1.694949489	5.93E-18	1.81E-16	COL4A6	X	108155607	108439497	-	9282	protein_cod	collagen type IV alpha 6 chain [Source:HGNC Symbol;Acc:HGNC:2208]
ENSG0000	726.5249	598.4088	532.4509	255.4307	282.897	276.2264	619.1282	271.518	1.189622126	6.26E-18	1.91E-16	ZWILCH	15	66504959	66550128	+	4849	protein_cod	zwilch kinetochore protein [Source:HGNC Symbol;Acc:HGNC:25468]
ENSG0000	667.0073	812.0522	864.5292	410.6168	322.9211	315.4327	781.1962	349.6569	1.160327846	6.60E-18	2.01E-16	KIF21B	1	200969390	201023700	-	12150	protein_cod	kinesin family member 21B [Source:HGNC Symbol;Acc:HGNC:29442]
ENSG0000	189.8405	185.6416	228.5149	453.028	543.9628	455.328	201.3323	484.1063	-1.267636832	7.23E-18	2.19E-16	PLA2G4A	1	186828953	186988981	+	2875	protein_cod	phospholipase A2 group IVA [Source:HGNC Symbol;Acc:HGNC:9035]
ENSG0000	114.9305	123.4153	139.5854	324.8307	322.9211	376.0243	125.9771	341.2587	-1.439798655	8.01E-18	2.42E-16	EIF3J-DT	15	44527257	44536923	-	3330	lincRNA	EIF3J divergent transcript [Source:HGNC Symbol;Acc:HGNC:48616]
ENSG0000	9.235486	25.92759	12.38258	116.6306	128.2588	140.7864	15.84855	128.5586	-3.016806185	1.01E-17	3.04E-16	AC006277.1	19	2949035	2950644	+	1610	TEC	TEC
ENSG0000	41.0466	33.18732	43.90187	189.8862	159.1864	170.1911	39.3786	173.0879	-2.137658222	1.15E-17	3.46E-16	AL354822.1	GL00021	51867	54893	-	3027	protein_coding	
ENSG0000	75.93622	56.0036	36.02205	213.9834	223.7707	281.5727	55.98729	239.7756	-2.093101668	1.19E-17	3.57E-16	SIK1	21	43414483	43427189	-	5268	protein_cod	salt inducible kinase 1 [Source:HGNC Symbol;Acc:HGNC:11142]
ENSG0000	564.3908	489.5129	554.9647	264.1057	246.5116	231.6737	536.2895	247.4303	1.116506696	1.22E-17	3.64E-16	GINS4	8	41529206	41545046	+	5475	protein_cod	GINS complex subunit 4 [Source:HGNC Symbol;Acc:HGNC:28226]
ENSG0000	56.43908	51.85518	67.54134	216.8751	211.0357	203.1601	58.61187	210.357	-1.846156303	1.35E-17	4.02E-16	NTN5	19	48661407	48673081	-	3115	protein_cod	netrin 5 [Source:HGNC Symbol;Acc:HGNC:25208]
ENSG0000	246.2796	266.5356	252.1544	78.07053	80.04804	93.56055	254.9899	83.89454	1.602675268	1.63E-17	4.86E-16	EFCAB11	14	89794669	89954777	-	8302	protein_cod	EF-hand calcium binding domain 11 [Source:HGNC Symbol;Acc:HGNC:20357]
ENSG0000	35.91578	43.55836	33.77067	182.1751	150.0901	174.6464	37.74827	168.9705	-2.160100819	1.82E-17	5.39E-16	AL162458.1	20	45926518	45935055	-	8538	antisense	novel transcript
ENSG0000	953.3073	901.2431	1046.891	415.4363	561.2459	457.1101	967.1471	477.9308	1.015974572	1.94E-17	5.74E-16	TAP2	6	32821833	32838780	-	6390	protein_cod	transporter 2, ATP binding cassette subfamily B member [Source:HGNC Symbol;Acc:HGNC:44]
ENSG0000	707.0277	601.5201	576.3528	306.5168	304.7283	293.1564	628.3002	301.4672	1.060272096	1.97E-17	5.81E-16	POLA1	X	24693919	24996986	+	6336	protein_cod	DNA polymerase alpha 1, catalytic subunit [Source:HGNC Symbol;Acc:HGNC:9173]
ENSG0000	454.5911	618.1138	581.9812	249.6473	216.4936	240.5843	551.5621	235.5751	1.227273859	2.91E-17	8.52E-16	CHAC1	15	40952962	40956519	+	2058	protein_cod	ChaC glutathione specific gamma-glutamylcyclotransferase 1 [Source:HGNC Symbol;Acc:HGNC:28680]
ENSG0000	50.28209	79.85698	63.03859	240.9723	214.6743	233.4559	64.39255	229.7008	-1.83472855	3.18E-17	9.28E-16	CEBPD	8	47736909	47739086	-	2178	protein_cod	CCAAT enhancer binding protein delta [Source:HGNC Symbol;Acc:HGNC:1835]
ENSG0000	97.48568	89.19092	92.3065	362.4224	225.5899	343.9464	92.99437	310.6529	-1.739506972	4.43E-17	1.28E-15	AC124944.3	3	195913078	195954485	-	5485	transcribed_	programmed cell death 6 (PDCD6) pseudogene
ENSG0000	493.5854	504.0324	473.9151	181.2112	236.5056	231.6737	490.511	216.4635	1.178936196	7.04E-17	2.00E-15	SASS6	1	100083563	100132955	-	3972	protein_cod	SAS-6 centriolar assembly protein [Source:HGNC Symbol;Acc:HGNC:25403]
ENSG0000	282.1954	293.5003	278.0452	106.0278	101.8793	108.7084	284.5803	105.5385	1.431178397	7.60E-17	2.15E-15	PLCH1	3	155375580	155745067	-	7173	protein_cod	phospholipase C eta 1 [Source:HGNC Symbol;Acc:HGNC:29185]
ENSG0000	15.39248	9.333933	7.879823	91.56949	113.7046	101.58	10.86874	102.2847	-3.226525345	8.81E-17	2.48E-15	AL033527.3	1	39788976	39790171	+	1196	antisense	uncharacterized LOC101929536 [Source:NCBI gene;Acc:101929536]
ENSG0000	258.5936	283.1293	305.0617	53.01391	99.15041	92.6695	282.2615	81.61127	1.786339868	9.49E-17	2.67E-15	FAM72A	1	206186179	206204414	-	3783	protein_cod	family with sequence similarity 72 member A [Source:HGNC Symbol;Acc:HGNC:24044]
ENSG0000	455.6173	403.4333	466.0353	180.2473	212.855	184.4479	441.6953	192.5168	1.197304609	9.87E-17	2.77E-15	ZNF888	19	52904417	52923481	-	4444	protein_cod	zinc finger protein 888 [Source:HGNC Symbol;Acc:HGNC:38695]
ENSG0000	647.5102	609.817	575.2271	229.4057	319.2825	288.7011	610.8514	279.1298	1.129005555	1.24E-16	3.47E-15	C4orf46	4	158666679	158672255	-	3730	protein_cod	chromosome 4 open reading frame 46 [Source:HGNC Symbol;Acc:HGNC:27320]
ENSG0000	289.3785	285.2035	321.9471	533.9947	826.8599	798.3834	298.843	719.746	-1.269408405	1.40E-16	3.89E-15	CD24	6	106969831	106975627	-	3097	protein_cod	CD24 molecule [Source:HGNC Symbol;Acc:HGNC:1645]
ENSG0000	459.722	424.1754	462.6582	226.514	159.1864	171.9732	448.8519	185.8912	1.273446872	2.25E-16	6.23E-15	CRYBG2	1	26321698	26360080	-	6895	protein_cod	crystallin beta-gamma domain containing 2 [Source:HGNC Symbol;Acc:HGNC:17295]
ENSG0000	295.5355	271.7212	288.1764	544.5975	563.9748	608.5891	285.1444	572.3871	-1.005697571	2.89E-16	7.94E-15	RDH13	19	55039108	55071291	-	8594	protein_cod	retinol dehydrogenase 13 [Source:HGNC Symbol;Acc:HGNC:19978]
ENSG0000	9.235486	15.55656	9.005513	101.2084	97.33114	98.01581	11.26585	98.85178	-3.128798623	3.10E-16	8.52E-15	AL133367.1	14	103525010	103529072	-	4063	antisense	novel transcript, antisense to TRMT61A
ENSG0000	527.4488	555.8876	486.2977	263.1418	242.873	254.8411	523.2114	253.6186	1.045548709	3.11E-16	8.55E-15	DCTN3	9	34613545	34620523	-	3740	protein_cod	dynactin subunit 3 [Source:HGNC Symbol;Acc:HGNC:2713]
ENSG0000	124.166	121.3411	118.1974	319.0474	302.9091	303.849	121.2348	308.6018	-1.347312811	3.29E-16	9.00E-15	SHANK1	19	50661827	50719450	-	7567	protein_cod	SH3 and multiple ankyrin repeat domains 1 [Source:HGNC Symbol;Acc:HGNC:15474]
ENSG0000	233.9656	203.2723	236.3947	51.08613	63.67458	81.08581	224.5442	65.28217	1.77887823	3.53E-16	9.65E-15	DMBX1	1	46506996	46514226	+	2896	protein_cod	diencephalon/mesencephalon homeobox 1 [Source:HGNC Symbol;Acc:HGNC:19026]
ENSG0000	79.01471	93.33933	109.1918	256.3946	323.8307	263.7516	93.84863	281.3256	-1.586730933	3.70E-16	1.01E-14	FRMD3	9	83242990	83538546	-	6486	protein_cod	FERM domain containing 3 [Source:HGNC Symbol;Acc:HGNC:24125]
ENSG0000	291.4309	321.5021	357.9691	580.2614	756.8178	733.3365	323.634	690.1386	-1.094198612	3.72E-16	1.01E-14	WNT16	7	121325367	121341104	+	3261	protein_cod	Wnt family member 16 [Source:HGNC Symbol;Acc:HGNC:16267]
ENSG0000	90.30253	115.1185	73.16979	255.4307	300.1801	292.2653	92.86361	282.6254	-1.60318521	4.23E-16	1.14E-14	ACP7	19	39083913	39111493	+	3107	protein_cod	acid phosphatase 7, tartrate resistant (putative) [Source:HGNC Symbol;Acc:HGNC:33781]
ENSG0000	185.7359	191.8642	160.9735	405.7974	418.4329	400.0827	179.5245	408.1044	-1.183276512	4.33E-16	1.17E-14	ERV3-1	7	64990809	65006743	-	4574	protein_cod	endogenous retrovirus group 3 member 1, envelope [Source:HGNC Symbol;Acc:HGNC:3454]
ENSG0000	24.62796	40.44704	52.90739	204.3445	150.0901	194.2495	39.32746	182.8947	-2.223575287	4.84E-16	1.30E-14	AC025048.2	17	60126535	60135644	-	1566	processed_t	uncharacterized LOC100996660 [Source:NCBI gene;Acc:100996660]
ENSG0000	849.6647	763.3083	747.4575	249.6473	422.9811	340.3822	786.8102	337.6702	1.219416098	4.90E-16	1.32E-14	RFC3	13	33818049	33966558	+	2874	protein_cod	replication factor C subunit 3 [Source:HGNC Symbol;Acc:HGNC:9971]
ENSG0000	119.0351	124.4524	119.323	298.8057	294.7223	343.9464	120.9369	312.4915	-1.369765398	4.99E-16	1.34E-14	MST1	3	49683947	49689501	-	5084	protein_cod	macrophage stimulating 1 [Source:HGNC Symbol;Acc:HGNC:7380]
ENSG0000	18.47097	16.59366	9.005513	101.2084	106.4275	118.51	14.69005	108.7153	-2.878394952	5.13E-16	1.38E-14	AC114811.2	4	98658904	98664550	+	5647	antisense	novel transcript, antisense TSPAN5

ENSG0000	114.9305	110.9701	91.18081	338.3252	312.0054	255.7322	105.6938	302.0209	-1.512003474	5.30E-16	1.42E-14	SERPING1	11	57597387	57614853	+	3603	protein_cod	serpin family G member 1 [Source:HGNC Symbol;Acc:HGNC:1228]
ENSG0000	146.7416	155.5656	115.946	328.6863	352.0294	391.1722	139.4177	357.296	-1.355852783	6.16E-16	1.64E-14	ZNF324	19	58467045	58475436	+	5664	protein_cod	zinc finger protein 324 [Source:HGNC Symbol;Acc:HGNC:14096]
ENSG0000	261.6721	277.9438	246.5259	99.2806	96.4215	89.10529	262.0473	94.9358	1.465949498	6.34E-16	1.69E-14	ACAT2	6	159760328	159779055	+	4299	protein_cod	acetyl-CoA acetyltransferase 2 [Source:HGNC Symbol;Acc:HGNC:94]
ENSG0000	5.130825	6.222622	10.1312	73.25559	97.33114	93.56055	7.16155	88.04909	-3.633038395	6.55E-16	1.74E-14	AC015802.4	17	76545668	76557683	-	1494	antisense	novel transcript, antisense to PRCD
ENSG0000	538.7367	436.6207	556.0904	224.5862	246.5116	228.1095	510.4826	233.0691	1.13037224	6.95E-16	1.84E-14	UBXN11	1	26281328	26318363	-	4704	protein_cod	UBX domain protein 11 [Source:HGNC Symbol;Acc:HGNC:30600]
ENSG0000	368.3933	408.6189	373.7288	177.3556	168.2828	158.6074	383.5803	168.082	1.191194549	8.22E-16	2.16E-14	SRPX2	X	100644166	100675788	+	7548	protein_cod	sushi repeat containing protein X-linked 2 [Source:HGNC Symbol;Acc:HGNC:30668]
ENSG0000	24.62796	32.15021	30.3936	171.5723	129.1684	129.2027	29.05726	143.3145	-2.30253881	9.02E-16	2.37E-14	LINC01145	1	145164099	145216058	-	6056	transcribed_	long intergenic non-protein coding RNA 1145 [Source:HGNC Symbol;Acc:HGNC:49462]
ENSG0000	110.8258	118.2298	108.0662	14.45834	20.92165	21.38527	112.3739	18.92175	2.566352077	9.40E-16	2.46E-14	SYT8	11	1828307	1837521	+	4704	protein_cod	synaptotagmin 8 [Source:HGNC Symbol;Acc:HGNC:19264]
ENSG0000	84.14554	94.37644	79.92392	246.7557	246.5116	245.9306	86.14863	246.3993	-1.514965946	1.13E-15	2.93E-14	FLJ20021	4	101347780	101348883	+	790	lincRNA	uncharacterized LOC90024 [Source:NCBI gene;Acc:90024]
ENSG0000	149.8201	147.2687	185.7387	343.1446	443.9028	409.8843	160.9425	398.9772	-1.312315647	1.13E-15	2.93E-14	CASC19	8	127072694	127227541	-	22208	processed_t	cancer susceptibility 19 [Source:HGNC Symbol;Acc:HGNC:49476]
ENSG0000	11.28782	3.111311	16.88534	106.9917	84.59622	123.8563	10.42815	105.1481	-3.348283294	1.15E-15	2.99E-14	AC008105.2	17	45245186	45251129	-	1649	transcribed_	novel pseudogene, ortholog of 1700023F06Rik (M. musculus)
ENSG0000	163.1602	159.714	158.7222	371.0974	347.4813	393.8454	160.5321	370.808	-1.207722365	1.25E-15	3.22E-14	GTF2IRD2B	7	75092573	75149817	+	5672	protein_cod	GTF2I repeat domain containing 2B [Source:HGNC Symbol;Acc:HGNC:33125]
ENSG0000	320.1635	336.0216	401.871	124.3417	158.2768	104.2532	352.6854	128.9572	1.450895244	1.40E-15	3.61E-14	NUSAP1	15	41332694	41381050	+	2875	protein_cod	nucleolar and spindle associated protein 1 [Source:HGNC Symbol;Acc:HGNC:18538]
ENSG0000	122.1136	142.0832	150.8423	339.289	339.2945	331.4717	138.3464	336.6851	-1.284567209	1.52E-15	3.88E-14	AC074117.1	2	27356246	27367622	+	3412	antisense	uncharacterized LOC105374363 [Source:NCBI gene;Acc:105374363]
ENSG0000	796.3041	714.5644	682.1676	271.8168	397.5113	360.8764	731.012	343.4015	1.089141812	1.77E-15	4.48E-14	IKBIP	12	98613405	98645113	-	4163	protein_cod	IKBKB interacting protein [Source:HGNC Symbol;Acc:HGNC:26430]
ENSG0000	21.54947	29.0389	37.14774	136.8723	143.7226	139.0042	29.24537	139.8664	-2.263828018	1.80E-15	4.55E-14	AC023908.3	15	39782571	39785617	+	3047	antisense	uncharacterized LOC105370941 [Source:NCBI gene;Acc:105370941]
ENSG0000	14.36631	12.44524	10.1312	78.07503	100.06	133.6579	12.31425	103.931	-3.073894354	1.94E-15	4.89E-14	AC003102.1	17	44221401	44223710	+	2012	antisense	novel transcript, antisense to UBTF
ENSG0000	121.0875	129.638	120.4487	353.7474	309.2765	286.028	123.7247	316.3506	-1.353365255	2.08E-15	5.25E-14	MKRN3	15	23565674	23630075	+	5735	protein_cod	makorin ring finger protein 3 [Source:HGNC Symbol;Acc:HGNC:7114]
ENSG0000	2164.182	2405.043	2340.308	4668.116	7004.203	6698.935	2303.178	6123.752	-1.410859855	2.22E-15	5.57E-14	PCDH7	4	30720415	31146805	+	12249	protein_cod	protocadherin 7 [Source:HGNC Symbol;Acc:HGNC:8659]
ENSG0000	505.8994	635.7446	607.8721	235.189	311.0958	254.8411	583.172	267.042	1.125893572	2.51E-15	6.27E-14	NCEH1	3	172630249	172711218	-	4808	protein_cod	neutral cholesterol ester hydrolase 1 [Source:HGNC Symbol;Acc:HGNC:29260]
ENSG0000	438.1725	495.7356	488.5491	242.9001	224.6803	200.4869	474.1524	222.6891	1.091034636	2.83E-15	7.03E-14	SLC2A8	9	127397138	127408424	+	3928	protein_cod	solute carrier family 2 member 8 [Source:HGNC Symbol;Acc:HGNC:13812]
ENSG0000	116.9828	141.0461	142.9625	353.7474	322.0114	314.5417	133.6638	330.1002	-1.305098699	3.15E-15	7.79E-14	LINC00894	X	149938628	150224580	+	6323	antisense	long intergenic non-protein coding RNA 894 [Source:HGNC Symbol;Acc:HGNC:48579]
ENSG0000	79.01471	95.41354	75.42117	239.0446	239.2345	243.2574	83.28314	240.5122	-1.528662069	3.39E-15	8.36E-14	AC092902.2	3	125827238	125916384	-	6732	processed_t	uncharacterized LOC101927056 [Source:NCBI gene;Acc:101927056]
ENSG0000	76.96238	56.0036	78.79823	274.7085	181.9274	297.6117	70.58807	251.4158	-1.833883381	3.75E-15	9.24E-14	AC099489.1	16	11359845	11527245	-	12027	protein_cod	vitellogenin [Source:NCBI gene;Acc:400499]
ENSG0000	401.2305	435.5836	434.516	195.6695	192.843	208.5064	423.7767	199.0063	1.089948539	3.83E-15	9.42E-14	APOL2	22	36226203	36239954	-	3484	protein_cod	apolipoprotein L2 [Source:HGNC Symbol;Acc:HGNC:619]
ENSG0000	939.9672	934.4304	767.7199	287.239	471.1919	417.0127	880.7059	391.8145	1.167760195	4.40E-15	1.08E-13	RFC5	12	118013588	118033130	+	3249	protein_cod	replication factor C subunit 5 [Source:HGNC Symbol;Acc:HGNC:9973]
ENSG0000	654.6933	575.5925	475.0408	939.7921	1387.196	1296.482	568.4422	1207.823	-1.086778893	5.38E-15	1.31E-13	CTGF	6	131948176	131951373	-	2339	protein_cod	connective tissue growth factor [Source:HGNC Symbol;Acc:HGNC:2500]
ENSG0000	28.73262	25.92759	28.14223	127.2334	136.4455	123.8563	27.60081	129.1784	-2.226877857	5.75E-15	1.39E-13	LINC01011	6	2987967	2991173	+	2811	lincRNA	long intergenic non-protein coding RNA 1011 [Source:HGNC Symbol;Acc:HGNC:33812]
ENSG0000	291.4309	284.1664	202.624	83.85837	74.59022	95.34266	259.4071	84.59708	1.617453042	5.75E-15	1.39E-13	CEP128	14	80476983	80959517	-	9400	protein_cod	centrosomal protein 128 [Source:HGNC Symbol;Acc:HGNC:20359]
ENSG0000	372.4979	384.7655	378.2315	142.6556	188.2948	159.4985	378.4983	163.483	1.210135476	5.80E-15	1.40E-13	FBXO5	6	152970519	152983579	-	3833	protein_cod	F-box protein 5 [Source:HGNC Symbol;Acc:HGNC:13584]
ENSG0000	504.8732	525.8116	543.7078	200.489	270.1621	265.5338	524.7975	245.395	1.094929457	6.24E-15	1.51E-13	PRIM1	12	56731596	56752373	-	2437	protein_cod	DNA primase subunit 1 [Source:HGNC Symbol;Acc:HGNC:9369]
ENSG0000	17.44481	13.48235	5.628445	78.07503	116.4335	118.51	12.1852	104.3395	-3.085856767	6.67E-15	1.61E-13	AC016737.1	2	173968351	173969418	+	1068	lincRNA	novel transcript
ENSG0000	428.937	412.7673	414.2536	213.0195	192.843	183.5569	418.6526	196.4731	1.092451101	6.87E-15	1.65E-13	PPIH	1	42658425	42676758	+	1444	protein_cod	peptidylprolyl isomerase H [Source:HGNC Symbol;Acc:HGNC:14651]
ENSG0000	489.4807	460.474	491.9261	117.5945	210.1261	213.8527	480.627	180.5244	1.410287185	7.39E-15	1.77E-13	RBM27	5	146203600	146289132	+	6578	protein_cod	RNA binding motif protein 27 [Source:HGNC Symbol;Acc:HGNC:29243]
ENSG0000	422.78	367.1347	360.2205	183.139	153.7286	167.5179	383.3784	168.1285	1.1904875	7.64E-15	1.83E-13	SMAD7	18	48919853	48952052	-	5033	protein_cod	SMAD family member 7 [Source:HGNC Symbol;Acc:HGNC:6773]
ENSG0000	613.6467	636.7817	551.5876	226.514	318.3729	302.0669	600.672	282.3179	1.088337862	8.82E-15	2.09E-13	C1orf109	1	37681570	37692249	-	4434	protein_cod	chromosome 1 open reading frame 109 [Source:HGNC Symbol;Acc:HGNC:26039]
ENSG0000	6.15699	3.111311	13.50827	87.71393	72.77095	105.1442	7.59219	88.54304	-3.564673889	9.34E-15	2.21E-13	AC023024.2	15	101299656	101301648	-	1993	TEC	TEC
ENSG0000	111.852	131.7122	110.3175	285.3112	301.9994	300.2848	117.9606	295.8652	-1.325971441	9.49E-15	2.24E-13	TRAF1	9	120902393	120929173	-	6449	protein_cod	TNF receptor associated factor 1 [Source:HGNC Symbol;Acc:HGNC:12031]
ENSG0000	489.4807	472.9193	445.7729	202.4168	215.5839	251.2769	469.391	223.0925	1.072464465	1.02E-14	2.42E-13	CENPU	4	184694618	184734133	-	2953	protein_cod	centromere protein U [Source:HGNC Symbol;Acc:HGNC:21348]
ENSG0000	169.3172	154.5285	149.7166	45.3028	31.83729	45.4437	157.8541	40.86126	1.951021214	1.09E-14	2.55E-13	ALPG	2	232406843	232410714	+	2493	protein_cod	alkaline phosphatase, germ cell [Source:HGNC Symbol;Acc:HGNC:441]
ENSG0000	475.1144	439.732	493.0518	241.9362	222.861	228.1095	469.2994	230.9689	1.022863566	1.23E-14	2.89E-13	KLK8	19	50996007	51002711	-	1865	protein_cod	kallikrein related peptidase 8 [Source:HGNC Symbol;Acc:HGNC:6369]
ENSG0000	257.5674	288.3148	289.3021	560.0197	506.6677	649.5775	278.3948	572.0883	-1.039881971	1.26E-14	2.94E-13	DNM1	9	128191655	128255248	+	10775	protein_cod	dynamin 1 [Source:HGNC Symbol;Acc:HGNC:2972]
ENSG0000	163.1602	186.6787	180.1103	387.4835	392.9631	376.9154	176.6497	385.7873	-1.127258445	1.34E-14	3.13E-13	AF117829.1	8	89609409	89757727	-	7481	lincRNA	uncharacterized LOC101929709 [Source:NCBI gene;Acc:101929709]
ENSG0000	161.1079	141.0461	157.5965	45.3028	40.93366	30.2958	153.2502	38.84408	1.982851519	1.40E-14	3.27E-13	DRD1	5	175440039	175444208	-	4054	protein_cod	dopamine receptor D1 [Source:HGNC Symbol;Acc:HGNC:3020]
ENSG0000	273.9861	316.3166	428.8875	761.4726	685.8662	732.4455	339.7301	726.5947	-1.098930203	1.51E-14	3.52E-13	AL365181.2	1	156637783	156641004	-	3222	lincRNA	novel transcript
ENSG0000	84.14554	89.19092	84.42668	220.7307	237.4152	256.6232	85.92104	238.2564	-1.4716861	1.87E-14	4.34E-13	LINC01910	18	70380140	70384591	-	2637	lincRNA	long intergenic non-protein coding RNA 1910 [Source:HGNC Symbol;Acc:HGNC:52729]
ENSG0000	343.7653	273.7954	293.8048	133.9806	113.7046	93.56055	303.7885	113.7486	1.419543586	1.88E-14	4.35E-13	ERFE	2	238158982	238168900	+	3572	protein_cod	erythroferrone [Source:HGNC Symbol;Acc:HGNC:26727]
ENSG0000	172.3957	208.4578	185.7387	54.94169	65.49385	53.46317	188.8641	57.96624	1.703835498	1.98E-14	4.57E-13	APOBEC3B	22	38982347	38992804	+	1896	protein_cod	apolipoprotein B mRNA editing enzyme catalytic subunit 3B [Source:HGNC Symbol;Acc:HGNC:17352]
ENSG0000	525.3965	481.2161	581.8056	180.2473	278.3489	235.238	529.1561	231.278	1.192218663	2.12E-14	4.89E-13	CCDC18	1	93179919	93278730	+	5633	protein_cod	coiled-coil domain containing 18 [Source:HGNC Symbol;Acc:HGNC:30370]
ENSG0000	36.94194	32.15021	37.14774	185.0667	119.1624	155.0432	35.4133	153.0908	-2.111986347	2.24E-14	5.15E-13	TBILA	3	112133423	112135359	+	1937	antisense	TGF-beta induced lncRNA [Source:HGNC Symbol;Acc:HGNC:53943]
ENSG0000	17.44481	16.59366	27.01654	116.6306	106.4275	114.9458	20.35167	112.668	-2.476047255	2.29E-14	5.26E-13	HLA-DRA	6	32439842	32445046	+	1280	protein_cod	major histocompatibility complex, class II, DR alpha [Source:HGNC Symbol;Acc:HGNC:4947]
ENSG0000	66.70073	49.78098	46.15325	203.3806	209.2165	161.2806	54.21165	191.2926	-1.815777265	2.47E-14	5.64E-13	AC005332.4	17	68205489	68207493	+	2005	lincRNA	novel transcript
ENSG0000	327.3467	326.6877	308.4388	140.7278	127.3492	146.1327	320.8244	138.0699	1.216697301	2.69E-14	6.15E-13	MSX2	5	174724533	174730893	+	2270	protein_cod	msh homeobox 2 [Source:HGNC Symbol;Acc:HGNC:7392]
ENSG0000	0	4.148415	6.754134	66.50836	103.6986	65.04686	3.634183	78.41794	-4.461518724	3.10E-14	7.05E-13	AC090152.1	8	59119040	59121346	+	1743	antisense	uncharacterized LOC100505501 [Source:NCBI gene;Acc:100505501]
ENSG0000	176.5004	173.1963	218.3837	384.5918	417.5233	466.9117	189.3601	423.0089	-1.162003028	3.43E-14	7.77E-13	CDKN1C	11	2883213	2885773	-	2478	protein_cod	cyclin dependent kinase inhibitor 1C [Source:HGNC Symbol;Acc:HGNC:1786]
ENSG0000	116.9828	104.7475	122.7001	312.3001	332.9271	251.2769	114.8101	298.8347	-1.380580303	3.63E-14	8.19E-13	NATD1	17	21238870	21253410	-	4931	protein_cod	N-acetyltransferase domain containing 1 [Source:HGNC Symbol;Acc:HGNC:30770]
ENSG0000	66.70073	50.81808	47.27894	189.8862	172.831	191.5764	54.93258	184.7645	-1.74709904	3.87E-14	8.73E-13	LINC01410	9	62801461	62813486	+	3038	lincRNA	long intergenic non-protein coding RNA 1410 [Source:HGNC Symbol;Acc:HGNC:50702]
ENSG0000	35.91578	61.18912	25.89085	163.8612	167.3732	200.4869	40.99858	177.2404	-2.107032718	4.16E-14	9.35E-13	AP003469.4	8	101166805	101169629	-	2825	lincRNA	novel transcript
ENSG0000	252.4366	205.3465	257.7828	485.8002	504.8484	473.1491	238.522	487.9326	-1.033409303	4.52E-14	1.01E-12	KAZALD1	10	101061841	101068131	+	3613	protein_cod	Kazal type serine peptidase inhibitor domain 1 [Source:HGNC Symbol;Acc:HGNC:25460]
ENSG0000	3.078495	8.29683	6.754134	64.58058	75.49986	90.88739	6.043153	76.98928	-3.677102325	4.57E-14	1.02E-12	AL358876.1	1	8189824	8192770	+	1075	processed_p	52kDa eukaryotic translation initiation factor 2 subunit 3 gamma (EIF2S3) pseudogene
ENSG0000	75.93622	65.33753	112.5689	292.0585	221.0417	277.1174	84.61422	263.4059	-1.642420612	5.78E-14	1.28E-12	AC009120.2	16	74305127	74335346	-	3297	processed_t	novel transcript, antisense to PSMD7
ENSG0000	440.2248	410.6931	443.5215	218.8029	205.5779	214.7437	431.4798	213.0415	1.018163735	6.09E-14	1.35E-12	TCTN2	12	123671113	123708403	+	5106	protein_cod	tectonic family member 2 [Source:HGNC Symbol;Acc:HGNC:25774]
ENSG0000	407.3875	417.9528	425.5105	210.1279	207.3972	164.8448	416.9503	194.1233	1.103817936	6.21E-14	1.37E-12	SERPINA1	14	94376747	94390693	-	4902	protein_cod	serpin family A member 1 [Source:HGNC Symbol;Acc:HGNC:8941]
ENSG0000	105.695	113.0443	115.946	23.13334	26.37947	16.93	111.5618	22.14761	2.334030246	6.52E-14	1.44E-12	CLIC5	6	45880827	46080348	-	9395	protein_cod	chloride intracellular channel 5 [Source:HGNC Symbol;Acc:HGNC:13517]
ENSG0000	212.4162	232.3112	196.9956	411.5807	443.9028	490.0791	213.9077	448.5209	-1.067733525	6.98E-14	1.53E-12	ZBTB26	9	122915566	122931500	-	4645	protein_cod	zinc finger and BTB domain containing 26 [Source:HGNC Symbol;Acc:HGNC:23383]
ENSG0000	19.49714	20.74207	11.25689	99.2806	105.5179	104.2532	17.16537	103.0172	-2.577666276	7.06E-14	1.55E-12	LRP1B	2	140231423	142131701	-	17040	protein_cod	LDL receptor related protein 1B [Source:HGNC Symbol;Acc:HGNC:6693]
ENSG0000	508.9779	523.7374	414.2536	180.2473	244.6923	228.1095	482.3229	217.683	1.147338472	7.26E-14	1.59E-12	MTBP	8	120445400	120542133	+	8326	protein_cod	MDM2 binding protein [Source:HGNC Symbol;Acc:HGNC:7417]
ENSG0000	293.4832	345.3555	273.5424	136.8723	120.0721	108.7084	304.1271	121.8843	1.321203422	8.04E-14	1.76E-12	CYTOR	2	87454781	87636740	+	17693	lincRNA	cytoskeleton regulator RNA [Source:HGNC Symbol;Acc:HGNC:28717]
ENSG0000	4.10466	2.074207	3.377067	83.85837	53.66857	76.63055	3.185312	71.38583	-4.486261136	8.07E-14	1.76E-12	DUX4L50	9	63817748	63818462	-	715	unprocessed	double homeobox 4 like 50 (pseudogene) [Source:HGNC Symbol;Acc:HGNC:51788]

ENSG0000	242.175	199.1239	193.6185	63.6167	82.77695	60.59159	211.6391	68.99508	1.617389835	9.40E-14	2.04E-12	CAVIN3	11	6318946	6320647	-	1672	protein_cod	caveolae associated protein 3 [Source:HGNC Symbol;Acc:HGNC:9400]
ENSG0000	381.7334	266.5356	332.0783	139.764	121.8913	128.3116	326.7824	129.989	1.330607231	9.56E-14	2.08E-12	HASPIN	17	3723903	3726699	+	2797	protein_cod	histone H3 associated protein kinase [Source:HGNC Symbol;Acc:HGNC:19682]
ENSG0000	105.695	82.9683	94.55788	11.56667	12.73492	19.60316	94.40706	14.63492	2.684745201	1.00E-13	2.17E-12	EPSTI1	13	42886388	42992271	-	4924	protein_cod	epithelial stromal interaction 1 [Source:HGNC Symbol;Acc:HGNC:16465]
ENSG0000	15.39248	11.40814	12.38258	109.8834	75.49986	93.56055	13.06107	92.98126	-2.829221067	1.02E-13	2.20E-12	BMS1P10	9	63299453	63385117	-	1596	transcribed_	BMS1, ribosome biogenesis factor pseudogene 10 [Source:HGNC Symbol;Acc:HGNC:49154]
ENSG0000	340.6868	375.4315	332.0783	142.6556	167.3732	161.2806	349.3989	157.1031	1.152631464	1.28E-13	2.74E-12	MIS18A	21	32268219	32279069	-	1723	protein_cod	MIS18 kinetochore protein A [Source:HGNC Symbol;Acc:HGNC:1286]
ENSG0000	130.323	130.6751	157.5965	315.1918	310.1862	345.7285	139.5315	323.7022	-1.216208632	1.53E-13	3.26E-12	LINC02593	1	916865	921016	-	4152	lincRNA	long intergenic non-protein coding RNA 2593 [Source:HGNC Symbol;Acc:HGNC:53933]
ENSG0000	255.5151	215.7176	290.4278	566.7669	503.9388	492.7522	253.8868	521.1526	-1.038652583	1.61E-13	3.41E-12	PLEKHA4	19	48837097	48868632	-	3851	protein_cod	pleckstrin homology domain containing A4 [Source:HGNC Symbol;Acc:HGNC:14339]
ENSG0000	17.44481	21.77918	13.50827	89.64171	125.5299	98.01581	17.57742	104.3958	-2.565922236	1.62E-13	3.43E-12	AC110285.7	17	81395609	81397144	-	1536	TEC	TEC
ENSG0000	536.6843	469.808	415.3793	146.5112	231.0478	219.199	473.9572	198.9193	1.251449507	1.98E-13	4.17E-12	PCLAF	15	64364311	64387687	-	3734	protein_cod	PCNA clamp associated factor [Source:HGNC Symbol;Acc:HGNC:28961]
ENSG0000	31.81112	36.29863	55.15876	167.7167	141.9033	162.1716	41.0895	157.2639	-1.942386022	2.68E-13	5.60E-12	AC009237.3	2	95525345	95532405	+	4978	transcribed_	Ankyrin repeat domain-containing protein pseudogene
ENSG0000	9.235486	16.59366	15.75965	85.78615	94.60223	90.88739	13.86293	90.42526	-2.709795286	3.95E-13	8.13E-12	NR4A3	9	99821855	99866891	+	6314	protein_cod	nuclear receptor subfamily 4 group A member 3 [Source:HGNC Symbol;Acc:HGNC:7982]
ENSG0000	17.44481	26.9647	16.88534	104.1	100.06	117.619	20.43161	107.2597	-2.389342454	4.06E-13	8.37E-12	AL024497.1	6	133435077	133439464	-	631	antisense	novel transcript, antisense to EYA4
ENSG0000	368.3933	323.5764	330.9526	158.0778	165.5539	139.0042	340.9741	154.212	1.145556774	4.28E-13	8.78E-12	VASN	16	4371848	4383528	+	2806	protein_cod	vasorin [Source:HGNC Symbol;Acc:HGNC:18517]
ENSG0000	141.6108	155.5656	118.1974	26.9889	37.29511	40.09738	138.4579	34.7938	1.990342794	4.47E-13	9.16E-12	NOSTRIN	2	168786539	168865514	+	5248	protein_cod	nitric oxide synthase trafficking [Source:HGNC Symbol;Acc:HGNC:20203]
ENSG0000	42.07277	42.52125	63.03859	172.5362	186.4755	152.37	49.21087	170.4606	-1.797073141	4.54E-13	9.30E-12	GPR68	14	91232532	91253925	-	3278	protein_cod	G protein-coupled receptor 68 [Source:HGNC Symbol;Acc:HGNC:4519]
ENSG0000	385.8381	436.6207	380.4829	188.9223	207.3972	187.1211	400.9805	194.4802	1.044131869	4.94E-13	1.01E-11	CCDC77	12	389273	442645	+	3614	protein_cod	coiled-coil domain containing 77 [Source:HGNC Symbol;Acc:HGNC:28203]
ENSG0000	22.57563	33.18732	29.26792	151.3306	96.4215	147.9148	28.34362	131.889	-2.218880238	5.18E-13	1.06E-11	AC245060.6	22	22264601	22273020	+	8420	lincRNA	novel transcript
ENSG0000	232.9395	202.2352	216.1323	429.8946	411.1558	474.0401	217.1023	438.3635	-1.01380141	5.20E-13	1.06E-11	ZNF789	7	99472841	99503650	+	6683	protein_cod	zinc finger protein 789 [Source:HGNC Symbol;Acc:HGNC:27801]
ENSG0000	166.2387	146.2316	175.6075	353.7474	338.3849	359.9854	162.6926	350.7059	-1.109155117	6.26E-13	1.27E-11	ADAMTS10	19	8580242	8610735	-	5703	protein_cod	ADAM metallopeptidase with thrombospondin type 1 motif 10 [Source:HGNC Symbol;Acc:HGNC:13201]
ENSG0000	103.6427	90.22802	77.67255	240.9723	251.9694	224.5453	90.51441	239.1623	-1.399254477	6.28E-13	1.27E-11	SMIM4	3	52534013	52579237	+	2482	protein_cod	small integral membrane protein 4 [Source:HGNC Symbol;Acc:HGNC:37257]
ENSG0000	178.5527	193.9384	191.3671	356.639	392.9631	436.6159	187.9528	395.406	-1.07388543	6.49E-13	1.31E-11	HOXA-AS2	7	27107777	27134302	+	5281	antisense	HOXA cluster antisense RNA 2 [Source:HGNC Symbol;Acc:HGNC:43745]
ENSG0000	28.73262	11.40814	14.63396	95.42504	125.5299	105.1442	18.25824	108.6997	-2.568809519	7.14E-13	1.44E-11	AC233280.2	3	195650146	195651472	-	1070	processed_p	protein phosphatase 4, regulatory subunit 2, pseudogene.
ENSG0000	182.6574	136.8977	200.3727	359.5307	387.5053	454.437	173.3092	400.491	-1.21056172	7.40E-13	1.49E-11	COL11A2	6	33162681	33192499	-	6740	protein_cod	collagen type XI alpha 2 chain [Source:HGNC Symbol;Acc:HGNC:2187]
ENSG0000	210.3638	190.8271	190.2415	80.00281	58.21676	65.93791	197.1441	68.05249	1.536746038	8.46E-13	1.69E-11	GRIN1	9	137138390	137168762	+	6729	protein_cod	glutamate ionotropic receptor NMDA type subunit 1 [Source:HGNC Symbol;Acc:HGNC:4584]
ENSG0000	198.0499	203.2723	202.624	80.00281	60.03603	74.84844	201.3154	71.62909	1.49180633	9.63E-13	1.91E-11	CALB2	16	71358713	71390438	+	1703	protein_cod	calbindin 2 [Source:HGNC Symbol;Acc:HGNC:1435]
ENSG0000	279.1169	306.9827	317.4443	133.0167	112.795	143.4595	301.1813	129.7571	1.214249861	1.03E-12	2.04E-11	HOXB2	17	48540894	48544989	-	2336	protein_cod	homeobox B2 [Source:HGNC Symbol;Acc:HGNC:5113]
ENSG0000	98.51185	95.41354	127.2029	274.7085	241.0538	296.7206	107.0428	270.8276	-1.341961477	1.30E-12	2.56E-11	LINC01089	12	121795267	121803906	-	4132	lincRNA	long intergenic non-protein coding RNA 1089 [Source:HGNC Symbol;Acc:HGNC:27886]
ENSG0000	9.235486	17.63076	12.38258	80.00281	80.95768	102.4711	13.08294	87.81052	-2.746963586	1.30E-12	2.57E-11	HRAT92	7	520391	525232	+	3506	lincRNA	heart tissue-associated transcript 92 [Source:NCBI gene;Acc:441307]
ENSG0000	143.6631	144.1574	106.9405	321.939	327.4693	285.1369	131.587	311.5151	-1.240434169	1.32E-12	2.59E-11	VWA5A	11	124115362	124147721	+	6403	protein_cod	von Willebrand factor A domain containing 5A [Source:HGNC Symbol;Acc:HGNC:6658]
ENSG0000	149.8201	127.5638	135.0827	39.51946	40.02402	36.53317	137.4888	38.69222	1.829908255	1.37E-12	2.68E-11	AC015849.5	17	35893707	35911023	-	5749	lincRNA	novel transcript
ENSG0000	133.4015	104.7475	113.6946	268.9251	270.1621	298.5027	117.2812	279.1967	-1.250920472	1.78E-12	3.45E-11	SAMD11	1	923928	944581	+	4166	protein_cod	sterile alpha motif domain containing 11 [Source:HGNC Symbol;Acc:HGNC:28706]
ENSG0000	176.5004	165.9366	195.8699	344.1085	390.2342	391.1722	179.4356	375.1716	-1.065750622	1.82E-12	3.53E-11	LRP2BP	4	185363879	185395899	-	7360	protein_cod	LRP2 binding protein [Source:HGNC Symbol;Acc:HGNC:25434]
ENSG0000	518.2134	518.5518	433.3903	215.9112	231.9574	278.8995	490.0518	242.256	1.016059486	1.84E-12	3.56E-11	COX17	3	119654513	119677454	-	2172	protein_cod	cytochrome c oxidase copper chaperone COX17 [Source:HGNC Symbol;Acc:HGNC:2264]
ENSG0000	381.7334	371.2831	298.3076	105.0639	179.1985	119.4011	350.4414	134.5545	1.380564494	1.87E-12	3.62E-11	CEP57L1	6	109095110	109163932	+	6432	protein_cod	centrosomal protein 57 like 1 [Source:HGNC Symbol;Acc:HGNC:21561]
ENSG0000	102.6165	91.26512	111.4432	270.8529	275.62	222.7632	101.7749	256.412	-1.333948732	1.96E-12	3.77E-11	DICER1-AS1	14	95157645	95179933	+	3722	antisense	DICER1 antisense RNA 1 [Source:HGNC Symbol;Acc:HGNC:43017]
ENSG0000	315.0327	294.5374	300.559	142.6556	104.6082	142.5685	303.3764	129.9441	1.22384901	2.34E-12	4.48E-11	SUV39H1	X	48695554	48709012	+	3368	protein_cod	suppressor of variegation 3-9 homolog 1 [Source:HGNC Symbol;Acc:HGNC:11479]
ENSG0000	55.41291	59.11491	48.40463	155.1862	158.2768	220.9811	54.31082	178.148	-1.712916221	2.64E-12	5.03E-11	DOCK4	7	111726110	112206411	-	11499	protein_cod	dedicator of cytokinesis 4 [Source:HGNC Symbol;Acc:HGNC:19192]
ENSG0000	260.6459	254.0904	256.6571	123.3778	100.9697	86.43213	257.1311	103.5932	1.313834115	2.77E-12	5.27E-11	STARD13	13	33103135	33350630	-	8388	protein_cod	StAR related lipid transfer domain containing 13 [Source:HGNC Symbol;Acc:HGNC:19164]
ENSG0000	100.5642	138.9719	108.0662	266.0335	281.0778	293.1564	115.8674	280.0892	-1.273022116	2.78E-12	5.27E-11	ARC	8	142611044	142614472	-	2991	protein_cod	activity regulated cytoskeleton associated protein [Source:HGNC Symbol;Acc:HGNC:648]
ENSG0000	14.36631	18.66787	16.88534	105.0639	69.1324	122.9653	16.63984	99.05388	-2.574309803	2.79E-12	5.29E-11	C9orf50	9	129612225	129620776	-	1620	protein_cod	chromosome 9 open reading frame 50 [Source:HGNC Symbol;Acc:HGNC:23677]
ENSG0000	199.076	217.7918	225.1378	435.678	386.5956	545.3244	214.0019	455.866	-1.092011878	2.84E-12	5.38E-11	CACNA1A	19	13206442	13633025	-	43159	protein_cod	calcium voltage-gated channel subunit alpha1 A [Source:HGNC Symbol;Acc:HGNC:1388]
ENSG0000	69.77922	57.0407	92.3065	187.9584	237.4152	217.4169	73.04214	214.2635	-1.556887094	3.01E-12	5.69E-11	CCDC28B	1	32200386	32205387	+	2818	protein_cod	coiled-coil domain containing 28B [Source:HGNC Symbol;Acc:HGNC:28163]
ENSG0000	393.0212	339.1329	363.5976	157.114	134.6262	196.0316	365.2506	162.5906	1.166950817	3.14E-12	5.90E-11	HIRIP3	16	29992321	29996436	-	3931	protein_cod	HIRA interacting protein 3 [Source:HGNC Symbol;Acc:HGNC:4917]
ENSG0000	45.15126	66.37464	45.02756	155.1862	172.831	180.8837	52.18449	169.6336	-1.699281941	3.14E-12	5.90E-11	AP000873.2	11	83184491	83193794	-	2986	processed_t	uncharacterized LOC100506282 [Source:NCBI gene;Acc:100506282]
ENSG0000	10.26165	18.66787	9.005513	80.00281	87.32513	85.54107	12.64501	84.28967	-2.731160476	3.54E-12	6.64E-11	LINC01138	1	148290889	148519604	-	7495	processed_t	long intergenic non-protein coding RNA 1138 [Source:HGNC Symbol;Acc:HGNC:49454]
ENSG0000	5.130825	7.259726	18.01103	48.19447	109.1564	147.0237	10.13386	101.4582	-3.341555949	3.55E-12	6.65E-11	AL596247.1	10	74005137	74027915	+	442	sense_intro	novel transcript
ENSG0000	11.28782	13.48235	11.25689	66.50836	97.33114	81.08581	12.00902	81.64177	-2.764711273	4.17E-12	7.76E-11	LINC00623	1	120913275	121009291	+	2075	lincRNA	long intergenic non-protein coding RNA 623 [Source:HGNC Symbol;Acc:HGNC:44252]
ENSG0000	322.2158	291.4261	300.559	148.439	139.1744	133.6579	304.7337	140.4238	1.118599994	4.20E-12	7.81E-11	ABCG2	4	88090264	88231322	-	5044	protein_cod	ATP binding cassette subfamily G member 2 (Junior blood group) [Source:HGNC Symbol;Acc:HGNC:74]
ENSG0000	66.70073	70.52305	64.16428	210.1279	162.825	206.7243	67.12935	193.2257	-1.524311417	4.29E-12	7.96E-11	CYTH4	22	37282027	37315345	+	6161	protein_cod	cytohesin 4 [Source:HGNC Symbol;Acc:HGNC:9505]
ENSG0000	10.26165	15.55656	12.38258	76.14726	84.59622	83.75897	12.7336	81.50082	-2.67845649	4.48E-12	8.28E-11	AC004477.1	17	48045141	48048073	-	2933	processed_t	novel transcript, antisense to NFE2L1
ENSG0000	70.80539	71.56015	88.92944	187.9584	217.4032	219.199	77.09833	208.1869	-1.436138358	5.49E-12	1.00E-10	ZNF436-AS1	1	23368997	23371839	+	2655	antisense	ZNF436 antisense RNA 1 [Source:HGNC Symbol;Acc:HGNC:25122]
ENSG0000	230.8871	222.9773	186.8644	471.3419	402.9691	414.3396	213.5763	429.5502	-1.006013966	5.82E-12	1.06E-10	MMP17	12	131828393	131851783	+	4003	protein_cod	matrix metallopeptidase 17 [Source:HGNC Symbol;Acc:HGNC:7163]
ENSG0000	108.7735	123.4153	113.6946	263.1418	280.1681	254.8411	115.2945	266.0503	-1.206260269	5.89E-12	1.07E-10	GCNT3	15	59594875	59640239	+	6475	protein_cod	glucosaminyl (N-acetyl) transferase 3, mucin type [Source:HGNC Symbol;Acc:HGNC:4205]
ENSG0000	282.1954	285.2035	294.9305	130.1251	138.2648	124.7474	287.4431	131.0458	1.133064159	5.89E-12	1.07E-10	LIN52	14	74084796	74201235	+	3692	protein_cod	lin-52 DREAM MuvB core complex component [Source:HGNC Symbol;Acc:HGNC:19856]
ENSG0000	31.81112	26.9647	37.14774	90.6056	154.6383	169.3	31.97452	138.1813	-2.115580759	8.59E-12	1.55E-10	AL355388.2	1	155978799	155982986	+	2157	antisense	uncharacterized LOC107985209 [Source:NCBI gene;Acc:107985209]
ENSG0000	213.4423	209.4949	244.2745	101.2084	76.40949	81.97686	222.4039	86.53158	1.362886925	1.10E-11	1.95E-10	OASL	12	121019111	121039242	-	3517	protein_cod	2'-5'-oligoadenylate synthetase like [Source:HGNC Symbol;Acc:HGNC:8090]
ENSG0000	6.15699	6.222622	6.754134	62.65281	55.48785	72.17528	6.377916	63.43864	-3.31612553	1.29E-11	2.28E-10	AC011921.1	17	60526293	60550798	+	718	antisense	novel transcript, antisense to APPBP2
ENSG0000	36.94194	38.37284	33.77067	112.7751	129.1684	155.0432	36.36182	132.3289	-1.863280551	1.40E-11	2.46E-10	AL353622.1	1	28239509	28241453	-	1945	antisense	novel transcript
ENSG0000	13.34015	18.66787	21.38809	92.53337	88.23477	91.77844	17.7987	90.84886	-2.356522303	1.59E-11	2.78E-10	TMC1	9	72521608	72838230	+	7004	protein_cod	transmembrane channel like 1 [Source:HGNC Symbol;Acc:HGNC:16513]
ENSG0000	193.9452	220.9031	201.4983	341.2168	587.6254	490.0791	205.4489	472.9738	-1.203627152	1.59E-11	2.78E-10	MTHFD2L	4	74114174	74303099	+	9819	protein_cod	methylenetetrahydrofolate dehydrogenase (NADP+ dependent) 2 like [Source:HGNC Symbol;Acc:HGNC:31865]
ENSG0000	149.8201	150.38	126.0772	348.9279	291.0838	297.6117	142.0924	312.5411	-1.135051675	1.63E-11	2.85E-10	SECTM1	17	82321024	82334074	-	2961	protein_cod	secreted and transmembrane 1 [Source:HGNC Symbol;Acc:HGNC:10707]
ENSG0000	86.19787	85.0425	68.66703	171.5723	245.6019	254.8411	79.96913	224.0051	-1.485082327	1.66E-11	2.90E-10	THAP2	12	71663009	71680639	+	6527	protein_cod	THAP domain containing 2 [Source:HGNC Symbol;Acc:HGNC:20854]
ENSG0000	153.9248	110.9701	146.3396	336.3974	266.5236	406.3201	137.0781	336.4137	-1.296014932	1.71E-11	2.98E-10	GAL3ST4	7	100159244	100168750	-	3397	protein_cod	galactose-3-O-sulfotransferase 4 [Source:HGNC Symbol;Acc:HGNC:24145]
ENSG0000	9.235486	6.222622	10.1312	43.37502	80.95768	115.8369	8.52977	80.05652	-3.236134807	1.75E-11	3.04E-10	PRCD	17	76527586	76553578	+	5305	protein_cod	photoreceptor disc component [Source:HGNC Symbol;Acc:HGNC:32528]
ENSG0000	94.40719	67.41174	73.16979	238.0807	177.3792	238.8022	78.32957	218.0873	-1.47579198	1.83E-11	3.17E-10	UNC13A	19	17601328	17688365	-	11188	protein_cod	unc-13 homolog A [Source:HGNC Symbol;Acc:HGNC:23150]
ENSG0000	16.41864	9.333933	18.01103	80.00281	105.5179	74.84844	14.58787	86.78971	-2.577448504	1.95E-11	3.36E-10	AL137009.1	6	118934770	119182745	+	1812	antisense	novel transcript, antisense to FAM184A
ENSG0000	73.88388	52.89229	55.15876	3.855557	7.277095	7.128423	60.64498	6.087025	3.3099621	1.96E-11	3.39E-10	HRNR	1	152212082	152224193	-	9623	protein_cod	hornerin [Source:HGNC Symbol;Acc:HGNC:20846]
ENSG0000	15.39248	17.63076	19.13671	78.07503	84.59622	111.3816	17.38665	91.35096	-2.396752081	2.02E-11	3.47E-10	AC024560.2	3	197634315	197635811	+	1497	processed_p	protein phosphatase 4, regulatory subunit 2 (PPP4R2) pseudogene
ENSG0000	6.15699	19.70497	3.377067	93.49726	67.31312	91.77844	9.746343	84.19628	-3.09792976	2.05E-11	3.53E-10	AC010478.1	5	51372736	51383332	-	7152	antisense	uncharacterized LOC642366 [Source:NCBI gene;Acc:642366]

ENSG0000	9.235486	21.77918	21.38809	124.3417	63.67458	130.0937	17.46759	106.0367	-2.606559613	3.01E-11	5.10E-10	GP6	19	55013705	55038264	-	2703	protein_coc	glycoprotein VI platelet [Source:HGNC Symbol;Acc:HGNC:14388]
ENSG0000	14.36631	22.81628	25.89085	121.4501	78.22877	109.5995	21.02448	103.0928	-2.298282501	3.25E-11	5.50E-10	AC126564.1	12	131235511	131237523	+	2013	TEC	novel transcript
ENSG0000	60.54374	74.67147	78.79823	172.5362	191.9334	220.0901	71.33781	194.8532	-1.452212286	3.39E-11	5.72E-10	AL590560.1	1	159854870	159867685	+	7134	antisense	uncharacterized LOC107985216 [Source:NCBI gene;Acc:107985216]
ENSG0000	144.6893	124.4524	160.9735	308.4446	352.0294	283.3548	143.3718	314.6096	-1.135256837	4.09E-11	6.84E-10	WDR5B	3	122412332	122416051	-	3720	protein_coc	WD repeat domain 5B [Source:HGNC Symbol;Acc:HGNC:17826]
ENSG0000	357.1054	434.5464	461.5325	174.464	209.2165	228.1095	417.7281	203.93	1.032488572	4.79E-11	7.95E-10	POC5	5	75674124	75717481	-	3448	protein_coc	POC5 centriolar protein [Source:HGNC Symbol;Acc:HGNC:26658]
ENSG0000	126.2183	121.3411	124.9515	270.8529	261.9754	274.4443	124.1703	269.0909	-1.115835974	4.84E-11	8.03E-10	DDIT4L	4	100185870	100190782	-	2994	protein_coc	DNA damage inducible transcript 4 like [Source:HGNC Symbol;Acc:HGNC:30555]
ENSG0000	51.30825	40.44704	57.41014	145.5473	143.7226	172.8643	49.72181	154.0447	-1.634274508	5.30E-11	8.75E-10	ZNF782	9	96816472	96875623	-	4622	protein_coc	zinc finger protein 782 [Source:HGNC Symbol;Acc:HGNC:33110]
ENSG0000	1.026165	0	0	71.32781	122.801	101.58	0.342055	98.5696	-8.017983844	5.62E-11	9.26E-10	MGAT4C	12	85955666	86838904	-	27763	protein_coc	MGAT4 family member C [Source:HGNC Symbol;Acc:HGNC:30871]
ENSG0000	72.85772	76.74567	91.18081	207.2362	180.1081	245.9306	80.2614	211.0916	-1.397494557	6.25E-11	1.03E-09	CCT6P3	7	65038354	65074713	+	3920	transcribed_	chaperonin containing TCP1 subunit 6 pseudogene 3 [Source:HGNC Symbol;Acc:HGNC:35137]
ENSG0000	439.1986	381.6542	353.4664	215.9112	197.3912	172.8643	391.4397	195.3889	1.004262922	6.80E-11	1.11E-09	CCDC150	2	196639554	196763490	+	9683	protein_coc	coiled-coil domain containing 150 [Source:HGNC Symbol;Acc:HGNC:26834]
ENSG0000	62.59607	34.22442	29.26792	161.9334	133.7166	163.0627	42.02947	152.9042	-1.857692995	7.51E-11	1.22E-09	KRT8P12	3	160565447	160569248	+	2047	transcribed_	keratin 8 pseudogene 12 [Source:HGNC Symbol;Acc:HGNC:28057]
ENSG0000	265.7768	257.2017	285.925	125.3056	126.4395	125.6385	269.6345	125.7945	1.099387014	7.70E-11	1.26E-09	ZNF845	19	53333746	53356906	+	6453	protein_coc	zinc finger protein 845 [Source:HGNC Symbol;Acc:HGNC:25112]
ENSG0000	53.36058	56.0036	46.15325	6.747225	2.72891	1.782106	51.83914	3.752747	3.807665759	7.87E-11	1.28E-09	ALPK2	18	58481247	58628957	-	9431	protein_coc	alpha kinase 2 [Source:HGNC Symbol;Acc:HGNC:20565]
ENSG0000	50.28209	59.11491	46.15325	162.8973	144.6323	153.2611	51.85008	153.5969	-1.564683689	8.14E-11	1.32E-09	PLEKHA8P1	12	45173064	45216041	-	2320	transcribed_	pleckstrin homology domain containing A8 pseudogene 1 [Source:HGNC Symbol;Acc:HGNC:30222]
ENSG0000	369.4194	352.6153	377.1058	198.5612	195.5719	150.5879	366.3802	181.5737	1.013758525	8.60E-11	1.39E-09	NTN1	17	9021542	9244000	+	6383	protein_coc	netrin 1 [Source:HGNC Symbol;Acc:HGNC:8029]
ENSG0000	152.8986	126.5267	160.9735	324.8307	282.897	327.0164	146.7996	311.5814	-1.08681142	9.84E-11	1.58E-09	DLG4	17	7187169	7219841	-	10317	protein_coc	discs large MAGUK scaffold protein 4 [Source:HGNC Symbol;Acc:HGNC:2903]
ENSG0000	46.17743	48.74387	64.16428	134.9445	166.4635	182.6658	53.02853	161.358	-1.609800253	9.88E-11	1.59E-09	AC015871.3	15	79920195	79922455	-	2261	sense_intro	novel transcript, sense intronic to ST20
ENSG0000	69.77922	75.70857	93.43219	187.9584	208.3068	211.1795	79.64	202.4816	-1.349434602	9.93E-11	1.60E-09	GLDN	15	51341629	51408013	+	7144	protein_coc	gliomedin [Source:HGNC Symbol;Acc:HGNC:29514]
ENSG0000	3.078495	6.222622	2.251378	77.11115	47.30111	45.4437	3.850832	56.61865	-3.865771709	1.03E-10	1.65E-09	VIPR2	7	159028175	159144957	-	5222	protein_coc	vasoactive intestinal peptide receptor 2 [Source:HGNC Symbol;Acc:HGNC:12695]
ENSG0000	62.59607	57.0407	70.91841	170.6084	194.6623	158.6074	63.51839	174.626	-1.460819975	1.08E-10	1.73E-09	LINC01270	20	50292720	50314922	+	3014	lincRNA	long intergenic non-protein coding RNA 1270 [Source:HGNC Symbol;Acc:HGNC:27658]
ENSG0000	65.67456	110.9701	90.05513	13.49445	12.73492	23.16737	88.89993	16.46558	2.428408404	1.27E-10	2.01E-09	FAM72C	1	143955364	143971965	-	2297	protein_coc	family with sequence similarity 72 member C [Source:HGNC Symbol;Acc:HGNC:30602]
ENSG0000	171.3696	124.4524	127.2029	286.2751	301.0898	352.8569	141.0083	313.4073	-1.151306392	1.34E-10	2.12E-09	AL133325.3	20	21499261	21502934	-	3674	lincRNA	novel transcript
ENSG0000	219.5993	175.2705	240.8975	91.56949	85.50586	65.04686	211.9224	80.7074	1.393651087	1.38E-10	2.18E-09	PRELID2	5	145471799	145835369	-	5375	protein_coc	PRELI domain containing 2 [Source:HGNC Symbol;Acc:HGNC:28306]
ENSG0000	20.5233	38.37267	40.52481	150.3667	103.6986	131.8758	33.14031	128.6471	-1.96050501	1.38E-10	2.18E-09	CDHR5	11	616565	626078	-	3873	protein_coc	cadherin related family member 5 [Source:HGNC Symbol;Acc:HGNC:7521]
ENSG0000	7.183155	10.37104	12.38258	84.82226	44.5722	97.12476	9.978924	75.50641	-2.925468799	1.52E-10	2.40E-09	SSTR2	17	73165012	73176633	+	7852	protein_coc	somatostatin receptor 2 [Source:HGNC Symbol;Acc:HGNC:11331]
ENSG0000	214.4685	210.532	207.1268	24.09723	3.638547	16.03895	210.7091	14.59158	3.85584351	1.52E-10	2.40E-09	ALPP	2	232378534	232382889	+	3059	protein_coc	alkaline phosphatase, placental [Source:HGNC Symbol;Acc:HGNC:439]
ENSG0000	27.70646	23.85338	10.1312	112.7751	100.06	87.32318	20.56368	100.0528	-2.2720031	1.58E-10	2.48E-09	TSPEAR	21	44497892	44711580	-	4471	protein_coc	thrombospondin type laminin G domain and EAR repeats [Source:HGNC Symbol;Acc:HGNC:1268]
ENSG0000	22.57563	47.70677	43.90187	100.2445	182.837	174.6464	38.06142	152.576	-2.007018219	1.62E-10	2.54E-09	SULT1C2	2	108288639	108309915	+	8399	protein_coc	sulfotransferase family 1C member 2 [Source:HGNC Symbol;Acc:HGNC:11456]
ENSG0000	304.771	250.9791	284.7993	138.8001	129.1684	126.5295	280.1832	131.4993	1.091869648	1.62E-10	2.54E-09	LRR1	14	49598697	49614672	+	2359	protein_coc	leucine rich repeat protein 1 [Source:HGNC Symbol;Acc:HGNC:19742]
ENSG0000	182.6574	140.009	206.0011	71.32781	58.21676	54.35422	176.2225	61.2996	1.524250258	1.62E-10	2.54E-09	SH3PXD2A-A	10	103745966	103755423	+	2217	antisense	SH3PXD2A antisense RNA 1 [Source:HGNC Symbol;Acc:HGNC:45242]
ENSG0000	3.078495	3.111311	5.628445	55.90558	50.03002	50.79001	3.939417	52.24187	-3.741880898	1.66E-10	2.60E-09	GPR183	13	99294530	99307405	-	1700	protein_coc	G protein-coupled receptor 183 [Source:HGNC Symbol;Acc:HGNC:3128]
ENSG0000	293.4832	365.0605	294.9305	166.7529	117.3431	148.8058	317.8247	144.3006	1.140598672	1.75E-10	2.73E-09	C1orf112	1	169662007	169854080	+	5967	protein_coc	chromosome 1 open reading frame 112 [Source:HGNC Symbol;Acc:HGNC:25565]
ENSG0000	50.28209	39.40994	55.15876	144.5834	174.6503	130.0937	48.2836	149.7758	-1.635309201	1.86E-10	2.91E-09	AC139795.2	5	177611253	177619754	+	1751	antisense	novel transcript
ENSG0000	120.0613	122.3782	100.1863	228.4418	260.1561	285.1369	114.2086	257.9116	-1.174173198	2.25E-10	3.47E-09	ZBED8	5	160393148	160400097	-	2925	protein_coc	zinc finger BED-type containing 8 [Source:HGNC Symbol;Acc:HGNC:30804]
ENSG0000	9.235486	6.222622	13.50827	72.2917	61.8553	65.93791	9.655459	66.69497	-2.797142001	2.33E-10	3.59E-09	AC073195.1	2	9555899	9556775	+	877	lincRNA	novel transcript
ENSG0000	126.2183	121.3411	142.9625	256.3946	287.4452	285.1369	130.174	276.3256	-1.087740857	2.34E-10	3.61E-09	TMEM161B-	5	88268895	88436685	+	4439	antisense	TMEM161B antisense RNA 1 [Source:HGNC Symbol;Acc:HGNC:43839]
ENSG0000	79.01471	78.81988	99.06064	214.9473	199.2105	206.7243	85.63174	206.9607	-1.275047346	2.64E-10	4.03E-09	DDX11L2	2	113599036	113601261	-	1398	unprocessec	DEAD/H-box helicase 11 like 2 [Source:HGNC Symbol;Acc:HGNC:37103]
ENSG0000	8.209321	14.51945	24.76516	92.53337	79.1384	85.54107	15.83131	85.73762	-2.449083343	2.84E-10	4.32E-09	SPACA6P-AS	19	51685363	51693456	-	3962	lincRNA	SPACA6P antisense RNA [Source:HGNC Symbol;Acc:HGNC:49383]
ENSG0000	361.2101	323.5764	290.4278	183.139	136.4455	139.0042	325.0714	152.8629	1.090992413	2.85E-10	4.33E-09	POPDC3	6	105158280	105179995	-	1881	protein_coc	popeye domain containing 3 [Source:HGNC Symbol;Acc:HGNC:17649]
ENSG0000	67.72689	77.78278	84.42668	220.7307	170.1021	196.9227	76.64545	195.9185	-1.355093114	2.95E-10	4.48E-09	MC1R	16	89912119	89920977	+	5566	protein_coc	melanocortin 1 receptor [Source:HGNC Symbol;Acc:HGNC:6929]
ENSG0000	23.6018	49.78098	48.40463	106.9917	170.1021	183.5569	40.5958	153.5502	-1.923710355	3.03E-10	4.58E-09	CD226	18	69831158	69961803	-	13638	protein_coc	CD226 molecule [Source:HGNC Symbol;Acc:HGNC:16961]
ENSG0000	337.6083	409.656	402.9967	151.3306	214.6743	196.9227	383.4203	187.6425	1.029119402	3.41E-10	5.12E-09	PECR	2	215996329	216082955	-	3182	protein_coc	peroxisomal trans-2-enoyl-CoA reductase [Source:HGNC Symbol;Acc:HGNC:18281]
ENSG0000	44.1251	56.0036	42.77618	1.927779	0	5.346317	47.63496	2.424699	4.283698107	3.54E-10	5.31E-09	TTC22	1	54779712	54801267	-	6055	protein_coc	tetratricopeptide repeat domain 22 [Source:HGNC Symbol;Acc:HGNC:26067]
ENSG0000	15.39248	14.51945	6.754134	58.79725	88.23477	79.3037	12.22202	75.44524	-2.616855616	3.59E-10	5.38E-09	AL024497.2	6	133452857	133456605	-	317	antisense	novel transcript
ENSG0000	9.235486	11.40814	10.1312	46.26669	96.4215	73.06633	10.25828	71.91817	-2.810889078	3.78E-10	5.65E-09	MIR4477B	9	63819574	63819654	+	81	miRNA	microRNA 4477b [Source:HGNC Symbol;Acc:HGNC:41898]
ENSG0000	275.0122	340.17	279.1709	104.1	159.1864	132.7669	298.1177	132.0178	1.173929189	3.83E-10	5.72E-09	ZNF738	19	21358930	21379302	+	3905	protein_coc	zinc finger protein 738 [Source:HGNC Symbol;Acc:HGNC:32469]
ENSG0000	109.7997	129.638	123.8258	35.6639	20.92165	42.77054	121.0878	33.1187	1.869842916	3.91E-10	5.82E-09	IL20RB	3	136946230	137011085	+	2473	protein_coc	interleukin 20 receptor subunit beta [Source:HGNC Symbol;Acc:HGNC:6004]
ENSG0000	93.38102	68.44884	52.90739	13.49445	12.73492	8.910529	71.57908	11.7133	2.616552022	4.19E-10	6.21E-09	ATP6V0A4	7	138706294	138799560	-	4124	protein_coc	ATPase H+ transporting V0 subunit a4 [Source:HGNC Symbol;Acc:HGNC:866]
ENSG0000	191.8929	166.9737	164.3506	305.5529	350.2102	437.507	174.4057	364.4233	-1.06327846	4.27E-10	6.32E-09	ZNF814	19	57848731	57889074	-	12600	protein_coc	zinc finger protein 814 [Source:HGNC Symbol;Acc:HGNC:33258]
ENSG0000	135.4538	164.8995	176.7332	319.0474	309.2765	335.0359	159.0288	321.1199	-1.015570192	4.37E-10	6.46E-09	AC048341.1	12	62602752	62622213	+	8693	lincRNA	novel transcript
ENSG0000	95.43335	94.37644	102.4377	18.3139	30.92765	24.05843	97.41583	24.43333	1.991958865	4.57E-10	6.74E-09	ISPD	7	16087527	16421322	-	6309	protein_coc	isoprenoid synthase domain containing [Source:HGNC Symbol;Acc:HGNC:37276]
ENSG0000	81.06704	66.37464	112.5689	11.56667	21.83128	17.82106	86.67019	17.073	2.337822547	4.88E-10	7.17E-09	SERTAD4	1	210232799	210246631	+	5467	protein_coc	SERTA domain containing 4 [Source:HGNC Symbol;Acc:HGNC:25236]
ENSG0000	62.59607	48.74387	39.39912	97.35282	259.2465	229.8916	50.24635	195.497	-1.958641296	5.02E-10	7.37E-09	CXCL8	4	73740506	73743716	+	2274	protein_coc	C-X-C motif chemokine ligand 8 [Source:HGNC Symbol;Acc:HGNC:6025]
ENSG0000	115.9567	126.5267	114.8203	287.239	269.2525	225.4364	119.1012	260.6426	-1.128751258	5.56E-10	8.12E-09	PDE2A	11	72576141	72674591	-	7182	protein_coc	phosphodiesterase 2A [Source:HGNC Symbol;Acc:HGNC:8777]
ENSG0000	988.197	1018.436	444.6472	139.764	166.4635	169.3	817.0933	158.5092	2.36596058	6.19E-10	8.97E-09	EIF3C	16	28688558	28735730	+	6982	protein_coc	eukaryotic translation initiation factor 3 subunit C [Source:HGNC Symbol;Acc:HGNC:3279]
ENSG0000	8.209321	12.44524	10.1312	67.47225	73.68058	56.13633	10.26192	65.76305	-2.679942544	6.20E-10	8.97E-09	AC021087.1	5	288833	290321	-	1203	processed_p	protein phosphatase 4, regulatory subunit 2 (PPP4R2) pseudogene
ENSG0000	217.547	231.2741	199.247	96.38893	101.8793	83.75897	216.0227	94.00907	1.201518862	6.48E-10	9.36E-09	NTN4	12	95657807	95791152	-	4166	protein_coc	netrin 4 [Source:HGNC Symbol;Acc:HGNC:13658]
ENSG0000	77.98855	106.8217	87.80375	191.814	231.9574	229.0006	90.87132	217.5906	-1.260121446	7.34E-10	1.05E-08	RAB11FIP1P	X	74202834	74204595	+	1762	processed_p	RAB11 family interacting protein 1 pseudogene 1 [Source:HGNC Symbol;Acc:HGNC:42366]
ENSG0000	28.73262	24.89049	20.2624	80.9667	140.9937	91.77844	24.6285	104.5796	-2.083524682	8.50E-10	1.21E-08	LINC01844	5	142745600	142760998	+	2237	lincRNA	long intergenic non-protein coding RNA 1844 [Source:HGNC Symbol;Acc:HGNC:52660]
ENSG0000	17.44481	20.74207	14.63396	60.72503	94.60223	104.2532	17.60695	86.52681	-2.295292866	8.63E-10	1.22E-08	AC013448.1	2	223498773	223504611	+	808	lincRNA	novel transcript
ENSG0000	11.28782	18.66787	20.2624	56.86947	99.15041	107.8174	16.73936	87.94576	-2.399522086	8.81E-10	1.25E-08	RGS17	6	153004459	153131249	-	7899	protein_coc	regulator of G protein signaling 17 [Source:HGNC Symbol;Acc:HGNC:14088]
ENSG0000	177.5266	198.0868	165.4763	47.23058	87.32513	54.35422	180.3632	62.96998	1.516942772	9.38E-10	1.32E-08	ALG10	12	34022281	34029694	+	4389	protein_coc	ALG10, alpha-1,2-glucosyltransferase [Source:HGNC Symbol;Acc:HGNC:23162]
ENSG0000	20.5233	22.81628	31.51929	91.56949	120.9817	87.32318	24.95296	99.95812	-2.0074023	9.59E-10	1.35E-08	AC068473.5	18	79677287	79679358	-	2072	lincRNA	novel transcript
ENSG0000	11.28782	24.89049	29.26792	98.31671	90.05404	99.79792	21.81541	96.05623	-2.145701588	9.76E-10	1.37E-08	PGAM1P8	11	65174117	65176470	-	2354	transcribed_	phosphoglycerate mutase 1 pseudogene 8 [Source:HGNC Symbol;Acc:HGNC:42455]
ENSG0000	9.235486	12.44524	10.1312	72.2917	60.03603	62.3737	10.60398	64.90048	-2.612424293	1.00E-09	1.40E-08	AC016773.1	4	1712821	1713622	+	802	lincRNA	novel transcript
ENSG0000	18.47097	34.22442	27.01654	93.49726	92.78296	134.549	26.57064	106.9431	-2.010711319	1.07E-09	1.51E-08	MYO1F	19	8520790	8577577	-	8549	protein_coc	myosin IF [Source:HGNC Symbol;Acc:HGNC:7600]
ENSG0000	356.0793	339.1329	321.9471	108.9195	161.0057	191.5764	339.0531	153.8339	1.138052304	1.13E-09	1.57E-08	MDFIC	7	114922154	115019202	+	5252	protein_coc	MyoD family inhibitor domain containing [Source:HGNC Symbol;Acc:HGNC:28870]
ENSG0000	345.8176	330.8361	361.3462	111.8112	151.9093	203.1601	346	155.6269	1.150093308	1.24E-09	1.72E-08	ZNF100	19	21722766	21767628	-	6612	protein_coc	zinc finger protein 100 [Source:HGNC Symbol;Acc:HGNC:12880]
ENSG0000	65.67456	60.15201	63.03859	184.1029	139.1744	177.3195	62.95506	166.8656	-1.405845336	1.31E-09	1.81E-08	MYH7B	20	34975403	35002437	+	7931	protein_coc	myosin heavy chain 7B [Source:HGNC Symbol;Acc:HGNC:15906]

ENSG0000	25.65413	16.59366	9.005513	74.21948	88.23477	94.4516	17.08443	85.63528	-2.316173599	1.31E-09	1.81E-08	AC009061.2	16	67481314	67505063	+	2770	antisense	uncharacterized LOC101927837 [Source:NCBI gene;Acc:101927837]
ENSG0000	23.6018	10.37104	23.63947	74.21948	100.06	94.4516	19.2041	89.57704	-2.226138221	1.34E-09	1.85E-08	AL645728.1	1	1574102	1577075	+	2349	processed_t	novel transcript
ENSG0000	14.36631	18.66787	29.26792	94.46115	70.95167	121.1832	20.76736	95.532	-2.20986044	1.42E-09	1.95E-08	AC107375.1	8	140505813	140508043	-	2231	antisense	novel transcript, antisense to CHRAC1
ENSG0000	21.54947	23.85338	20.2624	84.82226	95.51187	84.65002	21.88842	88.32805	-2.01142767	1.46E-09	2.01E-08	AC092123.1	16	89492017	89504460	-	1045	antisense	uncharacterized LOC101927863 [Source:NCBI gene;Acc:101927863]
ENSG0000	33.86345	33.18732	34.89636	106.0278	143.7226	98.90687	33.98238	116.2191	-1.774606555	1.47E-09	2.02E-08	AC027763.1	17	6927472	6928191	-	720	processed_p	Spi-C transcription factor (Spi-1/PU.1 related) (SPIC) pseudogene
ENSG0000	157.0033	190.8271	175.6075	67.47225	51.8493	79.3037	174.4793	66.20842	1.397372062	1.49E-09	2.05E-08	SYNE3	14	95407266	95475836	-	18164	protein_cod	spectrin repeat containing nuclear envelope family member 3 [Source:HGNC Symbol;Acc:HGNC:19861]
ENSG0000	112.8782	97.48775	117.0717	210.1279	264.7043	247.7127	109.1459	240.8483	-1.143425486	1.63E-09	2.22E-08	HAPLN3	15	88877288	88895626	-	3524	protein_cod	hyaluronan and proteoglycan link protein 3 [Source:HGNC Symbol;Acc:HGNC:21446]
ENSG0000	2.05233	2.074207	0	54.94169	32.74693	59.70054	1.375513	49.12972	-5.123608202	1.64E-09	2.23E-08	RN7SL521P	7	149125690	149125986	-	297	misc_RNA	RNA, 7SL, cytoplasmic 521, pseudogene [Source:HGNC Symbol;Acc:HGNC:46537]
ENSG0000	13.34015	5.185518	12.38258	53.01391	70.95167	75.73949	10.30275	66.56836	-2.696315513	1.66E-09	2.27E-08	AC110285.6	17	81345476	81345966	-	491	lincRNA	novel transcript
ENSG0000	53.36058	28.0018	31.51929	123.3778	121.8913	135.44	37.62723	126.9031	-1.750720485	1.74E-09	2.37E-08	AL669831.5	1	778770	810060	+	5517	lincRNA	novel transcript
ENSG0000	118.009	102.6733	133.957	246.7557	250.1501	254.8411	118.2131	250.5823	-1.085818854	1.82E-09	2.49E-08	AP006621.5	11	779617	780755	+	1139	TEC	TEC
ENSG0000	21.54947	36.29863	20.2624	82.89448	97.33114	147.9148	26.03683	109.3801	-2.068816134	2.07E-09	2.80E-08	AC008764.6	19	16542746	16544814	+	2069	lincRNA	novel transcript
ENSG0000	292.457	361.9492	372.6031	152.2945	150.0901	200.4869	342.3364	167.6238	1.028431697	2.15E-09	2.90E-08	PARP10	8	143977153	144012772	-	6033	protein_cod	poly(ADP-ribose) polymerase family member 10 [Source:HGNC Symbol;Acc:HGNC:25895]
ENSG0000	71.83155	93.33933	95.68357	236.1529	165.5539	257.5143	86.95149	219.7404	-1.338940959	2.29E-09	3.09E-08	FAM238C	10	26931206	26944418	-	5545	transcribed_	family with sequence similarity 238 member C [Source:HGNC Symbol;Acc:HGNC:24672]
ENSG0000	150.8463	152.4542	189.1158	62.65281	67.31312	62.3737	164.1388	64.11321	1.354826881	2.30E-09	3.10E-08	IFIT2	10	89283694	89309276	+	4101	protein_cod	interferon induced protein with tetratricopeptide repeats 2 [Source:HGNC Symbol;Acc:HGNC:5409]
ENSG0000	17.44481	13.48235	18.01103	68.43614	83.68659	75.73949	16.31273	75.95407	-2.22151386	2.52E-09	3.38E-08	AC109326.1	17	43360041	43361361	-	1321	TEC	TEC
ENSG0000	68.75306	90.22802	82.1753	162.8973	227.4092	205.8332	80.38546	198.7132	-1.307052998	2.66E-09	3.56E-08	PJVK	2	178451346	178461409	+	3788	protein_cod	pejvakin [Source:HGNC Symbol;Acc:HGNC:29502]
ENSG0000	105.695	80.89409	69.79272	213.9834	202.849	196.0316	85.4606	204.288	-1.254090124	2.86E-09	3.81E-08	PPP1R3E	14	23295643	23302848	-	5548	protein_cod	protein phosphatase 1 regulatory subunit 3E [Source:HGNC Symbol;Acc:HGNC:14943]
ENSG0000	29.75879	24.89049	15.75965	100.2445	91.87332	87.32318	23.46964	93.147	-1.981721931	3.11E-09	4.13E-08	AC092117.1	16	2777319	2780568	+	3250	lincRNA	novel transcript
ENSG0000	45.15126	66.37464	45.02756	5.783336	4.548184	8.910529	52.18449	6.414016	3.021298692	3.22E-09	4.27E-08	KRT81	12	52285913	52291534	-	1929	protein_cod	keratin 81 [Source:HGNC Symbol;Acc:HGNC:6458]
ENSG0000	231.9133	216.7547	177.8589	86.75004	94.60223	94.4516	208.8423	91.93462	1.184383063	3.47E-09	4.58E-08	STARD8	X	68647666	68725842	+	5558	protein_cod	StAR related lipid transfer domain containing 8 [Source:HGNC Symbol;Acc:HGNC:19161]
ENSG0000	221.6517	242.6823	219.5094	98.31671	109.1564	114.0548	227.9478	107.176	1.088041204	3.60E-09	4.74E-08	CENPL	1	173799550	173824720	-	4914	protein_cod	centromere protein L [Source:HGNC Symbol;Acc:HGNC:17879]
ENSG0000	192.919	183.5674	222.8864	724.8448	480.2882	1000.652	199.7909	735.2618	-1.880174457	3.71E-09	4.87E-08	SNHG19	16	2154797	2155358	-	342	lincRNA	small nucleolar RNA host gene 19 [Source:HGNC Symbol;Acc:HGNC:49574]
ENSG0000	58.49141	76.74567	61.9129	5.783336	10.006	16.93	65.71666	10.90645	2.582328918	3.77E-09	4.95E-08	CYP4F8	19	15615218	15630638	+	7048	protein_cod	cytochrome P450 family 4 subfamily F member 8 [Source:HGNC Symbol;Acc:HGNC:2648]
ENSG0000	127.2445	133.7864	146.3396	292.0585	267.4332	256.6232	135.7901	272.0383	-1.003105944	3.78E-09	4.97E-08	HIST3H2A	1	228456979	228457873	-	895	protein_cod	histone cluster 3 H2A [Source:HGNC Symbol;Acc:HGNC:20507]
ENSG0000	65.67456	62.22622	49.53032	159.0417	153.7286	149.6969	59.1437	154.1557	-1.379135654	3.97E-09	5.20E-08	CCSER1	4	90127535	91601913	+	10474	protein_cod	coiled-coil serine rich protein 1 [Source:HGNC Symbol;Acc:HGNC:29349]
ENSG0000	180.6051	148.3058	169.979	75.18337	66.40349	47.2258	166.2966	62.93755	1.40425561	4.03E-09	5.26E-08	KRTAP3-1	17	41008521	41019324	-	725	protein_cod	keratin associated protein 3-1 [Source:HGNC Symbol;Acc:HGNC:16778]
ENSG0000	7.183155	9.333933	7.879823	47.23058	63.67458	57.91844	8.132304	56.27453	-2.791124634	4.07E-09	5.31E-08	AL590326.1	14	104815449	104815753	-	305	TEC	novel transcript
ENSG0000	8.209321	8.29683	18.01103	50.12224	79.1384	77.5216	11.50573	68.92742	-2.596159901	4.44E-09	5.77E-08	AC069544.1	10	14877688	14878686	-	999	lincRNA	novel transcript
ENSG0000	3.078495	6.222622	6.754134	61.68892	33.65656	60.59159	5.351751	51.97902	-3.287228516	4.45E-09	5.78E-08	HSPA8P14	12	111381867	111385638	+	1805	processed_p	heat shock protein family A (Hsp70) member 8 pseudogene 14 [Source:HGNC Symbol;Acc:HGNC:44929]
ENSG0000	6.15699	4.148415	0	59.76114	33.65656	51.68107	3.435135	48.36626	-3.785580952	4.72E-09	6.12E-08	AC127496.5	17	81017969	81020011	-	935	lincRNA	novel transcript
ENSG0000	119.0351	96.45064	95.68357	222.6584	201.9394	318.1059	103.7231	247.5679	-1.254711647	4.76E-09	6.17E-08	EFEMP2	11	65866441	65873592	-	5397	protein_cod	EGF containing fibulin extracellular matrix protein 2 [Source:HGNC Symbol;Acc:HGNC:3219]
ENSG0000	124.166	126.5267	159.8478	315.1918	246.5116	290.4832	136.8468	284.0622	-1.055363275	5.00E-09	6.47E-08	PSD	10	102402617	102421539	-	6338	protein_cod	pleckstrin and Sec7 domain containing [Source:HGNC Symbol;Acc:HGNC:9507]
ENSG0000	382.7596	291.4261	339.9581	126.2695	158.2768	197.8137	338.0479	160.7867	1.07027637	5.05E-09	6.53E-08	LIN9	1	226231149	226309869	-	4442	protein_cod	lin-9 DREAM MuvB core complex component [Source:HGNC Symbol;Acc:HGNC:30830]
ENSG0000	48.22976	67.41174	41.6505	129.1612	173.7406	147.9148	52.43066	150.2722	-1.516850148	5.28E-09	6.81E-08	MEX3B	15	82041778	82046141	-	4364	protein_cod	mex-3 RNA binding family member B [Source:HGNC Symbol;Acc:HGNC:25297]
ENSG0000	12.31398	4.148415	6.754134	61.68892	52.75894	55.24528	7.738843	56.56438	-2.864316323	5.34E-09	6.89E-08	AC007743.1	2	56173534	56185770	-	3713	antisense	uncharacterized LOC100129434 [Source:NCBI gene;Acc:100129434]
ENSG0000	13.34015	9.333933	5.628445	53.9778	53.66857	76.63055	9.434175	61.42564	-2.693810513	5.79E-09	7.44E-08	AC058791.1	7	130936464	130939661	+	3198	lincRNA	novel transcript
ENSG0000	62.59607	42.52125	43.90187	2.891668	6.367458	8.019476	49.67306	5.759534	3.099089969	5.82E-09	7.48E-08	LYZ	12	69348341	69354234	+	1764	protein_cod	lysozyme [Source:HGNC Symbol;Acc:HGNC:6740]
ENSG0000	59.51757	74.67147	48.40463	173.5001	146.4515	161.2806	60.86456	160.4107	-1.394791434	5.96E-09	7.65E-08	ODF3B	22	50529710	50532580	-	2301	protein_cod	outer dense fiber of sperm tails 3B [Source:HGNC Symbol;Acc:HGNC:34388]
ENSG0000	5.130825	2.074207	5.628445	40.48335	45.48184	52.57212	4.277826	46.1791	-3.440717991	6.04E-09	7.75E-08	LINC01144	1	45303910	45305619	+	1710	lincRNA	long intergenic non-protein coding RNA 1144 [Source:HGNC Symbol;Acc:HGNC:49460]
ENSG0000	18.47097	40.44704	31.51929	110.8473	100.9697	111.3816	30.14577	107.7329	-1.838915712	6.08E-09	7.79E-08	SSSCA1-AS1	11	65568482	65570423	-	1942	processed_t	SSSCA1 antisense RNA 1 (head to head) [Source:HGNC Symbol;Acc:HGNC:27384]
ENSG0000	1016.93	1015.325	930.9449	1688.734	2595.194	2643.754	987.733	2309.227	-1.225249398	6.14E-09	7.86E-08	FTX	X	73946555	74293574	-	28487	lincRNA	FTX transcript, XIST regulator [Source:HGNC Symbol;Acc:HGNC:37190]
ENSG0000	12.31398	11.40814	16.88534	50.12224	75.49986	89.99634	13.53582	71.87281	-2.415315015	6.23E-09	7.96E-08	ZSCAN16-AS	6	28121795	28137293	-	2163	antisense	ZSCAN16 antisense RNA 1 [Source:HGNC Symbol;Acc:HGNC:48982]
ENSG0000	26.68029	44.59546	36.02205	167.7167	83.68659	136.3311	35.76593	129.2448	-1.853094942	6.33E-09	8.08E-08	CLEC18B	16	74408270	74421953	-	3143	protein_cod	C-type lectin domain family 18 member B [Source:HGNC Symbol;Acc:HGNC:33849]
ENSG0000	3.078495	8.29683	1.125689	46.26669	53.66857	43.66159	4.167005	47.86562	-3.50391706	6.38E-09	8.15E-08	RPL7AP73	13	22803296	22803648	+	353	processed_p	ribosomal protein L7a pseudogene 73 [Source:HGNC Symbol;Acc:HGNC:41968]
ENSG0000	53.36058	56.0036	55.15876	135.9084	130.9877	179.1016	54.84098	148.6659	-1.439611945	6.42E-09	8.20E-08	TMEM51-AS	1	15111815	15153237	-	7873	antisense	TMEM51 antisense RNA 1 [Source:HGNC Symbol;Acc:HGNC:26301]
ENSG0000	17.44481	13.48235	10.1312	68.43614	75.49986	62.3737	13.68612	68.7699	-2.322877102	7.00E-09	8.89E-08	GBP2	1	89106132	89150456	-	6276	protein_cod	guanylate binding protein 2 [Source:HGNC Symbol;Acc:HGNC:4183]
ENSG0000	5.130825	7.259726	1.125689	40.48335	44.5722	60.59159	4.505413	48.54905	-3.41117882	7.23E-09	9.16E-08	GNGT1	7	93591573	93911265	+	2361	protein_cod	G protein subunit gamma transducin 1 [Source:HGNC Symbol;Acc:HGNC:4411]
ENSG0000	25.65413	22.81628	18.01103	92.53337	101.8793	70.39318	22.16048	88.26862	-1.989632689	7.50E-09	9.48E-08	TMEM132B	12	125186836	125662377	+	15703	protein_cod	transmembrane protein 132B [Source:HGNC Symbol;Acc:HGNC:29397]
ENSG0000	5.130825	11.40814	11.25689	71.32781	48.21075	59.70054	9.265286	59.74637	-2.694137882	8.02E-09	1.01E-07	AC110285.1	17	81375144	81385464	-	2039	lincRNA	uncharacterized LOC100130370 [Source:NCBI gene;Acc:100130370]
ENSG0000	245.2535	206.3836	273.5424	79.03892	121.8913	115.8369	241.7265	105.589	1.192008307	8.33E-09	1.04E-07	ZNF675	19	23525631	23687220	-	6785	protein_cod	zinc finger protein 675 [Source:HGNC Symbol;Acc:HGNC:30768]
ENSG0000	197.0237	175.2705	194.7442	67.47225	88.23477	89.10529	189.0128	81.6041	1.209705818	8.61E-09	1.08E-07	RDM1	17	35918066	35930773	-	1975	protein_cod	RAD52 motif containing 1 [Source:HGNC Symbol;Acc:HGNC:19950]
ENSG0000	110.8258	135.8606	113.6946	215.9112	279.2585	266.4248	120.127	253.8648	-1.079700445	8.74E-09	1.09E-07	AC121761.1	5	75483454	75489820	-	1482	antisense	novel transcript, antisense to GLIPR1
ENSG0000	60.54374	63.26333	105.8148	187.9584	186.4755	206.7243	76.54061	193.7194	-1.344990606	9.81E-09	1.22E-07	AC055713.1	12	123575891	123585115	-	8279	lincRNA	novel transcript
ENSG0000	46.17743	67.41174	38.27343	134.9445	157.3672	139.8953	50.62087	144.069	-1.505802993	9.94E-09	1.24E-07	PDE3B	11	14643723	14872044	+	11707	protein_cod	phosphodiesterase 3B [Source:HGNC Symbol;Acc:HGNC:8779]
ENSG0000	27.70646	38.37284	48.40463	1.927779	0.909637	2.673159	38.16131	1.836858	4.372523899	1.03E-08	1.28E-07	AL031846.1	22	39091586	39092855	+	640	transcribed_	apolipoprotein B mRNA editing enzyme, catalytic polypeptide-like 3 (APOBEC3) family pseudogene
ENSG0000	197.0237	201.1981	171.1047	95.42504	71.86131	81.08581	189.7755	82.79072	1.198988383	1.14E-08	1.41E-07	HEG1	3	124965710	125055958	-	9925	protein_cod	heart development protein with EGF like domains 1 [Source:HGNC Symbol;Acc:HGNC:29227]
ENSG0000	13.34015	9.333933	10.1312	56.86947	59.12639	65.04686	10.93509	60.34757	-2.462499014	1.19E-08	1.47E-07	SLC6A12	12	190077	214570	-	5280	protein_cod	solute carrier family 6 member 12 [Source:HGNC Symbol;Acc:HGNC:11045]
ENSG0000	91.32869	85.0425	103.5634	205.3084	225.5899	186.23	93.31153	205.7095	-1.141896379	1.23E-08	1.51E-07	AC241952.1	1	120489860	120701289	-	9444	transcribed_	phosphodiesterase 4D interacting protein-like [Source:NCBI gene;Acc:100996724]
ENSG0000	65.67456	44.59546	59.66152	141.6917	140.9937	165.7358	56.64385	149.4738	-1.400808192	1.24E-08	1.52E-07	FAM71E1	19	50466785	50476753	-	1566	protein_cod	family with sequence similarity 71 member E1 [Source:HGNC Symbol;Acc:HGNC:25107]
ENSG0000	91.32869	95.41514	106.9405	209.164	188.2948	253.9501	97.89423	217.1363	-1.151067086	1.26E-08	1.55E-07	CATSPER2	15	43628503	43668118	-	8100	protein_cod	cation channel sperm associated 2 [Source:HGNC Symbol;Acc:HGNC:18810]
ENSG0000	10.26165	11.40814	20.2624	55.90558	93.69259	66.82896	13.9774	72.14238	-2.377788306	1.30E-08	1.59E-07	AC011462.4	19	41373971	41374419	+	449	sense_intro	novel transcript, sense intronic TMEM91
ENSG0000	109.7997	147.2687	101.312	31.80835	44.5722	40.09738	119.4601	38.82598	1.620484715	1.32E-08	1.62E-07	DSC3	18	30990008	31042815	-	7056	protein_cod	desmocollin 3 [Source:HGNC Symbol;Acc:HGNC:3037]
ENSG0000	61.5699	23.85338	52.90739	143.6195	146.4515	143.4595	46.11023	144.5102	-1.649404481	1.46E-08	1.78E-07	TRPV6	7	142871203	142885762	-	11925	protein_cod	transient receptor potential cation channel subfamily V member 6 [Source:HGNC Symbol;Acc:HGNC:14006]
ENSG0000	1.026165	10.37104	6.754134	53.9778	43.66257	59.70054	6.050445	52.44697	-3.121013902	1.62E-08	1.96E-07	AL355987.5	9	136728953	136729855	-	411	unprocesse	ral guanine nucleotide dissociation stimulator (RALGDS) pseudogene
ENSG0000	159.0556	144.1574	118.1974	37.59168	64.58421	24.94948	140.4701	42.37513	1.730100458	1.63E-08	1.98E-07	NAV2	11	19350724	20121598	+	14673	protein_cod	neuron navigator 2 [Source:HGNC Symbol;Acc:HGNC:15997]
ENSG0000	102.6165	117.1927	99.06064	178.3195	269.2525	278.0085	106.29	241.8602	-1.186615645	1.80E-08	2.17E-07	C8orf44	8	66667615	66685564	+	2505	protein_cod	chromosome 8 open reading frame 44 [Source:HGNC Symbol;Acc:HGNC:25646]
ENSG0000	11.28782	4.148415	16.88534	71.32781	67.31312	53.46317	10.77386	64.0347	-2.582033754	1.83E-08	2.21E-07	AC145207.9	17	81862778	81863102	+	325	TEC	TEC
ENSG0000	28.73262	38.37284	68.66703	148.439	142.813	130.9848	45.2575	140.7456	-1.644127106	1.86E-08	2.24E-07	GTF2IRD2	7	74796144	74851551	-	7549	protein_cod	GTF2I repeat domain containing 2 [Source:HGNC Symbol;Acc:HGNC:30775]

ENSG0000	16.41864	36.29863	31.51929	103.1362	114.6142	87.32318	28.07885	101.6912	-1.859212887	1.94E-08	2.33E-07	AC012358.2	2	55224280	55225908	+	1434	processed_p	pseudogene similar to RIKEN cDNA 2210021J22
ENSG0000	92.35486	60.15201	77.67255	167.7167	179.1985	204.9422	76.72647	183.9525	-1.261917124	1.97E-08	2.36E-07	TMEM9B-AS	11	8964675	8977527	+	1162	antisense	TMEM9B antisense RNA 1 [Source:HGNC Symbol;Acc:HGNC:19230]
ENSG0000	9.235486	11.40814	5.628445	49.15836	69.1324	49.89896	8.757357	56.06324	-2.670833463	1.97E-08	2.37E-07	RIMKLB	12	8681600	8783095	+	8619	protein_cod	ribosomal modification protein rimK like family member B [Source:HGNC Symbol;Acc:HGNC:29228]
ENSG0000	14.36631	11.40814	24.76516	98.31671	64.58421	70.39318	16.84654	77.7647	-2.214410081	2.01E-08	2.41E-07	COL6A3	2	237324003	237414375	-	19633	protein_cod	collagen type VI alpha 3 chain [Source:HGNC Symbol;Acc:HGNC:2213]
ENSG0000	146.7416	184.6045	141.8368	49.15836	65.49385	70.39318	157.7276	61.68179	1.352879987	2.06E-08	2.47E-07	CA8	8	60187347	60281412	-	5783	protein_cod	carbonic anhydrase 8 [Source:HGNC Symbol;Acc:HGNC:1382]
ENSG0000	99.53801	88.15381	75.42117	237.1168	169.1924	200.4869	87.70433	202.2654	-1.202747254	2.38E-08	2.83E-07	AC078846.1	7	129604548	129611630	-	7083	lincRNA	novel transcript
ENSG0000	272.9599	295.5746	273.5424	92.53337	171.9214	114.9458	280.6923	126.4669	1.148849742	2.46E-08	2.92E-07	XK	X	37685756	37732130	+	5209	protein_cod	X-linked Kx blood group [Source:HGNC Symbol;Acc:HGNC:12811]
ENSG0000	36.94194	20.74207	29.26792	111.8112	97.33114	88.21423	28.98398	99.11884	-1.772746271	2.61E-08	3.09E-07	AL121832.3	20	62402236	62405935	-	3700	sense_intro	novel transcript, sense intronic to CABLES2
ENSG0000	14.36631	26.9647	16.88534	98.31671	67.31312	78.41265	19.40545	81.3475	-2.065008214	2.66E-08	3.15E-07	AC087741.3	17	80147250	80148596	+	1347	TEC	TEC
ENSG0000	32.83728	46.66967	47.27894	108.9195	120.9817	134.549	42.26196	121.4834	-1.526311356	2.72E-08	3.21E-07	C1S	12	6988259	7071032	+	6462	protein_cod	complement C1s [Source:HGNC Symbol;Acc:HGNC:1247]
ENSG0000	91.32869	90.22802	75.42117	179.2834	181.9274	206.7243	85.65929	189.3117	-1.142696707	3.24E-08	3.78E-07	SNHG10	14	95532297	95534872	-	1945	antisense	small nucleolar RNA host gene 10 [Source:HGNC Symbol;Acc:HGNC:27510]
ENSG0000	145.7154	141.0461	129.4542	50.12224	60.03603	56.13633	138.7386	55.43153	1.323179466	3.44E-08	4.00E-07	IQCD	12	113195441	113221094	-	2460	protein_cod	IQ motif containing D [Source:HGNC Symbol;Acc:HGNC:25168]
ENSG0000	0	5.185518	1.125689	54.94169	25.46983	44.55264	2.103736	41.65472	-4.296938218	3.55E-08	4.12E-07	AC127496.2	17	81023545	81025503	-	368	lincRNA	novel transcript
ENSG0000	4.10466	8.29683	4.502756	45.3028	40.93366	51.68107	5.634749	45.97251	-3.02454933	3.62E-08	4.19E-07	AC123768.2	15	32613733	32615111	+	732	lincRNA	novel transcript
ENSG0000	1.026165	2.074207	3.377067	27.95279	40.93366	46.33475	2.159147	38.40707	-4.173198253	3.69E-08	4.28E-07	AP000894.4	18	813274	813756	+	483	lincRNA	novel transcript
ENSG0000	3.078495	15.55656	10.1312	53.01391	56.39748	71.28423	9.588751	60.23187	-2.654695008	3.92E-08	4.52E-07	AC233280.1	3	195655565	195657927	-	1684	lincRNA	novel transcript
ENSG0000	109.7997	122.3782	123.8258	22.16945	55.48785	24.05843	118.6679	33.90524	1.804947458	4.19E-08	4.81E-07	RASGRP3	2	33436324	33564750	+	11118	protein_cod	RAS guanyl releasing protein 3 [Source:HGNC Symbol;Acc:HGNC:14545]
ENSG0000	1.026165	6.222622	2.251378	38.55557	30.92765	56.13633	3.166722	41.87318	-3.720306622	4.28E-08	4.91E-07	PTGES2-AS1	9	128128529	128130628	+	2100	lincRNA	PTGES2 antisense RNA 1 (head to head) [Source:NCBI gene;Acc:389791]
ENSG0000	81.06704	131.7122	109.1918	203.3806	245.6019	261.0785	107.3237	236.687	-1.142247798	4.40E-08	5.03E-07	FBXL19-AS1	16	30919319	30923269	-	3951	antisense	FBXL19 antisense RNA 1 [Source:HGNC Symbol;Acc:HGNC:27557]
ENSG0000	76.96238	66.37464	78.79823	12.53056	22.74092	19.60316	74.04508	18.29155	2.012670404	4.41E-08	5.03E-07	KRT20	17	40875941	40885227	-	1846	protein_cod	keratin 20 [Source:HGNC Symbol;Acc:HGNC:20412]
ENSG0000	51.30825	64.30043	72.0441	150.3667	148.2708	157.7164	62.55093	152.118	-1.284814131	4.43E-08	5.05E-07	FAM86HP	3	130099092	130111472	-	1301	transcribed_	family with sequence similarity 86 member H, pseudogene [Source:HGNC Symbol;Acc:HGNC:42359]
ENSG0000	20.5233	19.70497	20.2624	57.83336	88.23477	94.4516	20.16356	80.17324	-1.992883682	4.49E-08	5.12E-07	TTC34	1	2635976	2801717	-	8814	protein_cod	tetratricopeptide repeat domain 34 [Source:HGNC Symbol;Acc:HGNC:34297]
ENSG0000	222.6778	185.6416	201.4983	99.2806	93.69259	98.90687	203.2726	97.29335	1.06330608	4.50E-08	5.13E-07	OIP5	15	41309268	41332621	-	1236	protein_cod	Opa interacting protein 5 [Source:HGNC Symbol;Acc:HGNC:20300]
ENSG0000	17.44481	19.70497	14.63396	59.76114	85.50586	72.17528	17.26124	72.48076	-2.067937829	4.60E-08	5.23E-07	ZNF300	5	150894392	150904983	-	4411	protein_cod	zinc finger protein 300 [Source:HGNC Symbol;Acc:HGNC:13091]
ENSG0000	66.70073	59.11491	56.28445	13.49445	15.46383	8.910529	60.70003	12.62293	2.268595061	4.61E-08	5.24E-07	ZNF85	19	20923222	20950697	+	4668	protein_cod	zinc finger protein 85 [Source:HGNC Symbol;Acc:HGNC:13160]
ENSG0000	38.99427	53.92939	37.14774	144.5834	121.8913	106.0353	43.35713	124.17	-1.515045134	4.69E-08	5.33E-07	GSTA4	6	52977948	52995378	-	2175	protein_cod	glutathione S-transferase alpha 4 [Source:HGNC Symbol;Acc:HGNC:4629]
ENSG0000	35.91578	67.41174	24.76516	1.927779	0	6.23737	42.69756	2.721716	3.960232901	4.83E-08	5.46E-07	AC068580.2	11	1776930	1778388	-	338	lincRNA	novel transcript
ENSG0000	58.49141	44.59546	50.65601	133.9806	141.9033	122.9653	51.24763	132.9498	-1.374692419	4.84E-08	5.46E-07	NRSN2-AS1	20	320313	348224	-	875	antisense	NRSN2 antisense RNA 1 [Source:HGNC Symbol;Acc:HGNC:51222]
ENSG0000	207.2853	183.5674	159.8478	87.71393	51.8493	85.54107	183.5668	75.03477	1.292515542	5.14E-08	5.78E-07	CYP4F2	19	15878023	15898120	-	3142	protein_cod	cytochrome P450 family 4 subfamily F member 2 [Source:HGNC Symbol;Acc:HGNC:2645]
ENSG0000	255.5151	332.9103	215.0066	133.9806	130.0781	124.7474	267.8107	129.602	1.048657172	5.21E-08	5.86E-07	E2F8	11	19224063	19241620	-	4140	protein_cod	E2F transcription factor 8 [Source:HGNC Symbol;Acc:HGNC:24727]
ENSG0000	15.39248	11.40814	19.13671	54.94169	74.59022	75.73949	15.31244	68.4238	-2.165633129	5.34E-08	5.99E-07	SOD1P3	8	125951861	125952314	-	454	processed_p	superoxide dismutase 1 pseudogene 3 [Source:HGNC Symbol;Acc:HGNC:45136]
ENSG0000	64.6484	82.9683	104.6891	188.9223	192.843	190.6853	84.10193	190.8169	-1.185631545	5.39E-08	6.04E-07	ZBTB12	6	31899607	31901992	-	1875	protein_cod	zinc finger and BTB domain containing 12 [Source:HGNC Symbol;Acc:HGNC:19066]
ENSG0000	10.26165	12.44524	16.88534	51.08613	67.31312	72.17528	13.19741	63.52485	-2.274304836	5.47E-08	6.12E-07	AC009962.1	2	181887851	181891663	-	3813	antisense	novel transcript, antisense to SSFA2
ENSG0000	63.62223	34.22442	47.27894	139.764	139.1744	123.8563	48.3752	134.2649	-1.471548067	5.49E-08	6.13E-07	RFTN1	3	16313574	16514026	-	4776	protein_cod	raftlin, lipid raft linker 1 [Source:HGNC Symbol;Acc:HGNC:30278]
ENSG0000	75.93622	77.78278	72.0441	201.4529	143.7226	179.9927	75.25436	175.0561	-1.216706666	5.52E-08	6.16E-07	FNDC11	20	63547891	63556708	+	3625	protein_cod	fibronectin type III domain containing 11 [Source:HGNC Symbol;Acc:HGNC:28764]
ENSG0000	18.47097	29.0389	51.7817	122.4139	125.5299	97.12476	33.09719	115.0229	-1.80542142	5.63E-08	6.28E-07	AC091563.1	8	120052180	120056201	+	752	lincRNA	novel transcript
ENSG0000	15.39248	11.40814	11.25689	51.08613	63.67458	70.39318	12.68584	61.71796	-2.280617464	5.82E-08	6.48E-07	REM2	14	22883165	22887686	+	2305	protein_cod	RRAD and GEM like GTPase 2 [Source:HGNC Symbol;Acc:HGNC:20248]
ENSG0000	59.51757	66.37464	77.67255	194.7056	195.5719	124.7474	67.85492	171.675	-1.340290167	6.01E-08	6.68E-07	MAP1LC3B2	12	116548105	116576448	+	1767	protein_cod	microtubule associated protein 1 light chain 3 beta 2 [Source:HGNC Symbol;Acc:HGNC:34390]
ENSG0000	12.31398	8.29683	7.879823	53.01391	47.30111	62.3737	9.496878	54.22958	-2.509364321	6.26E-08	6.94E-07	AC126407.1	15	23567264	23569567	-	566	antisense	MKRN3 antisense RNA 1
ENSG0000	12.31398	30.07601	22.51378	73.25559	90.96368	89.10529	21.63459	84.44152	-1.966854648	6.34E-08	7.03E-07	PRKG1	10	50990891	52298350	+	12831	protein_cod	protein kinase cGMP-dependent 1 [Source:HGNC Symbol;Acc:HGNC:9414]
ENSG0000	107.7473	136.8977	122.7001	316.1557	231.0478	222.7632	122.4484	256.6556	-1.066751935	6.37E-08	7.05E-07	AC009053.1	16	74332402	74368240	-	3828	transcribed_	pyruvate dehydrogenase phosphatase regulatory subunit pseudogene [Source:NCBI gene;Acc:283922]
ENSG0000	27.70646	29.0389	34.89636	75.18337	114.6142	108.7084	30.54724	99.50202	-1.707409619	6.56E-08	7.25E-07	IGDCC3	15	65327127	65378040	-	5552	protein_cod	immunoglobulin superfamily DCC subclass member 3 [Source:HGNC Symbol;Acc:HGNC:9700]
ENSG0000	51.30825	74.67147	56.28445	171.5723	145.5419	138.1132	60.75472	151.7425	-1.319050172	6.63E-08	7.32E-07	SLC44A3-AS	1	94613814	94855426	-	10245	transcribed_	SLC44A3 antisense RNA 1 [Source:HGNC Symbol;Acc:HGNC:49057]
ENSG0000	116.9828	94.37644	151.968	213.9834	278.3489	288.7011	121.1091	260.3445	-1.107639037	6.69E-08	7.38E-07	ZNF213-AS1	16	3110460	3134882	-	5145	antisense	ZNF213 antisense RNA 1 (head to head) [Source:HGNC Symbol;Acc:HGNC:50505]
ENSG0000	69.77922	58.07781	64.16428	133.9806	171.9214	155.0432	64.0071	153.6484	-1.263842228	6.82E-08	7.52E-07	ANK2	4	112818109	113383740	+	18224	protein_cod	ankyrin 2 [Source:HGNC Symbol;Acc:HGNC:493]
ENSG0000	93.38102	134.8235	90.05513	30.84446	38.20475	34.75106	106.0865	34.60009	1.616233011	7.29E-08	7.99E-07	AC091057.6	15	30624548	30685606	+	2297	protein_cod	Rho GTPase-activating protein 11B [Source:UniProtKB/Swiss-Prot;Acc:Q3KRB8]
ENSG0000	62.59607	54.9665	64.16428	166.7529	120.0721	166.0269	60.57561	151.1506	-1.319647927	7.29E-08	7.99E-07	PLIN4	19	4502180	4518465	-	6502	protein_cod	perilipin 4 [Source:HGNC Symbol;Acc:HGNC:29393]
ENSG0000	5.130825	10.37104	1.125689	51.08613	50.93966	40.09738	5.542517	47.37439	-3.076877781	7.44E-08	8.13E-07	LINC00870	3	72151257	72207770	+	3739	lincRNA	long intergenic non-protein coding RNA 870 [Source:HGNC Symbol;Acc:HGNC:27319]
ENSG0000	66.70073	97.48775	101.312	210.1279	170.1021	220.9811	88.50016	200.4037	-1.18127355	8.34E-08	9.08E-07	MYO15A	17	18108706	18179806	+	20002	protein_cod	myosin XVA [Source:HGNC Symbol;Acc:HGNC:7594]
ENSG0000	122.1136	132.7493	176.7332	48.19447	50.03002	66.82896	143.8654	55.01782	1.383292014	8.35E-08	9.08E-07	YBX2	17	7288252	7294615	-	4618	protein_cod	Y-box binding protein 2 [Source:HGNC Symbol;Acc:HGNC:17948]
ENSG0000	17.44481	9.333933	9.005513	93.49726	50.93966	50.79001	11.92808	65.07565	-2.44023866	8.55E-08	9.28E-07	AQP7P1	9	63317067	63333091	-	1045	unprocesse	aquaporin 7 pseudogene 1 [Source:HGNC Symbol;Acc:HGNC:32048]
ENSG0000	6.15699	6.222622	15.75965	63.6167	54.57821	48.11685	9.379753	55.43725	-2.577654514	8.72E-08	9.46E-07	KISS1R	19	917287	921015	+	1680	protein_cod	KISS1 receptor [Source:HGNC Symbol;Acc:HGNC:4510]
ENSG0000	165.2126	145.1945	142.9625	227.4779	343.8427	342.1643	151.1232	304.495	-1.011092097	9.01E-08	9.74E-07	AC138393.1	1	223992743	224010612	+	4349	transcribed_	septin 7 (SEPT7) pseudogene
ENSG0000	76.96238	67.41174	90.05513	186.9945	152.819	189.7943	78.14308	176.5359	-1.177732516	9.62E-08	1.04E-06	FAM157C	16	90102271	90178344	+	4524	lincRNA	family with sequence similarity 157 member C [Source:HGNC Symbol;Acc:HGNC:34081]
ENSG0000	6.15699	3.111311	2.251378	45.3028	31.83729	41.87948	3.839893	39.67319	-3.355756969	1.06E-07	1.14E-06	DANT2	X	115917271	115969089	-	2079	lincRNA	DXZ4 associated non-coding transcript 2, distal [Source:HGNC Symbol;Acc:HGNC:50862]
ENSG0000	100.5642	110.9701	96.80926	241.9362	194.6623	199.5958	102.7812	212.0648	-1.043396448	1.07E-07	1.15E-06	GRID2IP	7	6497462	6551436	-	4134	protein_cod	Grid2 interacting protein [Source:HGNC Symbol;Acc:HGNC:18464]
ENSG0000	3235.498	2912.187	2888.518	1063.17	1699.202	1566.471	3012.068	1442.947	1.06157758	1.11E-07	1.19E-06	ANP32E	1	150218417	150236156	-	3605	protein_cod	acidic nuclear phosphoprotein 32 family member E [Source:HGNC Symbol;Acc:HGNC:16673]
ENSG0000	1.026165	1.037104	1.125689	37.59168	33.65656	37.42422	1.062986	36.22416	-5.092794807	1.19E-07	1.27E-06	LINC00622	1	119597702	119599271	-	1570	sense_overl	long intergenic non-protein coding RNA 622 [Source:HGNC Symbol;Acc:HGNC:44251]
ENSG0000	250.3843	233.3483	245.4002	117.5945	146.4515	96.23371	243.0443	120.0932	1.01739412	1.19E-07	1.27E-06	ELOVL3	10	102226328	102229589	+	1371	protein_cod	ELOVL fatty acid elongase 3 [Source:HGNC Symbol;Acc:HGNC:18047]
ENSG0000	8.209321	15.55656	9.005513	48.19447	53.66857	71.28423	10.9238	57.71576	-2.399618688	1.22E-07	1.30E-06	AC009148.1	16	81310731	81313423	-	2693	antisense	novel transcript, antisense to GAN
ENSG0000	2.05233	6.222622	5.628445	59.76114	34.5662	33.86001	4.634466	42.72912	-3.209988938	1.36E-07	1.44E-06	AC092171.4	7	5425770	5426401	+	632	lincRNA	novel transcript
ENSG0000	93.38102	105.7846	138.4598	221.6945	228.3188	237.0201	112.5418	229.0111	-1.028290859	1.40E-07	1.49E-06	GABPB1-IT1	15	50348936	50354861	-	5926	sense_intro	GABPB1 intronic transcript [Source:HGNC Symbol;Acc:HGNC:25469]
ENSG0000	0	0	0	21.20557	22.74092	30.2958	0	24.74743	-6.985810842	1.42E-07	1.50E-06	BX539320.1	22	49933198	49934074	-	877	lincRNA	novel transcript
ENSG0000	8.209321	11.40814	14.63396	60.72503	51.8493	56.13633	11.41714	56.23689	-2.306541796	1.57E-07	1.65E-06	UNC5C	4	95162504	95549206	-	10685	protein_cod	unc-5 netrin receptor C [Source:HGNC Symbol;Acc:HGNC:12569]
ENSG0000	10.26165	8.29683	11.25689	48.19447	46.39148	65.04686	9.938457	53.21093	-2.424155571	1.59E-07	1.67E-06	LINC01481	12	69904033	70243360	-	1490	antisense	long intergenic non-protein coding RNA 1481 [Source:NCBI gene;Acc:101928062]
ENSG0000	37.96811	42.52125	52.90739	125.3056	122.801	106.9263	44.46558	118.3443	-1.415035576	1.60E-07	1.69E-06	HOXD8	2	176129694	176132695	+	2629	protein_cod	homeobox D8 [Source:HGNC Symbol;Acc:HGNC:5139]
ENSG0000	47.20359	58.07781	68.66703	121.4501	161.0057	140.7864	56.2543	141.0807	-1.330549715	1.63E-07	1.71E-06	SCAMP1-AS	5	78342365	78360507	-	1792	lincRNA	SCAMP1 antisense RNA 1 [Source:HGNC Symbol;Acc:HGNC:50544]
ENSG0000	144.6893	137.9348	141.8368	27.95279	73.68058	42.77054	141.487	48.13464	1.552583705	1.68E-07	1.76E-06	CMPK2	2	6840570	6866635	-	4302	protein_cod	cytidine/uridine monophosphate kinase 2 [Source:HGNC Symbol;Acc:HGNC:27015]
ENSG0000	137.5061	128.6009	127.2029	26.9889	43.66257	65.04686	131.1033	45.23278	1.530814587	1.75E-07	1.83E-06	PADI1	1	17205126	17246005	+	4903	protein_cod	peptidyl arginine deiminase 1 [Source:HGNC Symbol;Acc:HGNC:18367]

ENSG0000	111.852	129.638	178.9846	257.3584	270.1621	315.4327	140.1582	280.9844	-1.007067749	1.75E-07	1.83E-06	AC016831.6	7	130791264	131110161	-	11088	lincRNA	novel transcript
ENSG0000	91.32869	81.93119	66.41565	16.38612	30.92765	16.93	79.89185	21.41459	1.898928708	1.76E-07	1.84E-06	ALDH1L2	12	105019784	105084577	-	8507	protein_cod	aldehyde dehydrogenase 1 family member L2 [Source:HGNC Symbol;Acc:HGNC:26777]
ENSG0000	95.43335	78.81988	57.41014	205.3084	164.6443	168.409	77.22112	179.4539	-1.212248536	1.81E-07	1.89E-06	ZCCHC12	X	118823790	118826968	+	2231	protein_cod	zinc finger CCHC-type containing 12 [Source:HGNC Symbol;Acc:HGNC:27273]
ENSG0000	2.05233	1.037104	3.377067	30.84446	31.83729	39.20633	2.1555	33.96269	-3.995450214	1.91E-07	1.98E-06	AP001412.1	21	37267784	37268497	+	714	antisense	novel transcript, antisense to DSCR3
ENSG0000	40.02044	32.15021	24.76516	116.6306	110.9757	77.5216	32.31194	101.7093	-1.649283923	1.94E-07	2.01E-06	U73166.1	3	50260303	50263358	+	2625	lincRNA	novel transcript
ENSG0000	83.11937	73.63436	77.67255	182.1751	158.2768	166.6269	78.14209	169.0263	-1.112430697	1.97E-07	2.05E-06	LINC01569	16	4243943	4253817	-	5237	lincRNA	long intergenic non-protein coding RNA 1569 [Source:HGNC Symbol;Acc:HGNC:51380]
ENSG0000	1.026165	2.074207	2.251378	46.26669	19.10237	40.98843	1.783917	35.4525	-4.321417771	2.00E-07	2.07E-06	RF02246	2	44932320	44932406	+	87	misc_RNA	
ENSG0000	25.65413	16.59366	29.26792	101.2084	67.31312	84.65002	23.83857	84.39051	-1.826572343	2.07E-07	2.14E-06	AC245100.4	1	148402516	148432545	+	3330	transcribed_	phosphodiesterase 4D interacting protein (PDE4DIP) pseudogene
ENSG0000	55.41291	54.9665	57.41014	2.891668	14.55419	12.47474	55.92985	9.973532	2.477122917	2.12E-07	2.19E-06	EGF	4	109912884	110012266	+	6127	protein_cod	epidermal growth factor [Source:HGNC Symbol;Acc:HGNC:3229]
ENSG0000	35.91578	53.92939	38.27343	9.638893	2.72891	4.455264	42.7062	5.607689	2.941591043	2.20E-07	2.26E-06	CREG2	2	101345551	101387595	-	7150	protein_cod	cellular repressor of E1A stimulated genes 2 [Source:HGNC Symbol;Acc:HGNC:14272]
ENSG0000	61.5699	60.15201	63.03859	130.1251	149.1804	148.8058	61.58684	142.7038	-1.213236285	2.22E-07	2.29E-06	AC011450.1	19	49331617	49340303	-	615	lincRNA	novel transcript
ENSG0000	8.209321	12.44524	11.25689	53.01391	54.57821	51.68107	10.63715	53.09106	-2.321175518	2.33E-07	2.39E-06	AC110769.2	2	113888203	113889750	-	1548	lincRNA	novel transcript
ENSG0000	28.73262	48.74387	52.90739	1.927779	10.006	0.891053	43.46129	4.274945	3.340519586	2.42E-07	2.47E-06	ACTBL2	5	57480016	57482809	-	2794	protein_cod	actin, beta like 2 [Source:HGNC Symbol;Acc:HGNC:17780]
ENSG0000	5.130825	15.55656	2.251378	57.83336	40.93366	61.48265	7.646253	53.41655	-2.791148069	2.42E-07	2.47E-06	ASTL	2	96123850	96138436	-	1654	protein_cod	astacin like metalloendopeptidase [Source:HGNC Symbol;Acc:HGNC:31704]
ENSG0000	18.47097	26.9647	34.89636	79.03892	110.0661	81.08581	26.77734	90.0636	-1.755812899	2.43E-07	2.48E-06	CD4	12	6786858	6820808	+	4583	protein_cod	CD4 molecule [Source:HGNC Symbol;Acc:HGNC:1678]
ENSG0000	16.41864	22.81628	15.75965	89.64171	52.75894	76.63055	18.33152	73.0104	-1.990663841	2.51E-07	2.56E-06	FAM27E3	9	67717411	67719178	-	1593	transcribed_	family with sequence similarity 27 member E3 [Source:HGNC Symbol;Acc:HGNC:28655]
ENSG0000	10.26165	12.44524	4.502756	70.36392	38.20475	52.57212	9.069884	53.7136	-2.554461342	2.62E-07	2.66E-06	AC124944.2	3	195937243	195938739	+	1242	processed_	protein phosphatase 4, regulatory subunit 2 (PPP4R2) pseudogene
ENSG0000	26.68029	50.81808	22.51378	1.927779	3.638547	0.891053	33.33738	2.15246	3.956794582	2.65E-07	2.69E-06	KRT16P6	17	16817983	16822579	-	1906	transcribed_	keratin 16 pseudogene 6 [Source:HGNC Symbol;Acc:HGNC:50719]
ENSG0000	86.19787	59.11491	99.06064	181.2112	183.7466	177.3195	81.4578	180.7591	-1.152579756	2.97E-07	3.00E-06	SPAG4	20	35615892	35621049	+	2240	protein_cod	sperm associated antigen 4 [Source:HGNC Symbol;Acc:HGNC:11214]
ENSG0000	7.183155	21.77918	11.25689	59.76114	57.30712	73.06633	13.40641	63.3782	-2.23964916	3.08E-07	3.10E-06	SYPL1P2	17	27351858	27352584	-	727	processed_	synaptophysin like 1 pseudogene 2 [Source:HGNC Symbol;Acc:HGNC:53547]
ENSG0000	9.235486	19.70497	20.2624	68.43614	66.40349	65.93791	16.40095	66.92585	-2.034038689	3.08E-07	3.11E-06	AC009120.3	16	74313337	74315634	+	513	lincRNA	novel transcript
ENSG0000	11.28782	14.51945	19.13671	61.68892	65.49385	58.80949	14.98133	61.99742	-2.054970557	3.41E-07	3.42E-06	PCDHB9	5	141187127	141191541	+	4415	protein_cod	protocadherin beta 9 [Source:HGNC Symbol;Acc:HGNC:8694]
ENSG0000	2.05233	22.81628	14.63396	96.38893	55.48785	68.61107	13.16752	73.49595	-2.483237423	3.53E-07	3.53E-06	KCNA7	19	49067418	49072941	-	4372	protein_cod	potassium voltage-gated channel subfamily A member 7 [Source:HGNC Symbol;Acc:HGNC:6226]
ENSG0000	7.183155	9.333933	5.628445	34.70002	43.66257	62.3737	7.381845	46.91209	-2.664352678	3.58E-07	3.58E-06	MUC20P1	3	195614947	195620233	+	1680	unprocesse	mucin 20, cell surface associated pseudogene 1 [Source:HGNC Symbol;Acc:HGNC:51921]
ENSG0000	22.57563	28.0018	33.77067	85.78615	79.1384	98.01581	28.11603	87.64679	-1.644423792	3.59E-07	3.59E-06	AL109976.1	20	3239705	3245382	+	5678	lincRNA	novel transcript
ENSG0000	159.0556	169.0479	155.3451	73.25559	84.59622	65.04686	161.1495	74.29956	1.117530179	3.73E-07	3.72E-06	AC022150.4	19	52650437	52653284	-	2848	sense_intro	novel transcript, sense intronic to ZNF83
ENSG0000	22.57563	35.26153	10.1312	80.00281	81.86731	96.23371	22.65612	86.03461	-1.917002934	3.88E-07	3.86E-06	AC104964.3	8	10486807	10489666	+	2860	lincRNA	novel transcript
ENSG0000	87.22403	78.81988	82.1753	12.53056	22.74092	33.86001	82.73974	23.04383	1.837914473	4.12E-07	4.08E-06	CCL28	5	43376645	43412391	-	4524	protein_cod	C-C motif chemokine ligand 28 [Source:HGNC Symbol;Acc:HGNC:17700]
ENSG0000	47.20359	50.81808	48.40463	118.5584	112.795	129.2027	48.80877	120.1853	-1.300195482	4.22E-07	4.18E-06	KLHL31	6	53647901	53665708	-	5743	protein_cod	kelch like family member 31 [Source:HGNC Symbol;Acc:HGNC:21353]
ENSG0000	52.33442	59.11491	96.80926	133.0167	187.3852	199.5958	69.41953	173.3326	-1.32584896	4.31E-07	4.27E-06	AL662795.2	6	30282349	30286054	-	3706	TEC	TEC
ENSG0000	33.86345	25.92759	20.2624	87.71393	72.77095	98.90687	26.68448	86.46391	-1.691739756	4.41E-07	4.35E-06	Z84485.1	6	36146698	36197205	-	1463	antisense	novel transcript, antisense to BRPF3
ENSG0000	37.96811	67.41174	39.39912	137.8362	122.801	122.9653	48.25965	127.8675	-1.40321717	4.42E-07	4.36E-06	PPM1N	19	45488777	45502510	+	2571	protein_cod	protein phosphatase, Mg2+/Mn2+ dependent 1N (putative) [Source:HGNC Symbol;Acc:HGNC:26845]
ENSG0000	309.9019	291.4261	280.2966	98.31671	176.4695	164.8848	293.8749	146.5437	1.001943462	4.49E-07	4.43E-06	Mar-01	4	163524298	164384050	-	10421	protein_cod	membrane associated ring-CH-type finger 1 [Source:HGNC Symbol;Acc:HGNC:26077]
ENSG0000	0	3.111311	1.125689	28.91668	39.11438	28.51369	1.412333	32.18158	-4.508128365	4.54E-07	4.47E-06	BICC1	10	58513140	58831437	+	6569	protein_cod	BicC family RNA binding protein 1 [Source:HGNC Symbol;Acc:HGNC:19351]
ENSG0000	171.3696	147.2687	127.2029	61.68892	71.86131	62.3737	148.6137	65.30798	1.187148763	4.60E-07	4.53E-06	CENPP	9	92325484	92620533	+	9794	protein_cod	centromere protein P [Source:HGNC Symbol;Acc:HGNC:32933]
ENSG0000	11.28782	6.222622	6.754134	47.23058	40.02402	53.46317	8.088191	46.90592	-2.531376883	4.61E-07	4.54E-06	AC010201.2	12	89367807	89369301	+	1495	lincRNA	novel transcript
ENSG0000	21.54947	38.37284	41.6505	73.25559	105.5179	163.9537	33.8576	114.2424	-1.759536848	4.62E-07	4.54E-06	AL117336.3	10	35314552	35336401	-	1412	antisense	novel transcript, antisense to CCNY
ENSG0000	101.5903	173.1963	164.3506	320.9751	245.6019	325.2343	146.3791	297.2705	-1.023385085	4.64E-07	4.56E-06	MUC16	19	8848844	8981342	-	43830	protein_cod	mucin 16, cell surface associated [Source:HGNC Symbol;Acc:HGNC:15582]
ENSG0000	44.1251	21.77918	63.03859	105.0639	161.9154	128.3116	42.98095	131.7636	-1.622211779	4.89E-07	4.79E-06	AMY2B	1	103553815	103579534	+	7491	protein_cod	amylase, alpha 2B (pancreatic) [Source:HGNC Symbol;Acc:HGNC:478]
ENSG0000	3.078495	4.148415	1.125689	33.73613	38.20475	28.51369	2.7842	33.48485	-3.57133155	5.02E-07	4.91E-06	AC073210.1	7	65075023	65078780	+	232	unprocesse	pseudogene similar to part of sulfatase modifying factor 2 SUMF2
ENSG0000	0	4.148415	3.377067	45.3028	46.39148	17.82106	2.508494	36.50511	-3.874635986	5.14E-07	5.02E-06	HSPD1P12	7	8015024	8016406	+	1069	processed_	heat shock protein family D (Hsp60) member 1 pseudogene 12 [Source:HGNC Symbol;Acc:HGNC:35140]
ENSG0000	52.33442	35.26153	38.27343	6.747225	9.096368	2.673159	41.95646	6.172251	2.770126122	5.76E-07	5.57E-06	KRT7	12	52232520	52252186	+	4093	protein_cod	keratin 7 [Source:HGNC Symbol;Acc:HGNC:6445]
ENSG0000	26.68029	28.0018	24.76516	71.32781	115.8301	121.1832	26.48242	88.1241	-1.734785913	5.80E-07	5.62E-06	BMPER	7	33904308	34156356	+	7919	protein_cod	BMP binding endothelial regulator [Source:HGNC Symbol;Acc:HGNC:24154]
ENSG0000	93.38102	76.74567	67.54134	154.2223	161.0057	211.1795	79.22268	175.4692	-1.145918333	5.82E-07	5.63E-06	LINC02001	3	35568109	35574843	+	1504	lincRNA	long intergenic non-protein coding RNA 2001 [Source:HGNC Symbol;Acc:HGNC:52836]
ENSG0000	57.46524	48.74387	57.41014	126.2695	115.5239	150.5879	54.53975	130.7938	-1.263021861	5.93E-07	5.72E-06	TBX1	22	19756703	19783593	+	3580	protein_cod	T-box 1 [Source:HGNC Symbol;Acc:HGNC:11592]
ENSG0000	5.130825	3.111311	7.879823	40.48335	36.38547	40.98843	5.373987	39.28575	-2.88117211	6.03E-07	5.81E-06	AC080080.1	7	17940503	17942922	+	2420	TEC	tec
ENSG0000	7.183155	4.148415	12.38258	59.76114	38.20475	44.55264	7.904717	47.50618	-2.598423713	6.16E-07	5.94E-06	AL713922.2	10	147820	148751	-	716	unprocesse	DEAD (Asp-Glu-Ala-Asp) box polypeptide 20 (DDX20) pseudogene
ENSG0000	96.45952	78.81988	115.946	32.77224	36.38547	33.86001	97.07512	34.33924	1.4973343	6.29E-07	6.05E-06	CCDC163	1	45493866	45500079	-	2757	protein_cod	coiled-coil domain containing 163 [Source:HGNC Symbol;Acc:HGNC:27003]
ENSG0000	0	2.074207	4.502756	33.73613	23.65056	41.87948	2.192321	33.08872	-3.949925336	6.30E-07	6.06E-06	AL442128.2	13	110916004	110917827	+	1824	lincRNA	novel transcript
ENSG0000	131.3491	117.1927	119.323	187.9584	294.7223	253.059	122.6216	245.2466	-1.000598544	6.46E-07	6.21E-06	ACVR1C	2	157526767	157629005	-	9195	protein_cod	activin A receptor type 1C [Source:HGNC Symbol;Acc:HGNC:18123]
ENSG0000	84.14554	79.85698	99.06064	198.5612	173.7406	168.409	87.68772	180.2369	-1.04065559	6.86E-07	6.55E-06	LINC01770	1	1430539	1434573	-	851	lincRNA	long intergenic non-protein coding RNA 1770 [Source:HGNC Symbol;Acc:HGNC:52560]
ENSG0000	187.7882	178.3818	216.1323	83.85837	104.6082	101.58	194.1008	96.68221	1.003273141	6.95E-07	6.63E-06	IL1RAP	3	190514051	190659750	+	11558	protein_cod	interleukin 1 receptor accessory protein [Source:HGNC Symbol;Acc:HGNC:5995]
ENSG0000	232.9395	230.237	244.2745	107.9556	151.9093	91.77844	235.817	117.2145	1.008255348	6.98E-07	6.66E-06	AC068831.7	15	90966190	91022566	-	7148	protein_cod	novel transcript
ENSG0000	14.36631	13.48235	12.38258	69.40003	50.93966	51.68107	13.41041	57.34025	-2.093375974	7.01E-07	6.68E-06	AC008764.8	19	16572624	16575340	+	2717	antisense	novel transcript, antisense to MED26
ENSG0000	13.34015	12.44524	13.50827	74.21948	36.38547	70.39318	13.09789	60.33271	-2.203456427	7.13E-07	6.79E-06	HSPE1P21	6	133510386	133510693	-	308	processed_	heat shock protein family E (Hsp10) member 1 pseudogene 21 [Source:HGNC Symbol;Acc:HGNC:49340]
ENSG0000	9.235486	15.55656	6.754134	58.79725	52.75894	45.4437	10.51539	52.33329	-2.307670542	7.32E-07	6.96E-06	SPTLC3	20	13008979	13169103	+	8520	protein_cod	serine palmitoyltransferase long chain base subunit 3 [Source:HGNC Symbol;Acc:HGNC:16253]
ENSG0000	13.34015	12.44524	15.75965	56.86947	54.57821	59.70054	13.84835	57.04941	-2.045415252	7.36E-07	6.99E-06	AL512274.1	6	42092233	42094259	-	2027	lincRNA	novel transcript
ENSG0000	73.83888	88.15381	93.43219	201.4529	165.5539	165.7358	85.15663	177.5809	-1.061038615	7.63E-07	7.23E-06	AL118506.1	20	63861212	63864293	-	3082	antisense	novel transcript, antisense to ABHD16B
ENSG0000	28.73262	29.0389	15.75965	70.36392	80.04804	94.4516	24.51039	81.62119	-1.73020693	7.74E-07	7.32E-06	DRD4	11	637293	640706	+	1477	protein_cod	dopamine receptor D4 [Source:HGNC Symbol;Acc:HGNC:3025]
ENSG0000	66.70073	46.66967	86.67806	135.9084	171.0117	166.6269	66.68282	157.849	-1.247521942	7.80E-07	7.37E-06	RF00019	16	14915457	14915556	-	100	misc_RNA	
ENSG0000	12.31398	17.63076	14.63396	47.23058	67.31312	67.72002	14.85957	60.75457	-2.032922228	7.87E-07	7.43E-06	PCDHGA6	5	141373914	141512979	+	7910	protein_cod	protocadherin gamma subfamily A, 6 [Source:HGNC Symbol;Acc:HGNC:8704]
ENSG0000	14.36631	10.37104	9.005513	62.65281	43.66257	52.57212	11.24762	52.9625	-2.229828018	8.18E-07	7.70E-06	GLTPD2	17	4788959	4790390	+	1023	protein_cod	glycolipid transfer protein domain containing 2 [Source:HGNC Symbol;Acc:HGNC:33756]
ENSG0000	81.06704	64.30043	76.54686	154.2223	153.7286	164.8848	73.97144	157.5986	-1.091735751	8.33E-07	7.83E-06	RPS10P7	1	201518703	201584367	+	3018	transcribed_	ribosomal protein S10 pseudogene 7 [Source:HGNC Symbol;Acc:HGNC:36423]
ENSG0000	0	0	0	21.20557	15.46383	24.05843	0	20.24261	-6.695188902	8.40E-07	7.88E-06	AC074099.1	2	156305108	156320255	-	679	lincRNA	novel transcript
ENSG0000	37.96811	37.33573	34.89636	95.42504	105.5179	93.56055	36.7334	98.16782	-1.417327112	8.59E-07	8.05E-06	ARMC4	10	27812164	27999048	-	4773	protein_cod	armadillo repeat containing 4 [Source:HGNC Symbol;Acc:HGNC:25583]
ENSG0000	7.183155	2.074207	11.25689	61.68892	33.65656	42.77054	6.838085	46.03867	-2.763275257	8.95E-07	8.37E-06	SNX32	11	65833641	65856896	+	3966	protein_cod	sorting nexin 32 [Source:HGNC Symbol;Acc:HGNC:26423]
ENSG0000	16.41864	21.77918	20.2624	71.32781	50.03002	98.90687	19.48674	73.42157	-1.915296112	9.01E-07	8.41E-06	MROH7	1	54641754	54710266	+	9088	protein_cod	maestro heat like repeat family member 7 [Source:HGNC Symbol;Acc:HGNC:24802]
ENSG0000	6.15699	4.148415	19.13671	64.58058	40.02402	62.3737	9.81404	55.65943	-2.52163359	9.48E-07	8.83E-06	AC103706.1	8	133573183	133573861	+	679	lincRNA	novel transcript
ENSG0000	10.26165	1.037104	7.879823	52.05002	35.47584	46.33475	6.392859	44.6202	-2.805885195	9.55E-07	8.88E-06	AC010327.4	19	55216660	55221616	+	1577	lincRNA	novel transcript

ENSG0000	80.04088	105.7846	99.06064	34.70002	37.29511	32.0779	94.96203	34.69101	1.452566626	1.01E-06	9.35E-06	ZNF595	4	53285	88211	+	3933	protein_cod	zinc finger protein 595 [Source:HGNC Symbol;Acc:HGNC:27196]
ENSG0000	9.235486	12.44524	13.50827	43.37502	49.12039	69.50212	11.72967	53.99918	-2.207558855	1.01E-06	9.37E-06	AC008608.2	5	134005147	134005562	+	416	lincRNA	novel transcript
ENSG0000	16.41864	13.48235	16.88534	71.32781	48.21075	64.15581	15.59544	61.23146	-1.973873407	1.07E-06	9.88E-06	MIR31HG	9	21455642	21559669	-	745	sense_overl	MIR31 host gene [Source:HGNC Symbol;Acc:HGNC:37187]
ENSG0000	20.5233	32.15021	3.377067	87.71393	80.95768	81.08581	18.68353	83.25247	-2.144140519	1.09E-06	1.01E-05	AC010997.3	10	75409157	75411842	+	1890	lincRNA	uncharacterized LOC101929234 [Source:NCBI gene;Acc:101929234]
ENSG0000	9.235486	13.48235	6.754134	38.55557	47.30111	65.93791	9.823989	50.5982	-2.360089573	1.12E-06	1.03E-05	AC016831.4	7	130930209	130932206	-	1998	lincRNA	novel transcript
ENSG0000	16.41864	25.92759	27.01654	91.56949	67.31312	71.28423	23.12092	76.72228	-1.732905845	1.18E-06	1.08E-05	HOXC-AS2	12	53993810	53996785	-	2216	processed_t	HOXC cluster antisense RNA 2 [Source:HGNC Symbol;Acc:HGNC:43750]
ENSG0000	0	4.148415	0	23.13334	30.01801	40.09738	1.382805	31.08291	-4.465626727	1.21E-06	1.10E-05	AC073352.1	3	119497678	119498181	-	504	lincRNA	novel transcript
ENSG0000	16.41864	20.74207	25.89085	76.14726	70.04203	65.93791	21.01719	70.70907	-1.754444868	1.24E-06	1.13E-05	AC090502.1	12	74133166	74402535	-	2342	lincRNA	uncharacterized LOC100507377 [Source:NCBI gene;Acc:100507377]
ENSG0000	1.026165	4.148415	3.377067	22.16945	34.5662	40.98843	2.850549	32.57469	-3.523147726	1.24E-06	1.13E-05	C10orf111	10	15095385	15097319	-	1714	bidirectiona	chromosome 10 open reading frame 111 [Source:HGNC Symbol;Acc:HGNC:28582]
ENSG0000	168.2911	147.2687	207.1268	81.93059	92.78296	72.17528	174.2289	82.29628	1.08099048	1.26E-06	1.15E-05	PRADC1	2	73228006	73233238	-	1407	protein_cod	protease associated domain containing 1 [Source:HGNC Symbol;Acc:HGNC:16047]
ENSG0000	85.1717	66.37464	109.1918	195.6695	175.5599	177.3195	86.91273	182.8497	-1.075877358	1.27E-06	1.16E-05	AC132872.1	17	82293716	82294910	+	1195	lincRNA	novel transcript
ENSG0000	4.10466	7.259726	0	42.41113	31.83729	34.75106	3.788129	36.33316	-3.236802196	1.27E-06	1.16E-05	AC009237.8	2	95536117	95536495	-	379	processed_p	pseudogene similar to part of tripartite motif-containing family protein
ENSG0000	36.94194	48.74387	55.15876	83.85837	134.6262	168.409	46.94819	128.9645	-1.461884728	1.30E-06	1.19E-05	ZNF506	19	19785839	19821751	-	8414	protein_cod	zinc finger protein 506 [Source:HGNC Symbol;Acc:HGNC:23780]
ENSG0000	17.44481	12.44524	6.754134	53.01391	51.8493	59.70054	12.21473	54.85458	-2.157705805	1.33E-06	1.21E-05	ZFP69	1	40477215	40496343	+	3248	protein_cod	ZFP69 zinc finger protein [Source:HGNC Symbol;Acc:HGNC:24708]
ENSG0000	98.51185	52.89229	97.93495	180.2473	203.7586	171.9732	83.11303	185.3264	-1.158667374	1.35E-06	1.22E-05	FER1L5	2	96642737	96704887	+	10475	protein_cod	fer-1 like family member 5 [Source:HGNC Symbol;Acc:HGNC:19044]
ENSG0000	84.14554	35.26153	70.91841	155.1862	143.7226	178.2106	63.44182	159.0398	-1.326950457	1.39E-06	1.26E-05	SGK2	20	43558968	43588237	+	5184	protein_cod	SGK2, serine/threonine kinase 2 [Source:HGNC Symbol;Acc:HGNC:13900]
ENSG0000	148.7939	221.9402	178.9846	79.03892	83.68659	98.90687	183.2396	87.21079	1.070053631	1.49E-06	1.34E-05	C5orf34	5	43486701	43515145	-	3011	protein_cod	chromosome 5 open reading frame 34 [Source:HGNC Symbol;Acc:HGNC:24738]
ENSG0000	121.0875	122.3782	145.2139	53.01391	46.39148	66.82896	129.5599	55.41145	1.223307132	1.50E-06	1.35E-05	CD22	19	35319261	35347355	+	6159	protein_cod	CD22 molecule [Source:HGNC Symbol;Acc:HGNC:1643]
ENSG0000	24.62796	18.66787	28.14223	89.64171	81.86731	60.59159	23.81268	77.36687	-1.701865338	1.50E-06	1.35E-05	SLC6A13	12	220621	262873	-	4243	protein_cod	solute carrier family 6 member 13 [Source:HGNC Symbol;Acc:HGNC:11046]
ENSG0000	204.2068	154.5285	193.6185	95.42504	96.4215	82.86792	184.1179	91.57149	1.007994124	1.53E-06	1.38E-05	CLCN4	X	10156945	10237660	+	6789	protein_cod	chloride voltage-gated channel 4 [Source:HGNC Symbol;Acc:HGNC:2022]
ENSG0000	106.7212	103.7104	131.7056	51.08613	43.66257	46.33475	114.0457	47.02782	1.277599615	1.53E-06	1.38E-05	HOXA1	7	27092993	27095996	-	2539	protein_cod	homeobox A1 [Source:HGNC Symbol;Acc:HGNC:5099]
ENSG0000	91.32869	143.1203	87.80375	219.7668	212.855	220.0901	107.4176	217.5706	-1.016400029	1.54E-06	1.39E-05	AC022075.1	12	10363769	10398506	+	2830	antisense	uncharacterized LOC101928100 [Source:NCBI gene;Acc:101928100]
ENSG0000	47.20359	43.55836	47.27894	94.46115	109.1564	146.1327	46.01363	116.5834	-1.3428432	1.55E-06	1.39E-05	ARMH1	1	44674692	44725591	+	3290	protein_cod	armadillo-like helical domain containing 1 [Source:HGNC Symbol;Acc:HGNC:34345]
ENSG0000	4.10466	5.185518	10.1312	42.41113	39.11438	39.20633	6.473793	40.24395	-2.649590572	1.58E-06	1.42E-05	C9orf163	9	136483495	136486067	+	2573	lincRNA	chromosome 9 open reading frame 163 [Source:HGNC Symbol;Acc:HGNC:26718]
ENSG0000	133.4015	129.638	123.8258	53.01391	67.31312	49.00791	128.9551	56.44498	1.192172841	1.59E-06	1.42E-05	VANGL2	1	160400586	160428678	+	5340	protein_cod	VANGL planar cell polarity protein 2 [Source:HGNC Symbol;Acc:HGNC:15511]
ENSG0000	64.6484	123.4153	36.02205	16.38612	19.10237	16.03895	74.69526	17.17581	2.122756105	1.67E-06	1.48E-05	RGPD6	2	110513812	110577185	-	13309	protein_cod	RANBP2-like and GRIP domain containing 6 [Source:HGNC Symbol;Acc:HGNC:32419]
ENSG0000	10.26165	5.185518	16.88534	53.9778	48.21075	52.57212	10.7775	51.58689	-2.269930547	1.67E-06	1.49E-05	AC083880.1	7	139359032	139359566	-	535	antisense	novel transcript, antisense to LUC7L2
ENSG0000	72.85772	106.8217	85.55237	163.8612	178.2888	205.8332	88.41059	182.6611	-1.047424539	1.74E-06	1.54E-05	LINC01315	22	42364400	42369236	-	1267	lincRNA	long intergenic non-protein coding RNA 1315 [Source:HGNC Symbol;Acc:HGNC:50513]
ENSG0000	16.41864	26.9647	18.01103	90.6056	73.68058	53.46317	20.46479	72.58312	-1.823517596	1.81E-06	1.60E-05	HLA-DOA	6	33004178	33009612	-	4012	protein_cod	major histocompatibility complex, class II, DO alpha [Source:HGNC Symbol;Acc:HGNC:4936]
ENSG0000	52.33442	41.48415	58.53583	122.4139	120.9817	115.8369	50.7848	119.7442	-1.239543381	1.86E-06	1.64E-05	AC090114.2	7	128524016	128531069	-	7054	lincRNA	novel transcript
ENSG0000	29.75879	32.15021	47.27894	87.71393	99.15041	108.7084	36.39598	98.52426	-1.44230688	1.89E-06	1.66E-05	AXDND1	1	179365720	179554735	+	6089	protein_cod	axonemal dynein light chain domain containing 1 [Source:HGNC Symbol;Acc:HGNC:26564]
ENSG0000	30.78495	20.74207	34.89636	106.9917	70.95167	83.75897	28.8078	87.23412	-1.600516309	1.93E-06	1.70E-05	MB	22	35606764	35637951	-	2168	protein_cod	myoglobin [Source:HGNC Symbol;Acc:HGNC:6915]
ENSG0000	63.62223	35.26153	85.55237	226.514	109.1564	173.7553	61.47871	169.8086	-1.468534646	1.95E-06	1.71E-05	AC126755.2	16	18358086	18379331	-	3030	protein_cod	nuclear pore complex interacting protein family member A5 pseudogene [Source:NCBI gene;Acc:105376752]
ENSG0000	28.73262	45.63256	41.6505	103.1362	109.1564	92.6695	38.67189	101.654	-1.395767097	2.03E-06	1.78E-05	AL449212.1	3	129381298	129394149	+	517	unprocesse	pseudogene similar to intraflagellar transport 122 homolog
ENSG0000	9.235486	15.55656	9.005513	44.33891	49.12039	58.80949	11.26585	50.75626	-2.168978298	2.20E-06	1.92E-05	CFAP52	17	9576627	9643459	+	3078	protein_cod	cilia and flagella associated protein 52 [Source:HGNC Symbol;Acc:HGNC:16053]
ENSG0000	0	4.148415	5.628445	45.30668	33.65656	24.05843	3.258953	33.69667	-3.390254054	2.23E-06	1.95E-05	SAMD3	6	130144315	130365425	-	6903	protein_cod	sterile alpha motif domain containing 3 [Source:HGNC Symbol;Acc:HGNC:21574]
ENSG0000	10.26165	1.037104	6.754134	29.88057	59.12639	40.09738	6.01763	43.03478	-2.839807015	2.25E-06	1.96E-05	AC099487.1	5	102605635	102606197	+	391	lincRNA	novel transcript
ENSG0000	38.99427	45.63256	28.14223	120.4862	84.59622	99.79792	37.58969	101.6268	-1.43025489	2.47E-06	2.14E-05	LINC00243	6	30798654	30830659	-	2171	lincRNA	long intergenic non-protein coding RNA 243 [Source:HGNC Symbol;Acc:HGNC:30956]
ENSG0000	135.4538	138.9719	92.3065	41.44724	49.12039	58.80949	122.2441	49.79237	1.295554595	2.50E-06	2.17E-05	ARHGDIB	12	14942017	14961728	-	2102	protein_cod	Rho GDP dissociation inhibitor beta [Source:HGNC Symbol;Acc:HGNC:679]
ENSG0000	73.88388	76.74567	87.80375	170.6084	137.3552	187.1211	79.47777	165.0282	-1.055650058	2.60E-06	2.25E-05	BCO2	11	112175467	112224699	+	6789	protein_cod	beta-carotene oxygenase 2 [Source:HGNC Symbol;Acc:HGNC:18503]
ENSG0000	2.05233	7.259726	9.005513	57.83336	35.47584	30.2958	6.105856	41.20166	-2.764557027	2.62E-06	2.26E-05	AC010491.1	5	14661808	14664604	-	2797	antisense	novel transcript, antisense to FAM105B
ENSG0000	71.83155	74.67147	58.53583	164.8251	129.1684	147.9148	68.34628	147.3028	-1.105190905	2.62E-06	2.26E-05	PRRT2	16	29811382	29815892	+	3755	protein_cod	proline rich transmembrane protein 2 [Source:HGNC Symbol;Acc:HGNC:30500]
ENSG0000	12.31398	13.48235	5.628445	53.9778	39.11438	56.13633	10.47492	49.74284	-2.238005908	2.70E-06	2.33E-05	AC025031.4	12	46388856	46392126	+	3271	lincRNA	novel transcript
ENSG0000	47.20359	79.85698	59.66152	136.8723	128.2588	162.1716	62.2407	142.4342	-1.194607705	2.95E-06	2.53E-05	AC097376.2	4	139411927	139454034	-	7954	antisense	novel transcript, antisense to RAB33B
ENSG0000	141.6108	124.4524	108.0662	43.37502	56.39748	61.48265	124.7098	53.75172	1.21313754	3.01E-06	2.58E-05	AC092718.4	16	81030770	81031485	+	716	sense_intro	novel transcript
ENSG0000	3.078495	1.037104	2.251378	47.23058	20.01201	22.27632	2.122326	29.83964	-3.811172371	3.08E-06	2.64E-05	HSD3BP5	1	119601340	119609250	+	1132	transcribed	hydroxy-delta-5-steroid dehydrogenase, 3 beta, pseudogene 5 [Source:HGNC Symbol;Acc:HGNC:5223]
ENSG0000	33.86345	33.18732	47.27894	4.819447	9.096368	5.346317	38.1099	6.420711	2.564854798	3.13E-06	2.67E-05	LCTL	15	66547179	66565979	-	3518	protein_cod	lactase like [Source:HGNC Symbol;Acc:HGNC:15583]
ENSG0000	208.3115	200.161	151.968	96.38893	73.68058	105.1442	186.8135	91.73792	1.027389312	3.18E-06	2.71E-05	ZNF136	19	12163067	12189881	+	5425	protein_cod	zinc finger protein 136 [Source:HGNC Symbol;Acc:HGNC:12920]
ENSG0000	65.67456	37.33573	88.92944	137.8362	156.4575	168.409	63.97991	154.2342	-1.274217582	3.30E-06	2.81E-05	CDRT4	17	15436015	15503608	-	3245	protein_cod	CMT1A duplicated region transcript 4 [Source:HGNC Symbol;Acc:HGNC:14383]
ENSG0000	0	0	0	15.42223	14.55419	21.38527	0	17.12056	-6.454134559	3.35E-06	2.85E-05	RNU6-1	15	67839939	67840045	-	107	snRNA	RNA, U6 small nuclear 1 [Source:HGNC Symbol;Acc:HGNC:10227]
ENSG0000	4.10466	4.148415	6.754134	42.41113	30.01801	32.0779	5.002403	34.83568	-2.807592939	3.50E-06	2.96E-05	SELPLG	12	108622277	108633959	-	2494	protein_cod	selectin P ligand [Source:HGNC Symbol;Acc:HGNC:10722]
ENSG0000	2.05233	2.074207	2.251378	31.80835	26.37947	24.05843	2.125972	27.41541	-3.689733329	3.52E-06	2.98E-05	AC063948.1	12	101408372	101409060	-	379	lincRNA	novel transcript
ENSG0000	29.75879	33.18732	9.005513	78.07503	83.68659	85.54107	23.98387	82.43423	-1.772060801	3.54E-06	3.00E-05	SOCS2-AS1	12	93542463	93571768	-	3816	processed_t	SOCS2 antisense RNA 1 [Source:HGNC Symbol;Acc:HGNC:27054]
ENSG0000	20.5233	17.63076	22.51378	84.82226	53.66857	65.04686	20.22262	67.8459	-1.746969645	3.65E-06	3.08E-05	BACH2	6	89926529	90296908	-	9786	protein_cod	BTB domain and CNC homolog 2 [Source:HGNC Symbol;Acc:HGNC:14078]
ENSG0000	21.54947	34.22442	19.13671	91.56949	75.49986	66.82896	24.9702	77.9661	-1.638217089	3.71E-06	3.13E-05	MAPK4	18	50560078	50731824	+	7601	protein_cod	mitogen-activated protein kinase 4 [Source:HGNC Symbol;Acc:HGNC:6878]
ENSG0000	53.36058	44.59546	51.7817	96.38893	119.1624	139.0042	49.91258	118.1852	-1.245164213	3.81E-06	3.21E-05	SAXO2	15	82262810	82284930	+	4160	protein_cod	stabilizer of axonemal microtubules 2 [Source:HGNC Symbol;Acc:HGNC:33727]
ENSG0000	128.2706	131.7122	122.7001	56.86947	42.75293	68.61107	127.561	56.07782	1.185405725	3.85E-06	3.24E-05	TGFB2	1	218346235	218444619	+	5151	protein_cod	transforming growth factor beta 2 [Source:HGNC Symbol;Acc:HGNC:11768]
ENSG0000	14.36631	10.37104	19.13671	64.58058	41.84329	66.82896	14.62469	57.75095	-1.986701373	3.89E-06	3.27E-05	AC142472.1	17	45146730	45148470	-	1741	lincRNA	novel transcript
ENSG0000	53.36058	75.70857	59.66152	141.6917	130.9877	137.2221	62.91022	136.6339	-1.11831311	3.97E-06	3.33E-05	BEAN1	16	66427297	66493529	+	7379	protein_cod	brain expressed associated with NEDD4 1 [Source:HGNC Symbol;Acc:HGNC:24160]
ENSG0000	2.05233	4.148415	2.251378	28.91668	23.65056	34.75106	2.817374	29.1061	-3.364846088	4.11E-06	3.45E-05	AC091173.1	8	61759094	61765969	-	2750	lincRNA	novel transcript
ENSG0000	72.85772	77.78278	75.42117	149.4028	163.7346	143.4595	75.35389	152.199	-1.014269623	4.12E-06	3.45E-05	AC007996.1	18	26542971	26545791	-	2821	sense_intro	novel transcript, sense intronic to KCTD1
ENSG0000	23.6018	14.51945	15.75965	53.9778	64.58421	69.50212	17.9603	62.68805	-1.801344721	4.15E-06	3.48E-05	AC099522.2	5	73497550	73498293	-	744	lincRNA	novel transcript
ENSG0000	49.25592	58.07781	57.41014	138.8001	129.1684	105.1442	54.91462	124.3709	-1.179373096	4.15E-06	3.48E-05	RASGRP1	15	38488103	38565575	-	6097	protein_cod	RAS guanyl releasing protein 1 [Source:HGNC Symbol;Acc:HGNC:9878]
ENSG0000	37.96811	62.22622	83.30099	186.0306	116.4335	151.479	61.16511	151.3144	-1.310456296	4.16E-06	3.48E-05	LINC01772	1	16460948	16468481	+	3245	lincRNA	long intergenic non-protein coding RNA 1772 [Source:HGNC Symbol;Acc:HGNC:52562]
ENSG0000	36.94194	25.92759	16.88534	78.07503	67.31312	106.9263	26.58496	84.10483	-1.65617908	4.19E-06	3.51E-05	AC233723.2	17	4787057	4789162	+	2106	TEC	TEC
ENSG0000	5.130825	6.222622	9.005513	49.15836	27.2891	42.77054	6.78632	39.73933	-2.556658533	4.31E-06	3.60E-05	AC091181.1	17	57277920	57280017	+	2098	TEC	TEC
ENSG0000	51.30825	20.0389	20.26624	72.2917	140.0841	102.4711	33.53652	104.9489	-1.641222119	4.43E-06	3.69E-05	AC078778.1	12	54262615	54279063	+	735	antisense	novel transcript, antisense to CBX5
ENSG0000	46.17743	59.11491	64.16428	117.5945	125.5299	134.549	56.48554	125.8911	-1.158981074	4.45E-06	3.70E-05	AC080112.1	17	40360655	40364693	+	2094	antisense	novel transcript, antisense to GJD3
ENSG0000	59.51757	42.52125	36.02205	133.9806	113.7046	96.23371	46.02029	114.6396	-1.312282403	4.45E-06	3.70E-05	USP32P3	17	20415547	20431008	+	2935	transcribed	ubiquitin specific peptidase 32 pseudogene 3 [Source:HGNC Symbol;Acc:HGNC:43576]

ENSG0000	4.10466	11.40814	6.754134	31.80835	46.39148	46.33475	7.422312	41.51152	-2.484040846	4.52E-06	3.75E-05	NTF3		12	5432112	5521536	+	2919	protein_cod	neurotrophin 3 [Source:HGNC Symbol;Acc:HGNC:8023]
ENSG0000	96.45952	86.07961	94.55788	38.55557	30.01801	40.09738	92.36567	36.22366	1.35054758	4.63E-06	3.83E-05	AL136295.5		14	24151218	24167402	+	4798	protein_cod	novel transcript
ENSG0000	4.10466	0	13.50827	49.15836	31.83729	49.89896	5.870976	43.63153	-2.919677183	4.71E-06	3.89E-05	AC009237.1		2	95537969	95538469	+	501	lincRNA	novel transcript
ENSG0000	49.25592	56.0036	51.7817	116.6306	113.7046	121.1832	52.34707	117.1728	-1.162498473	4.71E-06	3.90E-05	AC005256.1		19	1748056	1748745	+	458	lincRNA	novel transcript
ENSG0000	22.57563	12.44524	16.88534	57.83336	50.93966	79.3037	17.30207	62.69224	-1.85699807	4.77E-06	3.94E-05	EID3		12	104303739	104305205	+	1467	protein_cod	EP300 interacting inhibitor of differentiation 3 [Source:HGNC Symbol;Acc:HGNC:32961]
ENSG0000	0	11.40814	10.1312	41.44724	46.39148	47.2258	7.179781	45.02151	-2.659191738	5.21E-06	4.26E-05	SLC47A1P2		17	19615789	19633825	+	1483	transcribed_	SLC47A1 pseudogene 2 [Source:HGNC Symbol;Acc:HGNC:53866]
ENSG0000	38.99427	43.55836	68.66703	96.38893	133.7166	143.4595	50.40655	124.5217	-1.310747095	5.26E-06	4.30E-05	MUC1		1	155185824	155192916	-	4717	protein_cod	mucin 1, cell surface associated [Source:HGNC Symbol;Acc:HGNC:7508]
ENSG0000	4.10466	23.85338	7.879823	53.9778	55.48785	63.26475	11.94596	57.5768	-2.265096443	5.27E-06	4.31E-05	RENBP	X		153935263	153944691	-	2057	protein_cod	renin binding protein [Source:HGNC Symbol;Acc:HGNC:9959]
ENSG0000	48.22976	45.63256	75.42117	14.45834	13.64455	16.93	56.42783	15.01097	1.906906756	5.39E-06	4.40E-05	LINC01529		19	35785697	35797902	-	4415	transcribed_	long intergenic non-protein coding RNA 1529 [Source:HGNC Symbol;Acc:HGNC:51268]
ENSG0000	3.078495	5.185518	7.879823	32.77224	32.74693	39.20633	5.381279	34.9085	-2.709942911	5.44E-06	4.43E-05	CSNK1A1P1		15	36798596	36818459	-	2629	transcribed_	casein kinase 1 alpha 1 pseudogene 1 [Source:HGNC Symbol;Acc:HGNC:30446]
ENSG0000	28.73262	30.07601	50.65601	98.31671	93.69259	96.23371	36.48821	96.081	-1.402854047	5.51E-06	4.49E-05	PSMB10		16	67934502	67937087	-	1853	protein_cod	proteasome subunit beta 10 [Source:HGNC Symbol;Acc:HGNC:9538]
ENSG0000	104.6688	81.93119	67.54134	34.70002	32.74693	16.03895	84.71379	27.82863	1.611120659	5.53E-06	4.51E-05	LRRC37A9P		17	35912635	35918010	-	5376	processed_	leucine rich repeat containing 37 member A9, pseudogene [Source:HGNC Symbol;Acc:HGNC:43813]
ENSG0000	32.83728	23.85338	16.88534	63.6167	74.59022	90.88739	24.52533	76.36477	-1.634096205	5.55E-06	4.52E-05	LINC01003		7	152463786	152465549	+	1764	lincRNA	long intergenic non-protein coding RNA 1003 [Source:HGNC Symbol;Acc:HGNC:48957]
ENSG0000	21.54947	18.66787	18.01103	67.47225	50.03002	76.63055	19.40945	64.71094	-1.73586801	5.59E-06	4.55E-05	DIAPH2-AS1	X		97431286	97642589	-	2309	antisense	DIAPH2 antisense RNA 1 [Source:HGNC Symbol;Acc:HGNC:16972]
ENSG0000	1.026165	5.185518	2.251378	24.09723	26.37947	37.42422	2.821021	29.30031	-3.374643784	5.63E-06	4.57E-05	AP001462.1		11	64778954	64779405	+	452	lincRNA	novel transcript
ENSG0000	57.46524	46.66967	57.41014	117.5945	115.5239	125.6385	53.84835	119.5856	-1.152111226	5.64E-06	4.58E-05	NPIPB15		16	74377878	74392080	+	1702	protein_cod	nuclear pore complex interacting protein family member B15 [Source:HGNC Symbol;Acc:HGNC:34409]
ENSG0000	57.46524	43.55836	47.27894	144.5834	92.78296	118.51	49.43418	118.6255	-1.260973779	5.67E-06	4.60E-05	AC104534.1		19	38820166	38823223	+	566	antisense	novel transcript, antisense to ECH1
ENSG0000	7.183155	8.29683	9.005513	46.26669	48.21075	30.2958	8.161833	41.59108	-2.350849436	5.79E-06	4.69E-05	AC027601.3		17	81339183	81339691	-	509	lincRNA	novel transcript
ENSG0000	127.2445	72.59726	91.18081	192.7779	184.6563	207.6153	97.00751	195.0165	-1.006447719	5.83E-06	4.72E-05	PMS2P3		7	75502930	75528148	-	1727	transcribed_	PMS1 homolog 2, mismatch repair system component pseudogene 3 [Source:HGNC Symbol;Acc:HGNC:9128]
ENSG0000	34.88961	74.67147	55.15876	121.4501	150.0901	125.6385	54.90661	132.3929	-1.270647194	5.94E-06	4.80E-05	MCF2L2		3	183178043	183428778	-	20568	protein_cod	MCF.2 cell line derived transforming sequence-like 2 [Source:HGNC Symbol;Acc:HGNC:30319]
ENSG0000	9.235486	8.29683	6.754134	36.62779	41.84329	42.77054	8.095483	40.41388	-2.31630961	6.07E-06	4.89E-05	AC010331.1		19	47607873	47608454	-	582	antisense	novel transcript, antisense to GLTSCR1
ENSG0000	9.235486	0	6.754134	44.33891	28.19874	44.55264	5.329873	39.0301	-2.875699725	6.16E-06	4.96E-05	AC125611.2		12	49389516	49390162	+	647	processed_	FGFR1 oncogene partner 2 (FGFR1OP2) pseudogene
ENSG0000	75.93622	85.0425	104.6891	212.0557	166.4635	155.0432	88.55593	177.8541	-1.00739869	6.42E-06	5.16E-05	SYT3		19	50621307	50639827	-	3790	protein_cod	synaptotagmin 3 [Source:HGNC Symbol;Acc:HGNC:11511]
ENSG0000	25.65413	31.11311	41.6505	93.49726	92.78296	77.5216	32.80591	87.93394	-1.426562609	6.58E-06	5.28E-05	AC009403.1		7	155611231	155645205	-	2031	antisense	uncharacterized LOC100506302 [Source:NCBI gene;Acc:100506302]
ENSG0000	5.130825	8.29683	7.879823	36.62779	39.11438	38.31527	7.102493	38.01915	-2.423678466	6.60E-06	5.29E-05	SERPINE3		13	51335773	51364735	+	2608	protein_cod	serpin family E member 3 [Source:HGNC Symbol;Acc:HGNC:24774]
ENSG0000	2.05233	0	1.125689	20.24168	28.19874	27.62264	1.05934	25.35435	-4.579920511	6.70E-06	5.37E-05	MLIP		6	53929982	54266280	+	7293	protein_cod	muscular LMNA interacting protein [Source:HGNC Symbol;Acc:HGNC:21355]
ENSG0000	6.15699	1.037104	1.125689	18.3139	31.83729	41.87948	2.773261	30.67689	-3.450699489	6.74E-06	5.39E-05	AC099811.1		17	42270517	42272683	-	1496	sense_intro	novel transcript, sense intronic to STAT5B
ENSG0000	15.39248	36.29863	49.53032	110.8473	83.68659	106.0353	33.74047	100.1897	-1.576651036	7.02E-06	5.59E-05	ZNF546		19	39984134	40021041	+	9007	protein_cod	zinc finger protein 546 [Source:HGNC Symbol;Acc:HGNC:28671]
ENSG0000	1709.591	1562.915	1338.444	555.2002	915.0946	815.3134	1536.984	761.8694	1.012320783	7.08E-06	5.63E-05	CAV2		7	116287380	116508541	+	6926	protein_cod	caveolin 2 [Source:HGNC Symbol;Acc:HGNC:1528]
ENSG0000	1.026165	1.037104	1.125689	16.38612	36.38547	24.05843	1.062986	25.61001	-4.593775808	7.14E-06	5.68E-05	AC104794.3		2	10021578	10022825	+	1248	lincRNA	novel transcript
ENSG0000	11.28782	21.77918	41.6505	0.963889	1.819274	0.891053	24.90583	1.224739	4.339679152	7.24E-06	5.76E-05	FPR1		19	51745172	51804110	-	2594	protein_cod	formyl peptide receptor 1 [Source:HGNC Symbol;Acc:HGNC:3826]
ENSG0000	138.5323	107.8588	141.8368	53.01391	60.94567	65.04686	129.4093	59.66881	1.114911692	7.35E-06	5.83E-05	AC022211.1		17	75145261	75146546	-	717	sense_intro	novel transcript, sense intronic to HN1
ENSG0000	1.026165	2.074207	1.125689	28.91668	15.46383	32.0779	1.408687	25.48614	-4.172204133	7.54E-06	5.97E-05	LSP1P4		2	91617160	91659972	-	3057	transcribed_	LSP1 pseudogene 4 [Source:HGNC Symbol;Acc:HGNC:53915]
ENSG0000	8.209321	6.222622	15.75965	47.23058	37.29511	54.35422	10.06386	46.2933	-2.213078671	7.84E-06	6.20E-05	PRKN		6	161347420	162727771	-	5686	protein_cod	parkin RBR E3 ubiquitin protein ligase [Source:HGNC Symbol;Acc:HGNC:8607]
ENSG0000	140.5846	155.5656	128.3286	64.58058	70.04203	73.95739	141.4929	69.52667	1.025026307	7.87E-06	6.22E-05	EPHX4		1	92029982	92063536	+	2251	protein_cod	epoxide hydrolase 4 [Source:HGNC Symbol;Acc:HGNC:23758]
ENSG0000	30.78495	28.0018	16.88534	76.14726	86.4155	64.15581	25.22403	75.57285	-1.577135892	8.17E-06	6.43E-05	DNAJC27-AS		2	24971390	25039694	+	5651	antisense	DNAJC27 antisense RNA 1 [Source:HGNC Symbol;Acc:HGNC:42943]
ENSG0000	23.6018	18.66787	19.13671	56.86947	70.95167	65.04686	20.46879	64.28933	-1.650280357	8.35E-06	6.57E-05	GPNMB		7	23235967	23275108	+	6040	protein_cod	glycoprotein nmb [Source:HGNC Symbol;Acc:HGNC:4462]
ENSG0000	1.026165	3.111311	9.005513	31.80835	35.47584	32.0779	4.380996	33.1207	-2.945082803	8.75E-06	6.86E-05	AP000845.1		18	268148	270278	+	2131	lincRNA	novel transcript
ENSG0000	16.41864	22.81628	36.02205	0	2.72891	2.673159	25.08566	1.80069	3.775859841	9.28E-06	7.25E-05	FLG-AS1		1	152168125	152445456	+	7691	antisense	FLG antisense RNA 1 [Source:HGNC Symbol;Acc:HGNC:27913]
ENSG0000	2.05233	0	3.377067	31.80835	21.83128	23.16737	1.809799	25.60234	-3.845549062	9.38E-06	7.32E-05	AC024257.3		12	48198357	48230275	+	6938	antisense	novel transcript, antisense to OR10AD1
ENSG0000	4.10466	2.074207	6.754134	25.06112	50.93966	24.05843	4.311001	33.35307	-2.965260045	9.40E-06	7.32E-05	TSC22D1-AS		13	44575893	44580432	+	3457	antisense	TSC22D1 antisense RNA 1 [Source:HGNC Symbol;Acc:HGNC:43684]
ENSG0000	2.05233	3.111311	5.628445	26.02501	22.74092	41.87948	3.597362	30.21514	-3.08643434	9.51E-06	7.40E-05	STK24-AS1		13	98577244	98578830	+	1278	antisense	STK24 antisense RNA 1 [Source:HGNC Symbol;Acc:HGNC:39935]
ENSG0000	52.33442	43.55836	46.15325	117.5945	83.68659	140.7864	47.34868	114.0225	-1.267623629	9.65E-06	7.50E-05	AC011476.3		19	55006193	55048086	+	1374	antisense	novel transcript, antisense to RDH13 and GP6
ENSG0000	6.15699	11.40814	5.628445	35.6639	52.75894	32.96896	7.731192	40.46393	-2.383139033	9.76E-06	7.59E-05	AC087257.2		12	27592461	27594039	-	928	unprocesse	chromosome 12 open reading frame 71 (C12orf71) pseudogene
ENSG0000	16.41864	22.81628	21.38809	61.68892	60.03603	67.72002	20.20767	63.14832	-1.645527943	9.97E-06	7.74E-05	AC012236.1		15	31221999	31230838	-	1563	lincRNA	uncharacterized LOC283710 [Source:NCBI gene;Acc:283710]
ENSG0000	161.1079	135.8606	119.323	71.32781	66.40349	64.15581	138.7638	67.2957	1.046021965	1.02E-05	7.87E-05	SLC37A2		11	125063067	125090312	+	6371	protein_cod	solute carrier family 37 member 2 [Source:HGNC Symbol;Acc:HGNC:20644]
ENSG0000	172.3957	84.0054	41.6505	33.73613	19.10237	28.51369	99.35054	27.1174	1.87723707	1.04E-05	8.02E-05	CNTNAP3C		9	61330717	61453030	+	1728	protein_cod	contactin associated protein like 3C [Source:HGNC Symbol;Acc:HGNC:53878]
ENSG0000	8.209321	1.037104	5.628445	19.27779	42.75293	48.11685	4.95829	36.71586	-2.891443753	1.04E-05	8.04E-05	SUCNR1		3	151873643	151884619	+	4181	protein_cod	succinate receptor 1 [Source:HGNC Symbol;Acc:HGNC:4542]
ENSG0000	15.39248	14.51945	5.628445	45.3028	57.30712	46.33475	11.84679	49.64822	-2.057264001	1.05E-05	8.09E-05	AC097478.4		4	89747802	89755853	+	522	antisense	novel transcript, antisense to SNCA
ENSG0000	15.39248	11.40814	19.13671	66.50836	36.38547	71.28423	15.31244	58.05935	-1.926719149	1.05E-05	8.14E-05	CYP27A1		2	218781749	218815293	+	2983	protein_cod	cytochrome P450 family 27 subfamily A member 1 [Source:HGNC Symbol;Acc:HGNC:2605]
ENSG0000	23.6018	34.22442	47.27894	53.9778	136.4455	133.6579	35.03505	108.0271	-1.630428131	1.05E-05	8.14E-05	AC120053.1		8	93715378	93716113	-	736	antisense	novel transcript, antisense to FAM92A1
ENSG0000	27.70646	57.0407	42.77618	105.0639	97.33114	116.7279	42.50778	106.3743	-1.324233577	1.06E-05	8.21E-05	AL117339.4		10	38137337	38144399	+	7063	lincRNA	novel transcript
ENSG0000	43.09893	33.18732	36.02205	80.00281	96.4215	102.4711	37.4361	92.96513	-1.312274407	1.08E-05	8.33E-05	EXOC3-AS1		5	441498	443160	-	1663	antisense	EXOC3 antisense RNA 1 [Source:HGNC Symbol;Acc:HGNC:25175]
ENSG0000	5.130825	5.185518	6.754134	31.80835	30.92765	38.31527	5.690159	33.68376	-2.570504462	1.10E-05	8.43E-05	AC069224.1		3	160753428	160755142	-	1715	antisense	novel transcript, antisense to PPM1L
ENSG0000	13.34015	14.51945	7.879823	47.23058	40.93366	57.02738	11.91314	48.39721	-2.01614275	1.12E-05	8.63E-05	AL160314.2		14	22701476	22766562	-	2513	processed_t	novel transcript, antisense to OXA1L
ENSG0000	49.25592	29.0389	55.15876	103.1362	95.51187	130.9848	44.48453	109.8776	-1.308039899	1.18E-05	9.03E-05	AC092171.3		7	5426277	5428927	-	2651	lincRNA	uncharacterized LOC100129484 [Source:NCBI gene;Acc:100129484]
ENSG0000	26.68029	22.81628	24.76516	63.6167	71.86131	75.73949	24.75391	70.40583	-1.508517635	1.19E-05	9.06E-05	AC129492.1		17	8079482	8081565	+	1804	antisense	uncharacterized LOC107985075 [Source:NCBI gene;Acc:107985075]
ENSG0000	0	0	0	3.855557	30.92765	15.1479	0	16.6437	-6.414706624	1.19E-05	9.09E-05	RN7SL316P		17	30702504	30702775	-	272	misc_RNA	RNA, 7SL, cytoplasmic 316, pseudogene [Source:HGNC Symbol;Acc:HGNC:46332]
ENSG0000	1.026165	0	1.125689	21.20557	24.56019	29.40474	0.717285	25.05683	-5.144717265	1.21E-05	9.18E-05	AP002981.1		8	102417979	102418953	+	975	processed_	heat shock 10kDa protein 1 (chaperonin 10) (HSPE1) pseudogene
ENSG0000	29.75879	36.29863	25.89085	78.07503	77.31913	87.32318	30.64942	80.90578	-1.398279785	1.24E-05	9.43E-05	AFAP1L2		10	114294824	114404756	-	5789	protein_cod	actin filament associated protein 1 like 2 [Source:HGNC Symbol;Acc:HGNC:25901]
ENSG0000	8.209321	5.185518	15.75965	38.55557	39.11438	57.91844	9.718162	45.19613	-2.230416915	1.30E-05	9.83E-05	Z97832.2		6	35220370	35224630	-	4261	lincRNA	novel transcript, antisense to SCUBE3
ENSG0000	22.57563	28.0018	15.75965	70.36392	70.95167	60.59159	22.11236	67.3024	-1.600623296	1.30E-05	9.83E-05	TBC1D10C		11	67403915	67410089	+	4993	protein_cod	TBC1 domain family member 10C [Source:HGNC Symbol;Acc:HGNC:24702]
ENSG0000	55.41291	79.85698	76.54686	165.789	148.2708	125.6385	70.60558	146.5661	-1.054280846	1.31E-05	9.92E-05	BDNF-AS		11	27506838	27698174	+	3726	antisense	BDNF antisense RNA [Source:HGNC Symbol;Acc:HGNC:20608]
ENSG0000	56.43908	60.15201	38.27343	150.3667	107.3371	104.2532	51.62151	120.6524	-1.220055452	1.31E-05	9.93E-05	FRAT2		10	97332497	97334709	-	2213	protein_cod	FRAT2, WNT signaling pathway regulator [Source:HGNC Symbol;Acc:HGNC:16048]
ENSG0000	35.91578	44.59546	66.41565	133.0167	111.8853	102.4711	48.97563	115.791	-1.245688174	1.35E-05	0.000102	LINC01312		6	133821147	133851800	+	1364	lincRNA	long intergenic non-protein coding RNA 1312 [Source:HGNC Symbol;Acc:HGNC:28525]
ENSG0000	6.15699	9.333933	9.005513	50.12224	39.11438	30.2958	8.165479	39.84414	-2.287968289	1.39E-05	0.000105	RF02247		2	44934055	44934271	+	217	misc_RNA	
ENSG0000	68.75306	92.30223	49.53032	24.09723	26.37947	17.82106	70.1952	22.76592	1.627817367	1.43E-05	0.000108	SPP1		4	87975650	87983426	+	2321	protein_cod	secreted phosphoprotein 1 [Source:HGNC Symbol;Acc:HGNC:11255]
ENSG0000	6.15699	1.037104	0	26.9889	21.83128	34.75106	2.398031	27.85708	-3.508329492	1.44E-05	0.000108	FLJ42393		3	188178543	188180812	+	2270	TEC	uncharacterized LOC401105 [Source:NCBI gene;Acc:401105]
ENSG0000	31.81112	36.29863	38.27343	117.5945	65.49385	96.23371	35.46106	93.10735	-1.392870346	1.46E-05	0.00011	AC124319.2		17	80351828	80415168	-	3983	antisense	uncharacterized LOC100294362 [Source:NCBI gene;Acc:100294362]

ENSG0000	95.43335	97.48775	90.05513	32.77224	44.5722	41.87948	94.32541	39.74131	1.245477218	1.48E-05	0.000111	CORO1A	16	30182827	30189076	+	4871	protein_cod	coronin 1A [Source:HGNC Symbol;Acc:HGNC:2252]
ENSG0000	8.209321	20.74207	16.88534	64.58058	50.93966	51.68107	15.27891	55.73377	-1.869034159	1.48E-05	0.000111	KRTAP2-5P	17	41071946	41072341	+	396	unprocesse	keratin associated protein 2-5, pseudogene [Source:HGNC Symbol;Acc:HGNC:18920]
ENSG0000	49.25592	40.44704	49.53032	114.7028	117.3431	88.21423	46.4111	106.7534	-1.201922827	1.49E-05	0.000112	AL031282.2	1	1702736	1737688	-	5079	processed_t	novel transcript, readthrough between SLC35E2 and CDK11A
ENSG0000	148.7939	122.3782	133.957	53.9778	52.75894	81.97686	135.0431	62.90453	1.100302387	1.54E-05	0.000115	GAPDHP1	X	39787132	39788136	-	1005	processed_p	glyceraldehyde-3-phosphate dehydrogenase pseudogene 1 [Source:HGNC Symbol;Acc:HGNC:4159]
ENSG0000	10.26165	15.55656	15.75965	56.86947	42.75293	52.57212	13.85928	50.73151	-1.874635867	1.63E-05	0.000121	AC009120.1	16	74312609	74313390	-	473	processed_p	ribosomal protein L21 (RPL21) pseudogene
ENSG0000	18.47097	28.0018	24.76516	91.56949	58.21676	63.26475	23.74598	71.017	-1.580126899	1.70E-05	0.000126	TRIM17	1	228407940	228416861	-	3548	protein_cod	tripartite motif containing 17 [Source:HGNC Symbol;Acc:HGNC:13430]
ENSG0000	29.75879	38.37284	36.02205	60.72503	112.795	104.2532	34.71789	92.59106	-1.417550579	1.73E-05	0.000128	SLC9C1	3	112140887	112294258	-	4250	protein_cod	solute carrier family 9 member C1 [Source:HGNC Symbol;Acc:HGNC:31401]
ENSG0000	10.26165	5.185518	7.879823	29.88057	33.65656	55.24528	7.775664	39.59414	-2.349271012	1.74E-05	0.000129	AC087257.1	12	27547050	27552773	-	2639	lincRNA	novel transcript
ENSG0000	0	0	0	15.42223	11.82528	14.25685	0	13.83478	-6.145645461	1.75E-05	0.00013	TRPC6	11	101451564	101872562	-	5537	protein_cod	transient receptor potential cation channel subfamily C member 6 [Source:HGNC Symbol;Acc:HGNC:12338]
ENSG0000	42.07277	70.52305	59.66152	126.2695	134.6262	114.9458	57.41911	125.2805	-1.12635973	1.76E-05	0.00013	PGBD4	15	34102073	34108684	+	6612	protein_cod	piggyBac transposable element derived 4 [Source:HGNC Symbol;Acc:HGNC:19401]
ENSG0000	6.15699	7.259726	1.125689	45.3028	25.46983	29.40474	4.847468	33.39246	-2.765445995	1.78E-05	0.000132	FGF1	5	142592178	142698070	-	5275	protein_cod	fibroblast growth factor 1 [Source:HGNC Symbol;Acc:HGNC:3665]
ENSG0000	4.10466	10.37104	10.1312	32.77224	40.02402	46.33475	8.2023	39.71034	-2.282401864	1.79E-05	0.000132	KLF3-AS1	4	38612701	38664883	-	4830	processed_t	KLF3 antisense RNA 1 [Source:HGNC Symbol;Acc:HGNC:25796]
ENSG0000	53.36058	52.89229	41.6505	99.2806	121.8913	107.8174	49.30112	109.6631	-1.15144062	1.82E-05	0.000134	STARD7-AS1	2	96208416	96242621	+	2349	processed_t	STARD7 antisense RNA 1 [Source:HGNC Symbol;Acc:HGNC:40827]
ENSG0000	13.34015	12.44524	10.1312	37.59168	71.86131	38.31527	11.9722	49.25609	-2.038302353	1.86E-05	0.000137	RPL9P29	17	78845953	78846868	-	593	processed_p	ribosomal protein L9 pseudogene 29 [Source:HGNC Symbol;Acc:HGNC:35533]
ENSG0000	47.20359	36.29863	47.27894	15.42223	9.096368	6.23737	43.59372	10.25199	2.095602445	1.87E-05	0.000138	KRT5	12	52514575	52520687	-	4292	protein_cod	keratin 5 [Source:HGNC Symbol;Acc:HGNC:6442]
ENSG0000	25.65413	22.81628	14.63396	0	0.909637	1.782106	21.03479	0.897248	4.524080836	1.91E-05	0.00014	MMP13	11	102942995	102955734	-	2834	protein_cod	matrix metallopeptidase 13 [Source:HGNC Symbol;Acc:HGNC:7159]
ENSG0000	129.2968	142.0832	157.5965	59.76114	82.77695	71.28423	142.9922	71.27411	1.002188748	1.92E-05	0.000141	HELLPAR	12	102197585	102402596	+	205012	macro_lncR	HELLP associated long non-coding RNA [Source:HGNC Symbol;Acc:HGNC:43984]
ENSG0000	2.05233	3.111311	1.125689	28.91668	26.37947	17.82106	2.096443	24.3724	-3.525627015	1.95E-05	0.000143	PLA2R1	2	159932006	160062610	-	15357	protein_cod	phospholipase A2 receptor 1 [Source:HGNC Symbol;Acc:HGNC:9042]
ENSG0000	16.41864	20.74207	15.75965	48.19447	70.95167	54.35422	17.64012	57.83345	-1.712060176	1.96E-05	0.000144	AC138904.1	16	28284885	28292064	-	2481	lincRNA	novel transcript LOC100506705
ENSG0000	52.33442	48.74387	61.9129	117.5945	116.4335	112.2727	54.3304	115.4336	-1.089196008	2.00E-05	0.000146	SGMS1-AS1	10	50624951	50641451	+	12420	antisense	SGMS1 antisense RNA 1 [Source:HGNC Symbol;Acc:HGNC:49683]
ENSG0000	34.88961	23.85338	36.02205	60.72503	99.15041	96.23371	31.58835	85.36972	-1.437580964	2.01E-05	0.000146	AC008537.3	19	40840159	40842039	+	1881	TEC	TEC
ENSG0000	50.28209	58.07781	49.53032	111.8112	121.8913	105.1442	52.63007	112.9489	-1.100852859	2.10E-05	0.000152	AC006449.6	17	38702452	38704747	+	2296	lincRNA	novel transcript
ENSG0000	119.0351	129.638	94.55788	27.95279	58.21676	55.24528	114.4103	47.13827	1.27670676	2.11E-05	0.000153	HMGN5	X	81113701	81201942	-	2416	protein_cod	high mobility group nucleosome binding domain 5 [Source:HGNC Symbol;Acc:HGNC:8013]
ENSG0000	28.73262	15.55656	27.01654	85.78615	59.12639	66.82896	23.76857	70.5805	-1.570875426	2.13E-05	0.000155	FAM186B	12	49582885	49605639	-	4756	protein_cod	family with sequence similarity 186 member B [Source:HGNC Symbol;Acc:HGNC:25296]
ENSG0000	46.17743	46.66967	55.15876	115.6667	97.33114	111.3816	49.33529	108.1265	-1.13357565	2.14E-05	0.000155	ALOX12	17	6996065	7010736	+	2616	protein_cod	arachidonate 12-lipoxygenase, 12S type [Source:HGNC Symbol;Acc:HGNC:429]
ENSG0000	6.15699	5.185518	6.754134	31.80835	27.2891	40.98843	6.032214	33.36196	-2.470563662	2.24E-05	0.000162	COL20A1	20	63293186	63334851	+	10974	protein_cod	collagen type XX alpha 1 chain [Source:HGNC Symbol;Acc:HGNC:14670]
ENSG0000	18.47097	9.333933	13.50827	56.86947	57.30712	39.20633	13.77106	51.12764	-1.89049609	2.28E-05	0.000165	AL137077.2	20	62066830	62068437	+	1608	lincRNA	novel transcript
ENSG0000	51.30825	58.07781	76.54686	10.60278	23.65056	22.27632	61.97764	18.84322	1.710862273	2.29E-05	0.000165	KIF7	15	89608789	89655451	-	5001	protein_cod	kinesin family member 7 [Source:HGNC Symbol;Acc:HGNC:30497]
ENSG0000	2.05233	9.333933	7.879823	37.59168	33.65656	34.75106	6.422029	35.3331	-2.466167376	2.43E-05	0.000175	RTCA-AS1	1	100251528	100266179	-	2776	lincRNA	RTCA antisense RNA 1 [Source:HGNC Symbol;Acc:HGNC:50573]
ENSG0000	112.8782	85.0425	90.05513	34.70002	52.75894	23.16737	95.99193	36.87544	1.381373599	2.44E-05	0.000175	GPR39	2	132416574	132646559	+	3387	protein_cod	G protein-coupled receptor 39 [Source:HGNC Symbol;Acc:HGNC:4496]
ENSG0000	113.9043	87.11671	108.0662	47.23058	33.65656	52.57212	103.0291	44.48642	1.211325044	2.48E-05	0.000178	NXF3	X	103075810	103093125	-	3526	protein_cod	nuclear RNA export factor 3 [Source:HGNC Symbol;Acc:HGNC:8073]
ENSG0000	1.026165	8.29683	4.502756	47.23058	29.10838	22.27632	4.608584	32.87176	-2.834180145	2.55E-05	0.000183	AC073107.2	7	65764535	65768306	+	238	unprocesse	sulfatase modifying factor 2 (SUMF2) pseudogene
ENSG0000	13.34015	14.51945	19.13671	49.15836	48.21075	60.59159	15.66544	52.65357	-1.753831641	2.58E-05	0.000185	LINC00869	1	149606334	149679523	+	8611	transcribed_	long intergenic non-protein coding RNA 869 [Source:HGNC Symbol;Acc:HGNC:29050]
ENSG0000	135.4538	174.2334	136.2084	93.49726	67.31312	50.79001	148.6319	70.53347	1.078912282	2.65E-05	0.000189	MILR1	17	64449037	64468643	+	1816	protein_cod	mast cell immunoglobulin like receptor 1 [Source:HGNC Symbol;Acc:HGNC:27570]
ENSG0000	26.68029	61.18912	51.7817	96.38893	112.795	130.9848	46.55037	113.3896	-1.287209896	2.67E-05	0.00019	AC018809.2	3	9958717	9962539	+	3823	antisense	novel transcript, antisense to TMEM111
ENSG0000	2.05233	2.074207	4.502756	26.02501	26.37947	23.16737	2.876431	25.19062	-3.145744145	2.69E-05	0.000191	AC099521.2	16	68367325	68370262	-	2938	TEC	novel transcript
ENSG0000	4.10466	12.44524	7.879823	33.73613	43.66257	39.20633	8.143243	38.86834	-2.256128013	2.69E-05	0.000191	AC087392.3	17	763565	765319	-	1755	lincRNA	novel transcript
ENSG0000	112.8782	110.9701	93.43219	57.83336	29.10838	47.2258	105.7601	44.72251	1.244887685	2.71E-05	0.000193	DOK3	5	177501907	177511274	-	4823	protein_cod	docking protein 3 [Source:HGNC Symbol;Acc:HGNC:24583]
ENSG0000	114.9305	141.0461	160.9735	80.00281	56.39748	67.72002	138.9834	68.0401	1.030555076	2.91E-05	0.000206	LINC01814	2	8506446	8592903	-	12173	lincRNA	long intergenic non-protein coding RNA 1814 [Source:HGNC Symbol;Acc:HGNC:52618]
ENSG0000	32.83728	23.85338	31.51929	86.75004	65.49385	78.42255	29.40332	76.88551	-1.387015298	2.92E-05	0.000206	CTC-338M1:	5	181246523	181272167	+	3312	processed_t	uncharacterized LOC101928649 [Source:NCBI gene;Acc:101928649]
ENSG0000	25.65413	42.52125	21.38809	74.21948	95.51187	73.95739	29.85449	81.22958	-1.4404822	2.93E-05	0.000207	KLRD1	12	10226058	10329600	+	17404	protein_cod	killer cell lectin like receptor D1 [Source:HGNC Symbol;Acc:HGNC:6378]
ENSG0000	2.05233	15.55656	1.125689	31.80835	42.75293	48.11685	6.244858	40.89271	-2.698918444	3.00E-05	0.000212	AC133528.1	2	241581922	241582726	+	805	lincRNA	novel transcript
ENSG0000	7.183155	6.222622	5.628445	40.48335	34.5662	25.84053	6.344741	33.63003	-2.402049831	3.01E-05	0.000212	AC019294.2	15	75737820	75763321	+	2489	processed_t	uncharacterized LOC101929408 [Source:NCBI gene;Acc:101929408]
ENSG0000	5.130825	8.29683	11.25689	52.05002	33.65656	31.18685	8.228182	38.96448	-2.24992937	3.01E-05	0.000212	AC024940.3	12	31311617	31312029	+	316	processed_p	solute carrier family 25, member 13 (citrin) (SLC25A13) pseudogene
ENSG0000	1.026165	12.44524	4.502756	32.77224	35.47584	40.98843	5.991389	36.41217	-2.601196715	3.08E-05	0.000217	IMPDH1P8	3	15878047	15879571	+	1525	processed_p	inosine monophosphate dehydrogenase 1 pseudogene 8 [Source:HGNC Symbol;Acc:HGNC:33963]
ENSG0000	50.28209	85.0425	95.68357	183.139	127.3492	176.4285	77.00272	162.3055	-1.078385212	3.08E-05	0.000217	ACAP1	17	7336529	7351478	+	7421	protein_cod	ArfGAP with coiled-coil, ankyrin repeat and PH domains 1 [Source:HGNC Symbol;Acc:HGNC:16467]
ENSG0000	27.70646	25.92759	18.01103	70.36392	92.78296	49.89896	23.88169	71.01528	-1.567869747	3.15E-05	0.000221	FAM111B	11	59107185	59127410	+	3863	protein_cod	family with sequence similarity 111 member B [Source:HGNC Symbol;Acc:HGNC:24200]
ENSG0000	72.85772	91.26512	64.16428	31.80835	26.37947	30.2958	76.09571	29.49454	1.369270947	3.20E-05	0.000225	SLC7A11-AS	4	138057464	138178177	+	6161	processed_t	SLC7A11 antisense RNA 1 [Source:HGNC Symbol;Acc:HGNC:44064]
ENSG0000	1.026165	7.259726	9.005513	35.6639	23.65056	46.33475	5.763801	35.2164	-2.624910896	3.21E-05	0.000225	KCNT1	9	135702185	135795508	+	12183	protein_cod	potassium sodium-activated channel subfamily T member 1 [Source:HGNC Symbol;Acc:HGNC:18865]
ENSG0000	103.6427	74.67147	79.92392	109.8834	255.6079	203.1601	86.07935	189.5505	-1.139228881	3.23E-05	0.000226	AC107308.1	12	65830750	65831050	+	301	sense_intro	novel transcript, sense intronic to HMGA2
ENSG0000	93.38102	123.4153	112.5689	41.44724	58.21676	50.79001	109.7884	50.15134	1.12842442	3.23E-05	0.000227	LANCL3	X	37571569	37684463	+	10298	protein_cod	LanC like 3 [Source:HGNC Symbol;Acc:HGNC:24767]
ENSG0000	2.05233	2.074207	1.125689	31.80835	19.10237	16.93	1.750742	22.61358	-3.678649696	3.36E-05	0.000235	UNC93B2	7	6855485	6859830	-	730	processed_p	unc-93 homolog B2, pseudogene [Source:HGNC Symbol;Acc:HGNC:13482]
ENSG0000	64.6484	38.37284	66.41565	120.4862	111.8853	135.44	56.47896	122.6038	-1.120478236	3.40E-05	0.000238	CYP27B1	12	57762334	57768986	-	3440	protein_cod	cytochrome P450 family 27 subfamily B member 1 [Source:HGNC Symbol;Acc:HGNC:2606]
ENSG0000	42.07277	26.9647	33.77067	74.21948	89.14441	90.88739	34.26938	84.75043	-1.306387191	3.44E-05	0.00024	TMEM91	19	41350911	41384083	+	2796	protein_cod	transmembrane protein 91 [Source:HGNC Symbol;Acc:HGNC:32393]
ENSG0000	37.96811	40.44704	43.90187	133.9806	62.76494	112.2727	40.77234	103.0061	-1.337101905	3.50E-05	0.000244	AC006504.1	19	27757184	27760849	-	3666	lincRNA	novel transcript
ENSG0000	57.46524	50.81808	31.51929	95.42504	119.1624	106.9263	46.60087	107.1713	-1.197270546	3.50E-05	0.000244	INCA1	17	4988130	4997610	-	1553	protein_cod	inhibitor of CDK, cyclin A1 interacting protein 1 [Source:HGNC Symbol;Acc:HGNC:32224]
ENSG0000	11.28782	6.222622	7.879823	24.09723	50.03002	45.4437	8.46342	39.85698	-2.235215627	3.55E-05	0.000248	AC138150.1	17	45150400	45161510	-	696	antisense	novel transcript, antisense to HEXIM1 and HEXIM2
ENSG0000	50.28209	74.67147	64.16428	151.3306	144.6323	103.3621	63.03928	133.1083	-1.077869508	3.67E-05	0.000255	COL5A2	2	189031896	189225312	-	7453	protein_cod	collagen type V alpha 2 chain [Source:HGNC Symbol;Acc:HGNC:2210]
ENSG0000	15.39248	25.92759	19.13671	65.54447	53.66857	62.3737	20.15226	60.52892	-1.585995912	3.68E-05	0.000255	DBH-AS1	9	133654586	133657313	-	2139	antisense	DBH antisense RNA 1 [Source:HGNC Symbol;Acc:HGNC:24155]
ENSG0000	8.209321	9.333933	6.754134	28.91668	47.30111	35.64211	8.099129	37.28664	-2.200431478	3.76E-05	0.000261	AC011476.2	19	55027593	55053928	-	1271	processed_t	novel transcript
ENSG0000	44.1251	58.07781	56.28445	103.1362	110.9757	121.1832	52.82912	111.765	-1.082861679	3.82E-05	0.000265	ANKRD24	19	4183354	4224814	+	4474	protein_cod	ankyrin repeat domain 24 [Source:HGNC Symbol;Acc:HGNC:29424]
ENSG0000	153.9248	163.8624	115.946	51.08613	82.77695	76.63055	144.5777	70.16454	1.042145429	3.91E-05	0.00027	FANCB	X	14835961	14873069	-	3593	protein_cod	FA complementation group B [Source:HGNC Symbol;Acc:HGNC:3583]
ENSG0000	116.9828	99.56195	119.323	50.12224	63.67458	42.77054	111.9559	52.18912	1.100843387	3.99E-05	0.000275	RCBTB2	13	48488959	48533256	-	3545	protein_cod	RCC1 and BTB domain containing protein 2 [Source:HGNC Symbol;Acc:HGNC:1914]
ENSG0000	8.209321	15.55656	11.25689	43.37502	32.74693	63.26475	11.67426	46.46223	-1.993568739	4.00E-05	0.000276	ADGRF3	2	26308173	26346817	-	5761	protein_cod	adhesion G protein-coupled receptor F3 [Source:HGNC Symbol;Acc:HGNC:18989]
ENSG0000	118.009	92.30223	81.04961	41.44724	44.5722	42.77054	97.12027	42.92999	1.179020009	4.11E-05	0.000283	PRR22	19	5782960	5784765	-	1806	protein_cod	proline rich 22 [Source:HGNC Symbol;Acc:HGNC:28354]
ENSG0000	50.28209	56.0036	59.66152	122.4139	106.4275	113.1637	55.31574	114.0017	-1.044182624	4.24E-05	0.000291	AC026471.1	16	31456711	31459736	-	3026	antisense	novel transcript, antisense to ARMC5
ENSG0000	10.26165	11.40814	18.01103	85.78615	26.37947	50.79001	13.22694	54.31854	-2.041911616	4.38E-05	0.0003	AC092902.5	3	125766516	125852936	+	4236	processed_t	novel transcript
ENSG0000	14.36631	13.48235	21.38809	42.41113	52.75894	66.82896	16.41225	53.99968	-1.724925142	4.40E-05	0.000302	FOXC2	16	86566829	86569728	+	2900	protein_cod	forkhead box C2 [Source:HGNC Symbol;Acc:HGNC:3801]
ENSG0000	5.130825	2.074207	0	16.38612	29.10838	29.40474	2.401678	24.96641	-3.351028755	4.41E-05	0.000302	AC239584.1	4	74552584	74589481	-	732	antisense	novel transcript

ENSG0000	19.49714	12.44524	16.88534	45.3028	46.39148	70.39318	16.27591	54.02915	-1.732412806	4.44E-05	0.000303	TOB1-AS1	17	50866679	50909737	+	2149	processed_t	TOB1 antisense RNA 1 [Source:HGNC Symbol;Acc:HGNC:44340]
ENSG0000	16.41864	19.70497	25.89085	0	0	3.564211	20.67149	1.18807	4.086169696	4.45E-05	0.000304	PRSS33	16	2783953	2787948	-	2112	protein_cod	serine protease 33 [Source:HGNC Symbol;Acc:HGNC:30405]
ENSG0000	27.70646	14.51945	22.51378	78.07503	45.48184	73.95739	21.5799	65.83809	-1.608741393	4.52E-05	0.000308	AC084346.2	8	93915734	93916682	-	949	lincRNA	novel transcript
ENSG0000	57.46524	51.85518	46.15325	21.20557	8.186731	16.03895	51.82456	15.14375	1.779885995	4.53E-05	0.000309	GPR19	12	12660891	12696207	-	2623	protein_cod	G protein-coupled receptor 19 [Source:HGNC Symbol;Acc:HGNC:4473]
ENSG0000	65.67456	62.22622	43.90187	114.7028	97.33114	174.6464	57.26755	128.8934	-1.168413367	4.56E-05	0.00031	AC131212.3	12	132550729	132554947	+	4219	TEC	novel transcript
ENSG0000	0	0	0	8.675004	13.64455	14.25685	0	12.19213	-5.964934405	4.78E-05	0.000324	NTS	12	85874295	85882992	+	1555	protein_cod	neurotensin [Source:HGNC Symbol;Acc:HGNC:8038]
ENSG0000	128.2706	97.48775	93.43219	57.83336	44.5722	43.66159	106.3969	48.68905	1.130906602	4.78E-05	0.000324	HSPA2	14	64535905	64546173	+	5777	protein_cod	heat shock protein family A (Hsp70) member 2 [Source:HGNC Symbol;Acc:HGNC:5235]
ENSG0000	32.83728	16.59366	27.01654	86.75004	56.39748	72.17528	25.48249	71.77427	-1.493404717	4.98E-05	0.000336	CDHR2	5	176542511	176595974	+	5348	protein_cod	cadherin related family member 2 [Source:HGNC Symbol;Acc:HGNC:18231]
ENSG0000	4.10466	8.29683	7.879823	43.37502	35.47584	24.05843	6.760438	34.30309	-2.345951295	5.08E-05	0.000342	PCDHGB7	5	141417645	141512979	+	4966	protein_cod	protocadherin gamma subfamily B, 7 [Source:HGNC Symbol;Acc:HGNC:8714]
ENSG0000	20.5233	21.77918	13.50827	73.25559	60.03603	42.77054	18.60358	58.68739	-1.651346013	5.11E-05	0.000344	GDF9	5	132861181	132866884	-	3279	protein_cod	growth differentiation factor 9 [Source:HGNC Symbol;Acc:HGNC:4224]
ENSG0000	8.209321	6.222622	14.63396	41.44724	40.93366	37.42422	9.688634	39.93504	-2.053119182	5.21E-05	0.00035	HAPLN2	1	156619294	156625725	+	2241	protein_cod	hyaluronan and proteoglycan link protein 2 [Source:HGNC Symbol;Acc:HGNC:17410]
ENSG0000	29.75879	19.70497	31.51929	121.4501	49.12039	70.39318	26.99435	80.32121	-1.5731978	5.28E-05	0.000354	SORCS2	4	7192538	7742836	+	6317	protein_cod	sortilin related VPS10 domain containing receptor 2 [Source:HGNC Symbol;Acc:HGNC:16698]
ENSG0000	0	1.037104	2.251378	25.06112	23.65056	13.36579	1.096161	20.69249	-4.273681707	5.33E-05	0.000358	PRUNE2	9	76611376	76906087	-	14656	protein_cod	prune homolog 2 [Source:HGNC Symbol;Acc:HGNC:25209]
ENSG0000	1.026165	3.111311	0	21.20557	21.83128	19.60316	1.379159	20.88	-3.89254121	5.34E-05	0.000358	SCRT1	8	144330565	144336281	-	3779	protein_cod	scratch family transcriptional repressor 1 [Source:HGNC Symbol;Acc:HGNC:15950]
ENSG0000	13.34015	3.111311	5.628445	39.51946	28.19874	43.66159	7.359967	37.1266	-2.328260631	5.61E-05	0.000374	SNORA31	13	45337480	45337609	-	130	snoRNA	small nucleolar RNA, H/ACA box 31 [Source:HGNC Symbol;Acc:HGNC:32621]
ENSG0000	5.130825	15.55656	9.005513	47.23058	38.20475	38.31527	9.897631	41.2502	-2.058002187	5.78E-05	0.000385	AL355472.1	1	234372807	234373593	-	575	antisense	uncharacterized LOC101927765 [Source:NCBI gene;Acc:101927765]
ENSG0000	9.235486	16.59366	24.76516	70.36392	33.65656	75.73949	16.86477	59.91999	-1.836330266	5.80E-05	0.000386	MUC12	7	100969623	101018949	+	17130	protein_cod	mucin 12, cell surface associated [Source:HGNC Symbol;Acc:HGNC:7510]
ENSG0000	62.59607	54.9665	41.6505	93.49726	125.5299	122.0742	53.07102	113.7005	-1.097055706	5.84E-05	0.000389	AC004812.2	12	120116907	120119000	+	2094	antisense	novel transcript, antisense to RAB35
ENSG0000	12.31398	21.77918	24.76516	53.01391	61.8553	61.48265	19.61944	58.78395	-1.588732225	5.86E-05	0.000389	AC127024.5	17	30792372	30792833	+	462	lincRNA	novel transcript
ENSG0000	42.07277	45.63256	49.53032	104.1	97.33114	93.56055	45.74522	98.33058	-1.104993867	5.86E-05	0.000389	GRIK2	6	101181257	102070083	+	7585	protein_cod	glutamate ionotropic receptor kainate type subunit 2 [Source:HGNC Symbol;Acc:HGNC:4580]
ENSG0000	35.91578	23.85338	27.01654	105.0639	73.68058	56.13633	28.92857	78.29362	-1.433212401	6.00E-05	0.000397	LINC02340	13	27178263	27251288	-	1098	lincRNA	long intergenic non-protein coding RNA 2340 [Source:HGNC Symbol;Acc:HGNC:53260]
ENSG0000	79.01471	100.5991	108.0662	30.84446	50.93966	42.77054	95.89331	41.51822	1.204219312	6.01E-05	0.000398	ZFPM2	8	104590733	105804532	+	7775	protein_cod	zinc finger protein, FOG family member 2 [Source:HGNC Symbol;Acc:HGNC:16700]
ENSG0000	3.078495	9.333933	7.879823	25.06112	37.29511	40.09738	6.764084	34.1512	-2.342049668	6.05E-05	0.0004	AP004609.3	11	118791254	118793137	+	1884	lincRNA	novel transcript
ENSG0000	10.26165	10.37104	5.628445	33.73613	50.93966	30.2958	8.753711	38.32386	-2.123311317	6.27E-05	0.000414	KDR	4	55078259	55125595	-	6563	protein_cod	kinase insert domain receptor [Source:HGNC Symbol;Acc:HGNC:6307]
ENSG0000	20.5233	20.74207	22.51378	0	4.548184	0.891053	21.25972	1.813079	3.536879418	6.32E-05	0.000417	KRT16	17	41609778	41615899	-	2475	protein_cod	keratin 16 [Source:HGNC Symbol;Acc:HGNC:6423]
ENSG0000	29.75879	37.33573	36.02205	79.03892	94.60223	72.17528	34.37219	81.93881	-1.254262005	6.38E-05	0.00042	IL1RAPL1	X	28587399	29956723	+	3668	protein_cod	interleukin 1 receptor accessory protein like 1 [Source:HGNC Symbol;Acc:HGNC:5996]
ENSG0000	9.235486	11.40814	24.76516	42.41113	58.21676	57.02738	15.13626	52.55176	-1.807711411	6.38E-05	0.000421	MBNL1-AS1	3	152262616	152269555	-	6585	antisense	MBNL1 antisense RNA 1 [Source:HGNC Symbol;Acc:HGNC:44584]
ENSG0000	3.078495	0	3.377067	17.35001	18.19274	33.86001	2.151854	23.13425	-3.44106069	6.42E-05	0.000423	CHMP4BP1	14	55298644	55299231	+	588	processed_	charged multivesicular body protein 4B pseudogene 1 [Source:HGNC Symbol;Acc:HGNC:43616]
ENSG0000	27.70646	9.333933	23.63947	1.927779	1.819274	0.891053	20.22662	1.546035	3.716285634	6.51E-05	0.000427	FLG	1	152302175	152325203	-	12747	protein_cod	filaggrin [Source:HGNC Symbol;Acc:HGNC:3748]
ENSG0000	6.15699	21.77918	18.01103	62.65281	66.40349	37.42422	15.31573	55.4935	-1.860235251	6.58E-05	0.000431	PAK5	20	9537389	9839041	-	4929	protein_cod	p21 (RAC1) activated kinase 5 [Source:HGNC Symbol;Acc:HGNC:15916]
ENSG0000	45.15126	72.59726	64.16428	144.5834	108.2468	123.8563	60.6376	125.5622	-1.050542697	6.60E-05	0.000433	TRPM2-AS	21	44414588	44425272	-	2292	antisense	TRPM2 antisense RNA [Source:HGNC Symbol;Acc:HGNC:50758]
ENSG0000	65.67456	98.52485	85.55237	34.70002	41.84329	23.16737	83.25059	33.23689	1.325635271	6.69E-05	0.000438	ACBD7	10	15077523	15088776	-	1498	protein_cod	acyl-CoA binding domain containing 7 [Source:HGNC Symbol;Acc:HGNC:17715]
ENSG0000	26.68029	17.63076	19.13671	3.855557	0.909637	1.782106	21.14926	2.182433	3.291974787	6.82E-05	0.000446	KRT86	12	52249300	52309163	+	2394	protein_cod	keratin 86 [Source:HGNC Symbol;Acc:HGNC:6463]
ENSG0000	61.5699	79.85698	64.16428	18.3139	31.83729	26.73159	68.53039	25.62759	1.416459841	6.83E-05	0.000446	MGAM2	7	142111749	142222324	+	8162	protein_cod	maltase-glucoamylase 2 (putative) [Source:HGNC Symbol;Acc:HGNC:28101]
ENSG0000	9.235486	21.77918	13.50827	44.33891	53.66857	52.57212	14.84098	50.1932	-1.757811277	7.05E-05	0.000459	LINC02352	15	31216020	31224445	+	1901	lincRNA	long intergenic non-protein coding RNA 2352 [Source:HGNC Symbol;Acc:HGNC:53274]
ENSG0000	36.94194	25.92759	30.3936	106.9917	68.22276	65.04686	31.08771	80.08711	-1.362802195	7.12E-05	0.000463	ABCA4	1	93992835	94121132	-	8530	protein_cod	ATP binding cassette subfamily A member 4 [Source:HGNC Symbol;Acc:HGNC:34]
ENSG0000	12.31398	5.185518	6.754134	30.84446	34.5662	43.66159	8.084545	36.35742	-2.165508277	7.16E-05	0.000465	CFL1P1	10	87817928	87845612	+	2695	transcribed_	cofilin 1 pseudogene 1 [Source:HGNC Symbol;Acc:HGNC:28560]
ENSG0000	6.15699	6.222622	1.125689	31.80835	26.37947	26.73159	4.501767	28.30647	-2.634601402	7.37E-05	0.000478	AP003696.1	8	102854455	102856075	+	770	lincRNA	novel transcript
ENSG0000	40.02044	61.18912	46.15325	116.6306	96.4215	103.3621	49.12094	105.4174	-1.101372151	7.44E-05	0.000482	HLA-L	6	30259584	30293014	+	5505	transcribed_	major histocompatibility complex, class I, L (pseudogene) [Source:HGNC Symbol;Acc:HGNC:4970]
ENSG0000	53.36058	39.40994	45.02756	95.42504	89.14441	114.9458	45.9327	99.83842	-1.119997676	7.49E-05	0.000484	AC105118.1	8	143412749	143417054	+	544	antisense	novel transcript
ENSG0000	8.209321	22.81628	11.25689	57.83336	51.8493	40.98843	14.09416	50.2237	-1.829977423	7.54E-05	0.000487	TMEM269	1	42785007	42816619	+	4373	protein_cod	transmembrane protein 269 [Source:HGNC Symbol;Acc:HGNC:52381]
ENSG0000	36.94194	30.07601	24.76516	59.76114	88.23477	83.75897	30.59437	77.25163	-1.33432818	7.61E-05	0.000491	MALINC1	5	140072857	140108630	-	3439	antisense	mitosis associated long intergenic non-coding RNA 1 [Source:HGNC Symbol;Acc:HGNC:49009]
ENSG0000	57.46524	50.81808	61.9129	122.4139	91.87332	139.0042	56.73207	117.7638	-1.054944646	7.73E-05	0.000499	PPIAP53	17	15506866	15507354	+	489	processed_	peptidylprolyl isomerase A pseudogene 53 [Source:HGNC Symbol;Acc:HGNC:53677]
ENSG0000	45.15126	57.0407	59.66152	111.8112	86.4155	157.7164	53.95116	118.6477	-1.139157351	7.79E-05	0.000502	TSPEAR-AS2	21	44517216	44525952	+	3026	antisense	TSPEAR antisense RNA 2 [Source:HGNC Symbol;Acc:HGNC:16428]
ENSG0000	22.57563	37.33573	42.77618	74.21948	78.22877	99.79792	34.22918	84.08205	-1.301472711	8.10E-05	0.000521	ASIC4	2	219514170	219538772	+	5250	protein_cod	acid sensing ion channel subunit family member 4 [Source:HGNC Symbol;Acc:HGNC:21263]
ENSG0000	6.15699	9.333933	6.754134	26.02501	36.38547	39.20633	7.415019	33.87227	-2.191562365	8.15E-05	0.000523	SLC47A2	17	19678288	19718979	-	6691	protein_cod	solute carrier family 47 member 2 [Source:HGNC Symbol;Acc:HGNC:26439]
ENSG0000	15.39248	5.185518	27.01654	46.26669	57.30712	66.82896	15.86484	56.80092	-1.851174313	8.17E-05	0.000524	CCDC17	1	45620044	45624057	-	3323	protein_cod	coiled-coil domain containing 17 [Source:HGNC Symbol;Acc:HGNC:26574]
ENSG0000	0	0	0	10.60278	15.46383	8.019476	0	11.36203	-5.861824076	8.29E-05	0.000532	AC117498.3	12	48800576	48801086	-	511	processed_	chromosome 20 open reading frame 27 (C20orf27) pseudogene
ENSG0000	4.10466	8.29683	3.377067	27.95279	29.10838	30.2958	5.259519	29.11899	-2.462125352	8.40E-05	0.000538	AC068790.2	12	123968023	123968579	-	557	sense_intro	novel transcript, sense intronic to CCDC92
ENSG0000	19.49714	15.55656	22.51378	72.2917	46.39148	52.57212	19.18916	57.0851	-1.574207275	8.44E-05	0.00054	FAM83E	19	48600810	48614854	-	2529	protein_cod	family with sequence similarity 83 member E [Source:HGNC Symbol;Acc:HGNC:25972]
ENSG0000	102.6165	72.59726	72.0441	30.84446	37.29511	36.53317	82.41929	34.89091	1.240119387	8.52E-05	0.000545	ITGA1	5	52787896	52959210	+	18566	protein_cod	integrin subunit alpha 1 [Source:HGNC Symbol;Acc:HGNC:6134]
ENSG0000	2.05233	3.111311	7.879823	23.13334	21.83128	39.20633	4.347822	28.05698	-2.710358456	8.71E-05	0.000556	AC107959.2	8	23068229	23071328	+	2636	antisense	TNF receptor superfamily member 10d pseudogene [Source:NCBI gene;Acc:286059]
ENSG0000	17.44481	34.22442	27.01654	56.86947	92.78296	65.93791	26.22859	71.86345	-1.455912762	8.73E-05	0.000557	KRT23	17	40922696	40937634	-	2902	protein_cod	keratin 23 [Source:HGNC Symbol;Acc:HGNC:6438]
ENSG0000	17.44481	13.48235	18.01103	47.23058	43.66257	61.48265	16.31273	50.79193	-1.640937123	9.26E-05	0.000589	SMTNL1	11	57542641	57550274	+	1649	protein_cod	smoothelin like 1 [Source:HGNC Symbol;Acc:HGNC:32394]
ENSG0000	9.235486	9.333933	15.75965	40.48335	43.66257	40.09738	11.44302	41.41443	-1.863147121	9.53E-05	0.000604	INSL3	19	17816512	17821574	-	923	protein_cod	insulin like 3 [Source:HGNC Symbol;Acc:HGNC:6086]
ENSG0000	32.83728	51.85518	40.52481	76.14726	112.795	96.23371	41.73909	95.05864	-1.188332405	9.59E-05	0.000607	WDR88	19	33132090	33175795	+	1841	protein_cod	WD repeat domain 88 [Source:HGNC Symbol;Acc:HGNC:26999]
ENSG0000	4.10466	5.185518	0	24.09723	24.56019	24.94948	3.096726	24.53564	-2.961942959	9.61E-05	0.000608	AC111170.3	17	77444704	77447167	+	2464	TEC	TEC
ENSG0000	18.47097	16.59366	12.38258	51.08613	44.5722	52.57212	15.81574	49.41015	-1.639234434	9.78E-05	0.000618	ENPP7P4	3	125848223	125909372	+	1086	transcribed_	ectonucleotide pyrophosphatase/phosphodiesterase 7 pseudogene 4 [Source:HGNC Symbol;Acc:HGNC:48687]
ENSG0000	63.62223	73.63436	52.90739	122.4139	102.789	163.9537	63.38799	129.7189	-1.031767021	9.85E-05	0.000622	VWCE	11	61258286	61295424	-	4874	protein_cod	von Willebrand factor C and EGF domains [Source:HGNC Symbol;Acc:HGNC:26487]
ENSG0000	90.30253	95.41354	117.0717	1.927779	7.277095	23.16737	100.9292	10.79075	3.219813357	9.88E-05	0.000623	AL445490.1	1	27669468	27703063	+	1651	antisense	novel transcript, antisense to IFI6
ENSG0000	8.209321	2.074207	4.502756	24.09723	27.2891	34.75106	4.928761	28.71247	-2.539692774	9.91E-05	0.000625	AC010531.3	16	87396704	87400295	-	513	antisense	novel transcript, antisense to MAP1LC3B
ENSG0000	13.34015	9.333933	13.50827	53.01391	24.56019	60.59159	12.06078	46.05523	-1.934626541	9.96E-05	0.000628	RHD	1	25272393	25330445	+	3313	protein_cod	Rh blood group D antigen [Source:HGNC Symbol;Acc:HGNC:10009]
ENSG0000	2.05233	0	0	15.42223	25.46983	16.93	0.68411	19.27402	-4.776607005	0.000105	0.000662	AC022144.1	19	38738284	38739863	+	1580	antisense	novel transcript, antisense to CAPN12
ENSG0000	4.10466	4.148415	0	22.16945	26.37947	21.38527	2.751025	23.3114	-3.057665083	0.000106	0.000664	AC068790.6	12	123971457	123971714	-	258	sense_intro	novel transcript, sense intronic to CCDC92
ENSG0000	26.68029	28.0018	38.27343	84.82226	58.21676	87.32318	30.98517	76.7874	-1.312689813	0.000108	0.000674	TMEM178A	2	39664982	39717963	+	2470	protein_cod	transmembrane protein 178A [Source:HGNC Symbol;Acc:HGNC:28517]
ENSG0000	2.05233	1.037104	2.251378	19.27779	19.10237	20.49422	1.780271	19.62479	-3.469366239	0.000109	0.000683	AL138831.3	6	3982673	3984130	+	1458	TEC	TEC
ENSG0000	20.5233	8.29683	16.88534	51.08613	42.75293	56.13633	15.23516	49.9918	-1.715430841	0.000109	0.000684	SRP54-AS1	14	34920858	34982532	-	1531	transcribed_	SRP54 antisense RNA 1 (head to head) [Source:HGNC Symbol;Acc:HGNC:50643]
ENSG0000	24.62796	19.70497	32.64498	68.43614	57.30712	74.84844	25.6593	66.8639	-1.386116283	0.000111	0.000692	AL731569.1	10	86521945	86525101	+	3058	antisense	novel transcript
ENSG0000	5.130825	11.40814	10.1312	38.55557	34.5662	35.64211	8.890056	36.25463	-2.031299999	0.000112	0.000701	AC011481.1	19	44950044	44954007	-	361	lincRNA	novel transcript

ENSG0000	3.078495	0	2.251378	26.02501	17.2831	17.82106	1.776624	20.37639	-3.522996336	0.000113	0.000707	AC099811.5	17	42272069	42275571	-	819	sense_intro	novel transcript, sense intronic to STAT5B
ENSG0000	3.078495	3.111311	4.502756	28.91668	31.83729	14.25685	3.564188	25.0036	-2.815667709	0.000114	0.000708	AL353708.3	1	179881607	179882595	-	989	lincRNA	novel transcript, antisense to TOR1AIP1
ENSG0000	20.5233	28.0018	54.03308	94.46115	73.68058	93.56055	34.18606	87.2341	-1.359113219	0.000117	0.000723	AC105429.1	16	87470370	87474370	-	4001	sense_intro	novel transcript, sense intronic to ZCCHC14
ENSG0000	15.39248	23.85338	14.63396	53.01391	46.39148	61.48265	17.95994	53.62935	-1.575795108	0.000117	0.000726	AL135818.1	14	91242759	91252211	+	1916	processed_t	novel transcript, antisense to GPR68
ENSG0000	0	0	0	6.747225	20.92165	6.23737	0	11.30208	-5.855113402	0.000123	0.000757	AC017007.2	4	112826307	112827607	+	1301	processed_p	TAF4b RNA polymerase II, TATA box binding protein (TBP)-associated factor, 105kDa (TAF4B) pseudogene
ENSG0000	3.078495	0	0	11.56667	30.92765	16.93	1.026165	19.80811	-4.235722645	0.000124	0.000762	AL161729.4	9	95514045	95514520	+	476	antisense	novel transcript, antisense to PTCH1
ENSG0000	17.44481	12.44524	23.63947	1.927779	1.819274	0	17.84317	1.249017	3.852058139	0.000124	0.000763	SAMD9L	7	93130055	93148369	-	7135	protein_cod	sterile alpha motif domain containing 9 like [Source:HGNC Symbol;Acc:HGNC:1349]
ENSG0000	26.68029	25.92759	25.89085	69.40003	61.8553	65.04686	26.16624	65.43406	-1.321780002	0.000125	0.000771	GPRC5D	12	12940775	12952147	-	1124	protein_cod	G protein-coupled receptor class C group 5 member D [Source:HGNC Symbol;Acc:HGNC:13310]
ENSG0000	3.078495	3.111311	1.125689	22.16945	19.10237	22.27632	2.438498	21.18272	-3.104242103	0.000127	0.000779	AL137003.1	6	16761138	16762652	+	698	antisense	uncharacterized LOC101928433 [Source:NCBI gene;Acc:101928433]
ENSG0000	72.85772	87.11671	47.27894	25.06112	31.83729	21.38527	69.08446	26.09456	1.40709325	0.000127	0.000781	FAM198B	4	158124474	158173318	-	7880	protein_cod	family with sequence similarity 198 member B [Source:HGNC Symbol;Acc:HGNC:25312]
ENSG0000	8.209321	7.259726	4.502756	38.55557	24.56019	31.18685	6.657268	31.43421	-2.231161061	0.000131	0.000802	AC091057.2	15	30616998	30625773	-	970	antisense	novel transcript, antisense to ARHGAP11B
ENSG0000	31.81112	18.66787	31.51929	53.9778	80.95768	77.5216	27.33276	70.81903	-1.376647299	0.000134	0.000819	USP6	17	5116438	5175034	+	11270	protein_cod	ubiquitin specific peptidase 6 [Source:HGNC Symbol;Acc:HGNC:12629]
ENSG0000	15.39248	18.66787	24.76516	46.26669	54.57821	67.72002	19.6085	56.1883	-1.524656257	0.000134	0.000819	GRIP2	3	14489111	14556075	-	11885	protein_cod	glutamate receptor interacting protein 2 [Source:HGNC Symbol;Acc:HGNC:23841]
ENSG0000	47.20359	31.11311	41.6505	80.00281	82.77695	106.0353	39.98907	89.60502	-1.165072005	0.000134	0.00082	AC005261.3	19	57261354	57262738	+	1385	lincRNA	novel transcript
ENSG0000	0	5.185518	3.377067	43.37502	20.92165	12.47474	2.854195	25.59047	-3.168676347	0.000135	0.000825	CCDC85A	2	56184123	56386173	+	3982	protein_cod	coiled-coil domain containing 85A [Source:HGNC Symbol;Acc:HGNC:29400]
ENSG0000	4.10466	6.222622	21.38809	62.65281	32.74693	40.98843	10.57179	45.46272	-2.120089825	0.000136	0.000834	AC068790.3	12	123966077	123966629	-	553	sense_intro	novel transcript, sense intronic to CCDC92
ENSG0000	99.53801	117.1927	114.8203	48.19447	38.20475	70.39318	110.517	52.26413	1.078411174	0.000137	0.000839	PGF	14	74941834	74955784	-	5962	protein_cod	placental growth factor [Source:HGNC Symbol;Acc:HGNC:8893]
ENSG0000	0	1.037104	2.251378	17.35001	18.19274	18.71211	1.096161	18.08495	-4.080384784	0.000138	0.00084	NLGN1	3	173396284	174286644	+	10148	protein_cod	neuroligin 1 [Source:HGNC Symbol;Acc:HGNC:14291]
ENSG0000	0	0	0	11.56667	8.186731	11.58369	0	10.4457	-5.740382685	0.000139	0.000846	OR10AD1	12	48202083	48203387	-	1305	protein_cod	olfactory receptor family 10 subfamily AD member 1 [Source:HGNC Symbol;Acc:HGNC:14819]
ENSG0000	45.15126	59.11491	46.15325	93.49726	93.69259	128.3116	50.13981	105.1672	-1.068679424	0.000139	0.000846	MAK	6	10762723	10838555	-	4226	protein_cod	male germ cell associated kinase [Source:HGNC Symbol;Acc:HGNC:6816]
ENSG0000	28.73262	28.0018	51.7817	92.53337	78.22877	84.65002	36.17204	85.13739	-1.240763	0.000139	0.00085	LENG8-AS1	19	54444813	54449045	-	1248	antisense	LENG8 antisense RNA 1 [Source:HGNC Symbol;Acc:HGNC:40705]
ENSG0000	47.20359	76.74567	77.67255	24.09723	22.74092	30.2958	67.20727	25.71132	1.383585318	0.00014	0.000851	EDRF1-AS1	10	125725634	125752110	-	1989	antisense	EDRF1 antisense RNA 1 [Source:HGNC Symbol;Acc:HGNC:49501]
ENSG0000	20.5233	11.40814	31.51929	62.65281	67.31312	53.46317	21.15025	61.14303	-1.538530852	0.000141	0.000858	DCT	13	94436808	94479682	-	7543	protein_cod	dopachrome tautomerase [Source:HGNC Symbol;Acc:HGNC:2709]
ENSG0000	37.96811	26.9647	31.51929	92.53337	71.86131	65.93791	32.1507	76.77753	-1.253939764	0.000144	0.000875	FLJ30679	16	86555320	86557299	+	1980	TEC	uncharacterized protein FLJ30679 [Source:NCBI gene;Acc:146512]
ENSG0000	12.31398	17.63076	9.005513	44.33891	39.11438	48.11685	12.98342	43.85672	-1.750986142	0.000146	0.000883	AL158835.2	10	125718771	125719365	-	300	sense_intro	novel transcript
ENSG0000	13.34015	16.59366	18.01103	66.50836	36.38547	48.11685	15.98161	50.3369	-1.65608629	0.000146	0.000884	AC011815.3	18	35257040	35257512	+	473	TEC	TEC
ENSG0000	32.83728	17.63076	18.01103	68.43614	44.5722	83.75897	22.82636	65.5891	-1.519239915	0.000146	0.000887	FAM131B	7	143353400	143362770	-	6430	protein_cod	family with sequence similarity 131 member B [Source:HGNC Symbol;Acc:HGNC:22202]
ENSG0000	0	0	0	8.675004	9.096368	13.36579	0	10.37905	-5.732362181	0.000147	0.000892	ASNSP3	13	100062296	100063601	-	1306	processed_p	asparagine synthetase pseudogene 3 [Source:HGNC Symbol;Acc:HGNC:39399]
ENSG0000	10.26165	12.44524	12.38258	40.48335	40.93366	39.20633	11.69649	40.20778	-1.782702806	0.000148	0.000895	AL845321.1	9	63330326	63343660	+	873	transcribed_	monofunctional C1-tetrahydrofolate synthase, mitochondrial-like [Source:NCBI gene;Acc:100996643]
ENSG0000	7.183155	15.55656	7.879823	31.80835	43.66257	41.87948	10.20651	39.1168	-1.935837828	0.000148	0.000897	INTS4P2	7	65647823	65718688	+	2019	transcribed_	integrator complex subunit 4 pseudogene 2 [Source:HGNC Symbol;Acc:HGNC:22351]
ENSG0000	27.70646	54.9665	25.89085	90.6056	81.86731	89.10529	36.18793	87.19273	-1.2652738	0.000151	0.000911	UGDH-AS1	4	39528019	39594707	+	2787	antisense	UGDH antisense RNA 1 [Source:HGNC Symbol;Acc:HGNC:40601]
ENSG0000	7.183155	9.333933	12.38258	0	0	0	9.633223	0	5.827565561	0.000152	0.000916	C6orf15	6	31111223	31112559	-	1140	protein_cod	chromosome 6 open reading frame 15 [Source:HGNC Symbol;Acc:HGNC:13927]
ENSG0000	55.41291	95.41354	48.40463	145.5473	114.6142	149.6969	66.41036	136.6195	-1.037873643	0.000158	0.000951	AC105219.3	8	143758153	143771822	-	1809	lincRNA	novel transcript
ENSG0000	14.36631	1.037104	7.879823	36.62779	28.19874	49.00791	7.761079	37.94481	-2.288436367	0.000159	0.000958	AC136475.1	11	318640	325631	+	1887	antisense	novel transcript, antisense to IFITM3
ENSG0000	47.20359	45.63256	64.16428	106.9917	110.0661	99.79792	52.33348	105.6186	-1.016201921	0.000161	0.000967	PAOX	10	133379234	133391694	+	2277	protein_cod	polyamine oxidase [Source:HGNC Symbol;Acc:HGNC:20837]
ENSG0000	66.70073	80.89409	57.41014	27.95279	18.19274	33.86001	68.33499	26.66851	1.358204593	0.000161	0.000968	AC073585.1	10	122879681	122898615	+	2135	transcribed_	chromosome 10 open reading frame 88 (C10orf88) pseudogene
ENSG0000	35.91578	30.07601	34.89636	47.23058	86.4155	130.9848	33.62938	88.21028	-1.393666715	0.000166	0.000996	AC009065.8	16	2240487	2241818	+	788	antisense	novel transcript, antisense to DCI
ENSG0000	2.05233	0	1.125689	20.24168	20.92165	12.47474	1.05934	17.87935	-4.074302179	0.000169	0.001009	AL139123.1	10	130104569	130104998	-	430	sense_intro	novel transcript
ENSG0000	23.6018	15.55656	27.01654	71.32781	62.76494	47.2258	22.0583	60.43952	-1.456280583	0.00017	0.001015	SCUBE3	6	35214419	35253079	+	7356	protein_cod	signal peptide, CUB domain and EGF like domain containing 3 [Source:HGNC Symbol;Acc:HGNC:13655]
ENSG0000	20.5233	29.0389	37.14774	57.83336	104.6082	63.26475	28.90331	75.23545	-1.38515636	0.000174	0.001035	CTAGE8	7	144266701	144269288	-	2588	protein_cod	CTAGE family member 8 [Source:HGNC Symbol;Acc:HGNC:37294]
ENSG0000	0	2.074207	0	17.35001	15.46383	20.49422	0.691402	17.76935	-4.656559027	0.000174	0.001037	RF00049	13	22803145	22803224	+	80	snoRNA	
ENSG0000	66.70073	51.85518	70.91841	8.675004	10.91564	36.53317	63.15811	18.70794	1.74783584	0.000174	0.001038	AC091057.4	15	30648797	30649529	+	733	lincRNA	novel transcript
ENSG0000	6.15699	22.81628	32.64498	65.54447	69.1324	57.91844	20.53942	64.19844	-1.652802774	0.000176	0.00105	PRSS51	8	10482878	10547585	-	3036	protein_cod	serine protease 51 [Source:HGNC Symbol;Acc:HGNC:37321]
ENSG0000	44.1251	39.40994	30.3936	70.36392	87.32513	98.01581	37.97621	85.23496	-1.164363995	0.000179	0.001062	LSMEM1	7	112480853	112491062	+	2592	protein_cod	leucine rich single-pass membrane protein 1 [Source:HGNC Symbol;Acc:HGNC:22036]
ENSG0000	3.078495	0	5.628445	15.42223	22.74092	32.0779	2.902314	23.41368	-3.035174969	0.000181	0.001074	AC011477.2	19	19788755	19790531	-	921	sense_intro	novel transcript, sense intronic to ZNF506
ENSG0000	32.83728	36.29863	45.02756	74.21948	103.6996	77.5216	38.05449	85.14656	-1.165200719	0.000183	0.001085	PA2G4P4	3	156809551	156810732	-	1182	processed_p	proliferation-associated 2G4 pseudogene 4 [Source:HGNC Symbol;Acc:HGNC:32217]
ENSG0000	18.47097	42.52125	40.52481	67.47225	93.69259	88.21423	33.83901	83.12636	-1.300747036	0.000183	0.001085	CD72	9	35609533	35646810	-	3386	protein_cod	CD72 molecule [Source:HGNC Symbol;Acc:HGNC:1696]
ENSG0000	32.83728	26.9647	29.26792	68.43614	59.12639	87.32318	29.68996	71.62857	-1.270753235	0.000185	0.001094	NFYC-AS1	1	40690380	40692066	-	1687	antisense	NFYC antisense RNA 1 [Source:HGNC Symbol;Acc:HGNC:49451]
ENSG0000	52.33442	52.89229	60.78721	10.60278	14.55419	29.40474	55.33797	18.18724	1.598584043	0.000185	0.001095	ZSCAN12	6	28378955	28399734	-	5495	protein_cod	zinc finger and SCAN domain containing 12 [Source:HGNC Symbol;Acc:HGNC:13172]
ENSG0000	33.86345	36.29863	31.51929	80.00281	62.76494	90.88739	33.89379	77.88505	-1.199455384	0.000191	0.001129	TECTA	11	121101173	121191493	+	8383	protein_cod	tectorin alpha [Source:HGNC Symbol;Acc:HGNC:11720]
ENSG0000	3.078495	12.44524	3.377067	28.91668	27.2891	39.20633	6.300269	31.80404	-2.328895328	0.000192	0.001133	AC068790.7	12	123960717	123961244	-	528	sense_intro	novel transcript, sense intronic to CCDC92
ENSG0000	2.05233	8.29683	0	24.09723	23.65056	28.51369	3.44972	25.42049	-2.861649131	0.000194	0.001141	ENPP3	6	131628442	131747418	+	3885	protein_cod	ectonucleotide pyrophosphatase/phosphodiesterase 3 [Source:HGNC Symbol;Acc:HGNC:3358]
ENSG0000	47.20359	45.63256	32.64498	80.00281	88.23477	102.4711	41.82705	90.23622	-1.106777398	0.0002	0.001175	AC010326.4	19	57867885	57868834	+	936	transcribed_	ubiquitin-conjugating enzyme E2C (UBE2C) pseudogene
ENSG0000	16.41864	16.59366	12.38258	45.3028	34.5662	64.15581	15.13163	48.00827	-1.66336349	0.000201	0.00118	AC026471.6	16	31470204	31472108	-	1905	TEC	novel transcript
ENSG0000	34.88961	31.11311	58.53583	81.93059	102.789	92.6695	41.51285	92.46302	-1.161446171	0.000202	0.001184	AC132872.2	17	82214227	82217352	+	2368	antisense	novel transcript, antisense CCDC57
ENSG0000	5.130825	12.44524	4.502756	29.88057	22.74092	50.79001	7.359609	34.4705	-2.222534928	0.000202	0.001186	AC015883.1	17	57448218	57450291	+	2074	TEC	TEC
ENSG0000	11.28782	10.37104	9.005513	40.48335	46.39148	26.73159	10.22146	37.8688	-1.886207974	0.000205	0.001201	ABHD11-AS1	7	73735038	73736158	+	662	transcribed_	ABHD11 antisense RNA 1 (tail to tail) [Source:HGNC Symbol;Acc:HGNC:18289]
ENSG0000	2.05233	4.148415	2.251378	17.35001	19.10237	27.62264	2.817374	21.35834	-2.919652514	0.000207	0.001213	AC119396.1	19	7348943	7383385	+	1558	protein_cod	novel protein
ENSG0000	44.1251	23.85338	34.89636	69.40003	88.23477	81.97686	34.29161	79.87056	-1.220209485	0.000209	0.001221	AC121839.2	14	91752856	91759798	-	6943	sense_intro	novel transcript, intronic to CATSPERB
ENSG0000	9.235486	7.259726	9.005513	28.91668	24.56019	52.57212	8.500241	35.34966	-2.058485569	0.000211	0.001232	AL031727.1	1	19608114	19608568	+	455	processed_p	ribosomal protein S14 (RPS14) pseudogene
ENSG0000	63.62223	43.55836	58.53583	81.93059	135.5359	126.5295	55.23881	114.6653	-1.055528219	0.000213	0.001243	C11orf71	11	114391443	114400511	-	2203	protein_cod	chromosome 11 open reading frame 71 [Source:HGNC Symbol;Acc:HGNC:25937]
ENSG0000	568.4954	526.8487	477.2922	123.3778	263.7947	262.8606	524.2121	216.6777	1.273995278	0.000213	0.001243	CENPK	5	65517766	65563171	-	4312	protein_cod	centromere protein K [Source:HGNC Symbol;Acc:HGNC:29479]
ENSG0000	2.05233	7.259726	9.005513	33.73613	17.2831	43.66159	6.105856	31.56027	-2.380559727	0.000213	0.001244	CCDC116	22	21632716	21637327	+	2949	protein_cod	coiled-coil domain containing 116 [Source:HGNC Symbol;Acc:HGNC:26688]
ENSG0000	1.026165	2.074207	4.502756	23.13334	10.91564	31.18685	2.534376	21.74528	-3.121708824	0.000215	0.001254	AC009093.6	16	29272220	29272772	+	553	lincRNA	novel transcript
ENSG0000	58.49141	49.78098	52.90739	80.9667	160.0961	103.3621	53.72659	114.8083	-1.096261675	0.000216	0.001258	RGS5	1	163111121	163321791	-	6821	protein_cod	regulator of G protein signaling 5 [Source:HGNC Symbol;Acc:HGNC:10001]
ENSG0000	18.47097	21.77918	27.01654	55.90558	56.39748	62.3737	22.42223	58.22559	-1.380741348	0.000217	0.001264	AL049869.3	14	64513952	64540368	-	1234	antisense	uncharacterized LOC102723809 [Source:NCBI gene;Acc:102723809]
ENSG0000	13.34015	6.222622	6.754134	43.37502	27.2891	35.64211	8.772301	35.43541	-2.007751683	0.00022	0.001278	AC243829.1	17	36183235	36196471	+	1401	antisense	novel transcript, antisense CCL3L3
ENSG0000	27.70646	32.15021	15.75965	57.83336	81.86731	58.80949	25.20544	66.17005	-1.387280329	0.000222	0.001288	AP000866.1	11	124800450	124834487	+	4854	antisense	uncharacterized LOC100507283 [Source:NCBI gene;Acc:100507283]
ENSG0000	21.54947	17.63076	23.63947	3.855557	1.819274	3.564211	20.9399	3.079681	2.767575412	0.000224	0.001298	KRT37	17	41420753	41424523	-	1475	protein_cod	keratin 37 [Source:HGNC Symbol;Acc:HGNC:6455]

ENSG0000	13.34015	39.40994	9.005513	61.68892	62.76494	70.39318	20.5852	64.94901	-1.65149504	0.000225	0.001305	F5	1	169514166	169586588	-	7158	protein_cod	coagulation factor V [Source:HGNC Symbol;Acc:HGNC:3542]
ENSG0000	4.10466	0	1.125689	11.56667	26.37947	21.38527	1.74345	19.77714	-3.491845601	0.000225	0.001306	AP003419.3	11	67374416	67374932	+	517	lincRNA	novel transcript
ENSG0000	10.26165	10.37104	12.38258	28.91668	47.30111	40.09738	11.00509	38.77172	-1.820522968	0.000226	0.001308	TTC25	17	41930635	41965651	+	2500	protein_cod	tetratricopeptide repeat domain 25 [Source:HGNC Symbol;Acc:HGNC:25280]
ENSG0000	9.235486	3.111311	14.63396	34.70002	34.5662	40.09738	8.993585	36.45453	-2.030550599	0.000227	0.001316	AL031281.1	1	22068340	22068827	-	488	processed_	M-phase phosphoprotein 6 (MPHOSPH6) pseudogene
ENSG0000	61.5699	64.30043	42.77618	18.3139	28.19874	8.910529	56.21551	18.47439	1.608575269	0.00023	0.001329	DENND2A	7	140518420	140673993	-	5148	protein_cod	DENN domain containing 2A [Source:HGNC Symbol;Acc:HGNC:22212]
ENSG0000	0	0	0	6.747225	10.006	12.47474	0	9.742657	-5.641571553	0.000232	0.00134	RNU6-1152I	17	48196680	48196786	-	107	snRNA	RNA, U6 small nuclear 1152, pseudogene [Source:HGNC Symbol;Acc:HGNC:48115]
ENSG0000	28.73262	14.51945	31.51929	65.54447	67.31312	60.59159	24.92379	64.48306	-1.374837896	0.000233	0.001346	LINC00937	12	8295986	8396803	-	5663	lincRNA	long intergenic non-protein coding RNA 937 [Source:HGNC Symbol;Acc:HGNC:48629]
ENSG0000	50.28209	48.74387	49.53032	105.0639	79.1384	120.2921	49.51876	101.4982	-1.035529891	0.000234	0.001351	GALNT9	12	132196372	132329349	-	6969	protein_cod	polypeptide N-acetylgalactosaminyltransferase 9 [Source:HGNC Symbol;Acc:HGNC:4131]
ENSG0000	58.49141	51.85518	46.15325	19.27779	13.64455	23.16737	52.16662	18.69657	1.480826552	0.000234	0.001352	AL683807.1	X	1732584	1763212	+	1001	lincRNA	novel transcript
ENSG0000	2.05233	2.074207	3.377067	25.06112	19.10237	16.03895	2.501202	20.06748	-3.012067259	0.000239	0.001377	RBMS3	3	28574791	30010391	+	18339	protein_cod	RNA binding motif single stranded interacting protein 3 [Source:HGNC Symbol;Acc:HGNC:13427]
ENSG0000	2.05233	11.40814	11.25689	33.73613	33.65656	37.42422	8.23912	34.93897	-2.092788706	0.000251	0.001442	AC013448.2	2	223505476	223510933	+	787	lincRNA	novel transcript
ENSG0000	49.25592	51.85518	57.41014	19.27779	20.92165	19.60316	52.84042	19.9342	1.40528629	0.000252	0.001448	RHEBL1	12	49064685	49070025	-	1290	protein_cod	RHEB like 1 [Source:HGNC Symbol;Acc:HGNC:21166]
ENSG0000	26.68029	32.15021	34.89636	112.7751	57.30712	63.26475	31.24229	77.78231	-1.315600494	0.000253	0.001452	AATBC	21	43805758	43812567	-	4874	antisense	apoptosis associated transcript in bladder cancer [Source:HGNC Symbol;Acc:HGNC:51526]
ENSG0000	8.209321	12.44524	1.125689	13.49445	46.39148	57.02738	7.260085	38.9711	-2.413369279	0.000259	0.001485	LINC01424	21	44802577	44804717	+	1031	antisense	long intergenic non-protein coding RNA 1424 [Source:HGNC Symbol;Acc:HGNC:40558]
ENSG0000	28.73262	21.77918	25.89085	71.32781	53.66857	64.15581	25.46755	63.05073	-1.307202586	0.000261	0.001491	AC005865.2	12	3296202	3366122	-	5981	transcribed_	tetraspanin 11 (TSPAN11) pseudogene
ENSG0000	75.93622	64.30043	56.28445	33.73613	25.46983	8.019476	65.50703	22.40848	1.554390758	0.000265	0.001514	KDM4D	11	94973681	94999518	+	2978	protein_cod	lysine demethylase 4D [Source:HGNC Symbol;Acc:HGNC:25498]
ENSG0000	18.47097	20.74207	16.88534	56.86947	46.39148	50.79001	18.69946	51.35032	-1.455193489	0.000266	0.001517	NFASC	1	204828651	205022822	+	21271	protein_cod	neurofascin [Source:HGNC Symbol;Acc:HGNC:29866]
ENSG0000	1.026165	1.037104	4.502756	14.45834	30.92765	15.1479	2.188675	20.17796	-3.233279286	0.000268	0.001526	SLC15A3	11	60937084	60952530	-	6118	protein_cod	solute carrier family 15 member 3 [Source:HGNC Symbol;Acc:HGNC:18068]
ENSG0000	11.28782	24.89049	12.38258	0.963889	0.909637	1.782106	16.18696	1.218544	3.725734696	0.000273	0.001552	FPR3	19	51795163	51826189	+	2772	protein_cod	formyl peptide receptor 3 [Source:HGNC Symbol;Acc:HGNC:3828]
ENSG0000	49.25592	35.26153	51.7817	106.9917	119.1624	69.50212	45.43305	98.55209	-1.117929566	0.000275	0.001567	MACROD2	20	13995369	16053197	+	11428	protein_cod	MACRO domain containing 2 [Source:HGNC Symbol;Acc:HGNC:16126]
ENSG0000	30.78495	30.07601	13.50827	58.79725	70.04203	65.04686	24.78974	64.62871	-1.375872903	0.000276	0.001569	ASPDH	19	50511600	50514690	-	1653	protein_cod	aspartate dehydrogenase domain containing [Source:HGNC Symbol;Acc:HGNC:33856]
ENSG0000	28.73262	22.81628	31.51929	61.68892	75.49986	61.48265	27.6894	66.22381	-1.260296681	0.00028	0.001591	KLHL30	2	238138722	238152947	+	3726	protein_cod	kelch like family member 30 [Source:HGNC Symbol;Acc:HGNC:24770]
ENSG0000	18.47097	13.48235	21.38809	34.70002	59.12639	62.3737	17.78047	52.0667	-1.554933866	0.000281	0.001596	USP3-AS1	15	63544247	63601589	-	7141	antisense	USP3 antisense RNA 1 [Source:HGNC Symbol;Acc:HGNC:44140]
ENSG0000	8.209321	2.074207	2.251378	26.9889	24.56019	23.16737	4.178302	24.90549	-2.562610186	0.000282	0.001598	CD96	3	111292719	111665750	+	5762	protein_cod	CD96 molecule [Source:HGNC Symbol;Acc:HGNC:16892]
ENSG0000	14.36631	5.185518	6.754134	35.6639	34.5662	33.86001	8.768655	34.6967	-1.978528969	0.000282	0.001601	STKLD1	9	133376367	133406096	+	4408	protein_cod	serine/threonine kinase like domain containing 1 [Source:HGNC Symbol;Acc:HGNC:28669]
ENSG0000	1.026165	0	2.251378	12.53056	12.73492	25.84053	1.092514	17.03534	-3.995621326	0.00029	0.001641	AC072022.2	3	187743686	187746028	+	1144	antisense	BCL6 antisense 1
ENSG0000	0	2.074207	2.251378	17.35001	8.186731	30.2958	1.441862	18.61085	-3.709575113	0.00029	0.001641	AL583810.2	14	104842663	104845916	+	3254	lincRNA	novel transcript
ENSG0000	0	0	0	21.20557	1.819274	8.910529	0	10.64512	-5.764974051	0.000296	0.00167	AC240565.2	17	64099	76866	-	2096	lincRNA	novel transcript
ENSG0000	2.05233	2.074207	0	17.35001	14.55419	19.60316	1.375513	17.16912	-3.612784508	0.000298	0.001683	OR7E136P	7	6866658	6867672	-	1015	unprocesse	olfactory receptor family 7 subfamily E member 136 pseudogene [Source:HGNC Symbol;Acc:HGNC:19557]
ENSG0000	40.02044	35.26153	33.77067	1.927779	17.2831	3.564211	36.35088	7.591697	2.254131037	0.000303	0.001706	KRT6A	12	52487174	52493257	-	2928	protein_cod	keratin 6A [Source:HGNC Symbol;Acc:HGNC:6443]
ENSG0000	17.44481	15.55656	21.38809	50.12224	39.11438	65.04686	18.12982	51.42783	-1.507686977	0.000303	0.001706	FOXN4	12	109277979	109309220	-	3471	protein_cod	forkhead box N4 [Source:HGNC Symbol;Acc:HGNC:21399]
ENSG0000	3.078495	2.074207	0	15.42223	15.46383	24.05843	1.717568	18.31483	-3.387154859	0.000304	0.001711	AC011773.4	8	85172077	85177062	-	1540	antisense	uncharacterized LOC102723322 [Source:NCBI gene;Acc:102723322]
ENSG0000	5.130825	3.111311	5.628445	33.73613	20.92165	20.49422	4.623527	25.05066	-2.440281692	0.000305	0.001713	AC010240.2	5	132942932	132945050	+	1205	processed_	eukaryotic translation elongation factor 1 alpha 1 (EEF1A1) pseudogene
ENSG0000	4.10466	10.37104	3.377067	37.59168	24.56019	24.94948	5.950921	29.03379	-2.277493701	0.000305	0.001717	AC087749.1	17	75683543	75684799	-	637	antisense	novel transcript, antisense to SAP30BP
ENSG0000	11.28782	19.70497	10.1312	50.12224	44.5722	36.53317	13.708	43.74254	-1.669083992	0.000311	0.001742	AC140479.2	2	110360608	110363737	-	3130	processed_	zinc finger protein 532 (ZNF532) pseudogene
ENSG0000	15.39248	17.63076	28.14223	55.90558	54.57821	53.46317	20.38849	54.64899	-1.428812844	0.000311	0.001743	SLC28A2	15	45252230	45277845	+	5192	protein_cod	solute carrier family 28 member 2 [Source:HGNC Symbol;Acc:HGNC:11002]
ENSG0000	2.05233	4.148415	5.628445	25.06112	20.92165	23.16737	3.943063	23.05005	-2.55795071	0.000313	0.001754	AL049830.4	14	31018446	31018796	+	351	processed_	intraflagellar transport 20 homolog (Chlamydomonas) (IFT20) pseudogene
ENSG0000	63.62223	47.70677	64.16428	26.02501	24.56019	18.71211	58.49776	23.09911	1.341800384	0.000313	0.001754	ALOXE3	17	8095900	8119047	-	3760	protein_cod	arachidonate lipoxygenase 3 [Source:HGNC Symbol;Acc:HGNC:13743]
ENSG0000	52.33442	58.07781	76.54686	82.89448	149.1804	147.9148	62.31969	126.6632	-1.027434827	0.000314	0.00176	SAMD13	1	84298366	84389957	+	3455	protein_cod	sterile alpha motif domain containing 13 [Source:HGNC Symbol;Acc:HGNC:24582]
ENSG0000	7.183155	1.037104	10.1312	31.80835	20.92165	39.20633	6.117154	30.64544	-2.33664343	0.000317	0.001773	LINC02019	3	50669989	50672048	+	2060	lincRNA	long intergenic non-protein coding RNA 2019 [Source:HGNC Symbol;Acc:HGNC:52854]
ENSG0000	41.0466	43.55836	36.02205	79.03892	98.24078	77.5216	40.209	84.93377	-1.077487675	0.000318	0.001774	AC062029.1	2	88627539	88631821	+	4283	antisense	uncharacterized LOC101928403 [Source:NCBI gene;Acc:101928403]
ENSG0000	17.44481	14.51945	29.26792	46.26669	49.12039	76.63055	20.41072	57.33921	-1.498078055	0.000318	0.001774	PLCB2	15	40278176	40307935	-	7350	protein_cod	phospholipase C beta 2 [Source:HGNC Symbol;Acc:HGNC:9055]
ENSG0000	41.0466	29.0389	19.13671	60.72503	75.49986	81.08581	29.74074	72.4369	-1.279637108	0.000318	0.001775	HIST2H2BF	1	149782689	149812373	-	3196	protein_cod	histone cluster 2 H2B family member f [Source:HGNC Symbol;Acc:HGNC:24700]
ENSG0000	1.026165	4.148415	0	13.49445	21.83128	20.49422	1.72486	18.60665	-3.408323543	0.000318	0.001776	AC008649.2	19	38683873	38693606	-	242	antisense	novel transcript, antisense to ACTN4
ENSG0000	0	0	0	6.747225	15.46383	6.23737	0	9.482807	-5.601700134	0.00032	0.001785	AL357552.1	1	8798475	8799506	+	415	unprocesse	ribosomal protein L27 (RPL27) pseudogene
ENSG0000	51.30825	74.67147	41.6505	5.783336	19.10237	26.73159	55.87674	17.20576	1.693437151	0.000327	0.001819	VN1R81P	19	21123817	21124188	+	372	unprocesse	vomeronasal 1 receptor 81 pseudogene [Source:HGNC Symbol;Acc:HGNC:37401]
ENSG0000	21.54947	23.85338	33.77067	62.65281	52.75894	80.19476	26.39117	65.20217	-1.309866225	0.000339	0.001882	TUBBP5	9	138150075	138177433	+	2814	transcribed_	tubulin beta pseudogene 5 [Source:HGNC Symbol;Acc:HGNC:23674]
ENSG0000	6.15699	10.37104	13.50827	39.51946	38.20475	30.2958	10.0121	36.00667	-1.853008833	0.00034	0.001884	DNM1P35	15	75727670	75738623	-	3188	antisense	dynamin 1 pseudogene 35 [Source:HGNC Symbol;Acc:HGNC:35182]
ENSG0000	8.209321	10.37104	4.502756	21.20557	40.02402	35.64211	7.694371	32.29057	-2.063103816	0.000348	0.001927	AC068792.1	12	31443792	31444208	-	417	sense_intro	novel transcript, sense intronic to DENND5B
ENSG0000	11.28782	26.9647	21.38809	39.51946	64.58421	65.93791	19.8802	56.68053	-1.514883677	0.000349	0.001928	INTS6-AS1	13	51452367	51552364	+	9866	antisense	INTS6 antisense RNA 1 [Source:HGNC Symbol;Acc:HGNC:42691]
ENSG0000	9.235486	37.33573	20.2624	84.82226	46.39148	67.72002	22.27787	66.31125	-1.572577154	0.000349	0.001932	AL121832.2	20	62386303	62386970	-	668	antisense	novel transcript, antisense to RPS21
ENSG0000	92.35486	84.0054	54.03308	41.44724	26.37947	31.18685	76.79778	33.00452	1.223052471	0.00035	0.001934	FBXO48	2	68459419	68467258	-	5666	protein_cod	F-box protein 48 [Source:HGNC Symbol;Acc:HGNC:33857]
ENSG0000	31.81112	36.29863	46.15325	50.12224	114.6142	106.9263	38.08767	90.55428	-1.25393495	0.00035	0.001934	KLRC2	12	10426854	10442300	-	1499	protein_cod	killer cell lectin like receptor C2 [Source:HGNC Symbol;Acc:HGNC:6375]
ENSG0000	32.83728	38.37284	31.51929	53.9778	76.40949	112.2727	34.24314	80.88665	-1.241083908	0.000351	0.00194	AP002748.3	11	66473490	66480233	-	2057	antisense	uncharacterized LOC101928069 [Source:NCBI gene;Acc:101928069]
ENSG0000	10.26165	13.48235	22.51378	62.65281	40.02402	40.09738	15.41926	47.5914	-1.632231101	0.000353	0.001949	AC093520.1	16	31402565	31404699	+	1767	sense_overl	novel transcript
ENSG0000	15.39248	23.85338	14.63396	57.83336	42.75293	50.79001	17.95994	50.45877	-1.486935609	0.000354	0.001952	AP001099.1	18	10594590	10604798	+	855	lincRNA	novel transcript
ENSG0000	17.44481	45.63256	19.13671	64.58058	81.86731	68.61107	27.40469	71.68632	-1.384365894	0.000355	0.001958	AL133338.1	6	100881471	100882987	+	1517	antisense	novel transcript, antisense to ASCC3
ENSG0000	44.1251	56.0036	74.29548	133.0167	89.14441	126.5295	58.14139	116.2302	-1.00247471	0.000358	0.001971	AP002884.1	11	112270749	112362534	+	5018	lincRNA	uncharacterized LOC283140 [Source:NCBI gene;Acc:283140]
ENSG0000	21.54947	5.185518	12.38258	43.37502	41.84329	47.2258	13.03919	44.14804	-1.757570456	0.000368	0.002026	HFM1	1	91260766	91404869	-	5827	protein_cod	HFM1, ATP dependent DNA helicase homolog [Source:HGNC Symbol;Acc:HGNC:20193]
ENSG0000	0	0	2.251378	13.49445	19.10237	15.1479	0.750459	15.91491	-4.475128246	0.000374	0.002054	BTF3P7	6	6794915	6795402	-	488	processed_	basic transcription factor 3 pseudogene 7 [Source:HGNC Symbol;Acc:HGNC:38569]
ENSG0000	0	0	0	8.675004	5.457821	13.36579	0	9.166206	-5.552869967	0.000374	0.002054	AC032011.1	15	62387327	62388875	-	1549	lincRNA	novel transcript
ENSG0000	1.026165	0	0	8.675004	17.2831	13.36579	0.342055	13.10797	-5.107682199	0.000381	0.002086	AC105046.1	8	22679013	22684009	-	4997	sense_overl	novel transcript, overlapping to EGR3
ENSG0000	8.209321	11.40814	5.628445	0	0	0	8.415302	0	5.636273387	0.000386	0.002108	C8orf31	8	143039209	143059942	+	2929	processed_t	chromosome 8 open reading frame 31 (putative) [Source:HGNC Symbol;Acc:HGNC:26731]
ENSG0000	14.36631	22.81628	21.38809	56.86947	43.66257	57.02738	19.52356	52.51981	-1.429439203	0.000387	0.002116	AC097103.2	3	139389815	139583319	+	2120	antisense	uncharacterized LOC100507291 [Source:NCBI gene;Acc:100507291]
ENSG0000	3.078495	21.77918	20.2624	68.43614	48.21075	40.09738	15.04003	52.24809	-1.801282151	0.0004	0.002177	WNT10A	2	218880363	218899581	+	3412	protein_cod	Wnt family member 10A [Source:HGNC Symbol;Acc:HGNC:13829]
ENSG0000	27.70646	22.81628	20.2624	37.59168	65.49385	86.43213	23.59505	63.17255	-1.420762054	0.000403	0.002189	AL359762.3	1	84498350	84556688	-	2686	processed_t	novel transcript
ENSG0000	6.15699	10.37104	2.251378	26.9889	32.74693	26.73159	6.259802	28.82247	-2.19097813	0.000415	0.002249	HCLS1	3	121631399	121660927	-	5093	protein_cod	hematopoietic cell-specific Lyn substrate 1 [Source:HGNC Symbol;Acc:HGNC:4844]
ENSG0000	0	1.037104	1.125689	37.59168	10.006	5.346317	0.720931	17.648	-4.630381043	0.000416	0.002253	AL158801.1	14	55282296	55282643	-	348	processed_	ribosomal protein L34 (RPL34) pseudogene
ENSG0000	14.36631	13.48235	10.1312	40.48335	32.74693	47.2258	12.65995	40.15203	-1.661699569	0.000419	0.002266	LRMDA	10	75431453	76560167	+	6560	protein_cod	leucine rich melanocyte differentiation associated [Source:HGNC Symbol;Acc:HGNC:23405]

ENSG0000	16.41864	29.0389	29.26792	57.83336	74.59022	54.35422	24.90849	62.25927	-1.324980296	0.00042	0.002272	RAPGEF3	12	47734367	47771040	-	12078	protein_cod	Rap guanine nucleotide exchange factor 3 [Source:HGNC Symbol;Acc:HGNC:16629]
ENSG0000	8.209321	19.70497	23.63947	72.2917	37.29511	48.11685	17.18459	52.56789	-1.617781884	0.000424	0.002289	HNRNPA1P1	17	2306761	2307715	-	955	processed_	heterogeneous nuclear ribonucleoprotein A1 pseudogene 16 [Source:HGNC Symbol;Acc:HGNC:39134]
ENSG0000	2.05233	2.074207	0	27.95279	7.277095	17.82106	1.375513	17.68365	-3.654616229	0.000425	0.002293	PDGFD	11	103907186	104164379	-	4266	protein_cod	platelet derived growth factor D [Source:HGNC Symbol;Acc:HGNC:30620]
ENSG0000	28.73262	22.81628	22.51378	60.72503	57.30712	60.59159	24.68756	59.54125	-1.268304198	0.000431	0.002325	AC017116.2	7	44011882	44038978	-	2698	processed_t	novel transcript
ENSG0000	5.130825	5.185518	7.879823	26.9889	22.74092	31.18685	6.065389	26.97222	-2.159531837	0.000434	0.002335	AC090260.1	15	78141243	78143173	+	1931	lincRNA	novel transcript
ENSG0000	19.49714	6.222622	4.502756	35.6639	39.11438	40.98843	10.07417	38.58891	-1.927086385	0.000434	0.002335	AC008610.1	5	180293245	180295253	+	393	antisense	novel transcript
ENSG0000	30.78495	3.111311	13.50827	67.47225	52.75894	47.2258	15.80151	55.819	-1.816720013	0.000438	0.002357	DOCK11	X	118495898	118686163	+	6721	protein_cod	dedicator of cytokinesis 11 [Source:HGNC Symbol;Acc:HGNC:23483]
ENSG0000	1.026165	5.185518	11.25689	37.59168	20.92165	29.40474	5.822858	29.30602	-2.349525496	0.000439	0.002358	AC012615.4	19	1860250	1862019	+	607	antisense	novel transcript, antisense to KLF16
ENSG0000	38.99427	37.33573	23.63947	84.82226	55.48785	91.77844	33.32316	77.36285	-1.21086086	0.000441	0.002369	AC004264.1	22	30246205	30246998	+	794	antisense	uncharacterized LOC91370 [Source:NCBI gene;Acc:91370]
ENSG0000	21.54947	22.81628	19.13671	55.90558	62.76494	44.55264	21.16749	54.40772	-1.359929699	0.000447	0.002396	AC109322.1	8	144078002	144079265	-	1264	antisense	TEC
ENSG0000	0	3.111311	0	12.53056	19.10237	16.93	1.037104	16.18765	-3.940677379	0.00045	0.002412	EPHA1-AS1	7	143407813	143523449	+	5316	antisense	EPHA1 antisense RNA 1 [Source:HGNC Symbol;Acc:HGNC:27799]
ENSG0000	35.91578	31.11311	21.38809	3.855557	4.548184	11.58369	29.47233	6.662476	2.139019074	0.000452	0.002422	AC097534.1	4	173322206	173329694	-	521	antisense	novel transcript
ENSG0000	28.73262	33.18732	42.77618	6.747225	6.367458	15.1479	34.89871	9.420861	1.881890674	0.000454	0.002432	TSPAN11	12	30926428	30996599	+	7113	protein_cod	tetraspanin 11 [Source:HGNC Symbol;Acc:HGNC:30795]
ENSG0000	5.130825	5.185518	2.251378	44.33891	15.46383	16.03895	4.189241	25.28056	-2.580308822	0.000459	0.002452	AC105137.2	15	75452964	75453947	-	984	sense_intro	novel transcript, sense intronic to SIN3A
ENSG0000	0	1.037104	2.251378	12.53056	17.2831	16.93	1.096161	15.58122	-3.864793732	0.000464	0.002475	HMGB1P7	10	31913143	31913754	+	612	processed_	high mobility group box 1 pseudogene 7 [Source:HGNC Symbol;Acc:HGNC:39096]
ENSG0000	3.078495	0	0	19.27779	15.46383	13.36579	1.026165	16.0358	-3.929469518	0.000476	0.002533	DNASE2B	1	84398532	84415018	+	1476	protein_cod	deoxyribonuclease 2 beta [Source:HGNC Symbol;Acc:HGNC:28875]
ENSG0000	25.65413	18.66787	16.88534	1.927779	3.638547	4.455264	20.40244	3.34053	2.603992441	0.000476	0.002533	AC096637.2	1	211675762	211690103	+	2452	antisense	uncharacterized LOC91548 [Source:NCBI gene;Acc:91548]
ENSG0000	3.078495	0	2.251378	22.16945	11.82528	18.71211	1.776624	17.56895	-3.309235885	0.000492	0.002609	AL627171.1	14	49863072	49864379	+	1308	lincRNA	novel transcript
ENSG0000	38.99427	21.77918	9.005513	1.927779	4.548184	4.455264	23.25965	3.643742	2.670975714	0.000492	0.002612	ANXA8	10	47460162	47484158	-	4955	protein_cod	annexin A8 [Source:HGNC Symbol;Acc:HGNC:546]
ENSG0000	35.91578	43.55836	41.6505	72.2917	84.59622	92.6695	40.37488	83.18581	-1.044465139	0.000498	0.002638	CYP2D7	22	42140203	42144577	-	2260	polymorphic	cytochrome P450 family 2 subfamily D member 7 (gene/pseudogene) [Source:HGNC Symbol;Acc:HGNC:2624]
ENSG0000	6.15699	2.074207	1.125689	22.16945	19.10237	21.38527	3.118962	20.8857	-2.726027817	0.000499	0.002639	AL357874.2	9	35642514	35643517	+	679	antisense	novel transcript
ENSG0000	10.26165	11.40814	12.38258	0	0	0.891053	11.35079	0.297018	5.103666549	0.000499	0.002641	MIR4483	10	113777993	113778054	-	62	miRNA	microRNA 4483 [Source:HGNC Symbol;Acc:HGNC:41761]
ENSG0000	0	0	0	9.638893	6.367458	9.801581	0	8.602644	-5.460316994	0.000503	0.002661	AC005237.1	2	241015599	241064116	-	559	antisense	novel transcript
ENSG0000	50.28209	59.11491	22.51378	117.5945	84.59622	91.77844	43.97026	97.98972	-1.149675106	0.000509	0.002683	NAT16	7	101170493	101180276	-	3871	protein_cod	N-acetyltransferase 16 (putative) [Source:HGNC Symbol;Acc:HGNC:22030]
ENSG0000	14.36631	15.55656	10.1312	33.73613	48.21075	40.98843	13.35136	40.97844	-1.614489918	0.000509	0.002686	AC000089.1	22	19792294	19793094	+	801	processed_	ribosomal protein L7a (RPL7A) pseudogene
ENSG0000	0	0	0	7.711115	7.277095	10.69263	0	8.560281	-5.454134959	0.000514	0.002706	AL391262.1	14	69154311	69154804	+	494	lincRNA	novel transcript
ENSG0000	25.65413	20.74207	30.3936	5.783336	6.367458	5.346317	25.5966	5.83237	2.132629734	0.000516	0.002712	PTHLH	12	27958084	27972705	-	3377	protein_cod	parathyroid hormone like hormone [Source:HGNC Symbol;Acc:HGNC:9607]
ENSG0000	20.5233	29.0389	27.01654	54.94169	61.8553	65.04686	25.52625	60.61462	-1.249610343	0.000517	0.002718	LINC00659	20	62774128	62775412	-	540	antisense	long intergenic non-protein coding RNA 659 [Source:HGNC Symbol;Acc:HGNC:44316]
ENSG0000	43.09893	49.78098	28.14223	90.6056	64.58421	111.3816	40.34071	88.85714	-1.135735421	0.000518	0.002724	SPRY4-AS1	5	142325293	142672001	+	1811	antisense	SPRY4 antisense RNA 1 [Source:HGNC Symbol;Acc:HGNC:53465]
ENSG0000	23.6018	40.44704	20.2624	2.891668	10.006	5.346317	28.10375	6.08133	2.20414257	0.000527	0.002767	RSAD2	2	6865806	6898239	+	4834	protein_cod	radical S-adenosyl methionine domain containing 2 [Source:HGNC Symbol;Acc:HGNC:30908]
ENSG0000	1.026165	1.037104	2.251378	4.819447	28.19874	19.60316	1.438216	17.54045	-3.626472115	0.000535	0.002807	AC005324.3	17	15571491	15651653	-	5514	protein_cod	novel tripartite motif-containing 16 (TRIM16) and CMT1A duplicated region transcript 1 (CDRT1) protein
ENSG0000	2.05233	4.148415	0	13.49445	20.01201	21.38527	2.066915	18.29724	-3.123209239	0.000536	0.002814	AC146507.2	5	177264766	177265241	+	476	processed_	ribosomal protein L21 (RPL21) pseudogene
ENSG0000	34.88961	18.66787	46.15325	77.11115	73.68058	78.41265	33.23691	76.40146	-1.205809537	0.000537	0.002814	AC010326.3	19	57867038	57868172	-	679	sense_intro	novel transcript, sense intronic to ZNF814
ENSG0000	21.54947	12.44524	18.01103	50.12224	43.66257	49.00791	17.33525	47.59757	-1.457088621	0.000538	0.002818	AP001527.2	11	102109827	102110457	-	631	lincRNA	novel transcript
ENSG0000	12.31398	21.77918	14.63396	45.3028	60.94567	35.64211	16.24237	47.29686	-1.540723202	0.000538	0.00282	AL358777.1	6	10659498	10692860	+	3467	antisense	novel transcript, antisense to C6orf52
ENSG0000	13.34015	13.48235	16.88534	42.41113	41.84329	41.87948	14.56928	42.04464	-1.531877105	0.000542	0.002838	AC008555.2	19	34837889	34855304	+	6828	lincRNA	novel transcript
ENSG0000	0	1.037104	1.125689	18.3139	8.186731	18.71211	0.720931	15.07091	-4.406871226	0.000543	0.002841	RARRES2P4	4	190037916	190038399	-	484	processed_	retinoic acid receptor responder 2 pseudogene 4 [Source:HGNC Symbol;Acc:HGNC:48703]
ENSG0000	5.130825	1.037104	1.125689	34.70002	8.186731	19.60316	2.431206	20.82997	-3.081656904	0.000556	0.002901	TMC2	20	2536607	2641784	+	5176	protein_cod	transmembrane channel like 2 [Source:HGNC Symbol;Acc:HGNC:16527]
ENSG0000	2.05233	0	5.628445	14.45834	15.46383	31.18685	2.560258	20.36967	-3.021437073	0.000558	0.002914	AC078909.2	15	37109587	37109984	+	398	lincRNA	novel transcript
ENSG0000	5.130825	10.37104	9.005513	31.80835	38.20475	24.05843	8.169125	31.35717	-1.942873049	0.000558	0.002915	STPG2-AS1	4	97366926	97490164	+	537	antisense	STPG2 antisense RNA 1 [Source:HGNC Symbol;Acc:HGNC:41209]
ENSG0000	6.15699	10.37104	9.005513	27.95279	29.10838	37.42422	8.51118	31.49513	-1.890315636	0.000562	0.002928	TOLLIP-AS1	11	1309769	1310707	+	939	antisense	TOLLIP antisense RNA 1 (head to head) [Source:HGNC Symbol;Acc:HGNC:27403]
ENSG0000	21.54947	7.259726	13.50827	38.55557	39.11438	55.24528	14.10582	44.30508	-1.65039607	0.000564	0.002939	FAM186A	12	50326230	50396622	-	8296	protein_cod	family with sequence similarity 186 member A [Source:HGNC Symbol;Acc:HGNC:26980]
ENSG0000	0	2.074207	0	12.53056	28.19874	7.128423	0.691402	15.95258	-4.502288225	0.000565	0.002941	RNU6ATAC8	8	102421197	102421322	+	126	snRNA	RNA, U6atac small nuclear 8, pseudogene [Source:HGNC Symbol;Acc:HGNC:46907]
ENSG0000	0	2.074207	0	20.24168	10.006	15.1479	0.691402	15.13186	-4.424203031	0.000565	0.002941	PRDX2P4	6	100136064	100136663	+	600	processed_	peroxiredoxin 2 pseudogene 4 [Source:HGNC Symbol;Acc:HGNC:44969]
ENSG0000	0	2.074207	4.502756	31.80835	8.186731	19.60316	2.192321	19.86608	-3.205174227	0.000567	0.002951	AC087491.1	17	39619613	39622513	+	616	lincRNA	novel transcript
ENSG0000	2.05233	1.037104	1.125689	16.38612	19.10237	11.58369	1.405041	15.69073	-3.473599067	0.000579	0.003007	AC011753.2	2	109594693	109605698	-	610	sense_intro	novel transcript, sense intronic to SEPT10
ENSG0000	10.26165	18.66787	12.38258	39.51946	34.5662	51.68107	13.7707	41.92224	-1.605651202	0.000593	0.003077	AC087500.1	17	5192084	5248069	+	2324	antisense	uncharacterized LOC100130950 [Source:NCBI gene;Acc:100130950]
ENSG0000	0	0	0	17.35001	2.72891	7.128423	0	9.069114	-5.533681377	0.000594	0.003077	AC005391.1	19	956485	958149	+	341	sense_intro	novel transcript, sense intronic to ARID3A
ENSG0000	12.31398	24.89049	24.76516	53.9778	56.39748	50.79001	20.65654	53.72177	-1.382612066	0.000595	0.003083	LINC01271	20	50310711	50321342	-	2250	lincRNA	long intergenic non-protein coding RNA 1271 [Source:HGNC Symbol;Acc:HGNC:50327]
ENSG0000	45.15126	42.52125	31.51929	85.78615	86.4155	73.95739	39.7306	82.05301	-1.042703375	0.000613	0.003163	KCNN3	1	154697455	154870280	-	13598	protein_cod	potassium calcium-activated channel subfamily N member 3 [Source:HGNC Symbol;Acc:HGNC:6292]
ENSG0000	34.88961	21.77918	34.89636	8.675004	10.91564	2.673159	30.52172	7.421268	2.043843967	0.000614	0.003168	IL1RN	2	113107214	113134016	+	3256	protein_cod	interleukin 1 receptor antagonist [Source:HGNC Symbol;Acc:HGNC:6000]
ENSG0000	42.07277	16.59366	19.13671	74.21948	68.22276	56.13633	25.93438	66.19286	-1.346696761	0.000616	0.00318	TEN1-CDK3	17	75979231	76005999	+	3898	protein_cod	TEN1-CDK3 readthrough (NMD candidate) [Source:HGNC Symbol;Acc:HGNC:44420]
ENSG0000	27.70646	29.0389	15.75965	51.08613	60.03603	67.72002	24.16834	59.61406	-1.298074263	0.000618	0.003187	ANO7	2	241188509	241225377	+	5695	protein_cod	anoctamin 7 [Source:HGNC Symbol;Acc:HGNC:31677]
ENSG0000	28.73262	37.33573	28.14223	71.32781	59.12639	78.41265	31.40353	69.62228	-1.14736367	0.000624	0.003211	ZNF503-AS2	10	75401519	75408982	+	3348	antisense	ZNF503 antisense RNA 2 [Source:HGNC Symbol;Acc:HGNC:23525]
ENSG0000	3.078495	7.259726	5.628445	14.45834	37.29511	26.73159	5.322222	26.16168	-2.301361835	0.000627	0.003224	AC026954.1	17	7330452	7335413	+	1293	unitary_pse	novel protein phosphatase inhibitor 2 (IPP-2) domain pseudogene
ENSG0000	45.15126	88.15381	45.02756	27.95279	24.56019	12.47474	59.44421	21.66257	1.46124642	0.00063	0.003241	PRICKLE1	12	42456757	42590355	-	8247	protein_cod	prickle planar cell polarity protein 1 [Source:HGNC Symbol;Acc:HGNC:17019]
ENSG0000	59.51757	77.78278	73.16979	30.84446	33.65656	32.96896	70.15671	32.48999	1.109509141	0.000631	0.003246	TRABD2A	2	84821650	84907008	-	8771	protein_cod	TraB domain containing 2A [Source:HGNC Symbol;Acc:HGNC:27013]
ENSG0000	96.45952	137.9348	100.1863	47.23058	78.22877	38.31527	111.5269	54.59154	1.030951341	0.000633	0.003253	STS	X	7219456	7354810	+	6521	protein_cod	steroid sulfatase [Source:HGNC Symbol;Acc:HGNC:11425]
ENSG0000	32.83728	21.77918	18.01103	53.01391	51.8493	76.63055	24.20916	60.49792	-1.318038219	0.000636	0.00327	AC132192.2	11	9459556	9460702	-	1147	antisense	uncharacterized LOC644656 [Source:NCBI gene;Acc:644656]
ENSG0000	13.34015	22.81628	25.89085	51.08613	48.21075	60.59159	20.68243	53.29616	-1.370602749	0.000653	0.003353	AC026471.4	16	31449535	31453493	-	449	lincRNA	novel transcript
ENSG0000	92.35486	69.48595	81.04961	46.26669	39.11438	32.0779	80.96347	39.15299	1.050482589	0.000657	0.003369	UBL7-AS1	15	74461265	74481302	+	1710	antisense	UBL7 antisense RNA 1 (head to head) [Source:HGNC Symbol;Acc:HGNC:48614]
ENSG0000	31.81112	43.55836	57.41014	107.9556	70.95167	98.01581	44.25987	92.3077	-1.063899261	0.000671	0.003431	GUCA1B	6	42184401	42194916	-	1113	protein_cod	guanylate cyclase activator 1B [Source:HGNC Symbol;Acc:HGNC:4679]
ENSG0000	3.078495	1.037104	3.377067	20.24168	14.55419	19.60316	2.497555	18.13301	-2.867362826	0.000674	0.003447	LINC01126	2	43227210	43228855	+	1646	lincRNA	long intergenic non-protein coding RNA 1126 [Source:HGNC Symbol;Acc:HGNC:49275]
ENSG0000	43.09893	28.0018	42.77618	5.783336	17.2831	10.69263	37.95897	11.25302	1.74847041	0.000675	0.003452	SNORD6	11	93731502	93731574	+	73	snoRNA	small nucleolar RNA, C/D box 6 [Source:HGNC Symbol;Acc:HGNC:32703]
ENSG0000	13.34015	30.07601	22.51378	61.68892	41.84329	69.50212	21.97664	57.67811	-1.393101002	0.000677	0.003459	CATSPERB	14	91580696	91780707	-	5635	protein_cod	cation channel sperm associated auxiliary subunit beta [Source:HGNC Symbol;Acc:HGNC:20500]
ENSG0000	13.34015	22.81628	9.005513	44.33891	116.4335	16.03895	15.05398	58.93712	-1.964993998	0.000678	0.003463	MAGEA2B	X	152714586	152718607	+	2422	protein_cod	MAGE family member A2B [Source:HGNC Symbol;Acc:HGNC:19340]
ENSG0000	0	0	0	7.711115	3.638547	14.25685	0	8.535503	-5.450372887	0.000683	0.003485	AC008521.1	19	33051515	33051701	+	187	processed_	MAD2 mitotic arrest deficient-like 1 (yeast) (MAD2L1) pseudogene
ENSG0000	8.209321	7.259726	15.75965	30.84446	30.01801	46.33475	10.40956	35.73241	-1.789627054	0.000685	0.003491	IRGM	5	150846523	150900736	+	2271	protein_cod	immunity related GTPase M [Source:HGNC Symbol;Acc:HGNC:29597]
ENSG0000	4.10466	3.111311	2.251378	18.3139	27.2891	14.25685	3.155783	19.95328	-2.653165738	0.000687	0.003504	AP001266.2	11	65745729	65771585	+	464	lincRNA	novel transcript

ENSG0000	70.80539	75.70857	63.03859	29.88057	39.11438	27.62264	69.85085	32.20586	1.117585335	0.000687	0.003504	KLHDC7A	1	18480982	18486126	+	5145	protein_cod	kelch domain containing 7A [Source:HGNC Symbol;Acc:HGNC:26791]
ENSG0000	2.05233	2.074207	1.125689	22.16945	10.91564	16.03895	1.750742	16.37468	-3.214180423	0.000692	0.003527	AC107956.1	11	17228279	17229151	+	873	processed_p	ribosomal protein S2 (RPS2) pseudogene
ENSG0000	18.47097	21.77918	29.26792	52.05002	64.58421	52.57212	23.17269	56.40212	-1.288029007	0.0007	0.003563	WNT11	11	76186325	76210736	-	2401	protein_cod	Wnt family member 11 [Source:HGNC Symbol;Acc:HGNC:12776]
ENSG0000	36.94194	53.92939	30.3936	81.93059	79.1384	91.77844	40.42165	84.28248	-1.05722921	0.0007	0.003565	IPO5P1	19	23255053	23257939	-	2887	transcribed_	importin 5 pseudogene 1 [Source:HGNC Symbol;Acc:HGNC:49687]
ENSG0000	3.078495	7.259726	0	22.16945	26.37947	17.82106	3.446074	22.12333	-2.661935317	0.000702	0.00357	RF00561	1	150600539	150600659	-	121	snoRNA	
ENSG0000	0	0	0	6.747225	12.73492	5.346317	0	8.276153	-5.404978778	0.000711	0.003613	Z99716.1	22	41981304	41982217	-	515	antisense	novel transcript, antisense to SEPT3
ENSG0000	23.6018	21.77918	27.01654	70.36392	45.48184	58.80949	24.1325	58.21842	-1.271297327	0.000711	0.003613	HOXA11-AS	7	27184507	27189298	+	3128	antisense	HOXA11 antisense RNA [Source:HGNC Symbol;Acc:HGNC:24957]
ENSG0000	3.078495	4.148415	5.628445	14.45834	25.46983	27.62264	4.285118	22.51694	-2.402903417	0.000713	0.003619	KRTAP4-7	17	41084150	41085141	+	992	protein_cod	keratin associated protein 4-7 [Source:HGNC Symbol;Acc:HGNC:18898]
ENSG0000	2.05233	4.148415	10.1312	27.95279	20.92165	28.51369	5.443982	25.79604	-2.262505975	0.000721	0.003655	NLRP6	11	278365	285359	+	3207	protein_cod	NLR family pyrin domain containing 6 [Source:HGNC Symbol;Acc:HGNC:22944]
ENSG0000	2.05233	4.148415	2.251378	19.27779	21.83128	15.1479	2.817374	18.75232	-2.730364124	0.000721	0.003655	FAM96AP2	1	228114997	228115473	-	477	processed_p	family with sequence similarity 96 member A pseudogene 2 [Source:HGNC Symbol;Acc:HGNC:43862]
ENSG0000	2.05233	1.037104	2.251378	16.38612	14.55419	16.93	1.780271	15.95677	-3.170329047	0.000728	0.003687	RN7SKP78	10	6149623	6149940	+	318	misc_RNA	RNA, 7SK small nuclear pseudogene 78 [Source:HGNC Symbol;Acc:HGNC:45802]
ENSG0000	8.209321	9.333933	5.628445	29.88057	27.2891	30.2958	7.7239	29.15516	-1.910974476	0.000728	0.003689	CFP	X	47623172	47630305	-	5617	protein_cod	complement factor properdin [Source:HGNC Symbol;Acc:HGNC:8864]
ENSG0000	0	0	1.125689	10.60278	9.096368	15.1479	0.37523	11.61568	-4.932755958	0.000731	0.0037	GPR15	3	98531899	98533150	+	1252	protein_cod	G protein-coupled receptor 15 [Source:HGNC Symbol;Acc:HGNC:4469]
ENSG0000	2.05233	0	6.754134	14.45834	20.92165	26.73159	2.935488	20.70386	-2.848780077	0.000732	0.003703	COX6CP2	20	50479767	50479991	+	225	processed_p	cytochrome c oxidase subunit 6C pseudogene 2 [Source:HGNC Symbol;Acc:HGNC:16600]
ENSG0000	2.05233	3.111311	4.502756	14.45834	26.37947	18.71211	3.222132	19.84997	-2.634168086	0.000737	0.003724	AK3P5	10	32944481	32944999	+	519	processed_p	adenylate kinase 3 pseudogene 5 [Source:HGNC Symbol;Acc:HGNC:39064]
ENSG0000	3.078495	2.074207	3.377067	25.06112	18.19274	13.36579	2.843257	18.87322	-2.732917834	0.000745	0.003757	RNF2P1	9	6668999	6670008	-	1010	processed_p	ring finger protein 2 pseudogene 1 [Source:HGNC Symbol;Acc:HGNC:33987]
ENSG0000	10.26165	9.333933	5.628445	27.95279	34.5662	29.40474	8.40801	30.64124	-1.85935757	0.000756	0.003804	ECT2L	6	138795926	138904070	+	4624	protein_cod	epithelial cell transforming 2 like [Source:HGNC Symbol;Acc:HGNC:21118]
ENSG0000	13.34015	0	5.628445	19.27779	43.66257	33.86001	6.322864	32.26679	-2.3486311	0.000756	0.003817	FTH1P26	6	133676729	133677429	-	701	processed_p	ferritin heavy chain 1 pseudogene 26 [Source:HGNC Symbol;Acc:HGNC:37644]
ENSG0000	1.026165	7.259726	2.251378	20.24168	18.19274	25.84053	3.512423	21.42498	-2.603620155	0.00076	0.003824	AC010148.1	2	234834315	234913384	-	2973	processed_t	novel transcript
ENSG0000	18.47097	11.40814	12.38258	48.19447	30.92765	46.33475	14.08723	41.81896	-1.566600539	0.000763	0.003837	GFY	19	49423749	49428818	+	1876	protein_cod	golgi associated olfactory signaling regulator [Source:HGNC Symbol;Acc:HGNC:44663]
ENSG0000	19.49714	16.59366	13.50827	42.41113	44.5722	47.2258	16.53302	44.73638	-1.433061431	0.000769	0.003861	AC008267.3	7	66511556	66545066	+	712	unprocesse	GTF2I repeat domain containing 1 (GTF2IRD1) pseudogene
ENSG0000	12.31398	28.0018	20.2624	2.891668	1.819274	5.346317	20.19273	3.35242	2.585259056	0.000779	0.003907	AC011294.1	7	46673785	46759851	-	2791	lincRNA	uncharacterized LOC730338 [Source:NCBI gene;Acc:730338]
ENSG0000	7.183155	12.44524	14.63396	36.62779	34.5662	37.42422	11.42079	36.20607	-1.670300756	0.000782	0.003919	AL031123.3	6	6685148	6686984	+	1837	TEC	TEC
ENSG0000	6.15699	7.259726	3.377067	15.42223	38.20475	24.94948	5.597928	26.19215	-2.220048729	0.000784	0.003926	AC104823.1	2	195448532	195478925	+	989	lincRNA	novel transcript
ENSG0000	11.28782	11.40814	10.1312	34.70002	28.19874	42.77054	10.94239	35.2231	-1.685665491	0.000787	0.003942	ESPNL	2	238100157	238133287	+	5362	protein_cod	espin like [Source:HGNC Symbol;Acc:HGNC:27937]
ENSG0000	0	0	0	10.60278	8.186731	5.346317	0	8.045277	-5.362378809	0.000793	0.003967	AC002347.1	17	10658542	10659082	-	541	lincRNA	novel transcript
ENSG0000	2.05233	1.037104	3.377067	20.24168	23.65056	8.910529	2.1555	17.60092	-3.042058539	0.000798	0.00399	AC011379.1	5	140200163	140203187	+	3025	sense_intro	novel transcript
ENSG0000	95.43335	78.81988	56.28445	43.37502	28.19874	34.75106	76.8459	35.44161	1.120658673	0.000812	0.004053	ZNF530	19	57599885	57612722	+	5019	protein_cod	zinc finger protein 530 [Source:HGNC Symbol;Acc:HGNC:29297]
ENSG0000	58.49141	49.78098	69.79272	16.38612	32.74693	24.05843	59.35504	24.39716	1.278356949	0.000814	0.004064	HAS2-AS1	8	121639293	121993876	+	3533	processed_t	HAS2 antisense RNA 1 [Source:HGNC Symbol;Acc:HGNC:34340]
ENSG0000	16.41864	6.222622	15.75965	38.55557	38.20475	41.87948	12.8003	39.5466	-1.630778214	0.000817	0.004074	DCDC2B	1	32209094	32216196	+	1869	protein_cod	doublecortin domain containing 2B [Source:HGNC Symbol;Acc:HGNC:32576]
ENSG0000	629.0392	644.0414	594.3638	171.5723	354.7584	351.9659	622.4815	292.7655	1.087767386	0.000818	0.004081	RP2	X	46836940	46882358	+	3803	protein_cod	RP2, ARL3 GTPase activating protein [Source:HGNC Symbol;Acc:HGNC:10274]
ENSG0000	11.28782	12.44524	6.754134	30.84446	25.46983	49.00791	10.1624	35.1074	-1.783801735	0.000823	0.004099	KLRC4-KLRK	12	10372353	10410146	-	2592	protein_cod	KLRC4-KLRK1 readthrough [Source:HGNC Symbol;Acc:HGNC:48357]
ENSG0000	15.39248	17.63076	18.01103	0	1.819274	4.455264	17.01142	2.091513	3.001510382	0.000828	0.004124	AC027627.1	4	177343004	177357032	-	753	antisense	novel transcript
ENSG0000	2.05233	3.111311	1.125689	18.3139	15.46383	16.03895	2.096443	16.60556	-2.973770682	0.000843	0.004189	AC012313.3	19	58346854	58362751	-	4151	processed_t	novel transcript
ENSG0000	18.47097	14.51945	13.50827	52.05002	40.02402	37.42422	15.49956	43.16609	-1.473864526	0.000859	0.004255	IGFBP5	2	216672105	216695525	-	6442	protein_cod	insulin like growth factor binding protein 5 [Source:HGNC Symbol;Acc:HGNC:5474]
ENSG0000	20.5233	35.26153	27.01654	68.43614	52.75894	69.50212	27.60045	63.56573	-1.203504308	0.000863	0.004274	AL353194.1	20	3888239	3888868	-	441	antisense	novel transcript
ENSG0000	19.49714	19.70497	18.01103	73.25559	32.74693	49.00791	19.07104	51.67014	-1.435196805	0.000868	0.004293	KRBA2	17	8356902	8376711	-	3701	protein_cod	KRAB-A domain containing 2 [Source:HGNC Symbol;Acc:HGNC:26989]
ENSG0000	26.68029	9.333933	19.13671	0.963889	0	5.346317	18.38365	2.103402	3.111700073	0.000878	0.004336	TNNI2	11	1838989	1841680	+	1082	protein_cod	troponin I2, fast skeletal type [Source:HGNC Symbol;Acc:HGNC:11946]
ENSG0000	4.10466	21.77918	9.005513	47.23058	30.92765	43.66159	11.62978	40.60661	-1.801299432	0.00089	0.00439	AC137630.5	3	48997221	48999442	-	763	unitary_pse	novel pseudogene, ortholog of avian secreted frizzled-related protein 5
ENSG0000	16.41864	14.51945	13.50827	40.48335	40.93366	41.87948	14.81545	41.09883	-1.470371224	0.000899	0.004434	ABCC6	16	16148928	16223522	-	7569	protein_cod	ATP binding cassette subfamily C member 6 [Source:HGNC Symbol;Acc:HGNC:57]
ENSG0000	2.05233	9.333933	2.251378	19.27779	22.74092	29.40474	4.545881	23.80782	-2.381417414	0.000902	0.004444	SELENOP	5	42799880	42887392	-	5688	protein_cod	selenoprotein P [Source:HGNC Symbol;Acc:HGNC:10751]
ENSG0000	24.62796	16.59366	18.01103	54.94169	46.39148	48.11685	19.74422	49.81667	-1.332701728	0.000915	0.004501	AC112907.2	3	186826549	186827337	+	330	unprocesse	MCF.2 cell line derived transforming sequence-like 2 (MCF2L2) pseudogene
ENSG0000	21.54947	31.11311	34.89636	57.83336	67.31312	69.50212	29.18631	64.88287	-1.156583795	0.000927	0.004556	IL3RA	X	1336616	1382689	+	1710	protein_cod	interleukin 3 receptor subunit alpha [Source:HGNC Symbol;Acc:HGNC:6012]
ENSG0000	25.65413	14.51945	36.02205	53.01391	60.94567	70.39318	25.39854	61.45092	-1.281221128	0.000959	0.004697	AC093690.1	2	28307691	28310459	-	2110	antisense	uncharacterized LOC100505716 [Source:NCBI gene;Acc:100505716]
ENSG0000	14.36631	6.222622	11.25689	22.16945	39.11438	47.2258	10.61527	36.16988	-1.770969217	0.000959	0.004697	AC010913.1	2	73113018	73115907	+	2890	processed_t	novel transcript
ENSG0000	3.078495	1.037104	0	17.35001	9.096368	19.60316	1.371866	15.34985	-3.453881681	0.000979	0.004778	AC114402.2	1	203805621	203806263	-	643	processed_p	SNF7 domain containing 2 (SNF7DC20) pseudogene
ENSG0000	31.81112	37.33573	20.2624	9.638893	6.367458	8.910529	29.80308	8.305627	1.847345295	0.000986	0.004809	HEY2	6	125747664	125761269	+	2741	protein_cod	hes related family bHLH transcription factor with YRPW motif 2 [Source:HGNC Symbol;Acc:HGNC:4881]
ENSG0000	0	1.037104	1.125689	5.783336	16.37346	19.60316	0.720931	13.91999	-4.294802758	0.000992	0.004839	AC008403.3	19	48513207	48513660	-	454	lincRNA	novel transcript
ENSG0000	28.73262	33.18732	36.02205	12.53056	8.186731	9.801581	32.64733	10.17296	1.684112891	0.000994	0.004848	EDAR	2	108894471	108989372	-	4426	protein_cod	ectodysplasin A receptor [Source:HGNC Symbol;Acc:HGNC:2895]
ENSG0000	12.31398	14.51945	12.38258	45.3028	34.5662	34.75106	13.072	38.20669	-1.545438842	0.000999	0.00487	AC097059.2	1	94318479	94324569	-	592	lincRNA	novel transcript
ENSG0000	23.6018	13.48235	20.2624	47.23058	53.66857	45.4437	19.11552	48.78095	-1.352084026	0.001012	0.004925	AC064807.1	8	51899325	51947173	+	2055	antisense	novel transcript
ENSG0000	19.49714	19.70497	16.88534	45.3028	55.48785	41.87948	18.69581	47.55671	-1.345321236	0.001021	0.004966	AL662884.3	6	32152802	32154365	-	1060	bidirectiona	uncharacterized LOC100507547 [Source:NCBI gene;Acc:100507547]
ENSG0000	4.10466	3.111311	1.125689	22.16945	17.2831	15.1479	2.780553	18.20015	-2.694316395	0.001027	0.004988	AL831711.1	1	161433444	161440996	-	569	lincRNA	novel transcript
ENSG0000	47.20359	35.26153	54.03308	97.35282	61.8553	125.6385	45.4994	94.94886	-1.06370616	0.001027	0.004991	TMPRSS6	22	37065436	37109713	-	4232	protein_cod	transmembrane serine protease 6 [Source:HGNC Symbol;Acc:HGNC:16517]
ENSG0000	0	0	0	6.747225	6.367458	9.801581	0	7.638755	-5.289918584	0.001034	0.005018	AP002336.1	11	70319928	70321415	+	422	sense_intro	novel transcript
ENSG0000	0	1.037104	0	5.783336	10.006	17.82106	0.345701	11.20347	-4.882367775	0.001046	0.005073	PHYHD1	9	128920895	128942041	+	2915	protein_cod	phytanoyl-CoA dioxygenase domain containing 1 [Source:HGNC Symbol;Acc:HGNC:23396]
ENSG0000	0	0	0	5.783336	10.006	7.128423	0	7.639255	-5.290027995	0.001047	0.005073	RN7SL15P	X	41345576	41345876	-	301	misc_RNA	RNA, 7SL, cytoplasmic 15, pseudogene [Source:HGNC Symbol;Acc:HGNC:46031]
ENSG0000	7.183155	6.222622	9.005513	28.91668	40.02402	17.82106	7.47043	28.92059	-1.956037086	0.001054	0.005103	AC009955.1	2	219625059	219626693	-	271	antisense	novel transcript
ENSG0000	2.05233	0	0	18.3139	12.73492	9.801581	0.68411	13.6168	-4.2755518	0.001056	0.005107	RPL34P20	10	97393197	97393547	-	351	processed_p	ribosomal protein L34 pseudogene 20 [Source:HGNC Symbol;Acc:HGNC:35535]
ENSG0000	6.15699	6.222622	5.628445	24.09723	16.37346	36.53317	6.002686	25.66795	-2.095970999	0.001076	0.005198	AP000704.1	21	36966492	36975164	+	2266	lincRNA	novel transcript
ENSG0000	11.28782	9.333933	5.628445	21.20557	40.02402	32.96896	8.750065	31.39951	-1.838070461	0.001078	0.005204	MORF4L2-A	X	103687284	103691772	+	2086	antisense	MORF4L2 antisense RNA 1 [Source:HGNC Symbol;Acc:HGNC:27991]
ENSG0000	33.86345	31.11311	47.27894	81.93059	70.04203	77.5216	37.4185	76.49808	-1.035187528	0.001079	0.005208	ITGAX	16	31355134	31382997	+	7074	protein_cod	integrin subunit alpha X [Source:HGNC Symbol;Acc:HGNC:6152]
ENSG0000	8.209321	3.111311	2.251378	26.02501	25.46983	16.93	4.524003	22.80828	-2.320824141	0.001082	0.005218	FBXW10	17	18744024	18779349	+	4390	protein_cod	F-box and WD repeat domain containing 10 [Source:HGNC Symbol;Acc:HGNC:1211]
ENSG0000	20.5233	14.51945	10.1312	44.33891	40.02402	41.87948	15.05798	42.0808	-1.476315504	0.001091	0.005261	MIR205HG	1	209428820	209432838	+	3518	processed_t	MIR205 host gene [Source:HGNC Symbol;Acc:HGNC:43562]
ENSG0000	18.47097	23.85338	30.3936	54.94169	50.93966	64.15581	24.23932	56.67905	-1.230127662	0.001092	0.005266	AL031123.2	6	6680309	6683633	-	3325	lincRNA	novel transcript
ENSG0000	10.26165	5.185518	19.13671	31.80835	40.93366	39.20633	11.52796	37.31611	-1.706124706	0.001095	0.005275	UOX	1	84363706	84397831	-	2412	transcribed_	urate oxidase (pseudogene) [Source:HGNC Symbol;Acc:HGNC:12575]
ENSG0000	4.10466	1.037104	1.125689	10.60278	20.01201	19.60316	2.089151	16.73932	-2.990047756	0.001096	0.005279	AC008443.4	5	181191924	181194429	+	486	antisense	novel transcript
ENSG0000	16.41864	24.89049	27.01654	58.79725	47.30111	57.02738	22.77522	54.37525	-1.258514588	0.001111	0.005344	MSTN	2	190055697	190062729	-	2822	protein_cod	myostatin [Source:HGNC Symbol;Acc:HGNC:4223]
ENSG0000	0	0	0	13.49445	5.457821	4.455264	0	7.802512	-5.316733985	0.001115	0.005358	AC084116.1	8	126497385	126500440	+	1513	lincRNA	novel transcript

ENSG0000	8.209321	3.111311	2.251378	23.13334	22.74092	21.38527	4.524003	22.41984	-2.296661154	0.001118	0.005369	ATP6V0CP4	1	42952202	42952641	-	440	processed_p	ATPase H+ transporting V0 subunit c pseudogene 4 [Source:HGNC Symbol;Acc:HGNC:40002]
ENSG0000	2.05233	3.111311	0	11.56667	22.74092	13.36579	1.721214	15.89113	-3.182459503	0.001127	0.005406	CACNA1C	12	1970786	2697950	+	19103	protein_cod	calcium voltage-gated channel subunit alpha1 C [Source:HGNC Symbol;Acc:HGNC:1390]
ENSG0000	82.09321	104.7475	70.91841	56.86947	38.20475	29.40474	85.9197	41.49299	1.05496578	0.001127	0.005406	ANK1	8	41653220	41896762	-	10797	protein_cod	ankyrin 1 [Source:HGNC Symbol;Acc:HGNC:492]
ENSG0000	14.36631	20.74207	25.89085	53.01391	37.29511	65.93791	20.33308	52.08231	-1.361809983	0.001129	0.005414	AC002128.1	19	35262846	35264804	+	1016	sense_intro	novel transcript
ENSG0000	19.49714	11.40814	10.1312	22.16945	56.39748	49.89896	13.67883	42.82197	-1.643969079	0.001134	0.005431	AL137003.2	6	16764346	16766883	+	2538	lincRNA	novel transcript
ENSG0000	11.28782	5.185518	1.125689	33.73613	16.37346	32.96896	5.866341	27.69285	-2.222493768	0.001135	0.005435	ANKRD20A1	13	23939557	23946782	-	2796	transcribed_	ankyrin repeat domain 20 family member A19, pseudogene [Source:HGNC Symbol;Acc:HGNC:42737]
ENSG0000	5.130825	3.111311	4.502756	21.20557	20.01201	21.38527	4.248298	20.86761	-2.296972891	0.00114	0.005457	LINC00235	16	525155	527407	-	2253	lincRNA	long intergenic non-protein coding RNA 235 [Source:HGNC Symbol;Acc:HGNC:14138]
ENSG0000	17.44481	30.07601	30.3936	62.65281	54.57821	61.48265	25.97147	59.57122	-1.200585451	0.001151	0.005506	ZNF497	19	58354357	58362848	-	3897	protein_cod	zinc finger protein 497 [Source:HGNC Symbol;Acc:HGNC:23714]
ENSG0000	0	1.037104	2.251378	8.675004	10.91564	23.16737	1.096161	14.25267	-3.735801277	0.001156	0.005532	AL365436.2	1	151540516	151561855	+	526	sense_intro	novel transcript
ENSG0000	24.62796	28.0018	16.88534	65.54447	47.30111	53.46317	23.1717	55.43625	-1.253339508	0.001159	0.005543	CDNF	10	14819250	14838575	-	1733	protein_cod	cerebral dopamine neurotrophic factor [Source:HGNC Symbol;Acc:HGNC:24913]
ENSG0000	19.49714	8.29683	18.01103	35.6639	40.93366	53.46317	15.26833	43.35358	-1.508995092	0.001166	0.005574	PDE7B	6	135851696	136195574	+	5946	protein_cod	phosphodiesterase 7B [Source:HGNC Symbol;Acc:HGNC:8792]
ENSG0000	14.36631	8.29683	11.25689	26.9889	47.30111	32.96896	11.30668	35.75299	-1.661259741	0.001166	0.005574	AC148477.4	12	132282814	132284606	+	1793	lincRNA	novel transcript
ENSG0000	11.28782	3.111311	2.251378	33.73613	23.65056	20.49422	5.550168	25.9603	-2.21110965	0.001186	0.005659	AC090519.1	15	44354691	44355692	+	1002	processed_p	glyceraldehyde-3-phosphate dehydrogenase (GAPDH) pseudogene
ENSG0000	18.47097	31.11311	22.51378	69.40003	48.21075	53.46317	24.03262	57.02465	-1.244926734	0.001195	0.005696	PLB1	2	28457145	28643788	+	8238	protein_cod	phospholipase B1 [Source:HGNC Symbol;Acc:HGNC:30041]
ENSG0000	41.0466	36.29863	65.28997	5.783336	9.096368	28.51369	47.54507	14.46447	1.707296559	0.001206	0.00574	NMRAL2P	3	185959943	185980872	+	2628	transcribed_	NmrA like redox sensor 2, pseudogene [Source:HGNC Symbol;Acc:HGNC:52332]
ENSG0000	14.36631	23.85338	29.26792	51.08613	41.84329	76.63055	22.49587	56.51999	-1.334756655	0.001224	0.005816	SPON1	11	13962689	14267884	+	5102	protein_cod	spondin 1 [Source:HGNC Symbol;Acc:HGNC:11252]
ENSG0000	31.81112	18.66787	5.628445	2.891668	2.72891	2.673159	18.70248	2.764579	2.763448153	0.00125	0.005931	AP001029.1	18	12432897	12437635	+	168	lincRNA	novel transcript
ENSG0000	22.57563	16.59366	21.38809	4.819447	5.457821	1.782106	20.18579	4.019791	2.333287475	0.001271	0.006024	RNA5SP323	10	93510548	93510680	-	133	rRNA_pseuc	RNA, 5S ribosomal pseudogene 323 [Source:HGNC Symbol;Acc:HGNC:43223]
ENSG0000	16.41864	9.333933	6.754134	33.73613	29.10838	41.87948	10.83557	34.908	-1.680915534	0.001277	0.006052	HNRNPA1P1	9	88410529	88411463	-	935	processed_p	heterogeneous nuclear ribonucleoprotein A1 pseudogene 14 [Source:HGNC Symbol;Acc:HGNC:39132]
ENSG0000	63.62223	81.93119	72.0441	35.6639	22.74092	43.66159	72.53251	34.02214	1.091651811	0.00129	0.006103	ECM1	1	150508062	150513789	+	3360	protein_cod	extracellular matrix protein 1 [Source:HGNC Symbol;Acc:HGNC:3153]
ENSG0000	5.130825	12.44524	6.754134	37.59168	19.10237	33.86001	8.110068	30.18469	-1.893093851	0.00131	0.006188	ZNF671	19	57719751	57727624	-	3532	protein_cod	zinc finger protein 671 [Source:HGNC Symbol;Acc:HGNC:26279]
ENSG0000	8.209321	10.37104	14.63396	27.95279	46.39148	30.2958	11.07144	34.88002	-1.662222261	0.001312	0.006195	AC006001.2	7	66654538	66669855	+	938	sense_intro	uncharacterized LOC100996437 [Source:NCBI gene;Acc:100996437]
ENSG0000	20.5233	11.40814	21.38809	37.59168	48.21075	53.46317	17.77318	46.42187	-1.389096384	0.001318	0.006222	AL359510.2	1	228121523	228123771	+	2249	TEC	TEC
ENSG0000	13.34015	19.70497	5.628445	0.963889	1.819274	0	12.89119	0.927721	3.807408971	0.001337	0.006303	GNG11	7	93921699	93928610	+	3055	protein_cod	G protein subunit gamma 11 [Source:HGNC Symbol;Acc:HGNC:4403]
ENSG0000	1.026165	1.037104	0	15.42223	10.006	12.47474	0.687756	12.63432	-4.16633716	0.001358	0.006392	CALHM1	10	103453387	103458888	-	3053	protein_cod	calcium homeostasis modulator 1 [Source:HGNC Symbol;Acc:HGNC:23494]
ENSG0000	1.026165	0	1.125689	11.56667	11.82528	14.25685	0.717285	12.5496	-4.145114568	0.001379	0.006481	AC044849.1	8	30082758	30083467	+	710	antisense	novel transcript, antisense to TMEM66
ENSG0000	1.026165	0	0	14.45834	10.91564	2.673159	0.342055	11.16832	-4.873528388	0.001382	0.00649	AC010999.2	15	56887107	56888219	+	596	lincRNA	novel transcript
ENSG0000	29.75879	32.15021	40.52481	75.18337	71.86131	63.26475	34.1446	70.10314	-1.040179798	0.001392	0.006528	CDC42	1	22052627	22092946	+	3598	protein_cod	cell division cycle 42 [Source:HGNC Symbol;Acc:HGNC:1736]
ENSG0000	22.57563	38.37284	43.90187	57.83336	86.4155	80.19476	34.95011	74.81454	-1.102995633	0.001398	0.006551	ZNF713	7	55887475	55942530	+	6461	protein_cod	zinc finger protein 713 [Source:HGNC Symbol;Acc:HGNC:22043]
ENSG0000	0	3.111311	1.125689	10.60278	24.56019	9.801581	1.412333	14.98819	-3.406484462	0.001399	0.006558	AC087639.1	15	68833830	68834749	+	920	processed_p	cytokine induced apoptosis inhibitor 1 (CIAPIN1)
ENSG0000	4.10466	3.111311	4.502756	19.27779	20.92165	18.71211	3.906243	19.63718	-2.332896883	0.001401	0.006563	PEAK3	19	2274622	2282176	-	2574	protein_cod	PEAK family member 3 [Source:HGNC Symbol;Acc:HGNC:24793]
ENSG0000	1.026165	0	2.251378	17.35001	8.186731	15.1479	1.092514	13.56155	-3.662566153	0.00141	0.006597	AC010336.1	19	7898804	7903542	-	4739	antisense	novel transcript, antisense to LRRC8E
ENSG0000	18.47097	22.81628	30.3936	58.79725	52.75894	53.46317	23.89362	55.00645	-1.207109113	0.001411	0.006597	AL445584.2	9	42231811	42354454	+	616	lincRNA	novel transcript
ENSG0000	4.10466	7.259726	12.38258	44.33891	26.37947	19.60316	7.915655	30.10718	-1.935482014	0.001429	0.006678	AP000924.1	11	110355130	110406400	+	781	lincRNA	uncharacterized LOC105369486 [Source:NCBI gene;Acc:105369486]
ENSG0000	3.078495	2.074207	1.125689	17.35001	18.19274	11.58369	2.092797	15.70881	-2.894608842	0.001449	0.006759	AC004834.1	7	99252452	99325826	-	4408	antisense	novel transcript
ENSG0000	3.078495	0	2.251378	10.60278	13.64455	22.27632	1.776624	15.50789	-3.131792695	0.001451	0.006769	HEXA-AS1	15	72376113	72378788	+	2676	antisense	HEXA antisense RNA 1 [Source:HGNC Symbol;Acc:HGNC:25810]
ENSG0000	6.15699	10.37104	6.754134	43.37502	15.46383	30.2958	7.760721	29.71155	-1.933150029	0.001454	0.00678	AC106900.2	2	173811076	173811392	+	317	lincRNA	novel transcript
ENSG0000	1.026165	2.074207	3.377067	19.27779	14.55419	13.36579	2.159147	15.73259	-2.879306224	0.001474	0.006867	PDXDC2P	16	70010751	70065945	-	2707	transcribed_	pyridoxal dependent decarboxylase domain containing 2, pseudogene [Source:HGNC Symbol;Acc:HGNC:27559]
ENSG0000	9.235486	6.222622	4.502756	34.70002	19.10237	24.94948	6.653621	26.25062	-1.971702968	0.001484	0.006911	AC064836.3	2	202374962	202375604	-	673	lincRNA	novel transcript
ENSG0000	6.15699	13.48235	3.377067	21.20557	29.10838	38.31527	7.672135	29.54307	-1.937682906	0.001493	0.006947	EPHA8	1	22563564	22603594	+	5358	protein_cod	EPH receptor A8 [Source:HGNC Symbol;Acc:HGNC:3391]
ENSG0000	0	0	0	3.855557	8.186731	9.801581	0	7.28129	-5.222070765	0.001493	0.006947	AP001062.2	21	44339376	44340470	+	127	antisense	novel transcript
ENSG0000	21.54947	16.59366	21.38809	65.54447	31.83729	54.35422	19.84374	50.57866	-1.34957332	0.001519	0.007054	FLJ45513	17	49845910	49848837	+	2100	antisense	uncharacterized LOC729220 [Source:NCBI gene;Acc:729220]
ENSG0000	4.10466	0	0	14.45834	10.91564	19.60316	1.36822	14.99238	-3.423082402	0.001521	0.007059	AC092868.2	15	59348648	59359889	-	497	sense_intro	novel transcript, sense intronic to MYO1E
ENSG0000	8.209321	9.333933	10.1312	36.62779	32.74693	22.27632	9.224818	30.55035	-1.728139566	0.001528	0.007089	AL451042.1	1	16159266	16161883	+	544	antisense	novel transcript
ENSG0000	36.94194	31.11311	34.89636	6.747225	15.46383	11.58369	34.31714	11.26491	1.602573584	0.001531	0.007097	KRT32	17	41459811	41467429	-	1750	protein_cod	keratin 32 [Source:HGNC Symbol;Acc:HGNC:6449]
ENSG0000	5.130825	12.44524	13.50827	31.80835	28.19874	40.09738	10.36145	33.36816	-1.693949221	0.001536	0.007117	LINC01301	8	60384588	60516795	-	4428	lincRNA	long intergenic non-protein coding RNA 1301 [Source:HGNC Symbol;Acc:HGNC:50464]
ENSG0000	5.130825	13.48235	5.628445	28.91668	36.38547	23.16737	8.08054	29.48984	-1.863025061	0.001543	0.007146	AC009509.1	12	27696388	27710770	-	1519	antisense	novel transcript
ENSG0000	4.10466	4.148415	1.125689	19.27779	16.37346	18.71211	3.126255	18.12112	-2.520018485	0.00157	0.007253	AL137060.1	13	50125816	50128463	+	2648	sense_intro	novel transcript, sense intronic to DLEU1
ENSG0000	42.07277	43.55836	25.89085	15.42223	10.006	13.36579	37.17399	12.93134	1.527793595	0.001575	0.007273	AC016949.1	3	196912646	196914579	+	1413	sense_intro	novel transcript
ENSG0000	48.22976	30.07601	29.26792	76.14726	75.49986	69.50212	35.85789	73.71641	-1.036328024	0.001576	0.007273	AC003072.1	22	30653877	30654814	-	612	unprocesse	ribosomal protein L13a (RPL13A) pseudogene
ENSG0000	24.62796	17.63076	7.879823	37.59168	51.8493	48.11685	16.71285	45.85261	-1.448427466	0.001599	0.007369	AL160269.1	9	96235331	96352058	-	4087	protein_cod	solute carrier family 35 member D2 [Source:NCBI gene;Acc:11046]
ENSG0000	15.39248	18.01103	29.88057	50.03002	51.68107	16.66572	43.86389	-1.399418563	0.001601	0.007373	STRC	15	43599563	43618800	-	8102	protein_cod	stereocilin [Source:HGNC Symbol;Acc:HGNC:16035]	
ENSG0000	14.36631	7.259726	18.01103	1.927779	1.819274	0	13.21235	1.249017	3.416746391	0.001616	0.007431	CACNA2D1	7	81946444	82443798	-	9010	protein_cod	calcium voltage-gated channel auxiliary subunit alpha2delta 1 [Source:HGNC Symbol;Acc:HGNC:1399]
ENSG0000	1.026165	5.185518	2.251378	16.38612	19.10237	16.93	2.821021	17.47283	-2.628395047	0.001621	0.007452	CSNK1G2-AS	19	1952531	1954586	-	1346	antisense	CSNK1G2 antisense RNA 1 [Source:HGNC Symbol;Acc:HGNC:28604]
ENSG0000	8.209321	4.148415	6.754134	30.84446	20.92165	23.16737	6.370623	24.97783	-1.970329021	0.001625	0.007469	SLC5A9	1	48222685	48248644	+	4535	protein_cod	solute carrier family 5 member 9 [Source:HGNC Symbol;Acc:HGNC:22146]
ENSG0000	0	1.037104	1.125689	17.35001	9.096368	10.69263	0.720931	12.37967	-4.121147235	0.00163	0.007486	TRGV2	7	38362864	38363518	-	544	TR_V_gene	T cell receptor gamma variable 2 [Source:HGNC Symbol;Acc:HGNC:12287]
ENSG0000	21.54947	18.66787	14.63396	42.41113	42.75293	51.68107	18.28376	45.61504	-1.316045251	0.001632	0.007494	AC135506.1	3	49140086	49160851	-	1799	unprocesse	laminin, beta 1 (LAMB1) pseudogene
ENSG0000	0	0	0	9.638893	7.277095	4.455264	0	7.123751	-5.186704862	0.001632	0.007494	PKIA-AS1	8	78426103	78558503	-	2656	lincRNA	PKIA antisense RNA 1 [Source:HGNC Symbol;Acc:HGNC:51659]
ENSG0000	7.183155	11.40814	9.005513	0.963889	0	0	9.198936	0.321296	4.801017265	0.001645	0.007548	HMMR-AS1	5	163483065	163494058	-	957	antisense	HMMR antisense RNA 1 [Source:HGNC Symbol;Acc:HGNC:49149]
ENSG0000	31.81112	42.52125	25.89085	49.15836	79.1384	89.10529	33.40774	72.46735	-1.116168873	0.001647	0.007556	AC097478.3	4	89748283	89749154	+	872	antisense	novel transcript, antisense to SNCA
ENSG0000	0	0	0	10.60278	4.548184	6.23737	0	7.129446	-5.187701461	0.001664	0.007622	RNF113B	13	98175785	98177265	-	1376	protein_cod	ring finger protein 113B [Source:HGNC Symbol;Acc:HGNC:17267]
ENSG0000	13.34015	25.92759	20.2624	54.94169	40.40402	52.57212	19.84338	49.17928	-1.309873928	0.00167	0.007645	LINC00640	14	51333393	51365557	+	1568	lincRNA	long intergenic non-protein coding RNA 640 [Source:HGNC Symbol;Acc:HGNC:44291]
ENSG0000	33.86345	47.70677	29.26792	80.00281	68.22276	75.73949	36.94604	74.65502	-1.011775215	0.001693	0.007746	AL357033.2	20	62732566	62735347	-	739	antisense	novel transcript
ENSG0000	6.15699	3.111311	1.125689	20.24168	20.01201	16.93	3.464664	19.06123	-2.443114562	0.001713	0.007816	C5AR2	19	47332147	47345227	+	6802	protein_cod	complement component 5a receptor 2 [Source:HGNC Symbol;Acc:HGNC:4527]
ENSG0000	4.10466	3.111311	2.251378	23.13334	20.92165	10.69263	3.155783	18.24921	-2.522926732	0.001716	0.00783	AC024270.4	15	83012661	83061845	+	421	antisense	novel transcript, antisense to BTBD1
ENSG0000	3.078495	4.148415	13.50827	25.06112	25.46983	31.18685	6.911726	27.23927	-1.997046313	0.001723	0.007858	CNGA4	11	6234765	6244429	+	2167	protein_cod	cyclic nucleotide gated channel alpha 4 [Source:HGNC Symbol;Acc:HGNC:2152]
ENSG0000	0	3.111311	0	19.27779	9.096368	12.47474	1.037104	13.6163	-3.689957929	0.001725	0.007863	AC131392.2	5	69898867	69903198	-	4332	unprocesse	POM121 membrane glycoprotein-like (POM121L1) pseudogene
ENSG0000	16.41864	14.51945	11.25689	41.44724	26.37947	51.68107	14.06499	39.83592	-1.498813996	0.00174	0.007926	NOG	17	56593699	56595590	+	1892	protein_cod	noggin [Source:HGNC Symbol;Acc:HGNC:7866]
ENSG0000	5.130825	4.148415	0	18.3139	28.19874	11.58369	3.09308	19.36544	-2.624065191	0.001759	0.007994	AC234782.3	X	103910305	103911352	-	1048	processed_p	E74-like factor 2 (ELF2) pseudogene

ENSG0000	6.15699	8.29683	6.754134	32.77224	13.64455	35.64211	7.069318	27.35297	-1.951168236	0.001776	0.008051	LINC02600	4	3758748	3763390	-	4525	lincRNA	long intergenic non-protein coding RNA 2600 [Source:HGNC Symbol;Acc:HGNC:53177]
ENSG0000	10.26165	18.66787	18.01103	41.44724	37.29511	45.4437	15.64685	41.39535	-1.406874579	0.001779	0.008063	RASA4	7	102573807	102616757	-	8077	protein_cod	RAS p21 protein activator 4 [Source:HGNC Symbol;Acc:HGNC:23181]
ENSG0000	7.183155	5.185518	3.377067	23.13334	16.37346	28.51369	5.24858	22.6735	-2.103486303	0.001807	0.008175	AC124319.1	17	80315651	80316633	+	653	sense_intro	novel transcript
ENSG0000	3.078495	2.074207	0	20.24168	8.186731	16.93	1.717568	15.11947	-3.110478676	0.001811	0.008187	ENPP7P5	12	8254527	8308855	-	1031	unprocesse	ectonucleotide pyrophosphatase/phosphodiesterase 7 pseudogene 5 [Source:HGNC Symbol;Acc:HGNC:48688]
ENSG0000	0	0	2.251378	12.53056	11.82528	12.47474	0.750459	12.27686	-4.096734576	0.001811	0.008188	BX537318.2	22	46006616	46010777	+	4162	TEC	dynein heavy chain -like pseudogene [Source:NCBI gene;Acc:730668]
ENSG0000	4.10466	5.185518	4.502756	31.80835	15.46383	16.93	4.597645	21.40073	-2.216043513	0.001823	0.008238	ZKSCAN7	3	44555193	44594173	+	4208	protein_cod	zinc finger with KRAB and SCAN domains 7 [Source:HGNC Symbol;Acc:HGNC:12955]
ENSG0000	12.31398	14.51945	11.25689	37.59168	30.01801	40.09738	12.69677	35.90236	-1.497734469	0.001834	0.008281	AL139424.2	1	10395416	10397432	+	1477	lincRNA	novel transcript
ENSG0000	6.15699	3.111311	4.502756	17.35001	12.73492	36.53317	4.590353	22.20603	-2.274854601	0.00185	0.008348	MAFA-AS1	8	143417679	143419150	+	417	antisense	MAFA antisense RNA 1 [Source:HGNC Symbol;Acc:HGNC:51203]
ENSG0000	27.70646	24.89049	40.52481	72.2917	43.66257	92.6695	31.04058	69.54125	-1.167902704	0.001872	0.00844	EYS	6	63719980	65707225	-	17070	protein_cod	eyes shut homolog (Drosophila) [Source:HGNC Symbol;Acc:HGNC:21555]
ENSG0000	17.44481	16.59366	28.14223	43.37502	49.12039	56.13633	20.7269	49.54391	-1.263741323	0.001917	0.008617	LRRC37A6P	10	27246350	27259455	-	5625	transcribed_	leucine rich repeat containing 37 member A6, pseudogene [Source:HGNC Symbol;Acc:HGNC:33746]
ENSG0000	22.57563	5.185518	22.51378	2.891668	2.72891	1.782106	16.75831	2.467561	2.765777801	0.00192	0.008627	MAB21L4	2	240886048	240896889	-	2772	protein_cod	mab-21 like 4 [Source:HGNC Symbol;Acc:HGNC:26216]
ENSG0000	13.34015	23.85338	30.3936	54.94169	40.93366	67.72002	22.52905	54.53179	-1.281100678	0.001929	0.008665	ACSBG2	19	6135247	6193094	+	3447	protein_cod	acyl-CoA synthetase bubblegum family member 2 [Source:HGNC Symbol;Acc:HGNC:24174]
ENSG0000	1.026165	5.185518	0	16.38612	10.006	23.16737	2.070561	16.51983	-2.975886121	0.001931	0.008674	AL008582.1	22	41430934	41431375	+	442	lincRNA	novel transcript
ENSG0000	0	0	0	3.855557	6.367458	10.69263	0	6.971883	-5.159557627	0.001935	0.008692	C19orf67	19	14081619	14085875	-	1540	protein_cod	chromosome 19 open reading frame 67 [Source:HGNC Symbol;Acc:HGNC:34354]
ENSG0000	7.183155	13.48235	16.88534	23.13334	41.84329	48.11685	12.51695	37.69783	-1.59879084	0.001938	0.008701	AC005592.1	5	142703782	142705421	+	1640	lincRNA	novel transcript
ENSG0000	11.28782	8.29683	5.628445	25.06112	33.65656	26.73159	8.404364	28.48309	-1.754722323	0.001979	0.008867	AL080250.1	6	75285014	75297003	+	1519	lincRNA	uncharacterized LOC100506804 [Source:NCBI gene;Acc:100506804]
ENSG0000	10.26165	11.40814	14.63396	42.41113	32.74693	29.40474	12.10125	34.85427	-1.528639973	0.001988	0.008893	MADCAM1	19	489176	505342	+	2651	protein_cod	mucosal vascular addressin cell adhesion molecule 1 [Source:HGNC Symbol;Acc:HGNC:6765]
ENSG0000	1.026165	4.148415	3.377067	13.49445	22.74092	15.1479	2.850549	17.12776	-2.59369267	0.001991	0.008901	GJA1	6	121435577	121449727	+	3413	protein_cod	gap junction protein alpha 1 [Source:HGNC Symbol;Acc:HGNC:4274]
ENSG0000	27.70646	20.74207	37.14774	60.72503	66.40349	57.02738	28.53209	61.3853	-1.109585604	0.001999	0.008938	SLC9A2	2	102619707	102711318	+	5984	protein_cod	solute carrier family 9 member A2 [Source:HGNC Symbol;Acc:HGNC:11072]
ENSG0000	0	1.037104	2.251378	15.42223	11.82528	10.69263	1.096161	12.64671	-3.559948099	0.002002	0.008945	RPL21P3	20	18485265	18486052	+	478	processed_p	ribosomal protein L21 pseudogene 3 [Source:HGNC Symbol;Acc:HGNC:16533]
ENSG0000	10.26165	3.111311	3.377067	15.42223	22.74092	35.64211	5.583343	24.60175	-2.132718244	0.002016	0.008999	AC006064.3	12	6532290	6533498	-	477	antisense	novel transcript
ENSG0000	13.34015	5.185518	5.628445	17.35001	35.47584	35.64211	8.05137	29.48932	-1.868520731	0.002021	0.009013	AC017104.3	2	231452195	231453153	+	568	lincRNA	novel transcript
ENSG0000	0	4.148415	6.754134	19.27779	24.56019	16.03895	3.634183	19.95898	-2.476911062	0.002025	0.009031	AP000640.1	11	59752578	59754975	-	1962	processed_t	novel transcript
ENSG0000	3.078495	0	1.125689	11.56667	9.096368	21.38527	1.401395	14.0161	-3.314640916	0.002027	0.009035	RF00019	13	85264296	85264402	+	107	misc_RNA	
ENSG0000	22.57563	21.77918	3.377067	40.48335	57.30712	43.66159	15.91063	47.15069	-1.557038375	0.002027	0.009035	HS6ST3	13	96090839	96839562	+	7806	protein_cod	heparan sulfate 6-O-sulfotransferase 3 [Source:HGNC Symbol;Acc:HGNC:19134]
ENSG0000	10.26165	2.074207	16.88534	35.6639	27.2891	38.31527	9.740398	33.75609	-1.804088957	0.002027	0.009035	CDC42-IT1	1	22059197	22064199	+	692	sense_intro	CDC42 intronic transcript 1 [Source:HGNC Symbol;Acc:HGNC:41317]
ENSG0000	10.26165	30.07601	21.38809	36.62779	59.12639	62.3737	20.57525	52.7093	-1.359971322	0.002031	0.009048	IGF2BP2-AS	3	185712528	185729787	+	3415	antisense	IGF2BP2 antisense RNA 1 [Source:HGNC Symbol;Acc:HGNC:32674]
ENSG0000	38.99427	14.51945	16.88534	67.47225	50.93966	54.35422	23.46635	57.58871	-1.289781217	0.002031	0.009048	AC008537.2	19	40831221	40837210	-	482	lincRNA	novel transcript
ENSG0000	74.91005	65.33753	46.15325	33.73613	17.2831	32.0779	62.13361	27.69904	1.168916502	0.002032	0.009051	AC069499.1	3	108543367	108543875	-	509	processed_p	ribosomal protein L13 (RPL13) pseudogene
ENSG0000	13.34015	11.40814	16.88534	2.891668	0.909637	1.782106	13.87787	1.861137	2.906233904	0.002036	0.009067	AC104561.1	8	23458601	23484971	+	630	lincRNA	novel transcript
ENSG0000	27.70646	25.92759	46.15325	72.2917	73.68058	61.48265	33.26243	69.15164	-1.060953127	0.002041	0.009086	PDZD2	5	31639410	32110931	+	15642	protein_cod	PDZ domain containing 2 [Source:HGNC Symbol;Acc:HGNC:18486]
ENSG0000	9.235486	4.148415	19.13671	35.6639	31.83729	36.53317	10.8402	34.67812	-1.689786103	0.002042	0.009086	ADAM20P1	14	70453541	70483775	-	3733	transcribed_	ADAM metallopeptidase domain 20 pseudogene 1 [Source:HGNC Symbol;Acc:HGNC:20102]
ENSG0000	20.5233	17.63076	28.14223	41.44724	61.8553	51.68107	22.09876	51.6612	-1.230269605	0.002045	0.009097	CAVIN2	2	191834302	191847255	-	3229	protein_cod	caveolae associated protein 2 [Source:HGNC Symbol;Acc:HGNC:10690]
ENSG0000	7.183155	1.037104	1.125689	15.42223	21.83128	18.71211	3.115316	18.65521	-2.566245266	0.002059	0.009154	IBA57-DT	1	228164086	228165512	-	641	lincRNA	IBA57 divergent transcript [Source:HGNC Symbol;Acc:HGNC:32062]
ENSG0000	17.44481	7.259726	4.502756	27.95279	31.83729	38.31527	9.735763	32.70178	-1.738740679	0.00206	0.009154	DCAF4L1	4	41981696	41986467	+	4772	protein_cod	DDB1 and CUL4 associated factor 4 like 1 [Source:HGNC Symbol;Acc:HGNC:27723]
ENSG0000	0	0	0	2.891668	8.186731	9.801581	0	6.959994	-5.157374511	0.002063	0.009165	RNVU1-3	1	148402715	148402875	+	161	snRNA	RNA, variant U1 small nuclear 3 [Source:HGNC Symbol;Acc:HGNC:48311]
ENSG0000	10.26165	12.44524	23.63947	46.26669	35.47584	42.77054	15.44879	41.50435	-1.434028983	0.002077	0.009226	AC015813.4	17	57955965	57956143	+	179	lincRNA	novel transcript
ENSG0000	8.209321	13.48235	9.005513	33.73613	30.92765	29.40474	10.23239	31.35617	-1.613459801	0.002079	0.00923	PCDH19	X	100291644	100410273	-	9756	protein_cod	protocadherin 19 [Source:HGNC Symbol;Acc:HGNC:14270]
ENSG0000	1.026165	6.222622	0	17.35001	14.55419	19.60316	2.416262	17.16912	-2.809279837	0.002086	0.009257	RNU6-415P	15	78898840	78898936	-	97	snRNA	RNA, U6 small nuclear 415, pseudogene [Source:HGNC Symbol;Acc:HGNC:47378]
ENSG0000	36.94194	37.33573	14.63396	9.638893	4.548184	10.69263	29.63721	8.293237	1.841499884	0.002099	0.009309	TUBBP1	8	30351873	30353518	+	1646	transcribed_	tubulin beta pseudogene 1 [Source:HGNC Symbol;Acc:HGNC:12414]
ENSG0000	55.41291	89.19092	66.41565	46.26669	29.10838	22.27632	70.33983	32.55046	1.116038318	0.002106	0.009332	ZNF658B	9	39443815	39508885	-	3897	transcribed_	zinc finger protein 658B (pseudogene) [Source:HGNC Symbol;Acc:HGNC:32033]
ENSG0000	12.31398	21.77918	22.51378	69.40003	26.37947	55.24528	18.86898	50.34159	-1.417492284	0.002109	0.009345	LRRC71	1	156920650	156933094	+	2631	protein_cod	leucine rich repeat containing 71 [Source:HGNC Symbol;Acc:HGNC:26556]
ENSG0000	40.02044	60.15201	51.78147	21.20557	20.92165	24.05843	50.65138	22.06188	1.19794591	0.002117	0.009374	SCG5	15	32641676	32697098	+	2196	protein_cod	secretogranin V [Source:HGNC Symbol;Acc:HGNC:10816]
ENSG0000	45.15126	21.77918	40.52481	45.3028	98.24078	95.34266	35.81842	79.62874	-1.155383738	0.00213	0.009424	AC002553.1	17	16040472	16041273	+	802	antisense	novel transcript, antisense to NCOR1
ENSG0000	5.130825	12.44524	9.005513	0.963889	0	0	8.860527	0.321296	4.746561523	0.002152	0.00951	STXBP6	14	24809656	25050297	-	8067	protein_cod	syntaxin binding protein 6 [Source:HGNC Symbol;Acc:HGNC:19666]
ENSG0000	0	0	0	4.819447	7.277095	8.019476	0	6.705339	-5.102377112	0.002173	0.009598	RNVU1-6	1	146052081	146052244	-	164	snRNA	RNA, variant U1 small nuclear 6 [Source:HGNC Symbol;Acc:HGNC:48314]
ENSG0000	0	0	0	6.747225	4.548184	8.910529	0	6.735313	-5.107940347	0.002173	0.009598	RGN	X	47078355	47093314	+	2811	protein_cod	regucalcin [Source:HGNC Symbol;Acc:HGNC:9989]
ENSG0000	9.235486	7.259726	9.005513	25.06112	31.83729	26.73159	8.500241	27.87667	-1.714808682	0.002179	0.009617	MIR4324	19	49308797	49308868	-	72	miRNA	microRNA 4324 [Source:HGNC Symbol;Acc:HGNC:38392]
ENSG0000	19.49714	9.333933	18.01103	0	4.548184	1.782106	15.61403	2.110097	2.872854822	0.002179	0.009618	AC093157.2	1	100995473	100996260	+	671	antisense	novel transcript
ENSG0000	1.026165	0	6.754134	14.45834	14.55419	24.05843	2.593433	17.69032	-2.806796733	0.002181	0.009623	AL008727.1	16	898967	905224	-	4728	sense_intro	novel transcript, intronic to LMF1
ENSG0000	0	8.29683	3.377067	17.35001	19.10237	26.73159	3.891299	21.06132	-2.437186422	0.002185	0.009632	RN7SKP69	20	17576206	17576510	-	305	misc_RNA	RNA, 7SK small nuclear pseudogene 69 [Source:HGNC Symbol;Acc:HGNC:45793]
ENSG0000	8.209321	5.185518	23.63947	41.44724	30.01801	44.55264	12.34477	38.67263	-1.66094118	0.00219	0.009653	AC022364.1	12	28163298	28190738	-	2728	antisense	novel transcript, antisense to CCDC91
ENSG0000	1.026165	0	0	6.747225	11.82528	9.801581	0.342055	9.458028	-4.63671742	0.002199	0.009689	AC007663.3	22	20320739	20321203	+	465	lincRNA	novel transcript
ENSG0000	11.28782	35.26153	14.63396	42.41113	54.57821	61.48265	20.39443	52.824	-1.371126544	0.0022	0.009689	RDH16	12	56951431	56959374	-	3464	protein_cod	retinol dehydrogenase 16 [Source:HGNC Symbol;Acc:HGNC:29674]
ENSG0000	30.78495	21.77918	46.15325	61.68892	60.03603	89.99634	32.90579	70.57376	-1.106843843	0.002201	0.009694	AL590627.1	9	137776539	137789235	-	5276	transcribed_	forkhead box H1 (FOXH1) pseudogene
ENSG0000	2.05233	4.148415	3.377067	9.638893	20.01201	24.05843	3.192604	17.90311	-2.49209925	0.002222	0.009774	GAPDHP55	15	44063511	44064313	+	803	processed_p	glyceraldehyde 3 phosphate dehydrogenase pseudogene 55 [Source:HGNC Symbol;Acc:HGNC:38561]
ENSG0000	23.6018	20.74207	29.26792	61.68892	56.39748	45.4437	24.53726	54.51003	-1.153470959	0.002234	0.009823	SDR42E2	16	22165583	22191754	+	2630	protein_cod	short chain dehydrogenase/reductase family 42E, member 2 [Source:HGNC Symbol;Acc:HGNC:35414]
ENSG0000	88.2502	68.44884	85.55237	48.19447	48.21075	22.27632	80.75047	39.56051	1.031871727	0.002244	0.009856	CCDC15	11	124954121	125041489	+	4823	protein_cod	coiled-coil domain containing 15 [Source:HGNC Symbol;Acc:HGNC:25798]
ENSG0000	0	0	0	15.42223	6.367458	0.891053	0	7.560247	-5.270379158	0.002248	0.009872	AC090220.1	18	35595901	35597599	+	1699	sense_intro	novel transcript
ENSG0000	0	0	1.125689	15.42223	5.457821	8.019476	0.37523	9.633175	-4.659559915	0.00225	0.00988	CRTC3-AS1	15	90620007	90717141	-	957	antisense	CRTC3 antisense RNA 1 [Source:HGNC Symbol;Acc:HGNC:51433]
ENSG0000	8.209321	15.55656	12.38258	39.51946	28.19874	36.53317	12.04949	34.75046	-1.528595689	0.002271	0.009958	DBH	9	133635348	133659344	+	3776	protein_cod	dopamine beta-hydroxylase [Source:HGNC Symbol;Acc:HGNC:2689]
ENSG0000	20.5233	11.40814	28.14223	35.6639	57.30712	57.02738	20.02456	49.99947	-1.327130586	0.002285	0.010007	CLMP	11	123069865	123195281	-	5097	protein_cod	CXADR like membrane protein [Source:HGNC Symbol;Acc:HGNC:24039]
ENSG0000	3.078495	0	1.125689	10.60278	17.2831	12.47474	1.401395	13.45354	-3.254857115	0.002306	0.010089	RN7SL812P	22	20481935	20482213	-	279	misc_RNA	RNA, 7SL, cytoplasmic 812, pseudogene [Source:HGNC Symbol;Acc:HGNC:46828]
ENSG0000	22.57563	11.40814	18.01103	61.68892	50.93966	26.73159	17.3316	46.45339	-1.420429394	0.002309	0.010101	FRAT1	10	97319267	97321915	+	2649	protein_cod	FRAT1, WNT signaling pathway regulator [Source:HGNC Symbol;Acc:HGNC:3944]
ENSG0000	3.078495	2.074207	3.377067	18.3139	10.91564	20.49422	2.843257	16.57458	-2.547184147	0.002323	0.010157	AF274858.1	X	153599315	153613937	+	5206	transcribed_	uncharacterized LOC105373383 [Source:NCBI gene;Acc:105373383]
ENSG0000	31.81112	31.11311	27.01654	59.76114	66.40349	58.80949	29.98026	61.65804	-1.038773382	0.002327	0.01017	UBAC2-AS1	13	99196377	99200710	-	1771	lincRNA	UBAC2 antisense RNA 1 [Source:HGNC Symbol;Acc:HGNC:42502]
ENSG0000	0	0	0	3.855557	7.277095	8.910529	0	6.68106	-5.097791312	0.002339	0.010212	AC027644.3	7	66739829	66740385	-	557	antisense	novel transcript, antisense to RABGEF1
ENSG0000	1.026165	5.185518	4.502756	19.27779	20.01201	16.03895	3.57148	18.44292	-2.375455267	0.002352	0.010263	MIR5702	2	226658710	226658793	-	84	miRNA	microRNA 5702 [Source:HGNC Symbol;Acc:HGNC:43503]
ENSG0000	51.30825	122.3782	56.28445	36.62779	31.83729	36.53317	76.65698	34.99942	1.132624403	0.00236	0.010296	GK	X	30653359	30731456	+	5359	protein_cod	glycerol kinase [Source:HGNC Symbol;Acc:HGNC:4289]

ENSG0000	22.57563	11.40814	27.01654	59.76114	34.5662	57.91844	20.33344	50.74859	-1.32322101	0.002362	0.0103	AC006252.1	3	52239258	52241097	+	1101	antisense	uncharacterized LOC101929054 [Source:NCBI gene;Acc:101929054]
ENSG0000	24.62796	14.51945	30.3936	52.05002	50.93966	56.13633	23.18034	53.04201	-1.198802943	0.002363	0.0103	MLANA	9	5890802	5910606	+	3083	protein_cod	melan-A [Source:HGNC Symbol;Acc:HGNC:7124]
ENSG0000	0	2.074207	0	14.45834	11.82528	8.910529	0.691402	11.73138	-4.058170323	0.0024	0.010446	LYVE1	11	10556966	10611689	-	3515	protein_cod	lymphatic vessel endothelial hyaluronan receptor 1 [Source:HGNC Symbol;Acc:HGNC:14687]
ENSG0000	0	0	1.125689	11.56667	9.096368	7.128423	0.37523	9.263821	-4.604315789	0.002412	0.010491	AC013640.1	15	36641186	36669097	-	565	antisense	novel transcript, antisense to C15orf41
ENSG0000	5.130825	21.77918	21.38809	52.05002	42.75293	39.20633	16.09937	44.66976	-1.477115766	0.002426	0.010546	AC105339.3	15	82744223	82750289	+	1091	antisense	novel transcript, antisense FSD2
ENSG0000	0	0	6.754134	18.3139	17.2831	15.1479	2.251378	16.91497	-2.95492735	0.002479	0.010747	FTH1P3	2	27392784	27393367	-	584	processed_p	ferritin heavy chain 1 pseudogene 3 [Source:HGNC Symbol;Acc:HGNC:3990]
ENSG0000	3.078495	1.037104	1.125689	17.35001	4.548184	23.16737	1.747096	15.02186	-3.093656292	0.002484	0.010767	AL356057.1	6	75610958	75611945	-	988	transcribed_	LSM14A, SCD6 homolog A (S. cerevisiae) (LSM14A) pseudogene
ENSG0000	10.26165	10.37104	11.25689	0	1.819274	0	10.62986	0.606425	4.118330831	0.002523	0.010923	SDR16C5	8	56300010	56320776	-	3279	protein_cod	short chain dehydrogenase/reductase family 16C member 5 [Source:HGNC Symbol;Acc:HGNC:30311]
ENSG0000	3.078495	2.074207	4.502756	13.49445	19.10237	18.71211	3.218486	17.10298	-2.419738128	0.002535	0.010965	AC108704.1	12	128826836	128827579	+	744	lincRNA	novel transcript
ENSG0000	21.54947	41.48415	51.7817	12.53056	10.91564	16.93	38.27177	13.45874	1.503347266	0.002544	0.011004	IFI44L	1	78619922	78646145	+	10240	protein_cod	interferon induced protein 44 like [Source:HGNC Symbol;Acc:HGNC:17817]
ENSG0000	3.078495	1.037104	2.251378	7.711115	15.46383	22.27632	2.122326	15.15042	-2.838361564	0.002546	0.011008	AL592295.3	1	161403409	161470523	-	863	lincRNA	novel transcript
ENSG0000	7.183155	6.222622	9.005513	18.3139	21.83128	40.09738	7.47043	26.74752	-1.845996753	0.00255	0.011022	AC072052.1	7	6490793	6491098	-	306	processed_p	ribosomal protein L31 (RPL31) pseudogene
ENSG0000	15.39248	8.29683	20.2624	32.77224	33.65656	54.35422	14.65057	40.26101	-1.465359158	0.002566	0.011083	AP002381.2	11	72793624	72814054	-	1299	lincRNA	Novel transcript
ENSG0000	19.49714	42.52125	15.75965	62.65281	47.30111	73.95739	25.92601	61.30377	-1.237428649	0.002588	0.011173	NLRC4	2	32224453	32265854	-	3624	protein_cod	NLR family CARD domain containing 4 [Source:HGNC Symbol;Acc:HGNC:16412]
ENSG0000	17.44481	6.222622	19.13671	37.59168	39.11438	40.98843	14.26805	39.2315	-1.464210671	0.0026	0.011219	MT-TG	MT	9991	10058	+	68	Mt_tRNA	mitochondrially encoded tRNA glycine [Source:HGNC Symbol;Acc:HGNC:7486]
ENSG0000	21.54947	15.55656	13.50827	44.33891	37.29511	44.55264	16.87143	42.06222	-1.314071476	0.002601	0.011221	DENND5B-A	12	31589923	31615666	+	1308	antisense	DENND5B antisense RNA 1 [Source:HGNC Symbol;Acc:HGNC:42517]
ENSG0000	1.026165	0	0	14.45834	8.186731	5.346317	0.342055	9.330463	-4.613330478	0.002604	0.01123	AL031432.2	1	25266102	25267136	-	459	unprocesse	novel pseudogene
ENSG0000	11.28782	3.111311	2.251378	24.09723	20.92165	25.84053	5.550168	23.6198	-2.076481962	0.002612	0.011251	CEACAM22F	19	44537058	44620821	-	3170	transcribed_	carcinoembryonic antigen related cell adhesion molecule 22, pseudogene [Source:HGNC Symbol;Acc:HGNC:38029]
ENSG0000	6.15699	0	0	25.06112	17.2831	8.910529	2.05233	17.08492	-3.029386695	0.002614	0.01126	PRR5L	11	36296288	36465204	+	6616	protein_cod	proline rich 5 like [Source:HGNC Symbol;Acc:HGNC:25878]
ENSG0000	31.81112	29.0389	30.3936	77.11115	49.12039	63.26475	30.41454	63.16543	-1.05312389	0.002625	0.011295	MMP25	16	3046681	3060726	+	4126	protein_cod	matrix metallopeptidase 25 [Source:HGNC Symbol;Acc:HGNC:14246]
ENSG0000	0	2.074207	2.251378	10.60278	18.19274	10.69263	1.441862	13.16272	-3.208570536	0.002628	0.011307	PCDHB12	5	141208697	141212571	+	3875	protein_cod	protocadherin beta 12 [Source:HGNC Symbol;Acc:HGNC:8683]
ENSG0000	13.34015	7.259726	13.50827	20.24168	36.38547	47.2258	11.36938	34.61765	-1.61125123	0.00263	0.01131	AC145285.2	16	28802743	28817828	+	1963	lincRNA	novel transcript
ENSG0000	9.235486	7.259726	18.01103	26.9889	30.92765	45.4437	11.50208	34.45342	-1.593468231	0.002633	0.011321	AC007114.2	17	57092145	57096425	-	4281	antisense	novel transcript, antisense to AKAP1
ENSG0000	25.65413	21.77918	19.13671	47.23058	47.30111	55.24528	22.19001	49.92566	-1.167961801	0.002641	0.011351	AC079781.5	7	97851688	97972985	-	4295	processed_t	novel transcript
ENSG0000	1.026165	0	0	3.855557	12.73492	11.58369	0.342055	9.391387	-4.627790484	0.002642	0.011355	AC012640.1	5	10248325	10249915	+	688	antisense	novel transcript
ENSG0000	1.026165	2.074207	0	7.711115	12.73492	16.03895	1.033457	12.16166	-3.531973166	0.002657	0.011406	AL449403.1	9	96095211	96101912	+	1577	lincRNA	novel transcript
ENSG0000	19.49714	26.9647	19.13671	75.18337	35.47584	47.2258	21.86618	52.62833	-1.263596779	0.002658	0.011408	AC084036.1	3	156523740	156524247	-	508	antisense	novel transcript, antisense to KCNAB1
ENSG0000	3.078495	3.111311	2.251378	12.53056	14.55419	21.38527	2.813728	16.15667	-2.518428051	0.00266	0.011414	AL359317.2	14	69183020	69214092	-	2364	lincRNA	novel transcript
ENSG0000	7.183155	3.111311	10.1312	29.88057	12.73492	37.42422	6.808556	26.6799	-1.978406877	0.002665	0.011428	EZR-AS1	6	158817979	158822252	+	362	antisense	EZR antisense RNA 1 [Source:HGNC Symbol;Acc:HGNC:40609]
ENSG0000	8.209321	13.48235	9.005513	28.91668	42.75293	23.16737	10.23239	31.61233	-1.625750058	0.002668	0.01144	AC068790.5	12	123969990	123970344	-	355	sense_intro	novel transcript, sense intronic to CCDC92
ENSG0000	0	0	0	7.711115	4.548184	7.128423	0	6.462574	-5.047335104	0.00267	0.011445	LINC01722	20	12865202	12952519	-	3033	lincRNA	long intergenic non-protein coding RNA 1722 [Source:HGNC Symbol;Acc:HGNC:52510]
ENSG0000	10.26165	13.48235	27.01654	44.33891	37.29511	49.89896	16.92018	43.84433	-1.383145293	0.002671	0.01145	KCNQ3	8	132120858	132481019	-	12646	protein_cod	potassium voltage-gated channel subfamily Q member 3 [Source:HGNC Symbol;Acc:HGNC:6297]
ENSG0000	0	1.037104	0	6.747225	17.2831	4.455264	0.345701	9.495196	-4.641578883	0.002706	0.011589	RANBP3L	5	36248434	36302114	-	3363	protein_cod	RAN binding protein 3 like [Source:HGNC Symbol;Acc:HGNC:26353]
ENSG0000	6.15699	11.40814	14.63396	29.88057	32.74693	32.96896	10.73303	31.86548	-1.577316625	0.00273	0.011679	AC021321.1	8	67343975	67345087	+	1113	lincRNA	novel transcript
ENSG0000	8.209321	5.185518	10.1312	21.20557	21.83128	38.31527	7.842014	27.11737	-1.796537994	0.002731	0.011681	AL031432.5	1	25239494	25240253	+	760	lincRNA	novel transcript
ENSG0000	4.10466	7.259726	4.502756	11.56667	25.46983	30.2958	5.289047	22.4441	-2.08564309	0.002732	0.011685	DOCK9-DT	13	99087819	99088625	+	807	antisense	DOCK9 divergent transcript [Source:HGNC Symbol;Acc:HGNC:43696]
ENSG0000	0	0	0	9.638893	6.367458	3.564211	0	6.523521	-5.059206081	0.002757	0.01177	AC008147.3	12	50217662	50218343	-	354	transcribed_	40S ribosomal protein S26 (RPS26) pseudogene
ENSG0000	4.10466	8.29683	2.251378	12.53056	24.56019	28.51369	4.884289	21.86815	-2.155252218	0.002769	0.011818	TOX	8	58805418	59119208	-	4131	protein_cod	thymocyte selection associated high mobility group box [Source:HGNC Symbol;Acc:HGNC:18988]
ENSG0000	21.54947	24.89049	24.76516	40.48335	50.03002	69.50212	23.73504	53.3385	-1.170686981	0.002777	0.011847	PRKAR2A-AS	3	48847572	48851981	+	3917	antisense	PRKAR2A antisense RNA 1 [Source:HGNC Symbol;Acc:HGNC:40471]
ENSG0000	2.05233	10.37104	6.754134	24.09723	27.2891	22.27632	6.3925	24.55422	-1.944021852	0.002807	0.011953	TRIM31	6	30102897	30113106	-	3535	protein_cod	tripartite motif containing 31 [Source:HGNC Symbol;Acc:HGNC:16289]
ENSG0000	0	0	0	7.711115	3.638547	8.019476	0	6.456379	-5.046129441	0.002818	0.011996	DSCAML1	11	117427773	117817525	-	7035	protein_cod	DS cell adhesion molecule like 1 [Source:HGNC Symbol;Acc:HGNC:14656]
ENSG0000	2.05233	4.148415	5.628445	19.27779	10.91564	27.62264	3.943063	19.27202	-2.299468754	0.002865	0.012171	MCF2L-AS1	13	112967484	112968824	-	712	antisense	MCF2L antisense RNA 1 [Source:HGNC Symbol;Acc:HGNC:39825]
ENSG0000	0	0	1.125689	9.638893	7.277095	9.801581	0.37523	8.905856	-4.548572789	0.002875	0.012208	NAMA	9	99355337	99377240	-	5342	lincRNA	non-protein coding RNA, associated with MAP kinase pathway and growth arrest [Source:NCBI gene;Acc:100996569]
ENSG0000	16.41864	11.40814	32.64498	70.36392	47.30111	36.53317	20.15725	51.3994	-1.356770803	0.002886	0.012248	AC024941.2	12	88580531	88598218	+	493	antisense	novel transcript, antisense to KITLG
ENSG0000	29.75879	22.81628	24.76516	43.37502	58.21676	65.93791	25.78008	55.84323	-1.115771388	0.002897	0.012291	RIN2	20	19757606	20002459	+	8273	protein_cod	Ras and Rab interactor 2 [Source:HGNC Symbol;Acc:HGNC:18750]
ENSG0000	25.65413	36.29863	32.64498	61.68892	54.57581	76.63055	31.53258	64.29922	-1.029347556	0.002901	0.012305	AC138207.4	17	30971652	30973312	+	1661	sense_intro	novel transcript, sense intronic RNF135
ENSG0000	37.96811	51.85518	41.6505	13.49445	10.91564	26.73159	43.8246	17.04723	1.358705824	0.002908	0.012325	FAM83A	8	123178960	123210079	+	6317	protein_cod	family with sequence similarity 83 member A [Source:HGNC Symbol;Acc:HGNC:28210]
ENSG0000	6.15699	3.111311	2.251378	14.45834	18.19274	23.16737	3.839893	18.60615	-2.268055437	0.002909	0.012329	AL445228.2	1	184385753	184386704	-	952	lincRNA	novel transcript
ENSG0000	5.130825	3.111311	4.502756	7.711115	29.10838	24.94948	4.248298	20.58966	-2.280328936	0.002911	0.012336	KRT18P5	22	20482815	20484105	-	1291	processed_p	keratin 18 pseudogene 5 [Source:HGNC Symbol;Acc:HGNC:19259]
ENSG0000	10.26165	12.44524	10.1312	34.70002	23.65056	37.42422	10.94603	31.92493	-1.543021735	0.002923	0.012374	SLC4A9	5	140360202	140375143	+	3635	protein_cod	solute carrier family 4 member 9 [Source:HGNC Symbol;Acc:HGNC:11035]
ENSG0000	1.026165	3.111311	0	18.3139	5.457821	16.93	1.379159	13.56724	-3.27390327	0.002934	0.012412	SETP5	9	137640986	137641547	-	562	processed_p	SET pseudogene 5 [Source:HGNC Symbol;Acc:HGNC:42924]
ENSG0000	15.39248	2.074207	9.005513	0.963889	0	0	8.824065	0.321296	4.741422607	0.002953	0.012487	IGFL2-AS1	19	46189166	46203083	-	1751	lincRNA	IGFL2 antisense RNA 1 [Source:HGNC Symbol;Acc:HGNC:52559]
ENSG0000	1.026165	4.148415	1.125689	18.3139	17.2831	8.910529	2.10009	14.83584	-2.809975231	0.002962	0.012522	AL449106.1	1	178724306	178726285	-	1980	antisense	novel transcript, antisense to RALGPS2
ENSG0000	20.5233	22.81628	11.25689	33.73613	51.8493	49.89896	18.19882	45.16146	-1.306857462	0.002987	0.012622	ATP6V1G2	6	31544462	31548427	-	2147	protein_cod	ATPase H+ transporting V1 subunit G2 [Source:HGNC Symbol;Acc:HGNC:862]
ENSG0000	40.02044	64.30043	60.78721	22.16945	15.46383	33.86001	55.03603	23.8311	1.20472094	0.003005	0.01269	LINC00460	13	106374477	106384315	+	4232	lincRNA	long intergenic non-protein coding RNA 460 [Source:HGNC Symbol;Acc:HGNC:42809]
ENSG0000	3.078495	11.40814	3.377067	19.27779	32.74693	20.49422	5.954568	24.17298	-2.015497497	0.003028	0.012775	AL136115.1	1	31933020	31933975	+	517	lincRNA	novel transcript
ENSG0000	22.57563	31.11311	34.89636	62.65281	47.30111	76.63055	29.52837	62.19482	-1.077911515	0.003041	0.012825	LINC00638	14	104821201	104823718	+	2518	lincRNA	long intergenic non-protein coding RNA 638 [Source:HGNC Symbol;Acc:HGNC:28325]
ENSG0000	0	0	0	6.747225	0.909637	13.36579	0	7.007552	-5.165741727	0.003047	0.012842	FMO3	1	171090877	171117819	+	3463	protein_cod	flavin containing monooxygenase 3 [Source:HGNC Symbol;Acc:HGNC:3771]
ENSG0000	9.235486	18.66787	3.377067	29.88057	33.65656	36.53317	10.42681	33.35677	-1.668293835	0.003055	0.01287	KCNK15-AS1	20	44694892	44746021	-	1226	antisense	KCNK15 and WISP2 antisense RNA 1 [Source:HGNC Symbol;Acc:HGNC:49901]
ENSG0000	19.49714	20.74207	16.88534	34.70002	34.5662	74.84844	19.04152	48.03822	-1.335458237	0.003063	0.012901	TSHZ2	20	52972407	53495330	+	12581	protein_cod	teashirt zinc finger homeobox 2 [Source:HGNC Symbol;Acc:HGNC:13010]
ENSG0000	23.6018	40.44704	38.27343	81.93059	43.66257	92.6695	34.10742	72.75422	-1.094665627	0.003142	0.013185	LINC01637	22	20957092	20964679	+	684	lincRNA	long intergenic non-protein coding RNA 1637 [Source:HGNC Symbol;Acc:HGNC:52424]
ENSG0000	4.10466	8.29683	5.628445	26.02501	17.2831	24.94948	6.009978	22.75253	-1.919641947	0.003161	0.013262	AC022507.1	12	75600047	75601551	+	1505	processed_p	phosphogluconate dehydrogenase (PGD) pseudogene
ENSG0000	13.34015	24.89049	12.38258	39.51946	46.39148	40.98843	16.87107	42.29979	-1.322778488	0.003172	0.013301	AL022326.1	22	39292988	39295589	+	1804	lincRNA	novel transcript
ENSG0000	17.44481	30.07601	33.77067	37.59168	69.1324	76.63055	27.09716	61.11821	-1.178977044	0.003198	0.013392	AL606834.1	14	50662511	50663178	-	668	sense_intro	novel transcript, sense intronic to SAV1
ENSG0000	27.70646	18.66787	16.88534	76.14726	27.2891	55.24528	21.08655	52.89388	-1.322207558	0.003215	0.013453	RN7SL3	14	49853616	49853914	-	299	misc_RNA	RNA, 7SL, cytoplasmic 3 [Source:HGNC Symbol;Acc:HGNC:23135]
ENSG0000	0	0	0	2.891668	18.19274	0.891053	0	7.325152	-5.229457677	0.003229	0.013504	RN7SL724P	3	139609669	139609939	-	271	misc_RNA	RNA, 7SL, cytoplasmic 724, pseudogene [Source:HGNC Symbol;Acc:HGNC:46740]
ENSG0000	4.10466	19.70497	11.25689	32.77224	40.02042	32.96896	11.68884	35.25507	-1.593576539	0.003238	0.013532	C21orf62-AS	21	32772100	32893735	+	3504	antisense	C21orf62 antisense RNA 1 [Source:HGNC Symbol;Acc:HGNC:1290]
ENSG0000	0	1.037104	0	0.963889	11.82528	16.93	0.345701	9.906391	-4.705747251	0.003239	0.013534	DUSP15	20	31847637	31870747	-	3791	protein_cod	dual specificity phosphatase 15 [Source:HGNC Symbol;Acc:HGNC:16236]
ENSG0000	19.49714	20.74207	22.51378	50.12224	32.74693	64.15581	20.91766	49.00833	-1.229919036	0.003247	0.01356	RN7SKP23	16	23412380	23416680	+	4301	TEC	RNA, 7SK small nuclear pseudogene 23 [Source:HGNC Symbol;Acc:HGNC:45747]

ENSG0000	18.47097	11.40814	10.1312	0.963889	0.909637	3.564211	13.33677	1.812579	2.868569726	0.003254	0.013582	P3H2	3	189956728	190122437	-	5605	protein_cod	prolyl 3-hydroxylase 2 [Source:HGNC Symbol;Acc:HGNC:19317]
ENSG0000	586.9664	488.4758	373.7288	149.4028	277.4392	290.4832	483.057	239.1084	1.014225091	0.003262	0.013607	CEP44	4	174283677	174333380	+	7132	protein_cod	centrosomal protein 44 [Source:HGNC Symbol;Acc:HGNC:29356]
ENSG0000	18.47097	22.81628	16.88534	59.76114	41.84329	36.53317	19.39086	46.05487	-1.24400331	0.003269	0.013633	TEX22	14	105398579	105450106	+	2837	protein_cod	testis expressed 22 [Source:HGNC Symbol;Acc:HGNC:40026]
ENSG0000	4.10466	5.185518	4.502756	16.38612	25.46983	16.93	4.597645	19.59532	-2.09197234	0.003274	0.013652	AC211476.6	7	72954797	72957106	+	2085	lincRNA	novel transcript
ENSG0000	24.62796	50.81808	43.90187	8.675004	24.56019	4.455264	39.78264	12.56349	1.661445436	0.003283	0.013683	CYP1A1	15	74719542	74725610	-	3435	protein_cod	cytochrome P450 family 1 subfamily A member 1 [Source:HGNC Symbol;Acc:HGNC:2595]
ENSG0000	11.28782	9.333933	12.38258	22.16945	27.2891	49.00791	11.00144	32.82216	-1.581145349	0.003294	0.013725	TMEM220	17	10699015	10730316	-	3508	protein_cod	transmembrane protein 220 [Source:HGNC Symbol;Acc:HGNC:33757]
ENSG0000	2.05233	7.259726	14.63396	35.6639	25.46983	24.05843	7.982005	28.39739	-1.844532094	0.003304	0.01376	SCTR	2	119439843	119525301	-	3426	protein_cod	secretin receptor [Source:HGNC Symbol;Acc:HGNC:10608]
ENSG0000	6.15699	3.111311	6.754134	22.16945	17.2831	24.05843	5.340812	21.17033	-1.991626936	0.003309	0.013777	LINC02447	4	7094571	7103385	-	3763	lincRNA	long intergenic non-protein coding RNA 2447 [Source:HGNC Symbol;Acc:HGNC:53379]
ENSG0000	22.57563	9.333933	9.005513	30.84446	48.21075	35.64211	13.63836	38.23244	-1.481689594	0.003314	0.013791	AC023509.4	12	53500162	53500936	-	775	antisense	novel transcript, antisense to TARBP2
ENSG0000	2.05233	5.185518	2.251378	10.60278	17.2831	23.16737	3.163076	17.01775	-2.424764745	0.003317	0.013802	AC093083.1	2	220127546	220212491	+	347	lincRNA	novel transcript
ENSG0000	6.15699	3.111311	5.628445	14.45834	14.55419	35.64211	4.965582	21.55155	-2.121930702	0.003323	0.013819	AL357055.3	1	113011687	113073105	-	2459	lincRNA	uncharacterized LOC100996251 [Source:NCBI gene;Acc:100996251]
ENSG0000	14.36631	8.29683	11.25689	0.963889	0.909637	1.782106	11.30668	1.218544	3.207640506	0.003332	0.013853	AP005136.2	18	2561172	2562220	-	1049	sense_intro	novel transcript, sense intronic METTL4
ENSG0000	0	0	0	2.891668	10.91564	5.346317	0	6.384542	-5.032055076	0.003343	0.013884	AC093330.1	18	76974690	76984162	+	2478	antisense	uncharacterized LOC105372217 [Source:NCBI gene;Acc:105372217]
ENSG0000	26.68029	28.0018	23.63947	48.19447	64.58421	52.57212	26.10719	55.11693	-1.077219034	0.003353	0.013917	ZNF433-AS1	19	11987617	12046275	+	5890	processed_t	ZNF433 and ZNF878 antisense RNA 1 [Source:HGNC Symbol;Acc:HGNC:53776]
ENSG0000	8.209321	2.074207	2.251378	13.49445	23.65056	22.27632	4.178302	19.80711	-2.235403688	0.003362	0.013952	AC073073.1	7	105530209	105530671	+	463	processed_j	ring finger protein 181 (RNF181) pseudogene
ENSG0000	14.36631	11.40814	15.75965	33.73613	35.47584	38.31527	13.8447	35.84241	-1.374985241	0.003387	0.014037	OMG	17	31272013	31297539	-	3837	protein_cod	oligodendrocyte myelin glycoprotein [Source:HGNC Symbol;Acc:HGNC:8135]
ENSG0000	4.10466	4.148415	7.879823	20.24168	25.46983	17.82106	5.377633	21.17752	-1.986969072	0.003392	0.014053	BX649632.1	9	134132242	134135772	-	974	antisense	novel transcript, antisense to WDR5
ENSG0000	33.86345	39.40994	33.77067	42.41113	89.14441	89.10529	35.68135	73.55361	-1.045176912	0.003394	0.014058	AGBL3	7	134986508	135147963	+	7307	protein_cod	ATP/GTP binding protein like 3 [Source:HGNC Symbol;Acc:HGNC:27981]
ENSG0000	17.44481	11.40814	32.64498	47.23058	50.93966	49.00791	20.49931	49.05938	-1.267470207	0.003403	0.014089	AC062017.1	2	239401436	239402364	+	613	antisense	uncharacterized LOC101928111 [Source:NCBI gene;Acc:101928111]
ENSG0000	0	2.074207	1.125689	2.891668	19.10237	16.03895	1.066632	12.67766	-3.578780371	0.003405	0.014094	ALDOB	9	101420578	101449664	-	3935	protein_cod	aldolase, fructose-bisphosphate B [Source:HGNC Symbol;Acc:HGNC:417]
ENSG0000	10.26165	17.63076	9.005513	39.51946	31.83729	31.18685	12.29931	34.1812	-1.46972685	0.003424	0.014169	ERICD	8	140636281	140638283	+	2003	lincRNA	E2F1-regulated inhibitor of cell death [Source:HGNC Symbol;Acc:HGNC:49404]
ENSG0000	6.15699	16.59366	13.50827	21.20557	45.48184	40.09738	12.08631	35.59493	-1.562821626	0.003449	0.014262	AL080317.2	6	111297126	111298510	+	1385	lincRNA	novel transcript
ENSG0000	0	0	1.125689	7.711115	4.548184	14.25685	0.37523	8.838715	-4.539107704	0.00346	0.014298	AC084116.3	8	126518698	126522351	+	429	lincRNA	uncharacterized LOC105375750 [Source:NCBI gene;Acc:105375750]
ENSG0000	21.54947	16.59366	14.63396	54.94169	35.47584	38.31527	17.59236	42.91093	-1.28181754	0.003478	0.014349	SMIM6	17	75646243	75647977	+	1086	protein_cod	small integral membrane protein 6 [Source:HGNC Symbol;Acc:HGNC:40032]
ENSG0000	0	1.037104	0	7.711115	4.548184	14.25685	0.345701	8.838715	-4.539104588	0.003478	0.014349	AL135818.2	14	91258299	91259003	+	705	lincRNA	novel transcript
ENSG0000	57.46524	43.55836	46.15325	20.24168	21.83128	24.94948	49.05895	22.34081	1.134223066	0.003482	0.01436	AC092919.2	3	171460612	171462256	+	1645	TEC	TEC
ENSG0000	0	0	0	2.891668	11.82528	4.455264	0	6.390737	-5.033263834	0.003488	0.014383	AL583844.1	1	235614674	235616397	+	1724	processed_j	solute carrier family 39 (zinc transporter), member 14 pseudogene (SLC39A14)
ENSG0000	11.28782	4.148415	4.502756	15.42223	28.19874	31.18685	6.646329	24.93594	-1.902542626	0.003491	0.014383	LINC00240	6	26956992	27023924	+	6481	lincRNA	long intergenic non-protein coding RNA 240 [Source:HGNC Symbol;Acc:HGNC:18772]
ENSG0000	0	0	0	9.638893	4.548184	4.455264	0	6.214114	-4.989036959	0.003496	0.014401	AL590399.5	9	62387171	62394004	-	3325	lincRNA	novel transcript
ENSG0000	0	1.037104	5.628445	15.42223	9.096368	22.27632	2.22185	15.59831	-2.84809631	0.003532	0.014526	A2M	12	9067664	9116229	-	6384	protein_cod	alpha-2-macroglobulin [Source:HGNC Symbol;Acc:HGNC:7]
ENSG0000	1.026165	5.185518	4.502756	15.42223	26.37947	12.47474	3.57148	18.09215	-2.347984261	0.00354	0.014552	AC090844.2	17	39927742	39939601	+	965	lincRNA	leucine rich repeat containing 3C [Source:NCBI gene;Acc:100505591]
ENSG0000	0	0	0	9.638893	3.638547	5.346317	0	6.207919	-4.987783507	0.003569	0.014664	AC004672.1	12	1380060	1391502	-	2088	antisense	novel transcript
ENSG0000	9.235486	6.222622	6.754134	19.27779	19.10237	39.20633	7.404081	25.86216	-1.80435388	0.003571	0.014667	AC006511.2	12	8017201	8017860	-	660	processed_j	heat shock 60kDa protein 1 (chaperonin) (HSPD1) pseudogene
ENSG0000	3.078495	0	0	9.638893	11.82528	14.25685	1.026165	11.90701	-3.504693964	0.003582	0.014711	AC009407.1	2	231052040	231052637	+	598	sense_overl	novel transcript
ENSG0000	0	0	0	4.819447	5.457821	8.019476	0	6.098914	-4.965508403	0.003597	0.014764	AL021920.3	1	16739938	16750589	-	819	lincRNA	novel transcript
ENSG0000	6.15699	17.63076	4.502756	25.06112	35.6639	20.92165	37.42422	9.43017	31.33659	-1.725034579	0.003611	0.014814	ACTG1P17	15	82725873	82738904	-	2096	transcribed_	actin gamma 1 pseudogene 17 [Source:HGNC Symbol;Acc:HGNC:51497]
ENSG0000	8.209321	5.185518	14.63396	34.70002	22.74092	30.2958	9.342932	29.24558	-1.655062791	0.00366	0.015002	PODXL2	3	127629181	127672809	+	2186	protein_cod	podocalyxin like 2 [Source:HGNC Symbol;Acc:HGNC:17936]
ENSG0000	38.99427	29.0389	36.02205	6.747225	18.19274	12.47474	34.68508	12.47157	1.47091811	0.003661	0.015006	AC139491.2	5	176143085	176185155	-	4206	processed_t	centrosomal protein 192kDa pseudogene [Source:NCBI gene;Acc:643201]
ENSG0000	29.75879	40.44704	27.01654	77.11115	56.39748	61.48265	32.40746	64.99709	-1.000845103	0.003676	0.015056	ACRBP	12	6638075	6647460	-	2843	protein_cod	acrosin binding protein [Source:HGNC Symbol;Acc:HGNC:17195]
ENSG0000	0	1.037104	0	12.53056	2.72891	11.58369	0.345701	8.94772	-4.554474757	0.003684	0.015086	AC020907.1	19	35090930	35113664	+	3099	lincRNA	novel transcript
ENSG0000	2.05233	17.63076	2.251378	25.06112	37.29511	25.84053	7.31149	29.39892	-1.999258997	0.003686	0.015092	PAH	12	102836885	102958410	-	7731	protein_cod	phenylalanine hydroxylase [Source:HGNC Symbol;Acc:HGNC:8582]
ENSG0000	14.36631	10.37104	15.75965	26.02501	30.01801	55.24528	13.499	37.0961	-1.46287884	0.003692	0.015114	AC092384.3	16	88936779	88939651	-	2873	sense_intro	novel transcript
ENSG0000	7.183155	1.037104	2.251378	28.91668	19.10237	8.910529	3.490546	18.97653	-2.430881655	0.003694	0.015115	MARK2P8	3	128748538	128749489	-	952	processed_j	microtubule affinity regulating kinase 2 pseudogene 8 [Source:HGNC Symbol;Acc:HGNC:39799]
ENSG0000	8.209321	3.111311	5.628445	8.675004	23.65056	40.09738	5.649692	24.14098	-2.097316408	0.003694	0.015115	AC087241.4	12	22638464	22638943	+	480	sense_intro	novel transcript, sense intronic to ETNK1
ENSG0000	20.5233	31.11311	18.01103	53.01391	45.48184	55.24528	23.21581	51.24701	-1.139296655	0.003717	0.015197	ADAMTSL4-	1	150560202	150574552	-	2622	processed_t	ADAMTSL4 antisense RNA 1 [Source:HGNC Symbol;Acc:HGNC:32041]
ENSG0000	21.54947	20.74207	38.27343	50.12224	61.8553	59.70054	26.85499	57.22603	-1.098333019	0.003742	0.01529	AL645608.2	1	911435	914948	+	3043	lincRNA	novel transcript
ENSG0000	41.0466	39.40994	25.89085	51.08613	85.50586	76.63055	35.44913	71.07418	-1.001395279	0.003754	0.015332	AL118516.1	22	46761894	46762563	-	670	antisense	novel transcript, antisense to TBC1D22A
ENSG0000	6.15699	6.222622	11.25689	0	0	0.891053	7.878834	0.297018	4.574723156	0.003755	0.015334	LRP2	2	169127109	169362685	-	16260	protein_cod	LDL receptor related protein 2 [Source:HGNC Symbol;Acc:HGNC:6694]
ENSG0000	6.15699	3.111311	7.879823	21.20557	14.55419	31.18685	5.716042	22.31553	-1.972488573	0.003757	0.015335	AC091153.3	17	4704230	4705529	+	472	antisense	uncharacterized LOC101559451 [Source:NCBI gene;Acc:101559451]
ENSG0000	0	0	1.125689	6.747225	6.367458	12.47474	0.37523	8.529808	-4.487955253	0.003757	0.015335	AC006270.1	17	63117019	63130947	-	478	antisense	novel transcript, antisense to TANC2
ENSG0000	0	0	0	7.711115	8.186731	2.673159	0	6.190335	-4.984275848	0.003764	0.01536	RNA5SP78	1	229549905	229550022	+	118	rRNA_pseuc	RNA, 5S ribosomal pseudogene 78 [Source:HGNC Symbol;Acc:HGNC:42855]
ENSG0000	7.183155	4.148415	5.628445	0	0	0	5.653339	0	5.060707364	0.003766	0.015367	AP003072.5	11	93171134	93171906	+	773	TEC	novel transcript
ENSG0000	5.130825	6.222622	9.005513	15.42223	27.2891	29.40474	6.78632	24.03869	-1.833106414	0.00378	0.015409	AL121917.1	20	58863528	58888809	-	1521	antisense	uncharacterized LOC101927932 [Source:NCBI gene;Acc:101927932]
ENSG0000	0	0	0	3.855557	9.096368	5.346317	0	6.099414	-4.965625808	0.003793	0.015449	AL035693.1	6	2227803	2230261	+	808	antisense	novel transcript, antisense to GMDS
ENSG0000	21.54947	8.29683	11.25689	44.33891	28.19874	40.09738	13.70106	37.54501	-1.449915751	0.003793	0.015449	GRIN3B	19	1000419	1009732	+	3281	protein_cod	glutamate ionotropic receptor NMDA type subunit 3B [Source:HGNC Symbol;Acc:HGNC:16768]
ENSG0000	2.05233	3.111311	0	12.53056	9.096368	18.71211	1.721214	13.44635	-2.942646517	0.003798	0.015464	NANOGP5	9	100175178	100176054	+	877	transcribed_	Nanog homeobox pseudogene 5 [Source:HGNC Symbol;Acc:HGNC:23103]
ENSG0000	20.5233	15.55656	12.38258	31.80835	58.21676	33.86001	16.15415	41.29504	-1.35094986	0.003799	0.015469	SNHG21	15	82750564	82757206	+	1053	antisense	small nucleolar RNA host gene 21 [Source:HGNC Symbol;Acc:HGNC:50284]
ENSG0000	1.026165	2.074207	0	12.53056	8.186731	13.36579	1.033457	11.36103	-3.43189621	0.003804	0.015487	AC015802.3	17	76549951	76550826	+	570	sense_intro	novel transcript, sense intronic to PRCD
ENSG0000	2.05233	2.074207	4.502756	16.38612	6.367458	27.62264	2.876431	16.79207	-2.558655575	0.003814	0.015513	SCT	11	626431	627143	-	366	protein_cod	secretin [Source:HGNC Symbol;Acc:HGNC:10607]
ENSG0000	12.31398	29.0389	29.26792	44.33891	63.67458	52.57212	23.54027	53.52853	-1.18993392	0.003831	0.015577	LINC01970	17	82290046	82292814	-	1810	lincRNA	long intergenic non-protein coding RNA 1970 [Source:HGNC Symbol;Acc:HGNC:52796]
ENSG0000	7.183155	7.259726	4.502756	14.45834	22.74092	30.2958	5.969511	22.49835	-1.91140524	0.003837	0.015597	AC009802.1	18	63966406	63970181	-	1609	antisense	uncharacterized LOC105372165 [Source:NCBI gene;Acc:105372165]
ENSG0000	3.078495	6.222622	10.1312	15.42223	21.83128	35.64211	6.47744	24.29854	-1.920403549	0.003868	0.015683	AC024575.1	19	11368123	11374935	-	682	antisense	novel transcript, antisense to SWSAP1
ENSG0000	5.130825	16.59366	16.88534	33.73613	47.30111	28.51369	12.86994	36.51698	-1.510223243	0.003868	0.015683	AC123768.3	15	32586105	32615158	-	1232	antisense	novel transcript, antisense to GOLGA8N
ENSG0000	56.43908	38.37284	63.03859	29.88057	20.01201	22.27632	52.61683	24.0563	1.13002194	0.003876	0.015713	KBTBD8	3	66998307	67011210	+	4736	protein_cod	kelch repeat and BTB domain containing 8 [Source:HGNC Symbol;Acc:HGNC:30691]
ENSG0000	0	0	0	4.819447	10.006	3.564211	0	6.129888	-4.971900239	0.003876	0.015713	EPB42	15	43197227	43221125	-	3039	protein_cod	erythrocyte membrane protein band 4.2 [Source:HGNC Symbol;Acc:HGNC:3381]
ENSG0000	2.05233	6.222622	2.251378	25.06112	12.73492	15.1479	3.508777	17.64798	-2.32233713	0.003904	0.015818	AL445471.1	1	24961345	24963097	+	820	antisense	novel transcript
ENSG0000	13.34015	20.74207	1.125689	28.91668	44.5722	40.98843	11.73597	38.15911	-1.69027763	0.003911	0.015836	ZNF660	3	44578223	44599694	+	6513	protein_cod	zinc finger protein 660 [Source:HGNC Symbol;Acc:HGNC:26720]
ENSG0000	11.28782	20.74207	23.63947	4.819447	4.548184	3.564211	18.55645	4.310614	2.105856733	0.003918	0.015859	TNFRSF9	1	7915894	7943165	-	6331	protein_cod	TNF receptor superfamily member 9 [Source:HGNC Symbol;Acc:HGNC:11924]
ENSG0000	19.49714	7.259726	21.38809	36.62779	38.20475	49.00791	16.04832	41.28015	-1.368186556	0.003922	0.015867	AC015912.3	17	56914186	56914533	+	348	lincRNA	novel transcript

ENSG0000	6.15699	14.51945	14.63396	32.77224	33.65656	32.0779	11.77013	32.83557	-1.485000395	0.003968	0.016031	PDCD4-AS1	10	110869868	110872233	-	778	antisense	PDCD4 antisense RNA 1 [Source:HGNC Symbol;Acc:HGNC:27425]
ENSG0000	1.026165	9.333933	4.502756	26.02501	14.55419	24.05843	4.954285	21.54588	-2.120194704	0.003983	0.016083	AC245297.3	1	149176022	149251013	+	1331	lincRNA	novel transcript
ENSG0000	1.026165	14.51945	1.125689	26.02501	74.59022	67.72002	5.557102	56.11175	-3.331208501	0.003983	0.016083	AADACL2-AS	3	151751443	151928175	-	635	antisense	AADACL2 antisense RNA 1 [Source:HGNC Symbol;Acc:HGNC:50301]
ENSG0000	48.22976	54.9665	43.90187	21.20557	20.01201	26.73159	49.03271	22.64972	1.113916482	0.004014	0.016193	ATP2A1-AS1	16	28878957	28879920	-	583	antisense	ATP2A1 antisense RNA 1 [Source:HGNC Symbol;Acc:HGNC:51370]
ENSG0000	16.41864	22.81628	31.51929	5.783336	9.096368	5.346317	23.58474	6.742007	1.80296452	0.004026	0.016232	TNFRSF1B	1	12167003	12209228	+	3766	protein_cod	TNF receptor superfamily member 1B [Source:HGNC Symbol;Acc:HGNC:11917]
ENSG0000	0	3.111311	1.125689	9.638893	10.006	17.82106	1.412333	12.48865	-3.144185347	0.004046	0.016309	RN7SL589P	4	2316197	2316506	-	310	misc_RNA	RNA, 7SL, cytoplasmic 589, pseudogene [Source:HGNC Symbol;Acc:HGNC:46605]
ENSG0000	13.34015	3.111311	1.125689	26.9889	17.2831	30.2958	5.859049	24.85593	-2.069403674	0.004047	0.01631	AC068987.1	12	51848223	51852729	+	427	lincRNA	uncharacterized LOC105369971 [Source:NCBI gene;Acc:105369971]
ENSG0000	38.99427	31.11311	48.40463	13.49445	20.92165	13.36579	39.504	15.9273	1.307731999	0.004048	0.016311	CKS1BP3	5	62512246	62512482	-	237	processed_p	CDC28 protein kinase regulatory subunit 1B pseudogene 3 [Source:HGNC Symbol;Acc:HGNC:24233]
ENSG0000	2.05233	3.111311	1.125689	7.711115	18.19274	16.03895	2.096443	13.98093	-2.729149592	0.004049	0.016312	AC096564.2	4	107936031	107941255	-	3147	antisense	novel transcript
ENSG0000	3.078495	6.222622	9.005513	12.53056	19.10237	40.98843	6.10221	24.20712	-1.999733756	0.004089	0.016454	BIRC6-AS2	2	32557273	32574818	-	1066	transcribed_	BIRC6 antisense RNA 2 [Source:HGNC Symbol;Acc:HGNC:50490]
ENSG0000	0	1.037104	0	5.783336	5.457821	14.25685	0.345701	8.499334	-4.48342414	0.004154	0.016694	NAALADL2-A	3	175079307	175115242	-	422	processed_t	NAALADL2 antisense RNA 3 [Source:HGNC Symbol;Acc:HGNC:41014]
ENSG0000	20.5233	18.66787	15.75965	36.62779	38.20475	54.35422	18.31694	43.06225	-1.232161476	0.004162	0.016716	LVRN	5	115962454	116027619	+	6636	protein_cod	laeverin [Source:HGNC Symbol;Acc:HGNC:26904]
ENSG0000	9.235486	10.37104	12.38258	29.88057	34.5662	25.84053	10.66303	30.09577	-1.499756893	0.004162	0.016716	WWC2-AS2	4	183097017	183099199	-	2183	lincRNA	WWC2 antisense RNA 2 [Source:HGNC Symbol;Acc:HGNC:26390]
ENSG0000	0	0	1.125689	7.711115	14.55419	3.564211	0.37523	8.609838	-4.49963672	0.004181	0.016777	PPEF1	X	18675909	18827921	+	3513	protein_cod	protein phosphatase with EF-hand domain 1 [Source:HGNC Symbol;Acc:HGNC:9243]
ENSG0000	1.026165	0	0	11.56667	5.457821	8.019476	0.342055	8.347989	-4.453878588	0.004185	0.016784	AC234781.1	X	155334858	155351957	-	1721	antisense	novel transcript
ENSG0000	27.70646	21.77918	21.38809	53.9778	50.03002	47.2258	23.62458	50.41121	-1.091179353	0.004219	0.016908	EDDM13	19	56272769	56310450	+	2326	protein_cod	epididymal protein 13 [Source:HGNC Symbol;Acc:HGNC:53168]
ENSG0000	5.130825	8.29683	1.125689	19.27779	23.65056	18.71211	4.851115	20.54682	-2.068398657	0.004225	0.016928	ADA2	22	17178790	17258235	-	8704	protein_cod	adenosine deaminase 2 [Source:HGNC Symbol;Acc:HGNC:1839]
ENSG0000	3.078495	1.037104	2.251378	8.675004	10.006	24.05843	2.122326	14.24648	-2.749488712	0.004232	0.016953	MT1P1	9	95413267	95413449	-	183	processed_p	metallothionein 1 pseudogene 1 [Source:HGNC Symbol;Acc:HGNC:23681]
ENSG0000	0	5.185518	4.502756	11.56667	18.19274	22.27632	3.229425	17.34524	-2.43721417	0.004254	0.017027	PCDHGA12	5	141430589	141512979	+	4815	protein_cod	protocadherin gamma subfamily A, 12 [Source:HGNC Symbol;Acc:HGNC:8699]
ENSG0000	4.10466	3.111311	0	21.20557	13.64455	10.69263	2.405324	15.18092	-2.632784254	0.004296	0.017168	KRTAP5-AS1	11	1571353	1599184	+	2931	antisense	KRTAP5-1/KRTAP5-2 antisense RNA 1 [Source:HGNC Symbol;Acc:HGNC:27877]
ENSG0000	2.05233	1.037104	1.125689	11.56667	21.83128	4.455264	1.405041	12.61774	-3.159988564	0.004306	0.017196	AC116667.1	16	73060245	73062316	+	2072	lincRNA	novel transcript
ENSG0000	9.235486	16.59366	3.377067	36.62779	19.10237	39.20633	9.735404	31.6455	-1.690956376	0.00433	0.017279	AL162411.1	9	6645956	6670635	+	526	lincRNA	novel transcript
ENSG0000	13.34015	28.0018	12.38258	2.891668	4.548184	4.455264	17.90818	3.965039	2.173149427	0.004331	0.01728	AL139241.1	10	98252023	98256575	+	1168	antisense	novel transcript
ENSG0000	24.62796	37.33573	29.26792	76.14726	57.30712	51.68107	30.41054	61.71181	-1.019304616	0.004334	0.017285	AC005921.3	17	50722074	50724224	+	2151	TEC	TEC
ENSG0000	17.44481	3.111311	9.005513	49.15836	24.56019	24.05843	9.853877	32.59233	-1.721021179	0.004342	0.01731	ANKRD23	2	96824526	96857934	-	4116	protein_cod	ankyrin repeat domain 23 [Source:HGNC Symbol;Acc:HGNC:24470]
ENSG0000	29.75879	41.48415	24.76516	73.25559	70.95167	49.89896	32.0027	64.70207	-1.011726852	0.004343	0.01731	SLC1A2	11	35251205	35420063	-	22835	protein_cod	solute carrier family 1 member 2 [Source:HGNC Symbol;Acc:HGNC:10940]
ENSG0000	16.41864	19.70497	19.13671	49.15836	40.93366	37.42422	18.42011	42.50541	-1.206095311	0.004345	0.017312	RTN1	14	59595976	59870966	-	6209	protein_cod	reticulon 1 [Source:HGNC Symbol;Acc:HGNC:10467]
ENSG0000	15.39248	16.59366	16.88534	26.9889	55.48785	39.20633	16.29049	40.56102	-1.318260497	0.004345	0.017312	CA3	8	85373436	85449040	+	2310	protein_cod	carbonic anhydrase 3 [Source:HGNC Symbol;Acc:HGNC:1374]
ENSG0000	29.75879	34.22442	28.14223	9.638893	6.367458	16.03895	30.70848	10.68177	1.521238204	0.004364	0.017376	P4HA3	11	74235801	74311657	-	4009	protein_cod	prolyl 4-hydroxylase subunit alpha 3 [Source:HGNC Symbol;Acc:HGNC:30135]
ENSG0000	2.05233	11.40814	5.628445	23.13334	25.46983	22.27632	6.362972	23.6265	-1.892148908	0.004375	0.017406	AC124069.1	8	17703988	17706187	+	735	antisense	novel transcript
ENSG0000	13.34015	8.29683	12.38258	34.70002	20.01201	42.77054	11.33985	32.49419	-1.52005599	0.004376	0.017407	ASGR1	17	7173431	7179564	-	3748	protein_cod	asialoglycoprotein receptor 1 [Source:HGNC Symbol;Acc:HGNC:742]
ENSG0000	5.130825	3.111311	5.628445	15.42223	20.01201	21.38527	4.623527	18.93984	-2.03949979	0.0044	0.017489	C1QTNF8	16	1090005	1096244	-	1762	protein_cod	C1q and TNF related 8 [Source:HGNC Symbol;Acc:HGNC:31374]
ENSG0000	0	0	0	5.783336	5.457821	6.23737	0	5.826176	-4.898464581	0.004402	0.017496	EMCN	4	100395341	100880126	-	5243	protein_cod	endomucin [Source:HGNC Symbol;Acc:HGNC:16041]
ENSG0000	5.130825	2.074207	5.628445	12.53056	13.64455	32.0779	4.277826	19.41767	-2.190214558	0.00441	0.01752	SNORD117	6	31536374	31536449	-	76	snoRNA	small nucleolar RNA, C/D box 117 [Source:HGNC Symbol;Acc:HGNC:32742]
ENSG0000	1.026165	2.074207	0	13.49445	5.457821	15.1479	1.033457	11.36672	-3.433088199	0.004414	0.017527	AC026401.1	16	15608474	15610563	+	530	antisense	novel transcript
ENSG0000	5.130825	7.259726	5.628445	28.91668	16.37346	20.49422	6.006332	21.92812	-1.865743419	0.004443	0.017622	C9orf131	9	35041095	35045991	+	3835	protein_cod	chromosome 9 open reading frame 131 [Source:HGNC Symbol;Acc:HGNC:31418]
ENSG0000	11.28782	26.9647	28.14223	62.65281	51.8493	39.20633	22.13158	51.23614	-1.214087621	0.00446	0.017682	AC087645.2	17	78315729	78347798	+	1145	lincRNA	novel transcript
ENSG0000	14.36631	9.333933	4.502756	32.77224	25.46983	28.51369	9.401	28.91859	-1.610879525	0.004479	0.017747	F10	13	113122814	113149529	+	2356	protein_cod	coagulation factor X [Source:HGNC Symbol;Acc:HGNC:3528]
ENSG0000	51.30825	19.70497	32.64498	5.783336	10.91564	17.82106	34.55274	11.50668	1.580837528	0.004526	0.017923	AC125611.4	12	49292631	49324576	-	4166	antisense	uncharacterized LOC101927267 [Source:NCBI gene;Acc:101927267]
ENSG0000	62.59607	78.81988	58.53583	33.73613	20.01201	44.55264	66.65059	32.76693	1.024441651	0.00453	0.017933	PTMAP5	13	81689911	81691072	+	1162	transcribed_	prothymosin alpha pseudogene 5 [Source:HGNC Symbol;Acc:HGNC:9628]
ENSG0000	3.078495	0	0	14.45834	6.367458	14.25685	1.026165	11.69421	-3.477891428	0.004533	0.017934	PHBP13	13	35251070	35251829	+	760	processed_p	prohibitin pseudogene 13 [Source:HGNC Symbol;Acc:HGNC:39292]
ENSG0000	14.36631	20.74207	12.38258	49.15836	45.48184	25.84053	15.83032	40.16024	-1.338457305	0.004537	0.017949	AC026464.5	16	69443240	69444290	-	722	processed_p	transmembrane protein 111 (TMEM111) pseudogene
ENSG0000	4.10466	12.44524	11.25689	45.3028	22.74092	21.38527	9.268932	29.80966	-1.687155662	0.004563	0.018041	CNTN2	1	205042937	205078284	+	17785	protein_cod	contactin 2 [Source:HGNC Symbol;Acc:HGNC:2172]
ENSG0000	0	0	1.125689	12.53056	3.638547	8.910529	0.37523	8.359879	-4.45571279	0.004567	0.018056	AL354707.3	9	6675284	6675614	+	331	processed_p	60S ribosomal protein L35a (RPL35A) pseudogene
ENSG0000	16.41864	20.74207	22.51378	41.44724	52.75894	40.09738	19.8915	44.76785	-1.172853224	0.004575	0.018077	AC091271.1	17	59618553	59619714	-	1162	lincRNA	novel transcript
ENSG0000	28.73262	19.70497	16.88534	6.747225	4.548184	7.128423	21.77431	6.141277	1.828573423	0.004583	0.018103	AC104971.1	18	58670009	58671877	-	1209	bidirectiona	uncharacterized LOC101927322 [Source:NCBI gene;Acc:101927322]
ENSG0000	8.209321	4.148415	6.754134	26.9889	18.19274	22.27632	6.370623	22.48599	-1.818936547	0.004609	0.018193	AC105020.1	15	75678548	75680752	+	2205	antisense	novel transcript, antisense to CSPG4
ENSG0000	10.26165	2.074207	2.251378	15.42223	16.37346	32.96896	4.862412	21.58822	-2.140608991	0.004613	0.0182	AP003733.4	11	61967794	61969490	+	1697	TEC	uncharacterized LOC399900 [Source:NCBI gene;Acc:399900]
ENSG0000	0	0	0	4.819447	8.186731	4.455264	0	5.820481	-4.89726435	0.004644	0.018318	FOXO1B	5	181099140	181100666	-	1527	processed_p	forkhead box O1B pseudogene [Source:HGNC Symbol;Acc:HGNC:3820]
ENSG0000	0	0	0	5.783336	8.186731	3.564211	0	5.84476	-4.902496021	0.004657	0.018363	AC105416.1	4	138481255	138482096	-	842	processed_p	armadillo repeat containing 1 (ARMC1) pseudogene
ENSG0000	15.39248	17.63076	19.13671	51.08613	25.46983	50.79001	17.38665	42.44866	-1.288751287	0.004704	0.01852	AL034417.2	1	8026738	8122702	+	2044	lincRNA	novel transcript
ENSG0000	0	0	0	3.855557	7.277095	6.23737	0	5.790007	-4.890667326	0.004732	0.01862	NCF1	7	74773962	74789315	+	3462	protein_cod	neutrophil cytosolic factor 1 [Source:HGNC Symbol;Acc:HGNC:7660]
ENSG0000	0	0	0	3.855557	6.367458	7.128423	0	5.783813	-4.889315011	0.004749	0.018681	AC112219.2	3	72178279	72178810	+	532	lincRNA	novel transcript
ENSG0000	1.026165	8.29683	7.879823	30.84446	14.55419	23.16737	5.734273	22.85534	-2.001614118	0.004804	0.018885	ADORA2A-A	22	24429206	24495074	-	4025	antisense	ADORA2A antisense RNA 1 [Source:HGNC Symbol;Acc:HGNC:37122]
ENSG0000	7.183155	2.074207	6.754134	17.35001	30.01801	16.03895	5.337166	21.13566	-1.98993671	0.004814	0.018913	AL049795.1	1	32204769	32206814	-	1357	antisense	novel transcript
ENSG0000	10.26165	5.185518	12.38258	20.24168	28.19874	36.53317	9.276583	28.32453	-1.617424722	0.004853	0.019048	AOC4P	17	42865922	42874369	+	3169	transcribed_	amine oxidase, copper containing 4, pseudogene [Source:HGNC Symbol;Acc:HGNC:48869]
ENSG0000	31.81112	11.40814	16.88534	56.86947	40.02402	46.33475	20.03486	47.74275	-1.248828367	0.00488	0.019136	AL451042.2	1	16155211	16157329	+	904	antisense	novel transcript
ENSG0000	2.05233	4.148415	0	13.49445	9.096368	19.60316	2.066915	14.06466	-2.745438703	0.004882	0.019139	YWHAZP4	6	127355756	127356789	+	708	processed_p	tyrosine 3-monooxygenase/tryptophan 5-monooxygenase activation protein zeta pseudogene 4 [Source:HGNC Symbol;Acc:HGNC:30851]
ENSG0000	4.10466	3.111311	1.125689	10.60278	14.55419	20.49422	2.780553	15.21706	-2.440244571	0.004908	0.019231	MBOAT4	8	30131824	30144686	-	1512	protein_cod	membrane bound O-acyltransferase domain containing 4 [Source:HGNC Symbol;Acc:HGNC:32311]
ENSG0000	0	3.111311	0	5.783336	10.006	19.60316	1.037104	11.7975	-3.488692637	0.004908	0.019231	AP003108.1	11	61496440	61500076	+	554	lincRNA	novel transcript
ENSG0000	1.026165	3.111311	1.125689	11.56667	16.37346	9.801581	1.754388	12.58057	-2.834973756	0.004911	0.019238	SPON1-AS1	11	14262846	14273691	-	240	antisense	SPON1 antisense RNA 1 [Source:HGNC Symbol;Acc:HGNC:53117]
ENSG0000	33.86345	22.81628	41.6505	13.49445	14.55419	8.019476	32.77674	12.02271	1.447285357	0.004919	0.019258	HNRNPLP2	15	34489002	34490571	-	1570	unprocessed	heterogeneous nuclear ribonucleoprotein L pseudogene 2 [Source:HGNC Symbol;Acc:HGNC:48747]
ENSG0000	3.078495	2.074207	12.38258	19.27779	18.19274	32.0779	5.845094	23.18281	-2.008548544	0.004919	0.019258	PRICKLE4	6	41780762	41787372	+	2718	protein_cod	prickle planar cell polarity protein 4 [Source:HGNC Symbol;Acc:HGNC:16805]
ENSG0000	8.209321	18.66787	11.25689	25.06112	38.20475	40.09738	12.71136	34.45442	-1.439064798	0.004922	0.019269	CAMTA1-DT	1	6783892	6784843	-	415	antisense	CAMTA1 divergent transcript [Source:HGNC Symbol;Acc:HGNC:53020]
ENSG0000	2.05233	3.111311	3.377067	26.9889	7.277095	13.36579	2.846903	15.87726	-2.480989396	0.004934	0.019307	AC073052.1	2	224499387	224500100	-	714	processed_p	ankyrin repeat domain 49 (ANKRD49) pseudogene
ENSG0000	7.183155	3.111311	6.754134	22.16945	20.92165	19.60316	5.682867	20.89809	-1.881167977	0.004944	0.01934	AC087239.1	12	25385670	25386241	-	572	lincRNA	novel transcript
ENSG0000	10.26165	5.185518	4.502756	22.16945	21.83128	24.94948	6.649975	22.98341	-1.782452142	0.004946	0.019346	PNCK	X	153669730	153689010	-	5091	protein_cod	pregnancy up-regulated nonubiquitous CaM kinase [Source:HGNC Symbol;Acc:HGNC:13415]
ENSG0000	0	0	0	10.60278	2.72891	4.455264	0	5.928986	-4.920597373	0.00495	0.019357	MIR5091	4	13627865	13627957	+	93	miRNA	microRNA 5091 [Source:HGNC Symbol;Acc:HGNC:43478]
ENSG0000	7.183155	2.074207	4.502756	21.20557	15.46383	20.49422	4.586706	19.05454	-2.052450346	0.004959	0.01939	AC008687.3	19	49064259	49064856	+	598	lincRNA	novel transcript

ENSG0000	3.078495	3.111311	2.251378	12.53056	11.82528	20.49422	2.813728	14.95002	-2.406378613	0.004963	0.019396	AL022157.1	X		57121662	57127243	+	665	antisense	novel transcript
ENSG0000	25.65413	25.92759	23.63947	8.675004	9.096368	7.128423	25.07373	8.299932	1.596617944	0.004996	0.019512	FAM57B		16	30024427	30052978	-	4920	protein_cod	family with sequence similarity 57 member B [Source:HGNC Symbol;Acc:HGNC:25295]
ENSG0000	1.026165	4.148415	2.251378	11.56667	12.73492	18.71211	2.475319	14.3379	-2.535163739	0.005018	0.019576	AC092053.2		3	39152906	39154723	+	1818	antisense	novel transcript, antisense CSRNP1
ENSG0000	8.209321	1.037104	11.25689	17.35001	37.29511	22.27632	6.834438	25.64048	-1.918258564	0.005023	0.019591	AC018628.2		17	62035944	62040352	+	1426	antisense	novel transcript, antisense to MED13
ENSG0000	1.026165	4.148415	2.251378	7.711115	13.64455	23.16737	2.475319	14.84101	-2.586334309	0.005052	0.019692	AC068790.4		12	123962555	123962817	-	263	sense_intro	novel transcript, sense intronic to CCDC92
ENSG0000	11.28782	11.40814	11.25689	23.13334	30.01801	40.09738	11.31762	31.08291	-1.459289738	0.005054	0.019696	METTL15P1		3	156713884	156714928	-	1045	processed_	methyltransferase like 15 pseudogene 1 [Source:HGNC Symbol;Acc:HGNC:31926]
ENSG0000	2.05233	0	0	13.49445	10.006	7.128423	0.68411	10.20963	-3.862408686	0.005104	0.019879	AL139805.1		6	131825981	131828413	-	2433	processed_	T-cell activation leucine repeat-rich protein (TA-LRRP) pseudogene
ENSG0000	4.10466	5.185518	2.251378	15.42223	24.56019	12.47474	3.847186	17.48572	-2.17602718	0.005117	0.019929	LINC02458		12	89010681	89019679	-	2941	lincRNA	long intergenic non-protein coding RNA 2458 [Source:HGNC Symbol;Acc:HGNC:53394]
ENSG0000	26.68029	29.0389	23.63947	45.3028	66.40349	51.68107	26.45289	54.46245	-1.041116084	0.005118	0.01993	NKX2-1		14	36516392	36521149	-	3165	protein_cod	NK2 homeobox 1 [Source:HGNC Symbol;Acc:HGNC:11825]
ENSG0000	0	0	2.251378	10.60278	9.096368	10.69263	0.750459	10.1306	-3.816482886	0.005162	0.020067	AL096701.1		22	31559483	31559939	+	457	processed_	ribosomal protein S18 (RPS18) pseudogene
ENSG0000	3.078495	2.074207	1.125689	8.675004	19.10237	12.47474	2.092797	13.41737	-2.670249451	0.005173	0.020102	AL359955.1		9	64817870	64836341	+	3275	unprocessed	zinc finger pseudogene
ENSG0000	11.28782	15.55656	24.76516	51.08613	32.74693	40.98843	17.20318	41.60716	-1.280009752	0.005181	0.020129	ACTBP7		15	43989061	43990184	-	1124	processed_	actin, beta pseudogene 7 [Source:HGNC Symbol;Acc:HGNC:140]
ENSG0000	6.15699	5.185518	4.502756	0	0	0	5.281755	0	4.963381831	0.005187	0.02014	TUBAL3		10	5393098	5404830	-	1798	protein_cod	tubulin alpha like 3 [Source:HGNC Symbol;Acc:HGNC:23534]
ENSG0000	0	0	0	8.675004	7.277095	1.782106	0	5.911401	-4.916915728	0.005223	0.020264	FAM180A		7	135728348	135748846	-	2526	protein_cod	family with sequence similarity 180 member A [Source:HGNC Symbol;Acc:HGNC:33773]
ENSG0000	70.80539	45.63256	58.53583	13.49445	26.37947	39.20633	58.32459	26.36008	1.140901452	0.005227	0.020267	AC141557.2		12	9415641	9416718	+	482	unprocessed	WAS protein family homolog 1 (WASH1) pseudogene
ENSG0000	30.78495	20.74207	67.54134	21.20557	9.096368	11.58369	39.68946	13.96187	1.507834229	0.005267	0.020408	TSSC2		11	3380961	3408978	+	3666	transcribed_	tumor suppressing subtransferable candidate 2 pseudogene [Source:HGNC Symbol;Acc:HGNC:12384]
ENSG0000	19.49714	24.89049	14.63396	44.33891	41.84329	46.33475	19.67386	44.17232	-1.163105452	0.005271	0.020422	POU2F2		19	42086110	42196585	-	9072	protein_cod	POU class 2 homeobox 2 [Source:HGNC Symbol;Acc:HGNC:9213]
ENSG0000	0	0	1.125689	1.927779	15.46383	8.019476	0.37523	8.47036	-4.478956078	0.005293	0.020501	AC073316.1		7	3140234	3174654	-	1725	lincRNA	uncharacterized LOC100129603 [Source:NCBI gene;Acc:100129603]
ENSG0000	0	0	1.125689	2.891668	12.73492	8.910529	0.37523	8.179037	-4.428394176	0.005296	0.020508	AC015802.5		17	76551352	76551750	+	399	sense_intro	novel transcript, sense intronic to PRCD
ENSG0000	35.91578	48.74387	54.03308	19.27779	20.01201	24.05843	46.23091	21.11607	1.127543342	0.005301	0.020525	RIMKLBP2		1	219199914	219200567	-	654	processed_	ribosomal modification protein rimK like family member B pseudogene 2 [Source:HGNC Symbol;Acc:HGNC:39163]
ENSG0000	4.10466	3.111311	1.125689	14.45834	9.096368	22.27632	2.780553	15.27701	-2.445192572	0.005339	0.020638	AC072061.1		7	39947522	39949755	-	2234	antisense	novel transcript, antisense to CDC2L5
ENSG0000	17.44481	20.74207	16.88534	34.70002	42.75293	48.11685	18.35741	41.8566	-1.189178172	0.005347	0.020659	PDE1B		12	54549350	54579239	+	7618	protein_cod	phosphodiesterase 1B [Source:HGNC Symbol;Acc:HGNC:8775]
ENSG0000	1.026165	1.037104	1.125689	10.60278	12.73492	8.019476	1.062986	10.45239	-3.298905705	0.00535	0.020666	CDC20P1		9	87011652	87013151	+	1500	processed_	cell division cycle 20 pseudogene 1 [Source:HGNC Symbol;Acc:HGNC:29487]
ENSG0000	1.026165	1.037104	1.125689	8.675004	11.82528	10.69263	1.062986	10.39764	-3.29254911	0.005382	0.02077	AL031590.1		22	39242188	39279897	+	4521	antisense	novel transcript, antisense to PDGFB
ENSG0000	11.28782	11.40814	10.1312	23.13334	39.11438	28.51369	10.94239	30.25381	-1.467010872	0.005402	0.020847	AC133552.2		16	25031744	25058109	+	1126	transcribed_	leucine carboxyl methyltransferase 1 pseudogene [Source:NCBI gene;Acc:554206]
ENSG0000	26.68029	20.74207	29.26792	59.76114	51.8493	46.33475	25.56343	52.6484	-1.043357084	0.005436	0.020961	AC092910.3		3	120094895	120136783	+	2194	lincRNA	novel transcript
ENSG0000	4.10466	0	0	15.42223	10.006	11.58369	1.36822	12.33731	-3.142171728	0.005447	0.021	LINC00677		14	103120847	103123007	-	584	lincRNA	long intergenic non-protein coding RNA 677 [Source:HGNC Symbol;Acc:HGNC:20121]
ENSG0000	17.44481	28.0018	33.77067	49.15836	79.1384	42.77054	26.40576	57.02243	-1.114922875	0.005497	0.021169	SUGT1P4-ST		9	97238449	97297314	+	3701	processed_t	SUGT1P4-STRA6LP readthrough [Source:HGNC Symbol;Acc:HGNC:53834]
ENSG0000	6.15699	8.29683	4.502756	18.3139	20.01201	27.62264	6.318859	21.98285	-1.794758946	0.005506	0.021198	AP002490.1		11	72014291	72020910	+	4233	antisense	uncharacterized LOC100128494 [Source:NCBI gene;Acc:100128494]
ENSG0000	26.68029	16.59366	30.3936	58.79725	53.66857	43.66159	24.55585	52.04247	-1.085837556	0.005512	0.021213	LINC02163		5	104079911	104105403	+	574	lincRNA	long intergenic non-protein coding RNA 2163 [Source:HGNC Symbol;Acc:HGNC:53024]
ENSG0000	0	5.185518	0	13.49445	13.64455	13.36579	1.728506	13.5016	-2.946464061	0.005514	0.021215	UBE2CP2		18	22900486	22900995	-	510	processed_	ubiquitin conjugating enzyme E2 C pseudogene 2 [Source:HGNC Symbol;Acc:HGNC:43551]
ENSG0000	18.47097	31.11311	7.879823	61.68892	41.84329	39.20633	19.15464	47.57951	-1.304065188	0.005532	0.021277	PIANP		12	6693792	6700800	-	3266	protein_cod	PILR alpha associated neural protein [Source:HGNC Symbol;Acc:HGNC:25338]
ENSG0000	0	6.222622	1.125689	21.20557	15.46383	9.801581	2.449437	15.49032	-2.650833195	0.005537	0.021292	AC084262.1		8	29352420	29353170	-	751	sense_overl	novel transcript, overlapping to DUSP4
ENSG0000	45.15126	40.44704	48.40463	23.13334	20.92165	17.82106	44.66765	20.62535	1.115746968	0.00555	0.021322	HEY1		8	79764010	79767863	-	3232	protein_cod	hes related family bHLH transcription factor with YRPW motif 1 [Source:HGNC Symbol;Acc:HGNC:4880]
ENSG0000	5.130825	7.259726	3.377067	0	0	0	5.255873	0	4.957344072	0.005553	0.021325	AC053503.4		2	219425071	219426184	-	224	antisense	novel transcript
ENSG0000	7.183155	3.111311	10.1312	35.6639	22.74092	14.25685	6.808556	24.22056	-1.836199498	0.005558	0.021342	MIR4713HG		15	51037488	51293912	+	561	lincRNA	MIR4713 host gene [Source:HGNC Symbol;Acc:HGNC:53124]
ENSG0000	2.05233	1.037104	0	17.35001	9.096368	6.23737	1.029811	10.89458	-3.37129222	0.00563	0.021585	RBP2		3	139452884	139480747	-	845	protein_cod	retinol binding protein 2 [Source:HGNC Symbol;Acc:HGNC:9920]
ENSG0000	0	0	0	2.891668	10.91564	3.564211	0	5.790507	-4.890725984	0.005648	0.021638	RF00019		20	5113993	5114083	-	91	misc_RNA	
ENSG0000	5.130825	5.185518	3.377067	25.06112	17.2831	13.36579	4.56447	18.57	-2.017006477	0.005694	0.021791	ENPP7P8		11	71711740	71781183	+	1607	transcribed_	ectonucleotide pyrophosphatase/phosphodiesterase 7 pseudogene 8 [Source:HGNC Symbol;Acc:HGNC:48691]
ENSG0000	0	0	0	6.747225	4.548184	5.346317	0	5.547242	-4.826730822	0.005701	0.021808	TMEM256-F		17	7389734	7404097	-	2557	protein_cod	TMEM256-PLSCR3 readthrough (NMD candidate) [Source:HGNC Symbol;Acc:HGNC:49186]
ENSG0000	23.6018	22.81628	18.01103	48.19447	40.02402	50.79001	21.47637	46.33617	-1.106745051	0.005711	0.021839	AC018690.1		2	99405218	99405843	+	626	antisense	novel transcript
ENSG0000	3.078495	4.148415	7.879823	27.95279	17.2831	14.25685	5.035578	19.83091	-1.986592357	0.005718	0.021859	MIR8070		11	11783135	11783222	-	88	miRNA	microRNA 8070 [Source:HGNC Symbol;Acc:HGNC:50274]
ENSG0000	1.026165	1.037104	3.377067	13.49445	13.64455	9.801581	1.813445	12.31353	-2.784488994	0.005726	0.021883	C1orf195		1	15164344	15171317	-	559	lincRNA	chromosome 1 open reading frame 195 [Source:HGNC Symbol;Acc:HGNC:32332]
ENSG0000	3.078495	1.037104	0	17.35001	5.457821	13.36579	1.371866	12.05787	-3.106829888	0.005727	0.021886	AC080129.2		3	194130616	194131201	-	430	antisense	novel transcript
ENSG0000	0	0	1.125689	7.711115	8.186731	7.128423	0.37523	7.675423	-4.33413518	0.005738	0.021923	AC055855.2		15	66278498	66293357	-	578	antisense	uncharacterized LOC105370868 [Source:NCBI gene;Acc:105370868]
ENSG0000	4.10466	1.037104	4.502756	16.38612	13.64455	16.93	3.21484	15.65356	-2.291143153	0.005752	0.021968	AL365436.1		1	151557446	151557940	+	495	processed_	ribosomal protein S10 (RPS10) pseudogene
ENSG0000	0	5.185518	2.251378	10.60278	21.83128	12.47474	2.478966	14.9696	-2.595897379	0.005756	0.021981	TMSB15B-A	X		103917411	103919548	-	1311	antisense	TMSB15B antisense RNA 1 [Source:HGNC Symbol;Acc:HGNC:53858]
ENSG0000	13.34015	20.74207	22.51378	53.01391	35.47584	40.98843	18.86533	43.15939	-1.196060351	0.005757	0.021981	ECEL1P2		2	232385750	232387457	-	1708	transcribed_	endothelin converting enzyme like 1 pseudogene 2 [Source:HGNC Symbol;Acc:HGNC:14019]
ENSG0000	21.54947	9.333933	25.89085	47.23058	40.02402	45.4437	18.92475	44.23276	-1.229437902	0.005792	0.022078	C20orf204		20	64034344	64039962	+	5480	protein_cod	chromosome 20 open reading frame 204 [Source:HGNC Symbol;Acc:HGNC:27655]
ENSG0000	2.05233	2.074207	3.377067	18.3139	10.006	13.36579	2.501202	13.89523	-2.480601523	0.005794	0.022078	AL163952.1		14	55896547	55962970	+	1516	processed_t	novel transcript
ENSG0000	7.183155	16.59366	14.63396	37.59168	30.01801	32.96896	12.80359	33.52622	-1.391215559	0.005796	0.022078	SAP30L-AS1		5	154329437	154445850	-	1972	antisense	SAP30L antisense RNA 1 (head to head) [Source:HGNC Symbol;Acc:HGNC:26760]
ENSG0000	2.05233	3.111311	2.251378	20.24168	10.91564	10.69263	2.471673	13.94998	-2.492799911	0.005832	0.022202	AC138466.5		12	132100013	132100820	-	808	TEC	novel transcript
ENSG0000	0	0	0	4.819447	5.457821	6.23737	0	5.504879	-4.81713504	0.005845	0.022243	ZNF833P		19	11680944	11686259	+	1592	transcribed_	zinc finger protein 833, pseudogene [Source:HGNC Symbol;Acc:HGNC:33819]
ENSG0000	0	3.111311	0	16.38612	7.277095	9.801581	1.037104	11.15493	-3.403370859	0.005894	0.022421	AC008740.1		16	21347139	21352138	+	255	unprocessed	BTG3 associated nuclear protein (BNAP) pseudogene
ENSG0000	12.31398	11.40814	23.63947	33.73613	30.92765	53.46317	15.7872	39.37565	-1.327191653	0.005935	0.022551	AC092119.2		16	21950218	21951708	-	1491	lincRNA	novel transcript
ENSG0000	0	0	1.125689	6.747225	3.638547	13.36579	0.37523	7.917188	-4.380424289	0.005937	0.022555	AL450124.1		20	26018832	26020684	+	353	unprocessed	zinc-finger pseudogene
ENSG0000	5.130825	22.81628	13.50827	33.73613	40.93366	36.53317	13.81846	37.06765	-1.424721625	0.005951	0.022587	SMC5-AS1		9	70193997	70258866	-	1818	antisense	SMC5 antisense RNA 1 (head to head) [Source:HGNC Symbol;Acc:HGNC:48718]
ENSG0000	13.34015	26.9647	4.502756	0.963889	1.819274	4.455264	14.93587	2.412809	2.622139863	0.005964	0.022627	RBPMS-AS1		8	30382119	30385401	-	1171	antisense	RBPMS antisense RNA 1 [Source:HGNC Symbol;Acc:HGNC:48721]
ENSG0000	29.75879	34.22442	16.88534	76.14726	57.30712	40.09738	26.95618	57.85058	-1.095108142	0.006043	0.022899	LINC02014		3	130089433	130094304	-	713	lincRNA	long intergenic non-protein coding RNA 2014 [Source:HGNC Symbol;Acc:HGNC:52849]
ENSG0000	0	0	0	13.49445	1.819274	2.673159	0	5.995628	-4.935248432	0.006075	0.023004	BMP2KL	X		74185929	74187153	+	1225	processed_	BMP2 inducible kinase-like, pseudogene [Source:HGNC Symbol;Acc:HGNC:17080]
ENSG0000	20.5233	18.66787	19.13671	29.88057	63.67458	40.98843	19.44263	44.84786	-1.206934577	0.006076	0.023005	LIPE-AS1		19	42397128	42652355	+	4208	antisense	LIPE antisense RNA 1 [Source:HGNC Symbol;Acc:HGNC:48589]
ENSG0000	2.05233	8.29683	1.125689	17.35001	24.56019	12.47474	3.82495	18.12831	-2.232621216	0.006081	0.023022	AL162730.1		9	69107926	69108217	-	292	processed_	adenylate kinase 3 (AK3) pseudogene
ENSG0000	4.10466	4.148415	2.251378	18.3139	12.73492	16.93	3.501484	15.99294	-2.183025573	0.006103	0.023099	AC008735.5		19	55467438	55467934	-	497	TEC	TEC
ENSG0000	12.31398	9.333933	5.628445	30.84446	14.55419	40.09738	9.09212	28.49868	-1.641942334	0.006124	0.023166	AC104819.1		4	151259503	151262849	+	486	unprocessed	trypsin domain contaiing protein pseudogene
ENSG0000	6.15699	4.148415	3.377067	20.24168	19.10237	15.1479	4.560824	18.16398	-1.986935453	0.006125	0.023166	AC093627.4		7	149571	155465	+	5175	lincRNA	uncharacterized LOC100507642 [Source:NCBI gene;Acc:100507642]
ENSG0000	6.15699	12.44524	10.1312	31.80835	49.12039	11.58369	9.577812	30.83747	-1.687468577	0.006149	0.023249	NME1-NME		17	51153590	51171744	+	1241	protein_cod	NME1-NME2 readthrough [Source:HGNC Symbol;Acc:HGNC:33531]
ENSG0000	11.28782	4.148415	1.125689	0	0	0	5.52064	0	5.030858542	0.00615	0.023249	LINC01118		2	46698940	46822804	+	3360	sense_overl	long intergenic non-protein coding RNA 1118 [Source:HGNC Symbol;Acc:HGNC:49261]
ENSG0000	2.05233	3.111311	0	13.49445	13.64455	9.801581	1.721214	12.31353	-2.814426446	0.006161	0.023286	AC130895.1		12	116174502	116181295	-	493	sense_intro	novel transcript, sense intronic to MED13L
ENSG0000	0	0	0	8.675004	2.72891	5.346317	0	5.58341	-4.83494958	0.006168	0.02331	AC104241.1		11	44719392	44736692	-	628	lincRNA	novel transcript

ENSG0000	1.026165	1.037104	4.502756	4.819447	22.74092	15.1479	2.188675	14.23609	-2.727625582	0.006178	0.023343	NECAB1	8	90791550	90959408	+	6408	protein_cod	N-terminal EF-hand calcium binding protein 1 [Source:HGNC Symbol;Acc:HGNC:20983]
ENSG0000	20.5233	11.40814	12.38258	40.48335	34.5662	33.86001	14.77134	36.30319	-1.29331767	0.006195	0.02339	CYP2F2P	19	40818414	40826772	-	1547	transcribed_	cytochrome P450 family 2 subfamily F member 2, pseudogene [Source:HGNC Symbol;Acc:HGNC:18851]
ENSG0000	1.026165	2.074207	2.251378	13.49445	9.096368	13.36579	1.783917	11.98554	-2.755320764	0.006211	0.023438	AC080038.3	17	62679403	62681354	-	1952	TEC	TEC
ENSG0000	0	0	0	9.638893	4.548184	2.673159	0	5.620079	-4.843285976	0.006218	0.023463	RPL23AP14	12	2781904	2782371	+	468	processed_p	ribosomal protein L23a pseudogene 14 [Source:HGNC Symbol;Acc:HGNC:21007]
ENSG0000	0	0	0	4.819447	3.638547	8.019476	0	5.49249	-4.814267225	0.006266	0.023624	AHCYP2	9	120720673	120721972	+	1300	processed_p	adenosylhomocysteinase pseudogene 2 [Source:HGNC Symbol;Acc:HGNC:44994]
ENSG0000	0	0	2.251378	9.638893	12.73492	7.128423	0.750459	9.834077	-3.772536865	0.00627	0.023634	AL035458.1	20	34245953	34246537	-	585	processed_p	density-regulated protein (DENR) pseudogene
ENSG0000	3.078495	0	0	8.675004	20.01201	5.346317	1.026165	11.34444	-3.436025678	0.006411	0.024082	RGS4	1	163068775	163076802	+	4836	protein_cod	regulator of G protein signaling 4 [Source:HGNC Symbol;Acc:HGNC:10000]
ENSG0000	1.026165	0	0	9.638893	3.638547	9.801581	0.342055	7.693007	-4.336951703	0.006419	0.024102	AC130456.4	16	19410729	19411662	-	665	antisense	novel transcript, antisense to TMC5
ENSG0000	5.130825	10.37104	2.251378	24.09723	12.73492	30.2958	5.917747	22.37598	-1.909015476	0.006475	0.02429	Z97192.1	22	49548681	49556473	+	3894	lincRNA	novel transcript
ENSG0000	0	3.111311	0	6.747225	16.37346	9.801581	1.037104	10.97409	-3.383094422	0.006524	0.024461	SPDYE19P	7	5031608	5037831	-	713	unprocessed	speedy/RINGO cell cycle regulator family member E19, pseudogene [Source:HGNC Symbol;Acc:HGNC:51515]
ENSG0000	14.36631	9.333933	10.1312	19.27779	34.5662	39.20633	11.27715	31.01677	-1.459763339	0.006546	0.024542	COL25A1	4	108808725	109302752	-	8744	protein_cod	collagen type XXV alpha 1 chain [Source:HGNC Symbol;Acc:HGNC:18603]
ENSG0000	0	0	0	4.819447	9.096368	2.673159	0	5.529658	-4.822813022	0.006558	0.024581	AL136980.1	9	5719021	5720244	-	543	antisense	novel transcript
ENSG0000	6.15699	20.74207	1.125689	47.23058	33.65656	18.71211	9.341584	33.19975	-1.817597527	0.00657	0.024616	AP001429.1	21	37100814	37101343	+	530	sense_intro	novel transcript, sense intronic to TTC3
ENSG0000	1.026165	0	5.628445	3.855557	26.37947	16.03895	2.218203	15.42466	-2.831439334	0.006593	0.024679	AC005332.1	17	68246629	68247938	-	1310	antisense	novel transcript, antisense to AMZ2
ENSG0000	12.31398	26.9647	9.005513	49.15836	40.02402	32.0779	16.09473	40.42009	-1.321719748	0.006742	0.025166	ALOX15P1	17	6657034	6693577	+	4151	transcribed_	arachidonate 15-lipoxygenase pseudogene 1 [Source:HGNC Symbol;Acc:HGNC:13742]
ENSG0000	0	0	0	8.675004	1.819274	6.23737	0	5.577216	-4.833598633	0.006749	0.025188	U62317.1	22	50542305	50542906	-	602	lincRNA	novel transcript
ENSG0000	20.5233	25.92759	12.38258	47.23058	52.75894	33.86001	19.61116	44.61651	-1.180011112	0.006788	0.02532	AC098820.1	2	216479030	216498761	-	1435	processed_t	uncharacterized LOC100507554 [Source:NCBI gene;Acc:100507554]
ENSG0000	1.026165	0	0	3.855557	11.82528	7.128423	0.342055	7.603086	-4.322381553	0.006795	0.02534	ATP8A2P2	13	32477274	32478841	+	1568	processed_p	ATPase phospholipid transporting 8A2 pseudogene 2 [Source:HGNC Symbol;Acc:HGNC:39663]
ENSG0000	0	0	0	7.711115	1.819274	7.128423	0	5.552937	-4.828059951	0.006806	0.025376	AC020558.3	17	17616043	17617745	+	1703	TEC	novel transcript
ENSG0000	0	4.148415	2.251378	11.56667	10.006	18.71211	2.133264	13.42826	-2.659240046	0.00681	0.025389	CFI	4	109740694	109802179	-	4875	protein_cod	complement factor I [Source:HGNC Symbol;Acc:HGNC:5394]
ENSG0000	0	0	0	10.60278	4.548184	1.782106	0	5.644357	-4.848916656	0.00682	0.025423	AC128687.2	3	113947005	113947570	-	566	antisense	novel transcript, antisense to GRAMD1C
ENSG0000	2.05233	4.148415	10.1312	17.35001	19.10237	24.94948	5.443982	20.46729	-1.928254001	0.006831	0.025447	DUSP8P3	10	46774421	46776180	-	1760	processed_p	dual specificity phosphatase 8 pseudogene 3 [Source:HGNC Symbol;Acc:HGNC:3077]
ENSG0000	26.68029	35.26153	19.13671	63.6167	61.8553	41.87948	27.02618	55.78383	-1.040563233	0.006832	0.025447	AC004706.1	17	6653464	6655009	-	879	lincRNA	novel transcript
ENSG0000	1.026165	8.29683	2.251378	9.638893	24.56019	20.49422	3.858124	18.2311	-2.236598789	0.006838	0.025465	AC026124.1	12	65281657	65286728	+	3457	sense_intro	novel transcript
ENSG0000	6.15699	5.185518	3.377067	20.24168	25.46983	11.58369	4.906525	19.0984	-1.953406453	0.006854	0.025519	ZNF423	16	49487524	49857919	-	8997	protein_cod	zinc finger protein 423 [Source:HGNC Symbol;Acc:HGNC:16762]
ENSG0000	16.41864	21.77918	13.50827	53.9778	35.47584	32.0779	17.23536	40.51051	-1.227875049	0.006869	0.025563	ITGAD	16	31393312	31426505	+	4411	protein_cod	integrin subunit alpha D [Source:HGNC Symbol;Acc:HGNC:6146]
ENSG0000	2.05233	0	1.125689	6.747225	10.006	14.25685	1.05934	10.33669	-3.286942362	0.006891	0.025635	FAM83C-AS	20	35285251	35285756	+	352	antisense	FAM83C antisense RNA 1 [Source:HGNC Symbol;Acc:HGNC:16113]
ENSG0000	0	1.037104	0	8.675004	6.367458	7.128423	0.345701	7.390295	-4.278887928	0.006904	0.025674	RNU1-120P	1	148263476	148263632	+	157	snRNA	RNA, U1 small nuclear 120, pseudogene [Source:HGNC Symbol;Acc:HGNC:48462]
ENSG0000	20.5233	15.55656	18.01103	53.9778	23.65056	50.79001	18.03029	42.80612	-1.246257167	0.006916	0.025715	AC010531.6	16	87317509	87318043	+	401	antisense	uncharacterized LOC101928659 [Source:NCBI gene;Acc:101928659]
ENSG0000	13.34015	12.44524	5.628445	32.77224	30.92765	24.05843	10.47128	29.25277	-1.473297595	0.006935	0.025779	SLC9A9	3	143265222	143848531	-	4087	protein_cod	solute carrier family 9 member A9 [Source:HGNC Symbol;Acc:HGNC:20653]
ENSG0000	7.183155	5.185518	3.377067	26.02501	20.92165	12.47474	5.24858	19.80713	-1.906629934	0.006937	0.025781	FTLP12	17	16391434	16391882	+	449	processed_p	ferritin light chain pseudogene 12 [Source:HGNC Symbol;Acc:HGNC:37961]
ENSG0000	0	1.037104	2.251378	13.49445	17.2831	2.673159	1.096161	11.15024	-3.374300232	0.006942	0.025795	AC087742.1	17	4267691	4268430	+	740	lincRNA	novel transcript
ENSG0000	0	1.037104	1.125689	10.60278	9.096368	8.019476	0.720931	9.239542	-3.698927756	0.006976	0.025902	AC087893.1	12	108833721	108834384	-	664	sense_intro	novel transcript, sense intronic to SSH1
ENSG0000	24.62796	25.92759	23.63947	8.675004	9.096368	8.019476	24.73167	8.596949	1.52521282	0.006985	0.025925	RF00019	17	40391232	40391338	-	107	misc_RNA	
ENSG0000	12.31398	6.222622	9.005513	35.6639	30.92765	16.03895	9.180705	27.5435	-1.582038807	0.007027	0.026075	ALPL	1	21509372	21578412	+	3007	protein_cod	alkaline phosphatase, liver/bone/kidney [Source:HGNC Symbol;Acc:HGNC:438]
ENSG0000	16.41864	12.44524	19.13671	33.73613	43.66257	34.75106	16.0002	37.38325	-1.227669757	0.007072	0.026235	FAM184B	4	17629306	17781512	-	6622	protein_cod	family with sequence similarity 184 member B [Source:HGNC Symbol;Acc:HGNC:29235]
ENSG0000	0	0	0	0.963889	10.91564	5.346317	0	5.741949	-4.879849226	0.007159	0.026525	AL390961.3	10	26695091	26697625	-	2535	TEC	TEC
ENSG0000	1.026165	1.037104	1.125689	21.20557	4.548184	6.23737	1.062986	10.66371	-3.323821971	0.007217	0.026729	SMCR5	17	17776686	17779529	-	2844	antisense	Smith-Magenis syndrome chromosome region, candidate 5 [Source:HGNC Symbol;Acc:HGNC:17918]
ENSG0000	23.6018	15.55656	16.88534	53.01391	41.84329	32.0779	18.68123	42.3117	-1.175879272	0.007239	0.026789	KCTD21-AS1	11	78139771	78175323	+	4095	antisense	KCTD21 antisense RNA 1 [Source:HGNC Symbol;Acc:HGNC:48674]
ENSG0000	0	3.111311	0	6.747225	13.64455	11.58369	1.037104	10.65849	-3.341214195	0.007263	0.026869	DGCR9	22	18970525	19031242	+	4911	processed_t	DiGeorge syndrome critical region gene 9 [Source:HGNC Symbol;Acc:HGNC:17227]
ENSG0000	31.81112	41.48415	64.16428	23.13334	16.37346	22.27632	45.81985	20.59438	1.151215684	0.007314	0.02703	MMP19	12	55835433	55842966	-	4672	protein_cod	matrix metallopeptidase 19 [Source:HGNC Symbol;Acc:HGNC:7165]
ENSG0000	0	4.148415	2.251378	18.3139	3.638547	21.38527	2.133264	14.4459	-2.762893149	0.00732	0.027044	RN7SL648P	X	74242608	74242908	-	301	misc_RNA	RNA, 7SL, cytoplasmic 648, pseudogene [Source:HGNC Symbol;Acc:HGNC:46664]
ENSG0000	3.078495	2.074207	0	20.24168	13.64455	4.455264	1.717568	12.7805	-2.868627187	0.007372	0.027222	FALEC	1	150515757	150518032	+	566	lincRNA	focally amplified long non-coding RNA in epithelial cancer [Source:HGNC Symbol;Acc:HGNC:43713]
ENSG0000	0	0	1.125689	13.49445	8.186731	1.782106	0.37523	7.821096	-4.357955064	0.007395	0.027283	AC097493.2	4	9563171	9563950	+	780	processed_p	ribosomal protein S24 (RPS24) pseudogene
ENSG0000	0	0	0	5.783336	4.548184	5.346317	0	5.225946	-4.741127334	0.007486	0.027592	HIST3H2BA	1	228464103	228464626	-	524	unitary_pse	histone cluster 3 H2B family member a (pseudogene) [Source:HGNC Symbol;Acc:HGNC:20515]
ENSG0000	15.39248	2.074207	5.628445	26.02501	25.46983	25.84053	7.698376	25.77846	-1.737442837	0.007494	0.027612	PCDHA10	5	140855883	141012344	+	15224	protein_cod	protocadherin alpha 10 [Source:HGNC Symbol;Acc:HGNC:8664]
ENSG0000	15.39248	22.81628	22.51378	2.891668	6.367458	8.019476	20.24085	5.759534	1.804763034	0.007499	0.027627	SEMA3E	7	83363238	83649113	-	8424	protein_cod	semaphorin 3E [Source:HGNC Symbol;Acc:HGNC:10727]
ENSG0000	8.209321	6.222622	5.628445	24.09723	25.46983	16.03895	6.686796	21.86867	-1.70516042	0.007513	0.027667	NID2	14	52004803	52069228	-	6392	protein_cod	nidogen 2 [Source:HGNC Symbol;Acc:HGNC:13389]
ENSG0000	2.05233	0	5.628445	8.675004	20.92165	14.25685	2.560258	14.61783	-2.539566802	0.007554	0.027798	AC116021.1	11	46238382	46239267	+	653	lincRNA	novel transcript
ENSG0000	0	1.037104	1.125689	8.675004	6.367458	12.47474	0.720931	9.172401	-3.689784391	0.007556	0.0278	LINC02288	14	77041064	77069503	+	2924	lincRNA	long intergenic non-protein coding RNA 2288 [Source:HGNC Symbol;Acc:HGNC:27505]
ENSG0000	18.47097	6.222622	14.63396	0.963889	4.548184	0.891053	13.10918	2.134375	2.613563791	0.007564	0.027825	AC009630.2	8	41540381	41545044	-	1189	antisense	novel transcript, antisense to GINS4
ENSG0000	1.026165	2.074207	4.502756	13.49445	7.277095	21.38527	2.534376	14.05227	-2.489804003	0.007602	0.027945	TMEM211	22	24934954	24946695	-	1123	protein_cod	transmembrane protein 211 [Source:HGNC Symbol;Acc:HGNC:33725]
ENSG0000	16.41864	22.81628	14.63396	38.55557	43.66257	38.31527	17.95629	40.1778	-1.159405454	0.007618	0.027998	AL359220.1	14	61298164	61322818	-	4852	antisense	novel transcript, antisense to PRKCH
ENSG0000	0	4.148415	4.502756	19.27779	10.006	16.03895	2.883724	15.10758	-2.401999979	0.007618	0.027998	AC010547.1	16	71462278	71465941	+	2793	antisense	novel transcript, antisense to ZNF23
ENSG0000	2.05233	0	0	7.711115	14.55419	6.23737	0.68411	9.500891	-3.761445738	0.007621	0.028005	LINC01397	1	110082688	110109719	+	579	antisense	long intergenic non-protein coding RNA 1397 [Source:HGNC Symbol;Acc:HGNC:50677]
ENSG0000	6.15699	8.29683	5.628445	17.35001	25.46983	22.27632	6.694088	21.69872	-1.69506063	0.007647	0.028089	AC092168.2	2	100822661	100847220	+	717	sense_intro	novel transcript
ENSG0000	2.05233	0	7.879823	10.60278	26.37947	14.25685	3.310718	17.0797	-2.39407773	0.007669	0.028164	CD7	17	82314868	82317602	-	2735	protein_cod	CD7 molecule [Source:HGNC Symbol;Acc:HGNC:1695]
ENSG0000	15.39248	21.77918	20.2624	56.86947	23.65056	53.46317	19.14469	44.66107	-1.222270547	0.007669	0.028164	AL022323.4	22	25052122	25065241	-	13120	antisense	novel transcript
ENSG0000	4.10466	13.48235	9.005513	20.24168	32.74693	26.73159	8.864174	26.5734	-1.586372244	0.007752	0.028419	ATP2B1-AS1	12	89708959	89712590	+	3632	lincRNA	ATP2B1 antisense RNA 1 [Source:HGNC Symbol;Acc:HGNC:27883]
ENSG0000	0	0	0	8.675004	3.638547	3.564211	0	5.292588	-4.757109151	0.007798	0.028579	AC128688.1	3	112185480	112185998	-	519	processed_p	chromosome 1 open reading frame 106 (C1orf106) pseudogene
ENSG0000	2.05233	5.185518	3.377067	19.27779	9.096368	19.60316	3.538305	15.99244	-2.175858651	0.00782	0.028637	AC010359.1	5	65924629	65925135	-	507	lincRNA	novel transcript
ENSG0000	8.209321	14.51945	19.13671	26.02501	31.83729	48.11685	13.95516	35.32638	-1.347840101	0.007843	0.028719	ACOXL-AS1	2	111098345	111115588	-	3432	antisense	ACOXL antisense RNA 1 [Source:HGNC Symbol;Acc:HGNC:41112]
ENSG0000	0	0	0	4.819447	7.277095	3.564211	0	5.220251	-4.739797752	0.007848	0.028733	SEPT7P6	7	152367519	152368661	+	1143	processed_p	septin 7 pseudogene 6 [Source:HGNC Symbol;Acc:HGNC:38042]
ENSG0000	0	1.037104	0	3.855557	10.006	8.019476	0.345701	7.293679	-4.262613379	0.007877	0.02883	AC010997.4	10	75408973	75409326	-	354	lincRNA	novel transcript
ENSG0000	1.026165	0	0	7.711115	13.64455	1.782106	0.342055	7.712591	-4.340310538	0.007882	0.028843	OR7E59P	7	6879627	6880626	-	1000	unprocessed	olfactory receptor family 7 subfamily E member 59 pseudogene [Source:HGNC Symbol;Acc:HGNC:8434]
ENSG0000	24.62796	12.44524	21.38809	6.747225	5.457821	4.455264	19.4871	5.553437	1.813944012	0.007924	0.028966	PFN1P4	1	148129497	148129898	+	402	processed_p	profilin 1 pseudogene 4 [Source:HGNC Symbol;Acc:HGNC:42992]
ENSG0000	5.130825	2.074207	2.251378	11.56667	10.91564	23.16737	3.152137	15.21656	-2.265792467	0.007972	0.029098	Z97192.2	22	49572264	49575426	-	2379	lincRNA	novel transcript
ENSG0000	0	0	0	10.60278	0.909637	5.346317	0	5.619579	-4.843435643	0.007989	0.029143	RPL7P14	2	203040108	203040899	+	741	processed_p	ribosomal protein L7 pseudogene 14 [Source:HGNC Symbol;Acc:HGNC:35984]
ENSG0000	11.28782	7.259726	14.63396	28.91668	30.92765	27.62264	11.0605	29.15566	-1.403516521	0.008083	0.029459	SNORD62B	9	131490486	131490571	+	86	snoRNA	small nucleolar RNA, C/D box 62B [Source:HGNC Symbol;Acc:HGNC:23031]

ENSG0000	19.49714	19.70497	25.89085	3.855557	6.367458	9.801581	21.69765	6.674865	1.692477671	0.008138	0.02964	AC008013.1	12	30978308	31006010	-	1995	transcribed_	tetraspanin family pseudogene
ENSG0000	0	3.111311	0	2.891668	19.10237	11.58369	1.037104	11.19258	-3.413709501	0.008147	0.029664	CYCSP2	15	41108878	41109178	+	301	processed_p	cytochrome c, somatic pseudogene 2 [Source:HGNC Symbol;Acc:HGNC:24410]
ENSG0000	19.49714	14.51945	13.50827	30.84446	30.92765	50.79001	15.84162	37.52071	-1.242877742	0.008183	0.029771	FAM19A2	12	61708259	62279150	-	9255	protein_cod	family with sequence similarity 19 member A2, C-C motif chemokine like [Source:HGNC Symbol;Acc:HGNC:21589]
ENSG0000	1.026165	3.111311	3.377067	6.747225	11.82528	23.16737	2.504848	13.91329	-2.485464749	0.008188	0.029786	AC011939.1	15	65771176	65772139	-	964	processed_p	DnaJ (Hsp40) homolog, subfamily B, member 14 pseudogene
ENSG0000	4.10466	1.037104	0	13.49445	10.006	12.47474	1.713921	11.99173	-2.779853207	0.008193	0.029797	HLA-DRB1	6	32578769	32589848	-	1229	protein_cod	major histocompatibility complex, class II, DR beta 1 [Source:HGNC Symbol;Acc:HGNC:4948]
ENSG0000	0	0	3.377067	11.56667	5.457821	15.1479	1.125689	10.72413	-3.305183279	0.008206	0.029831	AC078962.3	12	64654060	64655019	-	482	antisense	novel transcript
ENSG0000	14.36631	15.55656	19.13671	28.91668	43.66257	40.09738	16.35319	37.55888	-1.203755311	0.008213	0.02985	AC099778.1	3	47379089	47380999	-	1911	antisense	novel transcript, antisense to PTPN23
ENSG0000	13.34015	7.259726	12.38258	1.927779	0	2.673159	10.99415	1.533646	2.840897506	0.008229	0.029887	TGM5	15	43232595	43266857	-	3631	protein_cod	transglutaminase 5 [Source:HGNC Symbol;Acc:HGNC:11781]
ENSG0000	1.026165	3.111311	0	7.711115	11.82528	13.36579	1.379159	10.9674	-2.9700262	0.008244	0.029936	NKAIN3-IT1	8	62977861	62984900	+	7040	lincRNA	NKAIN3 intronic transcript [Source:HGNC Symbol;Acc:HGNC:48595]
ENSG0000	1.026165	0	4.502756	9.638893	13.64455	13.36579	1.842974	12.21641	-2.763357697	0.008249	0.029948	RF00015	1	150608507	150608623	-	117	snRNA	
ENSG0000	13.34015	13.48235	6.754134	34.70002	31.83729	23.16737	11.19221	29.90156	-1.409912295	0.008257	0.029974	HACD4	9	20999515	21031636	-	9152	protein_cod	3-hydroxyacyl-CoA dehydratase 4 [Source:HGNC Symbol;Acc:HGNC:20920]
ENSG0000	4.10466	4.148415	1.125689	14.45834	16.37346	13.36579	3.126255	14.73253	-2.22257912	0.008288	0.030071	PLBD1	12	14503661	14568349	-	2841	protein_cod	phospholipase B domain containing 1 [Source:HGNC Symbol;Acc:HGNC:26215]
ENSG0000	0	0	1.125689	8.675004	7.277095	5.346317	0.37523	7.099472	-4.220477864	0.008292	0.030083	AC008060.3	7	155421206	155457099	-	534	lincRNA	novel transcript
ENSG0000	7.183155	3.111311	5.628445	24.09723	17.2831	16.03895	5.307637	19.13976	-1.849164617	0.008325	0.03019	LINC00921	16	3263743	3267567	+	2468	lincRNA	long intergenic non-protein coding RNA 921 [Source:HGNC Symbol;Acc:HGNC:26830]
ENSG0000	19.49714	21.77918	12.38258	35.6639	45.48184	39.20633	17.8863	40.11736	-1.16119011	0.008367	0.030317	PCAT6	1	202810954	202812156	+	890	antisense	prostate cancer associated transcript 6 [Source:HGNC Symbol;Acc:HGNC:43714]
ENSG0000	45.15126	45.63256	59.66152	18.3139	33.65656	20.49422	50.14845	24.15489	1.050638204	0.008368	0.030317	ZNF649	19	51889224	51905040	-	4365	protein_cod	zinc finger protein 649 [Source:HGNC Symbol;Acc:HGNC:25741]
ENSG0000	17.44481	14.51945	25.89085	36.62779	41.84329	48.11685	19.28504	42.19598	-1.135740179	0.008381	0.030349	FRZB	2	182833275	182867162	-	3162	protein_cod	frizzled related protein [Source:HGNC Symbol;Acc:HGNC:3959]
ENSG0000	2.05233	6.222622	3.377067	35.6639	8.186731	10.69263	3.884006	18.18109	-2.221658275	0.008392	0.030381	LINC02443	12	5233997	5243151	-	1393	lincRNA	long intergenic non-protein coding RNA 2443 [Source:HGNC Symbol;Acc:HGNC:53375]
ENSG0000	7.183155	14.51945	6.754134	34.70002	24.56019	22.27632	9.485581	27.17884	-1.512951723	0.008439	0.030522	ASIP	20	34194569	34269344	+	778	protein_cod	agouti signaling protein [Source:HGNC Symbol;Acc:HGNC:745]
ENSG0000	5.130825	1.037104	1.125689	15.42223	10.006	15.1479	2.431206	13.52538	-2.461296075	0.008485	0.030666	AC106038.1	8	90646441	90656598	+	3808	antisense	novel transcript
ENSG0000	10.26165	9.333933	13.50827	18.3139	28.19874	43.66159	11.03462	30.05808	-1.451781354	0.008488	0.030672	AC006449.2	17	38703480	38706261	-	486	antisense	novel transcript, antisense to MLLT6
ENSG0000	2.05233	8.29683	20.2624	33.73613	20.92165	40.09738	10.20385	31.58505	-1.644471531	0.008502	0.030715	AC011495.1	19	49719498	49720081	-	584	processed_p	ribosomal protein S9 (RPS9) pseudogene
ENSG0000	2.05233	1.037104	1.125689	5.783336	6.367458	21.38527	1.405041	11.17869	-2.989135183	0.008507	0.030729	RNU6-1263P	3	98908459	98908564	+	106	snRNA	RNA, U6 small nuclear 1263, pseudogene [Source:HGNC Symbol;Acc:HGNC:48226]
ENSG0000	1.026165	5.185518	7.879823	12.53056	17.2831	26.73159	4.697169	18.84842	-2.020238942	0.00852	0.03077	SNRPGP4	17	69318733	69318961	+	229	processed_p	small nuclear ribonucleoprotein polypeptide G pseudogene 4 [Source:HGNC Symbol;Acc:HGNC:39323]
ENSG0000	0	0	0	8.675004	1.819274	5.346317	0	5.280198	-4.754214177	0.00862	0.031092	CASP5	11	104994235	105023168	-	1592	protein_cod	caspase 5 [Source:HGNC Symbol;Acc:HGNC:1506]
ENSG0000	8.209321	15.55656	3.377067	31.80835	25.46983	24.05843	9.047648	27.1122	-1.57297279	0.008678	0.031279	HIC1	17	2054154	2063241	+	8108	protein_cod	HIC ZBTB transcriptional repressor 1 [Source:HGNC Symbol;Acc:HGNC:4909]
ENSG0000	6.15699	2.074207	4.502756	15.42223	17.2831	17.82106	4.244651	16.84213	-1.988913315	0.008751	0.031494	AC009309.1	2	86195590	86196049	+	460	antisense	novel transcript, antisense to IMMT
ENSG0000	4.10466	0	6.754134	15.42223	20.01201	14.25685	3.619598	16.56369	-2.209976546	0.008787	0.031617	C1orf220	1	178542752	178548889	+	2775	lincRNA	chromosome 1 open reading frame 220 (putative) [Source:HGNC Symbol;Acc:HGNC:33805]
ENSG0000	14.36631	7.259726	13.50827	26.9889	23.65056	41.87948	11.71144	30.83965	-1.399953479	0.008825	0.031744	AP001628.1	21	42831040	42836477	-	285	lincRNA	novel transcript
ENSG0000	5.130825	6.222622	0	25.06112	13.64455	13.36579	3.784483	17.35716	-2.176708862	0.008849	0.031811	AL592295.1	1	161406068	161407082	+	884	processed_p	ribosomal protein S2 (RPS2) pseudogene
ENSG0000	0	2.074207	0	16.38612	10.006	2.673159	0.691402	9.688427	-3.782440872	0.008864	0.031855	THSD4-AS1	15	71147650	71189042	-	3015	antisense	THSD4 antisense RNA 1 [Source:HGNC Symbol;Acc:HGNC:51420]
ENSG0000	2.05233	2.074207	1.125689	9.638893	9.096368	15.1479	1.750742	11.29439	-2.681993216	0.008927	0.032047	AC009053.3	16	74422120	74435254	+	665	antisense	uncharacterized LOC107984827 [Source:NCBI gene;Acc:107984827]
ENSG0000	1.026165	3.111311	0	8.675004	17.2831	7.128423	1.379159	11.02884	-2.977330689	0.008953	0.03213	HHATL	3	42692663	42702827	-	2891	protein_cod	hedgehog acyltransferase like [Source:HGNC Symbol;Acc:HGNC:13242]
ENSG0000	10.26165	16.59366	23.63947	28.91668	56.39748	34.75106	16.83159	40.02174	-1.256757225	0.008959	0.032142	PLA2G4C	19	48047843	48110817	-	6613	protein_cod	phospholipase A2 group IVC [Source:HGNC Symbol;Acc:HGNC:9037]
ENSG0000	0	0	0	3.855557	2.72891	8.910529	0	5.164999	-4.726315361	0.008977	0.032189	LILRA4	19	54333185	54339150	-	2171	protein_cod	leukocyte immunoglobulin like receptor A4 [Source:HGNC Symbol;Acc:HGNC:15503]
ENSG0000	5.130825	12.44524	12.38258	44.33891	27.2891	16.03895	9.986216	29.22232	-1.55067151	0.008977	0.032189	ATP5PDP4	12	68642519	68642993	-	475	processed_p	ATP synthase peripheral stalk subunit d pseudogene 4 [Source:HGNC Symbol;Acc:HGNC:49626]
ENSG0000	4.10466	1.037104	1.125689	12.53056	12.73492	11.58369	2.089151	12.28305	-2.542605852	0.00901	0.032283	RNU6-256P	11	122004355	122004451	+	97	snRNA	RNA, U6 small nuclear 256, pseudogene [Source:HGNC Symbol;Acc:HGNC:47219]
ENSG0000	13.34015	21.77918	31.51929	41.44724	50.03002	49.89896	22.21287	47.12541	-1.092174818	0.009102	0.032565	RNASEH2B-A	13	50862172	50910764	-	4642	antisense	RNASEH2B antisense RNA 1 [Source:HGNC Symbol;Acc:HGNC:39967]
ENSG0000	1017.956	481.2161	382.7343	318.0835	272.891	256.6232	627.302	282.5326	1.151071477	0.009128	0.032648	GTF2IP1	7	75185385	75237696	-	7453	transcribed_	general transcription factor IIi pseudogene 1 [Source:HGNC Symbol;Acc:HGNC:4660]
ENSG0000	0	0	1.125689	4.819447	10.91564	5.346317	0.37523	7.027135	-4.20784888	0.009154	0.032729	BTNL3	5	180988845	181006727	+	2440	protein_cod	butyrophilin like 3 [Source:HGNC Symbol;Acc:HGNC:1143]
ENSG0000	19.49714	26.9647	25.89085	44.33891	47.30111	53.46317	24.11756	48.36773	-1.006061989	0.009213	0.032908	AC138409.1	5	34190056	34193653	+	3598	processed_p	integral membrane glycoprotein-like [Source:NCBI gene;Acc:646652]
ENSG0000	0	2.074207	0	16.38612	4.548184	7.128423	0.691402	9.354242	-3.731708333	0.009216	0.032913	FDPSP3	14	54884849	54886213	-	1013	transcribed_	farnesyl diphosphate synthase pseudogene 3 [Source:HGNC Symbol;Acc:HGNC:3634]
ENSG0000	45.15126	34.22442	40.52481	5.783336	19.10237	24.05843	39.96683	16.31471	1.285946363	0.009227	0.032941	AC093788.1	4	163529771	163530697	+	927	lincRNA	novel transcript
ENSG0000	8.209321	9.333933	12.38258	2.891668	0	0.891053	9.975278	1.260907	3.004855524	0.009299	0.033173	AC022165.1	16	84495599	84497495	+	596	antisense	novel transcript, antisense to KIAA1609
ENSG0000	3.078495	6.222622	1.125689	12.53056	11.82528	23.16737	3.475602	15.84107	-2.177384473	0.009324	0.033238	NEUROD2	17	39603536	39609777	-	6242	protein_cod	neuronal differentiation 2 [Source:HGNC Symbol;Acc:HGNC:7763]
ENSG0000	12.31398	10.37104	21.38809	37.59168	41.84329	26.73159	14.69104	35.38885	-1.274627298	0.009343	0.033296	ERBB4	2	211375717	212538841	-	13015	protein_cod	erb-b2 receptor tyrosine kinase 4 [Source:HGNC Symbol;Acc:HGNC:3432]
ENSG0000	14.36631	3.111311	22.51378	37.59168	33.65656	35.64211	13.33047	35.63012	-1.427015518	0.009354	0.033331	SLC11A1	2	218382029	218396894	+	8676	protein_cod	solute carrier family 11 member 1 [Source:HGNC Symbol;Acc:HGNC:10907]
ENSG0000	5.130825	0	0	16.38612	14.55419	7.128423	1.710275	12.68958	-2.863693197	0.009431	0.033571	PRSS55	8	10525546	10554166	+	3135	protein_cod	serine protease 55 [Source:HGNC Symbol;Acc:HGNC:30824]
ENSG0000	12.31398	5.185518	9.005513	18.3139	41.84329	19.60316	8.835004	26.58678	-1.589878036	0.009431	0.033571	ZC3H11A	1	203795654	203854999	+	12798	protein_cod	zinc finger CCCH-type containing 11A [Source:HGNC Symbol;Acc:HGNC:29093]
ENSG0000	1.026165	6.222622	4.502756	13.49445	21.83128	14.25685	3.917181	16.52753	-2.082180061	0.009441	0.033597	RNA5SP195	5	139299852	139299968	+	117	rRNA_pseud	RNA, 5S ribosomal pseudogene 195 [Source:HGNC Symbol;Acc:HGNC:43095]
ENSG0000	4.10466	8.29683	13.50827	20.24168	30.92765	24.94948	8.636586	25.37294	-1.565789415	0.009532	0.033889	ZNF396	18	35366697	35377337	-	6077	protein_cod	zinc finger protein 396 [Source:HGNC Symbol;Acc:HGNC:18824]
ENSG0000	8.209321	20.74207	16.88534	40.48335	41.84329	27.62264	15.27891	36.64976	-1.26369588	0.009703	0.034445	AL353748.2	9	88391362	88391832	-	471	processed_p	developmental pluripotency associated 3 (DPPA3) pseudogene
ENSG0000	1.026165	9.333933	6.754134	23.13334	10.006	32.0779	5.704744	21.73908	-1.934913551	0.009718	0.034489	DNAJB13	11	73950319	73970366	+	3475	protein_cod	DnaJ heat shock protein family (Hsp40) member B13 [Source:HGNC Symbol;Acc:HGNC:30718]
ENSG0000	6.15699	6.222622	12.38258	25.06112	22.74092	24.05843	8.254064	23.95349	-1.545846753	0.009747	0.034577	LEMD1-AS1	1	205373252	205387440	+	2781	antisense	LEMD1 antisense RNA 1 [Source:HGNC Symbol;Acc:HGNC:44132]
ENSG0000	26.68029	32.15021	31.51929	10.60278	18.19274	2.673159	30.1166	10.48956	1.523647673	0.009806	0.034757	STXBP5L	3	120908072	121424761	+	11085	protein_cod	syntaxin binding protein 5 like [Source:HGNC Symbol;Acc:HGNC:30757]
ENSG0000	10.26165	16.59366	14.63396	22.16945	38.20475	40.98843	13.82976	33.78754	-1.292121693	0.009847	0.03489	AC009509.4	12	27700066	27700574	+	509	antisense	novel transcript, antisense to a lncRNA
ENSG0000	42.07277	56.0036	39.39912	26.02501	20.01201	21.38527	45.82516	22.4741	1.030628493	0.009887	0.035028	CYBB	X	37780011	37813461	+	4423	protein_cod	cytochrome b-245 beta chain [Source:HGNC Symbol;Acc:HGNC:2578]
ENSG0000	9.235486	6.222622	10.1312	24.09723	28.19874	20.49422	8.52977	24.2634	-1.51096195	0.009902	0.035075	CFAP99	4	2418974	2462942	+	5198	protein_cod	cilia and flagella associated protein 99 [Source:HGNC Symbol;Acc:HGNC:51180]
ENSG0000	17.44481	5.185518	11.25689	33.73613	31.83729	24.94948	11.29574	30.1743	-1.415625533	0.009947	0.035204	OR7E7P	7	97946987	97947998	-	1012	unprocessed	olfactory receptor family 7 subfamily E member 7 pseudogene [Source:HGNC Symbol;Acc:HGNC:8457]
ENSG0000	5.130825	6.222622	6.754134	26.9889	25.46983	9.801581	6.035861	20.75344	-1.781891484	0.009953	0.035216	TULP1	6	35497874	35512938	-	2958	protein_cod	tubby like protein 1 [Source:HGNC Symbol;Acc:HGNC:12423]
ENSG0000	11.28782	25.92759	9.005513	44.33891	26.37947	43.66159	15.40697	38.12666	-1.301710489	0.009955	0.035216	LINC00271	6	135497801	135716055	+	3825	lincRNA	long intergenic non-protein coding RNA 271 [Source:HGNC Symbol;Acc:HGNC:32526]
ENSG0000	4.10466	2.074207	1.125689	9.638893	10.006	19.60316	2.434852	13.08269	-2.414616011	0.00996	0.035228	PCOLCE-AS1	7	100589402	100604206	-	2705	antisense	PCOLCE antisense RNA 1 [Source:HGNC Symbol;Acc:HGNC:40430]
ENSG0000	25.65413	31.11311	14.63396	57.83336	46.39148	43.66159	23.8004	49.29548	-1.044111469	0.009982	0.035295	AC021087.2	5	213898	217279	-	2881	processed_t	novel transcript
ENSG0000	3.078495	3.111311	0	14.45834	13.64455	8.910529	2.063269	12.33781	-2.556203504	0.009995	0.035327	ZNF101P2	1	192993449	192994677	-	1229	processed_p	zinc finger protein 101 pseudogene 2 [Source:HGNC Symbol;Acc:HGNC:44942]
ENSG0000	1.026165	1.037104	6.754134	15.42223	16.37346	11.58369	2.939134	14.45979	-2.325399311	0.010009	0.035364	AC099811.4	17	42268587	42269807	+	496	antisense	novel transcript
ENSG0000	1.026165	2.074207	0	4.819447	13.64455	10.69263	1.033457	9.718878	-3.210610174	0.010015	0.035382	AC098820.3	2	216483032	216487196	-	976	antisense	novel transcript
ENSG0000	22.57563	29.0389	20.2624	54.94169	56.39748	35.64211	23.95898	48.99376	-1.028898424	0.010035	0.035447	DDR2	1	162631373	162787400	+	10730	protein_cod	discoidin domain receptor tyrosine kinase 2 [Source:HGNC Symbol;Acc:HGNC:2731]
ENSG0000	5.130825	5.185518	1.125689	19.27779	13.64455	15.1479	3.814011	16.02341	-2.055511102	0.010037	0.03545	JPH3	16	87601835	87698156	+	5941	protein_cod	junctophilin 3 [Source:HGNC Symbol;Acc:HGNC:14203]
ENSG0000	13.34015	7.259726	7.879823	24.09723	21.83128	32.96896	9.493232	26.29916	-1.467321451	0.010067	0.035539	LY6E-DT	8	142981738	143018437	-	3853	lincRNA	LY6E divergent transcript [Source:HGNC Symbol;Acc:HGNC:53728]

ENSG0000	0	1.037104	1.125689	13.49445	6.367458	6.23737	0.720931	8.699759	-3.609649071	0.010121	0.035694	AL591846.1	1	206695837	206696269	-	433	processed_p	ribosomal protein S14 (RPS14) pseudogene
ENSG0000	3.078495	1.037104	3.377067	9.638893	15.46383	13.36579	2.497555	12.82284	-2.36821788	0.010183	0.035866	AL022722.1	6	119269133	119269714	-	582	processed_p	60S Ribosomal Protein L13A (RPL13) pseudogene
ENSG0000	7.183155	3.111311	2.251378	25.06112	9.096368	17.82106	4.181948	17.32618	-2.03892314	0.010193	0.035896	RF00004	17	43251829	43252019	-	191	snRNA	
ENSG0000	1.026165	0	0	6.747225	8.186731	5.346317	0.342055	6.760091	-4.150764932	0.010201	0.035917	AC120349.1	18	47878295	47882444	+	282	antisense	novel transcript
ENSG0000	6.15699	14.51945	10.1312	33.73613	27.2891	22.27632	10.26921	27.76718	-1.434251972	0.010207	0.035928	AC106782.1	16	30204316	30209071	-	2707	transcribed_	SAGA complex associated factor 29 pseudogene [Source:NCBI gene;Acc:613038]
ENSG0000	3.078495	6.222622	4.502756	0	0	0	4.601291	0	4.763304526	0.010239	0.036023	LINC02262	4	117314597	117360639	-	735	lincRNA	long intergenic non-protein coding RNA 2262 [Source:HGNC Symbol;Acc:HGNC:53174]
ENSG0000	1.026165	2.074207	0	14.45834	6.367458	8.019476	1.033457	9.615091	-3.190524128	0.010256	0.036072	AC093635.1	2	48440043	48440597	-	555	lincRNA	novel transcript
ENSG0000	1.026165	0	1.125689	5.783336	10.91564	8.910529	0.717285	8.536502	-3.58874622	0.010377	0.036453	AC090286.2	17	18908279	18908865	+	587	transcribed_	RAN binding protein 1 (RANBP1) pseudogene
ENSG0000	0	0	0	11.56667	0.909637	3.564211	0	5.34684	-4.770551614	0.010448	0.036673	AC009806.1	11	12538083	12540967	-	519	lincRNA	novel transcript
ENSG0000	3.078495	3.111311	3.377067	8.675004	15.46383	18.71211	3.188958	14.28365	-2.166857256	0.010508	0.036846	AL121672.2	22	46067356	46069891	-	867	lincRNA	novel transcript
ENSG0000	1.026165	0	6.754134	11.56667	15.46383	15.1479	2.593433	14.05947	-2.472431901	0.010526	0.036899	AC004241.4	12	47768529	47769648	+	1120	sense_intro	novel transcript, sense intronic to SLC48A1
ENSG0000	3.078495	5.185518	3.377067	13.49445	20.01201	13.36579	3.88036	15.62408	-2.0079227	0.010566	0.037022	ACR	22	50738196	50745334	+	2059	protein_cod	acrosin [Source:HGNC Symbol;Acc:HGNC:126]
ENSG0000	0	0	0	4.819447	7.277095	2.673159	0	4.923233	-4.654915646	0.010592	0.037102	LINGO1-AS1	15	77641764	77652288	+	2720	antisense	LINGO1 antisense RNA 1 [Source:HGNC Symbol;Acc:HGNC:27368]
ENSG0000	2.05233	4.148415	3.377067	18.3139	11.82528	12.47474	3.192604	14.20464	-2.154065323	0.010593	0.037102	AL031710.2	16	1707252	1707973	+	722	sense_intro	novel transcript, sense intronic to MAPK8IP3
ENSG0000	1.026165	1.037104	9.005513	14.45834	15.46383	19.60316	3.689594	16.50844	-2.190028834	0.010666	0.037335	IGLV1-51	22	22322472	22322969	+	389	IG_V_gene	immunoglobulin lambda variable 1-51 [Source:HGNC Symbol;Acc:HGNC:5882]
ENSG0000	0	0	1.125689	4.819447	6.367458	8.910529	0.37523	6.699144	-4.139570654	0.010678	0.037374	RF01225	4	139436368	139436494	-	127	snoRNA	
ENSG0000	9.235486	5.185518	11.25689	32.77224	27.2891	15.1479	8.559298	25.06975	-1.553156452	0.010715	0.037481	AC131212.2	12	132565071	132566425	+	1355	TEC	novel transcript
ENSG0000	14.36631	12.44524	5.628445	26.02501	29.10838	30.2958	10.81333	28.4764	-1.389343191	0.010722	0.037488	AL135910.1	6	169426420	169452475	-	4107	lincRNA	novel transcript
ENSG0000	6.15699	15.55656	6.754134	0.963889	1.819274	0.891053	9.489227	1.224739	2.953623995	0.010759	0.037596	RERG	12	15107783	15348675	-	4157	protein_cod	RAS like estrogen regulated growth inhibitor [Source:HGNC Symbol;Acc:HGNC:15980]
ENSG0000	0	0	0	2.891668	6.367458	5.346317	0	4.868481	-4.64084974	0.010793	0.037697	AL391987.6	9	61858710	61858908	+	199	processed_p	family with sequence similarity 27-like (FAM27L) pseudogene
ENSG0000	18.47097	24.89049	16.88534	43.37502	44.5722	38.31527	20.08227	42.0875	-1.064922482	0.010799	0.037708	AL139424.3	1	10381906	10387150	-	2381	lincRNA	novel transcript
ENSG0000	0	2.074207	0	8.675004	4.548184	13.36579	0.691402	8.862994	-3.65764842	0.010841	0.037826	SERPINC1	1	173903804	173917378	-	1768	protein_cod	serpin family C member 1 [Source:HGNC Symbol;Acc:HGNC:775]
ENSG0000	12.31398	11.40814	14.63396	28.91668	32.74693	30.2958	12.78536	30.65313	-1.264243297	0.01089	0.03797	NXNL2	9	88534033	88584274	+	2498	protein_cod	nucleoredoxin like 2 [Source:HGNC Symbol;Acc:HGNC:30482]
ENSG0000	0	0	0	7.711115	5.457821	1.782106	0	4.98368	-4.67035776	0.010896	0.037987	AC068533.4	7	66087761	66152277	+	922	protein_cod	novel protein
ENSG0000	1.026165	3.111311	4.502756	15.42223	13.64455	11.58369	2.880077	13.55016	-2.246594724	0.010915	0.038036	DPH6-DT	15	35546195	35859001	+	1531	lincRNA	DPH6 divergent transcript [Source:HGNC Symbol;Acc:HGNC:44147]
ENSG0000	0	0	0	2.891668	7.277095	4.455264	0	4.874676	-4.642447897	0.010925	0.038066	RN7SL255P	5	104260531	104260826	-	296	misc_RNA	RNA, 7SL, cytoplasmic 255, pseudogene [Source:HGNC Symbol;Acc:HGNC:46271]
ENSG0000	2.05233	0	1.125689	9.638893	13.64455	5.346317	1.05934	9.543254	-3.169142572	0.010944	0.03812	AC021752.1	15	50839875	50908599	-	2669	lincRNA	novel transcript
ENSG0000	1.026165	5.185518	0	13.49445	15.46383	8.910529	2.070561	12.62293	-2.588077661	0.010994	0.038283	DDX3P1	X	74121012	74131923	+	1950	processed_p	DEAD-box helicase 3 pseudogene 1 [Source:HGNC Symbol;Acc:HGNC:42171]
ENSG0000	9.235486	4.148415	9.005513	19.27779	24.56019	23.16737	7.463138	22.33512	-1.585220463	0.010998	0.03829	AC009086.3	16	29595130	29596137	-	1008	TEC	novel transcript
ENSG0000	0	0	1.125689	10.60278	7.277095	2.673159	0.37523	6.851012	-4.167456089	0.011052	0.038463	PCDHB8	5	141177790	141180529	+	2740	protein_cod	protocadherin beta 8 [Source:HGNC Symbol;Acc:HGNC:8693]
ENSG0000	0	0	0	2.891668	4.548184	7.128423	0	4.856092	-4.637614742	0.011057	0.038474	LINC02181	16	87057784	87063991	+	4083	lincRNA	long intergenic non-protein coding RNA 2181 [Source:HGNC Symbol;Acc:HGNC:53043]
ENSG0000	0	0	0	7.711115	1.819274	5.346317	0	4.958902	-4.663996163	0.011067	0.038499	AL031428.1	1	23020147	23088058	-	666	antisense	novel transcript
ENSG0000	0	7.259726	3.377067	14.45834	21.83128	12.47474	3.545598	16.25479	-2.198333116	0.011077	0.038522	PRSS37	7	141836286	141841487	-	2203	protein_cod	serine protease 37 [Source:HGNC Symbol;Acc:HGNC:29211]
ENSG0000	0	1.037104	0	6.747225	3.638547	9.801581	0.345701	6.729118	-4.145031373	0.0111	0.038597	HCG17	6	30234039	30326134	-	759	lincRNA	HLA complex group 17 [Source:HGNC Symbol;Acc:HGNC:31339]
ENSG0000	7.183155	3.111311	1.125689	21.20557	10.006	17.82106	3.806719	16.34421	-2.086160925	0.011132	0.038679	AC073592.1	12	124513222	124514813	+	1282	lincRNA	novel transcript
ENSG0000	0	0	0	5.783336	7.277095	1.782106	0	4.947512	-4.661153215	0.011146	0.038717	RPS27AP9	5	80498534	80498971	-	438	processed_p	ribosomal protein S27a pseudogene 9 [Source:HGNC Symbol;Acc:HGNC:35630]
ENSG0000	12.31398	10.37104	18.01103	26.02501	33.65656	37.42422	13.56535	32.3686	-1.261185535	0.011158	0.038749	AL353795.3	9	35038625	35044326	-	5702	TEC	TEC
ENSG0000	3.078495	6.222622	6.754134	20.24168	24.56019	11.58369	5.351751	18.79519	-1.816906464	0.011189	0.038842	AL354993.2	20	53453058	53504314	-	542	antisense	novel transcript, antisense to TSHZ2
ENSG0000	2.05233	1.037104	11.25689	22.16945	21.83128	13.36579	4.782108	19.12218	-2.022028331	0.011221	0.03895	RPL12P25	9	477750	478423	+	674	transcribed_	ribosomal protein L12 pseudogene 25 [Source:HGNC Symbol;Acc:HGNC:37008]
ENSG0000	0	3.111311	0	12.53056	11.82528	5.346317	1.037104	9.900719	-3.232431709	0.011235	0.038992	AC016074.1	8	125749055	125750241	-	636	lincRNA	uncharacterized LOC105375747 [Source:NCBI gene;Acc:105375747]
ENSG0000	1.026165	0	0	0.963889	9.096368	11.58369	0.342055	7.214648	-4.248485513	0.011237	0.038995	AL022324.3	22	25434324	25435070	-	747	lincRNA	novel transcript
ENSG0000	4.10466	16.59366	12.38258	34.70002	28.19874	25.84053	11.02697	29.57976	-1.425580421	0.011259	0.039065	ZACN	17	76071961	76083666	+	4307	protein_cod	zinc activated ion channel [Source:HGNC Symbol;Acc:HGNC:29504]
ENSG0000	4.10466	3.111311	7.879823	19.27779	20.01201	14.25685	5.031932	17.84888	-1.836738259	0.011264	0.03907	LHFPL5	6	35805293	35833874	+	2727	protein_cod	LHFPL tetraspan subfamily member 5 [Source:HGNC Symbol;Acc:HGNC:21253]
ENSG0000	1.026165	0	4.502756	6.747225	17.2831	11.58369	1.842974	11.87134	-2.721356409	0.011273	0.039095	BBIP1P1	2	101068378	101068656	+	279	processed_p	BBSome interacting protein 1 pseudogene 1 [Source:HGNC Symbol;Acc:HGNC:52781]
ENSG0000	14.36631	22.81628	5.628445	29.88057	40.93366	35.64211	14.27035	35.48545	-1.30669178	0.011352	0.039323	AC068620.3	4	56410516	56431548	+	4585	transcribed_	novel Myb/SANT-like DNA-binding domain containing pseudogene
ENSG0000	22.57563	8.29683	14.63396	27.95279	34.5662	46.33475	15.16881	36.28458	-1.258392845	0.011423	0.039527	COL26A1	7	101362820	101559024	+	3156	protein_cod	collagen type XXVI alpha 1 chain [Source:HGNC Symbol;Acc:HGNC:18038]
ENSG0000	4.10466	8.29683	4.502756	17.35001	16.37346	23.16737	5.634749	18.96361	-1.748647404	0.01146	0.039617	PHBP19	12	51124628	51125381	-	754	processed_p	prohibitin pseudogene 19 [Source:HGNC Symbol;Acc:HGNC:51552]
ENSG0000	19.49714	14.51945	6.754134	21.20557	44.5722	37.42422	13.59024	34.40066	-1.334060446	0.011476	0.039667	LRRC29	16	67207139	67227048	-	2807	protein_cod	leucine rich repeat containing 29 [Source:HGNC Symbol;Acc:HGNC:13605]
ENSG0000	2.05233	5.185518	5.628445	17.35001	21.83128	10.69263	4.288765	16.62464	-1.960956645	0.011477	0.039667	Z94160.1	22	37352190	37354839	-	1965	sense_intro	uncharacterized LOC100506271 [Source:NCBI gene;Acc:100506271]
ENSG0000	1.026165	0	2.251378	15.42223	9.096368	4.455264	1.092514	9.657954	-3.167487695	0.011531	0.03981	RNA5SP151	3	183258300	183258416	-	117	rRNA_pseuc	RNA, 5S ribosomal pseudogene 151 [Source:HGNC Symbol;Acc:HGNC:43051]
ENSG0000	2.05233	0	1.125689	10.60278	9.096368	8.019476	1.05934	9.239542	-3.122291366	0.011534	0.03981	AP000787.2	11	95145437	95146004	+	568	processed_p	splicing factor 3a, subunit 2, 66kDa, pseudogene
ENSG0000	1.026165	9.333933	3.377067	19.27779	22.74092	12.47474	4.579055	18.16448	-1.98453647	0.011562	0.039868	KLHL33	14	20425852	20436158	-	5501	protein_cod	kelch like family member 33 [Source:HGNC Symbol;Acc:HGNC:31952]
ENSG0000	0	0	0	0.963889	4.548184	9.801581	0	5.104552	-4.711159661	0.011594	0.039967	AP000654.1	11	86833068	86837615	+	1192	lincRNA	novel transcript
ENSG0000	4.10466	1.037104	4.502756	12.53056	20.01201	10.69263	3.21484	14.41174	-2.171773531	0.011627	0.040063	AC026412.2	5	1614836	1616334	+	1075	processed_p	protein phosphatase 4, regulatory subunit 2 (PPP4R2) pseudogene
ENSG0000	0	1.037104	1.125689	11.56667	8.186731	5.346317	0.720931	8.366573	-3.553767274	0.011675	0.040187	IGSF10	3	151425384	151458709	-	11727	protein_cod	immunoglobulin superfamily member 10 [Source:HGNC Symbol;Acc:HGNC:26384]
ENSG0000	1.026165	0	0	5.783336	1.819274	13.36579	0.342055	6.989467	-4.20088743	0.011695	0.040241	GAPDHP20	1	32402109	32403115	-	1007	processed_p	glyceraldehyde 3 phosphate dehydrogenase pseudogene 20 [Source:HGNC Symbol;Acc:HGNC:37772]
ENSG0000	15.39248	11.40814	4.502756	30.84446	45.48184	14.25685	10.43446	30.19438	-1.523735145	0.011732	0.040357	LOH12CR2	12	12355406	12357067	-	1540	lincRNA	loss of heterozygosity, 12, chromosomal region 2 [Source:HGNC Symbol;Acc:HGNC:26524]
ENSG0000	1.026165	0	1.125689	6.747225	10.91564	7.128423	0.717285	8.263763	-3.540974284	0.011855	0.040751	C19orf85	19	55463002	55464707	-	1292	protein_cod	chromosome 19 open reading frame 85 [Source:HGNC Symbol;Acc:HGNC:53653]
ENSG0000	1.026165	1.037104	0	10.60278	2.72891	12.47474	0.687756	8.602144	-3.615937592	0.011905	0.040894	INSC	11	15112424	15247208	+	3435	protein_cod	INSC, spindle orientation adaptor protein [Source:HGNC Symbol;Acc:HGNC:33116]
ENSG0000	17.44481	7.259726	9.005513	23.13334	28.19874	36.53317	11.23668	29.28842	-1.379486618	0.011908	0.040898	AC068870.2	15	80999593	80999981	-	389	lincRNA	novel transcript
ENSG0000	12.31398	25.92759	18.01103	0.963889	3.638547	9.801581	18.75087	4.801339	1.953100757	0.011916	0.040911	RF01241	21	31664306	31664482	+	177	snoRNA	
ENSG0000	4.10466	0	0	9.638893	10.91564	11.58369	1.36822	10.71274	-2.941219504	0.011921	0.040919	AL359878.2	10	933026	942743	+	407	antisense	uncharacterized LOC101927762 [Source:NCBI gene;Acc:101927762]
ENSG0000	0	0	0	1.927779	5.457821	7.128423	0	4.838007	-4.632865493	0.011951	0.04101	AC020765.2	16	28553709	28554140	-	432	antisense	novel transcript, antisense to CCDC101
ENSG0000	12.31398	19.70497	18.01103	45.3028	33.65656	32.0779	16.67666	37.01242	-1.150419668	0.012039	0.041275	CMYA5	5	79689877	79800240	+	14179	protein_cod	cardiomyopathy associated 5 [Source:HGNC Symbol;Acc:HGNC:14305]
ENSG0000	1.026165	1.037104	0	3.855557	7.277095	14.25685	0.687756	8.463166	-3.595664237	0.012065	0.041353	AC007431.2	17	57522248	57523597	-	563	antisense	uncharacterized LOC101927557 [Source:NCBI gene;Acc:101927557]
ENSG0000	17.44481	14.51945	5.628445	39.51946	20.92165	36.53317	12.5309	32.32476	-1.358000132	0.012091	0.041424	AC068707.2	10	43325833	43350792	-	1590	lincRNA	novel transcript
ENSG0000	42.07277	25.92759	30.3936	15.42223	11.82528	15.1479	32.79799	14.1318	1.216225717	0.012146	0.041589	RFESD	5	95646754	95684773	+	5062	protein_cod	Rieske Fe-S domain containing [Source:HGNC Symbol;Acc:HGNC:29587]
ENSG0000	8.209321	3.111311	2.251378	0	0	0	4.524003	0	4.742306836	0.012184	0.041701	TMPOP2	16	74667506	74668706	+	1201	processed_p	thymopoietin pseudogene 2 [Source:HGNC Symbol;Acc:HGNC:50860]
ENSG0000	0	0	0	2.891668	2.72891	8.910529	0	4.843702	-4.634287901	0.012188	0.041709	AC010320.5	19	52241765	52242440	-	676	processed_p	vomeronasal 1 receptor 2 (VN1R2) pseudogene

ENSG0000	4.10466	3.111311	2.251378	11.56667	16.37346	13.36579	3.155783	13.76864	-2.119455295	0.012192	0.041716	ARHGAP31-.	3	119314293	119322760	-	624	antisense	ARHGAP31 antisense RNA 1 [Source:HGNC Symbol;Acc:HGNC:41235]
ENSG0000	7.183155	4.148415	1.125689	6.747225	26.37947	19.60316	4.15242	17.57662	-2.070273555	0.012224	0.041813	AL513320.1	1	3623190	3624743	-	622	antisense	novel transcript
ENSG0000	0	13.48235	7.879823	0	0.909637	0	7.120724	0.303212	4.430571513	0.012226	0.041816	COX6CP13	1	65298755	65298980	-	226	processed_p	cytochrome c oxidase subunit 6C pseudogene 13 [Source:HGNC Symbol;Acc:HGNC:49365]
ENSG0000	23.6018	13.48235	7.879823	22.16945	39.11438	49.89896	14.98799	37.06093	-1.301192122	0.012255	0.041897	LIVAR	18	70335929	70337310	-	384	lincRNA	liver cell viability associated lncRNA [Source:HGNC Symbol;Acc:HGNC:53130]
ENSG0000	16.41864	25.92759	10.1312	73.25559	29.10838	26.73159	17.49248	43.03185	-1.291040122	0.012264	0.041916	AL669942.1	9	66047084	66055006	-	7923	processed_t	family with sequence similarity 95 member B1-like [Source:NCBI gene;Acc:105379252]
ENSG0000	1.026165	0	2.251378	13.49445	7.277095	7.128423	1.092514	9.299989	-3.114194245	0.012291	0.042002	AHCYP7	15	50417385	50418692	+	1308	processed_p	adenosylhomocysteinase pseudogene 7 [Source:HGNC Symbol;Acc:HGNC:44999]
ENSG0000	3.078495	10.37104	4.502756	23.13334	30.01801	9.801581	5.984096	20.98431	-1.806017842	0.012306	0.042036	AIMP1P2	1	172885947	172886258	-	312	processed_p	aminoacyl tRNA synthetase complex interacting multifunctional protein 1 pseudogene 2 [Source:HGNC Symbol;Acc:HGNC:43693]
ENSG0000	6.15699	11.40814	12.38258	16.38612	19.10237	50.79001	9.98257	28.7595	-1.533219409	0.012332	0.042117	AL163051.1	14	96500810	96502321	-	1512	antisense	novel transcript, antisense to PAPOLA
ENSG0000	8.209321	2.074207	7.879823	15.42223	16.37346	29.40474	6.05445	20.40015	-1.758474846	0.012342	0.042146	AL451050.2	1	167219831	167220512	-	682	lincRNA	novel transcript
ENSG0000	1.026165	4.148415	5.628445	19.27779	15.46383	10.69263	3.601008	15.14475	-2.083146575	0.012382	0.042253	AC233723.1	17	4801159	4807013	-	651	protein_cod	novel protein
ENSG0000	2.05233	1.037104	2.251378	9.638893	14.55419	8.019476	1.780271	10.73752	-2.598134887	0.012396	0.042295	CFAP54	12	96489571	96875555	+	12914	protein_cod	cilia and flagella associated protein 54 [Source:HGNC Symbol;Acc:HGNC:26456]
ENSG0000	1.026165	1.037104	1.125689	10.60278	3.638547	13.36579	1.062986	9.202374	-3.115484714	0.01251	0.042634	CYP2W1	7	983199	989640	+	2773	protein_cod	cytochrome P450 family 2 subfamily W member 1 [Source:HGNC Symbol;Acc:HGNC:20243]
ENSG0000	0	2.074207	1.125689	4.819447	21.83128	3.564211	1.066632	10.07165	-3.244534794	0.012552	0.042722	LINC02204	15	70570958	70586606	-	3206	lincRNA	long intergenic non-protein coding RNA 2204 [Source:HGNC Symbol;Acc:HGNC:53070]
ENSG0000	9.235486	24.89049	11.25689	35.6639	41.84329	30.2958	15.12762	35.93433	-1.245125465	0.01257	0.042772	XKRX	X	100913445	100929433	-	3372	protein_cod	XK related X-linked [Source:HGNC Symbol;Acc:HGNC:29845]
ENSG0000	3.078495	1.037104	1.125689	14.45834	10.91564	7.128423	1.747096	10.83413	-2.620317921	0.012599	0.042865	TMEM88B	1	1426128	1427787	+	492	protein_cod	transmembrane protein 88B [Source:HGNC Symbol;Acc:HGNC:37099]
ENSG0000	0	0	1.125689	10.60278	4.548184	4.455264	0.37523	6.53541	-4.09941403	0.012612	0.042898	AC006453.4	2	89533185	89547106	+	1252	unprocesse	otopetrin 1 (OTPO1) pseudogene
ENSG0000	2.05233	2.074207	3.377067	7.711115	11.82528	17.82106	2.501202	12.45248	-2.326020017	0.012631	0.042943	AL662890.1	6	28837869	28839006	-	709	lincRNA	novel transcript
ENSG0000	15.39248	11.40814	11.25689	14.45834	24.56019	31.18685	12.68584	30.47032	-1.261122208	0.012712	0.043181	ARIH2OS	3	48917782	48919385	-	1604	protein_cod	ariadne RBR E3 ubiquitin protein ligase 2 opposite strand [Source:HGNC Symbol;Acc:HGNC:34425]
ENSG0000	0	0	0	8.675004	0.909637	5.346317	0	4.976986	-4.668756156	0.012736	0.043241	AL049758.1	22	42853403	42853799	-	397	processed_p	chromosome 7 open reading frame 44 (C7orf44) pseudogene
ENSG0000	36.94194	18.66787	12.38258	2.891668	7.277095	10.69263	22.66413	6.953799	1.700395435	0.012741	0.043251	AC117402.1	3	110888384	110893476	-	1363	lincRNA	novel transcript
ENSG0000	6.15699	17.63076	4.502756	15.42223	43.66255	25.84053	9.43017	28.30844	-1.581008974	0.012752	0.043274	N4BP2L1	13	32400723	32428311	-	6053	protein_cod	NEDD4 binding protein 2 like 1 [Source:HGNC Symbol;Acc:HGNC:25037]
ENSG0000	12.31398	13.48235	7.879823	27.95279	21.83128	35.64211	11.22538	28.4754	-1.338662315	0.012768	0.043309	AC112722.1	4	185370725	185390928	+	587	antisense	novel transcript
ENSG0000	0	3.111311	0	14.45834	7.277095	7.128423	1.037104	9.621286	-3.190613935	0.012775	0.043322	WNK3	X	54192823	54358642	-	11472	protein_cod	WNK lysine deficient protein kinase 3 [Source:HGNC Symbol;Acc:HGNC:14543]
ENSG0000	1.026165	2.074207	0	6.747225	17.2831	4.455264	1.033457	9.495196	-3.176004063	0.012778	0.043325	EDIL3	5	83940554	84384793	-	5738	protein_cod	EGF like repeats and discoidin domains 3 [Source:HGNC Symbol;Acc:HGNC:3173]
ENSG0000	3.078495	1.037104	0	14.45834	10.006	6.23737	1.371866	10.2339	-2.870346957	0.01286	0.043575	AL353586.1	10	12877459	12965275	+	1946	antisense	novel transcript, antisense to CCDC3
ENSG0000	48.22976	49.78098	30.3936	22.16945	20.01201	20.49422	42.80145	20.89189	1.037938081	0.012867	0.043588	AL117339.5	10	38094368	38360098	+	3955	protein_cod	novel transcript
ENSG0000	3.078495	1.037104	1.125689	16.38612	8.186731	8.019476	1.747096	10.86411	-2.623769272	0.012923	0.043758	AF196972.1	X	48506523	48508838	-	653	antisense	uncharacterized LOC101927635 [Source:NCBI gene;Acc:101927635]
ENSG0000	2.05233	4.148415	1.125689	12.53056	10.91564	13.36579	2.442145	12.27067	-2.318144124	0.012942	0.043808	AL132712.1	14	103854366	103880111	-	582	lincRNA	novel transcript
ENSG0000	17.44481	21.77918	21.38809	37.59168	50.93966	36.53317	20.20403	41.68817	-1.04640889	0.012946	0.043811	SNORD19	3	52689248	52689315	+	68	snoRNA	small nucleolar RNA, C/D box 19 [Source:HGNC Symbol;Acc:HGNC:32717]
ENSG0000	4.10466	1.037104	0	7.711115	10.006	16.03895	1.713921	11.25202	-2.691341492	0.012946	0.043811	CCDC92B	17	2724411	2749504	-	885	unitary_pse	coiled-coil domain containing 92B [Source:HGNC Symbol;Acc:HGNC:52279]
ENSG0000	1.026165	8.29683	6.754134	8.675004	28.19874	23.16737	5.359043	20.01371	-1.908965348	0.012956	0.043836	AC006946.3	22	17080701	17081456	+	756	lincRNA	novel transcript
ENSG0000	9.235486	4.148415	4.502756	0.963889	0	0	5.962219	0.321296	4.177413919	0.012962	0.043852	ANXA8L1	10	46375627	46391784	+	2860	protein_cod	annexin A8 like 1 [Source:HGNC Symbol;Acc:HGNC:23334]
ENSG0000	2.05233	0	0	7.711115	8.186731	8.910529	0.68411	8.269458	-3.561940274	0.012986	0.043916	RNA5SP490	21	32563075	32563210	+	136	rRNA_pseud	RNA, 5S ribosomal pseudogene 490 [Source:HGNC Symbol;Acc:HGNC:43390]
ENSG0000	1.026165	0	0	0.963889	10.006	9.801581	0.342055	6.923825	-4.188940626	0.013006	0.043975	AC093835.1	4	147609552	147617245	-	568	antisense	novel transcript
ENSG0000	0	2.074207	0	7.711115	9.096368	8.019476	0.691402	8.275653	-3.558124356	0.013016	0.044003	AJ009632.2	21	15370500	15627342	-	8941	lincRNA	uncharacterized LOC101927745 [Source:NCBI gene;Acc:101927745]
ENSG0000	0	0	0	4.819447	4.548184	4.455264	0	4.607632	-4.559611128	0.013048	0.044093	AL391987.1	9	61861853	61862567	-	200	processed_p	novel pseudogene
ENSG0000	3.078495	1.037104	0	9.638893	7.277095	13.36579	1.371866	10.09393	-2.85320946	0.01306	0.044121	AL162615.1	20	49829125	49831085	+	1653	processed_p	cytokine-like nuclear factor n-pac (N-PAC) pseudogene
ENSG0000	10.26165	15.55656	6.754134	0.963889	3.638547	0.891053	10.85745	1.831163	2.566222315	0.013088	0.044204	Z94721.2	6	166999405	167139141	+	5310	processed_t	novel transcript
ENSG0000	20.5233	25.92759	25.89085	9.638893	1.819274	12.47474	24.11391	7.977636	1.595308627	0.013198	0.044525	HMGN1P38	15	92711808	92712110	+	303	processed_p	high mobility group nucleosome binding domain 1 pseudogene 38 [Source:HGNC Symbol;Acc:HGNC:39422]
ENSG0000	14.36631	7.259726	6.754134	26.02501	20.01201	31.18685	9.460057	25.74129	-1.438932553	0.013206	0.044544	HRH2	5	175658030	175710756	+	5679	protein_cod	histamine receptor H2 [Source:HGNC Symbol;Acc:HGNC:5183]
ENSG0000	0	0	0	4.819447	3.638547	5.346317	0	4.601437	-4.557913162	0.013269	0.044726	OPRD1	1	28812142	28871267	+	9345	protein_cod	opioid receptor delta 1 [Source:HGNC Symbol;Acc:HGNC:8153]
ENSG0000	13.34015	22.81628	10.1312	39.51946	30.92765	35.64211	15.42921	35.36308	-1.191298004	0.013275	0.044739	FAM222A-A	12	109734166	109773508	-	1068	antisense	FAM222A antisense RNA 1 [Source:HGNC Symbol;Acc:HGNC:28223]
ENSG0000	2.05233	3.111311	4.502756	17.35001	9.096368	15.1479	3.222132	13.86476	-2.113137801	0.013363	0.044992	AL022341.1	16	648473	649200	-	389	antisense	uncharacterized LOC105371038 [Source:NCBI gene;Acc:105371038]
ENSG0000	6.15699	6.222622	11.25689	16.38612	17.2831	36.53317	7.878834	23.4008	-1.579898308	0.013389	0.04506	RPL23P2	21	28997613	28998033	-	421	processed_p	ribosomal protein L23 pseudogene 2 [Source:HGNC Symbol;Acc:HGNC:10324]
ENSG0000	4.10466	9.333933	10.1312	26.02501	13.64455	30.2958	7.856598	23.32179	-1.575184963	0.013401	0.045082	C7orf61	7	100456615	100464271	-	1191	protein_cod	chromosome 7 open reading frame 61 [Source:HGNC Symbol;Acc:HGNC:22135]
ENSG0000	0	0	1.125689	11.56667	5.457821	2.673159	0.37523	6.565884	-4.10536639	0.013426	0.045155	RF00019	5	132945342	132945454	+	113	misc_RNA	
ENSG0000	0	0	0	6.747225	2.72891	4.455264	0	4.6438	-4.569426694	0.013433	0.04517	RNU6-869P	8	126504763	126504869	+	107	snRNA	RNA, U6 small nuclear 869, pseudogene [Source:HGNC Symbol;Acc:HGNC:47832]
ENSG0000	10.26165	6.222622	29.26792	2.891668	1.819274	5.346317	15.25073	3.35242	2.175706446	0.013505	0.045343	GGT6	17	4556927	4560818	-	3161	protein_cod	gamma-glutamyltransferase 6 [Source:HGNC Symbol;Acc:HGNC:26891]
ENSG0000	0	0	0	7.711115	3.638547	2.673159	0	4.674273	-4.577714707	0.013524	0.0454	AC003989.2	7	111394875	111395043	+	169	processed_p	pseudogene similar to part of adenosine deaminase, tRNA-specific 2, TAD2 homolog (S. cerevisiae) (ADAT2)
ENSG0000	4.10466	1.037104	2.251378	20.24168	3.638547	16.03895	2.464381	13.30639	-2.427788867	0.013558	0.045497	KLHDC8A	1	205336065	205357090	-	3651	protein_cod	kelch domain containing 8A [Source:HGNC Symbol;Acc:HGNC:25573]
ENSG0000	0	0	0	7.711115	2.72891	3.564211	0	4.668079	-4.576027563	0.013579	0.045552	OR7E161P	8	11928597	11933203	-	1047	transcribed_	olfactory receptor family 7 subfamily E member 161 pseudogene [Source:HGNC Symbol;Acc:HGNC:31234]
ENSG0000	0	0	0	3.855557	6.367458	3.564211	0	4.595742	-4.556397277	0.013605	0.045628	TPTE2	13	19422877	19536762	-	2287	protein_cod	transmembrane phosphoinositide 3-phosphatase and tensin homolog 2 [Source:HGNC Symbol;Acc:HGNC:17299]
ENSG0000	0	1.037104	0	12.53056	3.638547	3.564211	0.345701	6.577773	-4.107661208	0.013641	0.045741	AC010201.1	12	89371820	89372359	+	540	lincRNA	novel transcript
ENSG0000	26.68029	13.48235	13.50827	48.19447	27.2891	44.55264	17.8903	40.01207	-1.156413885	0.013673	0.045822	CGB2	19	49031912	49033238	+	859	protein_cod	chorionic gonadotropin subunit beta 2 [Source:HGNC Symbol;Acc:HGNC:16722]
ENSG0000	9.235486	0	12.38258	26.02501	22.74092	23.16737	7.206022	23.97777	-1.743760721	0.013698	0.04589	AC092171.5	7	5428731	5429672	+	942	lincRNA	novel transcript
ENSG0000	1.026165	7.259726	11.25689	21.20557	13.64455	31.18685	6.514261	22.01232	-1.769941213	0.013732	0.045977	TSKS	19	49739753	49763330	-	2391	protein_cod	testis specific serine kinase substrate [Source:HGNC Symbol;Acc:HGNC:30719]
ENSG0000	14.36631	21.77918	20.2624	37.59168	30.92765	51.68107	18.80263	40.0668	-1.093832612	0.013741	0.045996	C1orf54	1	150268200	150280916	+	1351	protein_cod	chromosome 1 open reading frame 54 [Source:HGNC Symbol;Acc:HGNC:26258]
ENSG0000	2.05233	2.074207	1.125689	9.638893	9.096368	12.47474	1.750742	10.40333	-2.563042203	0.013749	0.046015	AC017083.1	2	68252870	68253848	+	979	antisense	novel transcript
ENSG0000	2.05233	1.037104	6.754134	27.95279	5.457821	13.36579	3.281189	15.59213	-2.264972651	0.013753	0.046022	AL445483.1	1	200669507	200694250	+	736	lincRNA	uncharacterized LOC101929224 [Source:NCBI gene;Acc:101929224]
ENSG0000	0	1.037104	1.125689	6.747225	10.006	7.128423	0.720931	7.960551	-3.484546138	0.013812	0.046186	FAM178B	2	96875882	96986592	-	3545	protein_cod	family with sequence similarity 178 member B [Source:HGNC Symbol;Acc:HGNC:28036]
ENSG0000	19.49714	5.185518	7.879823	31.80835	30.01801	24.94948	10.85416	28.92528	-1.40805786	0.013974	0.046684	Z93930.2	22	28800683	28848559	+	1470	antisense	novel transcript
ENSG0000	11.28782	12.44524	4.502756	25.06112	20.01201	32.0779	9.411939	25.71701	-1.442239024	0.013977	0.046687	AC253536.6	22	23969211	23969873	+	663	lincRNA	novel transcript
ENSG0000	5.130825	4.148415	4.502756	14.45834	23.65056	11.58369	4.593999	16.56419	-1.849714633	0.014015	0.046795	C10orf143	10	130020025	130110817	-	6794	protein_cod	chromosome 10 open reading frame 143 [Source:HGNC Symbol;Acc:HGNC:48677]
ENSG0000	10.26165	6.222622	14.63396	36.62779	14.55419	32.96896	10.37274	28.05031	-1.44010039	0.014046	0.046884	PIPOX	17	28950513	29057220	+	4059	protein_cod	pipecolic acid and sarcosine oxidase [Source:HGNC Symbol;Acc:HGNC:17804]
ENSG0000	2.05233	2.074207	0	4.819447	13.64455	11.58369	1.375513	10.0159	-2.842366546	0.01407	0.04693	SLC47A1P1	17	19579943	19596058	+	1402	transcribed_	SLC47A1 pseudogene 1 [Source:HGNC Symbol;Acc:HGNC:51849]
ENSG0000	14.36631	17.63076	25.89085	5.783336	8.186731	4.455264	19.29597	6.141777	1.649044369	0.014071	0.04693	GRPR	X	16123556	16153021	+	1929	protein_cod	gastrin releasing peptide receptor [Source:HGNC Symbol;Acc:HGNC:4609]
ENSG0000	8.209321	16.59366	13.50827	4.819447	2.72891	0	12.77042	2.516119	2.359189633	0.014083	0.046963	NLRP12	19	53793603	53824394	-	5088	protein_cod	NLR family pyrin domain containing 12 [Source:HGNC Symbol;Acc:HGNC:22938]
ENSG0000	1.026165	2.074207	0	11.56667	10.006	5.346317	1.033457	8.972998	-3.091717704	0.014086	0.046967	RF00017	14	55285227	55285558	+	332	misc_RNA	
ENSG0000	0	0	0	0.963889	5.457821	8.019476	0	4.813729	-4.626299504	0.01409	0.046973	GJA9	1	38874451	38881587	-	2057	protein_cod	gap junction protein alpha 9 [Source:HGNC Symbol;Acc:HGNC:19155]

ENSG0000	8.209321	5.185518	9.005513	20.24168	24.56019	19.60316	7.466784	21.46834	-1.527108578	0.014105	0.046996	HLA-DPA1	6	33064569	33080775	-	5748	protein_cod	major histocompatibility complex, class II, DP alpha 1 [Source:HGNC Symbol;Acc:HGNC:4938]
ENSG0000	0	0	0	6.747225	5.457821	1.782106	0	4.662384	-4.574561621	0.014125	0.047051	CD6	11	60971680	61020377	+	5958	protein_cod	CD6 molecule [Source:HGNC Symbol;Acc:HGNC:1691]
ENSG0000	8.209321	13.48235	5.628445	19.27779	36.38547	20.49422	9.106705	25.38582	-1.474183177	0.014152	0.047133	NFAM1	22	42380410	42432395	-	5817	protein_cod	NFAT activating protein with ITAM motif 1 [Source:HGNC Symbol;Acc:HGNC:29872]
ENSG0000	0	0	0	7.711115	4.548184	1.782106	0	4.680468	-4.579468538	0.014156	0.047139	BNIP3P4	9	64810865	64811429	-	565	processed_p	BCL2 interacting protein 3 pseudogene 4 [Source:HGNC Symbol;Acc:HGNC:39657]
ENSG0000	0	0	0	2.891668	4.548184	6.23737	0	4.559074	-4.546314533	0.014205	0.047259	LINC00370	13	109281973	109297690	+	1337	processed_t	long intergenic non-protein coding RNA 370 [Source:HGNC Symbol;Acc:HGNC:42692]
ENSG0000	4.10466	6.222622	12.38258	13.49445	35.47584	20.49422	7.569954	23.15483	-1.62515082	0.014226	0.047319	SPDYE21P	7	67279778	67286230	+	827	unprocessec	speedy/RINGO cell cycle regulator family member E21, pseudogene [Source:HGNC Symbol;Acc:HGNC:51517]
ENSG0000	0	1.037104	1.125689	5.783336	10.91564	7.128423	0.720931	7.942467	-3.481664019	0.014285	0.047497	HSPE1P3	15	90634725	90635033	+	309	processed_p	heat shock protein family E (Hsp10) member 1 pseudogene 3 [Source:HGNC Symbol;Acc:HGNC:49322]
ENSG0000	2.05233	1.037104	4.502756	9.638893	7.277095	21.38527	2.53073	12.76709	-2.352861032	0.014292	0.047508	LINC02280	14	104661120	104665558	-	3816	lincRNA	long intergenic non-protein coding RNA 2280 [Source:HGNC Symbol;Acc:HGNC:53196]
ENSG0000	0	0	0	7.711115	1.819274	4.455264	0	4.661884	-4.574391637	0.014327	0.047612	ARF4P2	20	62872250	62873076	-	526	processed_p	ADP ribosylation factor 4 pseudogene 2 [Source:HGNC Symbol;Acc:HGNC:16599]
ENSG0000	8.209321	8.29683	11.25689	28.91668	14.55419	32.0779	9.254347	25.18292	-1.447596974	0.014368	0.047726	PRR26	10	649948	669581	+	9247	protein_cod	proline rich 26 [Source:HGNC Symbol;Acc:HGNC:30724]
ENSG0000	23.6018	24.89049	9.005513	40.48335	29.10838	59.70054	19.16593	43.09742	-1.163058451	0.014386	0.047759	GS1-124K5.4	7	66493706	66495474	+	540	lincRNA	uncharacterized LOC100289098 [Source:NCBI gene;Acc:100289098]
ENSG0000	2.05233	2.074207	5.628445	18.3139	10.006	13.36579	3.251661	13.89523	-2.109071942	0.014389	0.047762	AC034111.1	8	10050485	10054254	-	3770	antisense	novel transcript, antisense to MSRA
ENSG0000	7.183155	13.48235	11.25689	22.16945	29.10838	29.40474	10.6408	26.89419	-1.340362488	0.01439	0.047762	AC005865.1	12	3318718	3325343	+	3004	lincRNA	novel transcript
ENSG0000	7.183155	8.29683	12.38258	21.20557	26.37947	25.84053	9.287522	24.47519	-1.40473602	0.014413	0.047828	AC025682.1	17	47603860	47649420	-	1242	antisense	novel transcript, antisense to NPEPPS
ENSG0000	10.26165	7.259726	6.754134	30.84446	18.19274	19.60316	8.091837	22.88012	-1.494246991	0.014428	0.047856	LINC01431	20	23356594	23358116	-	841	lincRNA	long intergenic non-protein coding RNA 1431 [Source:HGNC Symbol;Acc:HGNC:50744]
ENSG0000	3.078495	0	7.879823	10.60278	19.10237	17.82106	3.652773	15.84207	-2.138749101	0.014429	0.047856	RUFY4	2	218034960	218090581	+	4357	protein_cod	RUN and FYVE domain containing 4 [Source:HGNC Symbol;Acc:HGNC:24804]
ENSG0000	0	0	0	3.855557	2.72891	7.128423	0	4.570964	-4.549540445	0.014476	0.047981	AC023509.2	12	53513984	53517608	+	2297	antisense	uncharacterized LOC100652999 [Source:NCBI gene;Acc:100652999]
ENSG0000	6.15699	14.51945	9.005513	19.27779	37.29511	23.16737	9.893985	26.58009	-1.426171212	0.014518	0.048086	COL14A1	8	120059780	120372036	+	8647	protein_cod	collagen type XIV alpha 1 chain [Source:HGNC Symbol;Acc:HGNC:2191]
ENSG0000	7.183155	19.70497	9.005513	2.891668	0	3.564211	11.96455	2.15196	2.477710479	0.014524	0.048098	RPL22P12	2	110633947	110634333	+	387	processed_p	ribosomal protein L22 pseudogene 12 [Source:HGNC Symbol;Acc:HGNC:36345]
ENSG0000	1.026165	0	4.502756	8.675004	6.367458	19.60316	1.842974	11.54854	-2.680746066	0.014547	0.048161	NEUROG2	4	112513516	112516172	-	2287	protein_cod	neurogenin 2 [Source:HGNC Symbol;Acc:HGNC:13805]
ENSG0000	4.10466	6.222622	15.75965	15.42223	28.19874	32.0779	8.695643	25.23296	-1.551850701	0.014567	0.048221	UFL1-AS1	6	96358374	96521669	-	548	antisense	UFL1 antisense RNA 1 [Source:HGNC Symbol;Acc:HGNC:41007]
ENSG0000	6.15699	5.185518	6.754134	10.60278	20.92165	26.73159	6.032214	19.41867	-1.691446171	0.014571	0.048224	AC007613.1	16	11741910	11744506	+	2597	antisense	novel transcript, antisense to TXNDC11
ENSG0000	6.15699	7.259726	4.502756	10.60278	27.2891	20.49422	5.973158	19.46203	-1.702091572	0.014572	0.048224	AL139384.2	13	112602828	112606417	-	3590	lincRNA	novel transcript
ENSG0000	12.31398	19.70497	11.25689	26.02501	41.84329	32.0779	14.42528	33.3154	-1.205697522	0.014577	0.048233	CIART	1	150282543	150287093	+	2696	protein_cod	circadian associated repressor of transcription [Source:HGNC Symbol;Acc:HGNC:25200]
ENSG0000	4.10466	0	2.251378	11.56667	10.91564	12.47474	2.118679	11.65235	-2.458283689	0.014624	0.048376	RPSAP74	8	89243401	89243793	-	393	processed_p	ribosomal protein SA pseudogene 74 [Source:HGNC Symbol;Acc:HGNC:51928]
ENSG0000	0	1.037104	1.125689	11.56667	9.096368	3.564211	0.720931	8.07575	-3.501877543	0.014639	0.048418	AL158801.2	14	55262767	55272075	-	748	lincRNA	novel transcript
ENSG0000	0	0	1.125689	7.711115	6.367458	4.455264	0.37523	6.177946	-4.019729699	0.014745	0.048743	PDE1C	7	31751179	32299329	-	13821	protein_cod	phosphodiesterase 1C [Source:HGNC Symbol;Acc:HGNC:8776]
ENSG0000	0	8.29683	1.125689	11.56667	11.82528	23.16737	3.14084	15.51977	-2.297575149	0.014751	0.048755	NAPSB	19	50333796	50344767	-	1915	transcribed_	napsin B aspartic peptidase, pseudogene [Source:HGNC Symbol;Acc:HGNC:13396]
ENSG0000	3.078495	6.222622	2.251378	12.53056	17.2831	15.1479	3.850832	14.98719	-1.953921394	0.014799	0.048896	FCF1P7	1	243267257	243268119	-	558	processed_p	FCF1 pseudogene 7 [Source:HGNC Symbol;Acc:HGNC:44619]
ENSG0000	1.026165	0	1.125689	5.783336	3.638547	15.1479	0.717285	8.189927	-3.529178825	0.014822	0.048951	CCDC166	8	143706694	143708109	-	1320	protein_cod	coiled-coil domain containing 166 [Source:HGNC Symbol;Acc:HGNC:41910]
ENSG0000	3.078495	2.074207	0	3.855557	5.457821	26.73159	1.717568	12.01499	-2.788197301	0.014832	0.048978	TAS2R50	12	10985913	10986912	-	1000	protein_cod	taste 2 receptor member 50 [Source:HGNC Symbol;Acc:HGNC:18882]
ENSG0000	4.10466	7.259726	4.502756	10.60278	22.74092	20.49422	5.289047	17.94597	-1.76266699	0.014841	0.049001	LINC01771	1	53328233	53336509	+	567	lincRNA	long intergenic non-protein coding RNA 1771 [Source:HGNC Symbol;Acc:HGNC:52561]
ENSG0000	3.078495	6.222622	3.377067	0	0	0	4.226062	0	4.641654455	0.014849	0.049019	AC090023.2	12	65644334	65663299	+	701	lincRNA	novel transcript
ENSG0000	3.078495	3.111311	2.251378	16.38612	11.82528	9.801581	2.813728	12.67099	-2.164795434	0.014856	0.049037	AL731533.2	10	3065424	3066001	-	578	lincRNA	novel transcript
ENSG0000	16.41864	12.44524	13.50827	42.41113	22.74092	32.96896	14.12405	32.707	-1.208978978	0.014867	0.049066	ISM1	20	13221771	13300651	+	2593	protein_cod	isthmin 1 [Source:HGNC Symbol;Acc:HGNC:16213]
ENSG0000	4.10466	10.37104	1.125689	14.45834	16.37346	25.84053	5.200462	18.89078	-1.850200455	0.014881	0.049105	Z97055.2	22	43812456	43817394	+	1981	antisense	novel transcript, antisense to EFCAB6
ENSG0000	12.31398	7.259726	6.754134	1.927779	0.909637	0.891053	8.775947	1.242823	2.832511815	0.014915	0.049205	RASL10A	22	29312933	29319679	-	2240	protein_cod	RAS like family 10 member A [Source:HGNC Symbol;Acc:HGNC:16954]
ENSG0000	1.026165	0	0	8.675004	4.548184	5.346317	0.342055	6.189835	-4.022101073	0.014954	0.049321	AP000553.4	22	21661859	21661929	-	71	unitary_pse	novel pseudogene
ENSG0000	10.26165	6.222622	5.628445	32.77224	16.37346	16.93	7.370906	22.02523	-1.571936005	0.014971	0.049368	AL022334.1	22	35501861	35503586	-	1726	processed_p	tRNA methyltransferase 11 homolog (S. cerevisiae) (TRMT11) pseudogene
ENSG0000	0	0	1.125689	4.819447	2.72891	11.58369	0.37523	6.377348	-4.068932203	0.014975	0.049373	AC091153.4	17	4731756	4732371	-	480	antisense	novel transcript, antisense to MED11
ENSG0000	6.15699	14.51945	9.005513	28.91668	33.65656	16.93	9.893985	26.50108	-1.419789575	0.015014	0.049476	FAM198A	3	42979267	43060211	+	4411	protein_cod	family with sequence similarity 198 member A [Source:HGNC Symbol;Acc:HGNC:24485]
ENSG0000	3.078495	3.111311	3.377067	25.06112	5.457821	12.47474	3.188958	14.33123	-2.165887547	0.015025	0.049499	OR7E128P	11	71893410	71894433	+	1024	unprocessec	olfactory receptor family 7 subfamily E member 128 pseudogene [Source:HGNC Symbol;Acc:HGNC:15305]
ENSG0000	0	0	0	7.711115	8.186731	0	0	5.299282	-4.759461216	0.015039	0.049533	AC092140.1	16	56409320	56411683	+	549	antisense	novel transcript, antisense to AMFR
ENSG0000	13.34015	10.37104	16.88534	26.02501	46.39148	24.05843	13.53217	32.15831	-1.252892005	0.015111	0.049748	CNIH2	11	66278190	66285301	+	2069	protein_cod	cornichon family AMPA receptor auxiliary protein 2 [Source:HGNC Symbol;Acc:HGNC:28744]
ENSG0000	4.10466	0	2.251378	7.711115	18.19274	9.801581	2.118679	11.90181	-2.489907455	0.015137	0.049826	PCDHB7	5	141172619	141176383	+	3765	protein_cod	protocadherin beta 7 [Source:HGNC Symbol;Acc:HGNC:8692]
ENSG0000	25.65413	21.77918	14.63396	35.6639	55.48785	37.42422	20.68909	42.85866	-1.046944898	0.015145	0.049847	FUZ	19	49806869	49817376	-	2950	protein_cod	fuzzy planar cell polarity protein [Source:HGNC Symbol;Acc:HGNC:26219]
ENSG0000	0	0	7.879823	15.42223	19.10237	8.910529	2.626608	14.47838	-2.499625262	0.015202	0.049973	AC009902.3	8	81275399	81277570	-	2172	lincRNA	novel transcript
ENSG0000	8.209321	6.222622	9.005513	26.9889	20.01201	18.71211	7.812485	21.90434	-1.48834161	0.015204	0.049973	LAGE3P1	9	33019682	33020165	-	484	processed_p	L antigen family member 3 pseudogene 1 [Source:HGNC Symbol;Acc:HGNC:49909]
